# National, regional, and provincial disease burden attributed to *Streptococcus pneumoniae* and *Haemophilus influenzae* type b in children in China: Modelled estimates for 2010–17

**DOI:** 10.1016/j.lanwpc.2022.100430

**Published:** 2022-03-16

**Authors:** Xiaozhen Lai, Brian Wahl, Wenzhou Yu, Tingting Xu, Haijun Zhang, Cristina Garcia, Ying Qin, Yan Guo, Zundong Yin, Maria Deloria Knoll, Hai Fang

**Affiliations:** a China Center for Health Development Studies, Peking University, Beijing, China; b Department of Health Policy and Management, School of Public Health, Peking University, Beijing, China; c Johns Hopkins India, Lucknow, India; d Department of International Health, Johns Hopkins Bloomberg School of Public Health, Baltimore, USA; e International Vaccine Access Center, Johns Hopkins Bloomberg School of Public Health, Baltimore, USA; f National Immunization Programme, Chinese Center for Disease Control and Prevention, Beijing, China; g Department of Health Policy and Management, School of Public Health, Capital Medical University, Beijing, China; h Division of Infectious Diseases, Chinese Center for Disease Control and Prevention, Beijing, China; i Peking University Health Science Center-Chinese Center for Disease Control and Prevention Joint Research Center for Vaccine Economics, Peking University, Beijing, China; j Key Laboratory of Reproductive Health, National Health Commission of the People's Republic of China, Beijing, China

**Keywords:** Immunization, *Streptococcus pneumoniae*, *Haemophilus influenzae* type b, China

## Abstract

**Background:**

Vaccination against *Streptococcus pneumoniae* (pneumococcus) and *Haemophilus influenzae* type b (Hib) is not included in China's national immunization programme. To inform China's immunization polices, we estimated annual national, regional, and provincial childhood mortality and morbidity attributable to pneumococcus and Hib in 2010–17.

**Methods:**

We estimated proportions of pneumonia and meningitis deaths and cases attributable to pneumococcus and Hib using evidence from vaccine clinical trials and surveillance studies of bacterial meningitis and pathogen-specific case fatality ratios (CFR). Then we applied the proportions to model provincial-level pneumonia cases and deaths, meningitis deaths and meningitis CFR in children aged 1–59 months, accounting for vaccine coverage. Non-pneumonia, non-meningitis (NPNM) invasive disease cases were derived by applying NPNM meningitis ratios to meningitis estimates.

**Findings:**

In 2010–17, annual pneumococcal deaths fell by 49% from 15 600 (uncertainty range: 10 800–17 300) to 8 000 (5 500–8 900), and Hib deaths fell by 56% from 6 500 (4 500–8 800) to 2 900 (2 000–3 900). Severe pneumococcal and Hib cases decreased by 16% to 218 200 (161 500–252 200) in 2017 and 29% to 49 900 (29 000–99 100). Estimated 2017 national three-dose coverage in private market was 1·3% for PCV and 33·4% for Hib vaccine among children aged 1–59 months. Provinces in the west region had the highest disease burden.

**Interpretation:**

Childhood mortality and morbidity attributable to pneumococcal and Hib has decreased in China, but still substantially varied by region and province. Higher vaccine coverage could further reduce disease burden.

**Funding:**

Bill & Melinda Gates Foundation.

**Research in context**

**Evidence before this study**

We searched PubMed in English and CNKI, Wanfang and CQVIP in Chinese for national and subnational estimates of pneumococcal and Hib morbidity and mortality in children in China from January 1, 2010 to December 31, 2019. No regional and provincial estimates of pneumococcal and Hib morbidity and mortality were identified in the literature. Estimates of annual national pneumococcal and Hib morbidity and mortality for 2000–15 among children aged 1–59 months were reported by Wahl et al. in 2018,^1^ with 7 400 (uncertainty range [UR] 5 000–8 400) pneumococcal deaths, 3400 (2 200–4 600) Hib deaths, 214 800 (158 200–249 500) severe pneumococcal cases, and 77 500 (44 900–150 600) Hib cases in 2015. A major limitation of these national estimates is that they used World Health Organization (WHO)/UNICEF WEUNIC estimates for vaccine coverage of Hib vaccine and PCV, which were both 0% in China.^1^ However, evidence suggests that a substantial proportion of children in some parts of the country receive these vaccines through the private sector, which should affect the pneumococcal and Hib estimates of mortality and morbidity in China.

**Added value of this study**

The study presents comprehensive (i.e. pneumonia, meningitis, and non-pneumonia, non-meningitis) subnational estimates of pneumococcal and Hib morbidity and mortality in children aged 1–59 months in China. In addition, we identified Hib vaccine and PCV doses administered at the provincial level to estimate subnational vaccine coverage. Given the high efficacy of these vaccines on disease outcomes, we consider our national estimates more reliable than previous estimates that assumed 0% vaccine coverage. Our estimates are based on recently published subnational cause of death and case estimates of pneumonia and meningitis in China by the Chinese Center for Disease Control and Prevention (CDC) and the Institute of Health Metrics and Evaluation. Hib vaccine and PCV coverage rates were estimated using the total doses of Hib vaccine and PCV in 31 provinces, survey results of Hib vaccine and PCV dose distributions in ten provinces, and child population data by age from National Bureau of Statistics of China and Chinese CDC immunization population database. We also incorporated new meningitis data from studies published between 2006 and 2017, including eight studies from China, and meningitis surveillance data from five sites in China from the Chinese CDC. These studies reported pathogen-specific meningitis case fatality and/or the distribution of meningitis case estimates by cause.

**Implications of all the available evidence**

Our study provides estimates of the remaining vaccine-preventable Hib and pneumococcal disease burden in China. Furthermore, subnational estimates available here highlight provinces that have not benefited from vaccination where private market use is very limited. In the provinces with sufficient private market use of PCV and Hib vaccine, our estimates provide insight into the contribution of these vaccines to the reduction of childhood morbidity and mortality associated with pneumococcal and Hib diseases. These data can be used in economic modeling to inform national and provincial policies related to expanding access to PCV and Hib vaccine to all children in China.

Alt-text: Unlabelled box

## Introduction

China has made substantial progress in improving child health outcomes in recent decades. China reduced the under-five mortality rate by 70% between 1990 and 2012, and achieved the under-five mortality rate target (i.e. two-thirds reduction in child mortality) specified by the Millennium Development Goal 4 in 2008.^2^

*Streptococcus pneumoniae* (pneumococcus) and *Haemophilus influenzae* type b (Hib) commonly cause pneumonia, meningitis, and other serious infections in children. Highly efficacious vaccines that provide protection against these pathogens are now used in many countries globally. Invasive Hib diseases in children have been virtually eliminated in countries with high routine Hib vaccine coverage.^3^, ^4^, ^5^ Vaccine-type invasive pneumococcal diseases in children have been substantially reduced where pneumococcal conjugate vaccine (PCV) has been used.^6^, ^7^, ^8^ China was estimated to be among the ten countries with the greatest number of pneumococcal and Hib deaths in children aged 1–59 months in 2000.^9^^,^^10^ Wahl, et al. estimated that China still had approximately 7 400 pneumococcal deaths and 3 400 Hib deaths in 2015.^1^

Hib vaccine and 7-valent PCV were made available in the private sector in China in 1996 and 2008, respectively. Up to June 2020, 193 of 194 countries globally have included Hib vaccine in their national immunization programmes (NIP), and PCV has been used in the NIP of 144 countries. However, China has not yet included either of these vaccines into its NIP.

Given the challenges of disease surveillance for the two pathogens, there are no directly measured estimates of pneumococcal or Hib disease burden from China. Modelled estimates can help support decision-making in the absence of empirical data. However, previous modeling studies have assumed vaccination coverage for Hib vaccine and PCV was 0%, thereby overestimating disease burden due to these pathogens. Subnational estimates are particularly useful, as provinces in China can make their own policies of adding new vaccines according to the Vaccine Management Law newly released in 2019. China is the only country in the Western Pacific Region that has not introduced Hib vaccine in its NIP and one of a handful of countries that have not introduced PCV. We used updated data, including new estimates of Hib vaccine and PCV coverage, to prepare national, regional, and provincial level estimates of Hib and pneumococcal morbidity and mortality. These data can be used for policy making in the country.

## Methods

We estimated pneumococcal and Hib specific disease burden in children aged 1–59 months at the national, regional and provincial levels in China using methods similar to those that have been previously published.^1^ In brief, we estimated the burden separately for the three clinical syndromes associated with pneumococcus and Hib: pneumonia, meningitis, and invasive non-pneumonia, non-meningitis disease (NPNM). We used the WHO 2005 case definitions for pneumonia and meningitis (WHO 2005).^11^ NPNM was considered as disease outcomes associated with the isolation of pneumococcus or Hib from normally sterile body fluid but without the clinical findings of pneumonia or meningitis. Input parameters for each model are described in Table 1.

**Table 1:** Province-specific model parameters and sources.

Model Parameter	Source of data from China	Sources of data from outside China and approach when data are not applicable
**Pathogen-specific pneumonia (lower respiratory infection)**		
All-cause pneumonia deaths	Modelled estimates based on the Global Disease Burden (GBD) study from China^1^	Not applicable
Pneumonia cases	Modelled estimates based on the Global Disease Burden (GBD) study from China^1^	Not applicable
Proportion of pneumonia cases and deaths attributable to each pathogen	Not available	Global estimates with clinical trial data^2^
**Pathogen-specific meningitis**		
All-cause meningitis deaths	Modelled estimates based on the Global Disease Burden (GBD) study from China^1^	Not applicable
Proportion of meningitis cases attributable to each pathogen	Meningitis surveillance in 5 sites^3^ and 6 studies^4^, ^5^, ^6^, ^7^, ^8^, ^9^	Meningitis surveillance from Asia: 31 studiesǂ
Pneumococcal meningitis CFR	Pneumococcal meningitis surveillance from low mortality settings: 2 studies^10^^,^^11^; medium mortality settings: 1 study^5^	Pneumococcal meningitis surveillance from low mortality settings: 37 studies; medium mortality settings: 13 studiesǂ
Hib meningitis CFR	Hib meningitis surveillance from low mortality settings: not available; medium mortality settings: not available	Hib meningitis surveillance from low mortality settings: 31 studies; medium mortality settings: 18 studiesǂ
**Pathogen-specific NPNM**		
Pneumococcal NPNM case multiplier	Severe pneumococcal disease surveillance from low or medium mortality settings: not available	Severe pneumococcal disease surveillance from low or medium mortality settings: 26 studies
Hib NPNM case multiplier	Hib disease surveillance from low or medium mortality settings: not available	Hib disease surveillance from low or medium mortality settings: 26 studies
Pneumococcal NPNM CFR multiplier	Pneumococcal disease surveillance: not available	Pneumococcal disease surveillance: 2 studies
Hib NPNM CFR multiplier	Hib disease surveillance: not available	Hib disease surveillance: 5 studies
**Population at risk and demographic model parameters**		
Child mortality	Modelled estimates based on the Global Disease Burden (GBD) study from China^1^	Not applicable
Child population	China National Bureau of Statistics in 2010-2017, Child Immunization Population Statistics in 2010-2017 from Chinese CDC	Not applicable
Access to care for meningitis	Data from China Family Panel Studies in 2010, 2012, 2014, and 2016	Linear interpolation and extrapolation to 2011, 2013, 2015, and 2017
Hib vaccine doses	Chinese CDC in 2010-2017	Not applicable
PCV doses	Chinese CDC in 2010-2017	Not applicable

1. Zhou M, Wang H, Zeng X, et al. Mortality, morbidity, and risk factors in China and its provinces, 1990-2017: a systematic analysis for the Global Burden of Disease Study 2017. *Lancet* 2019; **394**: 1145–58.		
2. Wahl B, O'Brien KL, Greenbaum A, et al. Burden of *Streptococcus pneumoniae* and *Haemophilus influenzae* type b disease in children in the era of conjugate vaccines: global, regional, and national estimates for 2000-15. *Lancet Glob Health* 2018; **6**: e744–e57.		
3. Li Y, Yin Z, Shao Z, et al. Population-based surveillance for bacterial meningitis in China, September 2006-December 2009. *Emerg Infect Dis* 2014; **20**: 61–9.		
4. Mao F, Wang J, Li J, Yu X. Aetiological spectrum and antibiotic susceptibility pattern of bacterial meningitis in infants and children in Hangzhou, China. *Acta Paediatr* 2005; **94**: 1162–3.		
5. Lin M, Dong B, Tang Z, et al. Epidemiological features of bacterial meningitis among children under 5 years old in Nanning. *South China Journal of Preventive Medicine* 2004; **30**: 30–3. In Chinese		
6. Gao J, Yi Z, Zhong J. The etiology and antibiotic resistance patterns of childhood purulent meningitis: a report of 164 cases. *Journal of Pediatric Pharmacy* 2013; **19**: 35–8. In Chinese		
7. Wang L, Tian F, Chen G, et al. Distribution of pathogenic bacteria and analysis of drug resistance of purulent meningitis in Chongqing Area from 2009 to 2013. *Journal of Pediatric Pharmacy* 2013; **19**: 37–42. In Chinese		
8. Du L, Han H, Shi K, et al. Pathogen distributions and drug resistance of children's bacterial meningitis in Taiyuan. *Journal of Clinical Medical Literatures* 2014; **11**: 2068–70. In Chinese		
9. Yang Y, Leng Z, Shen X, et al. Acute bacterial meningitis in children in Hefei, China 1990-1992. *Chin Med J* 1996; **109**: 385–8.		
10. Peng Q, Liao H, Tang J. Clinical analysis of purulent meningitis caused by *streptococcus pneumoniae* in 12 children. *Chinese Pediatric Emergency Medicine* 2013; **20**: 169–71. In Chinese		
11. Shi X, Zhang B, Li Y, et al. Analysis of clinical manifestation and serotype of 39 children with meningitis caused by *Streptococcus pneumoniae* in Suzhou. *Journal of Nantong University (Medical Sciences)* 2018; **38**: 408–12. In Chinese		

ǂ A full list of citations for studies provided in Webappendix 3 & 4.

We supplemented a previous systematic review of pneumococcal and Hib invasive disease with published and unpublished data from three Chinese-language databases (i.e. CNKI, Wanfang, CQVIP).^12^ We followed the same literature review methodology described in the previous literature review, including review procedures, inclusion criteria, exclusion criteria, quality assessment criteria and search strategy (Webappendix 2).

All analyses were done with Stata, version 15 and compliant with the Guidelines for Accurate and Transparent Health Estimates Reporting (GATHER) statement (Webappendix 1).

### Pathogen-specific pneumonia model

**Figure 1:**
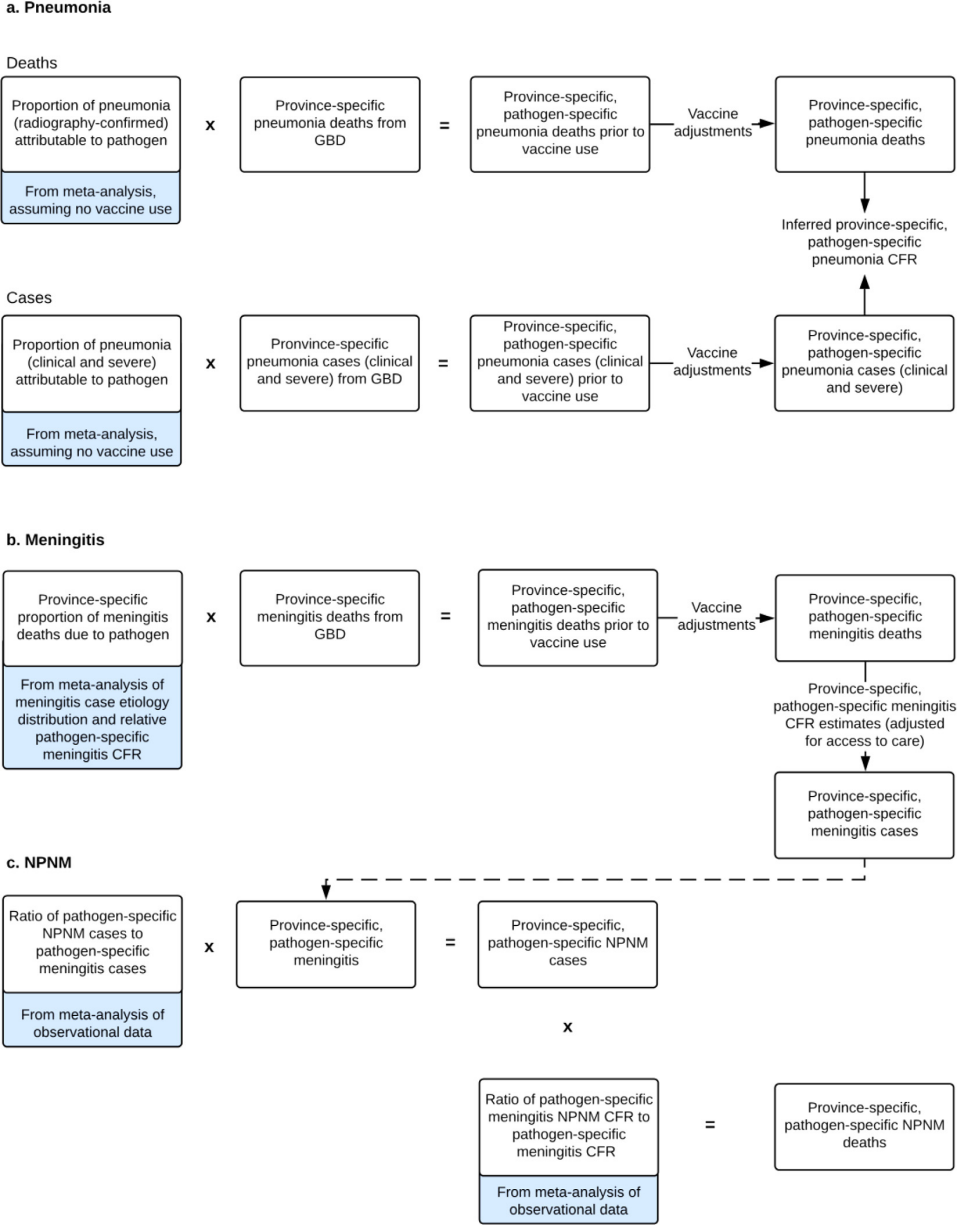
Pathogen-specific pneumonia, meningitis, and NPNM morbidity and mortality conceptual models. NPNM=non-pneumonia, non-meningitis. CFR=case-fatality ratio.

Pathogen-specific pneumonia deaths and cases were prepared by applying estimates of the proportion of pneumonia deaths and cases attributable to each pathogen to all-cause pneumonia mortality and morbidity estimates (Figure 1, A. Pneumonia). The latter were obtained from annual modelled provincial-level estimates for children aged 1–59 months in China for 2010 to 2017 prepared by Global Burden of Disease, Injuries, and Risk Factors Study (GBD),^13^ led by the Chinese CDC and Institute of Health Metrics and Evaluation. The GBD study populated the model using mortality and morbidity data for children aged 1–59 months from the Disease Surveillance Point System (including Cause of Death Reporting System) maintained by the Chinese CDC, the Maternal and Child Health Surveillance System maintained by the National Office for Maternal and Child Health Surveillance of China, and various surveys, cancer registries and censuses in China. Detailed methods for deriving these estimates are described elsewhere.^13^, ^14^, ^15^

Efficacy against pneumonia endpoints from randomized controlled PCV and Hib vaccine trials were used to estimate the attributable fraction for each pathogen.^1^ Vaccine efficacy and effectiveness estimates were combined in a random effects meta-analysis, and we used the delta method to calculate standard errors for each trial. A jackknife, leave-one-study-out approach was adopted for the upper and lower bounds of pathogen-specific pneumonia fractions to address the between-study variation in vaccine efficacy estimates (Webappendix 5). We used efficacy against radiograph-confirmed, primary endpoint pneumonia as a proxy for efficacy against pneumonia mortality, as PCV and Hib vaccine trials did not explore the efficacy against pneumonia mortality. Efficacy against clinical severe pneumonia was used to estimate pathogen-specific pneumonia morbidity. We adjusted the efficacy estimates to account for the proportion of diseases not preventable by vaccines due to non-vaccine serotypes and imperfect efficacy, as described elsewhere.^1^ Because efficacy against invasive pneumococcal disease may overestimate pneumococcal pneumonia efficacy, we performed sensitivity analyses: (1) using vaccine efficacy against nasopharyngeal carriage and (2) adjusting the proportion of pneumonia deaths due to pneumococcus by the ratio of IPD to pneumococcal pneumonia efficacy from the adult Dutch CAPiTA trial in Webappendix 12.^16^

### Pathogen-specific meningitis model

We used the proportion of meningitis cases attributable to each pathogen and pathogen-specific meningitis case fatality ratios (CFR) to estimate the proportion of meningitis deaths attributable to pneumococcus and Hib as previously described (Figure 1, B. Meningitis).^1^ For each province, we prepared summary estimates for each of these parameters by meta-analyzing data reported in literature (Webappendices 3–4). The proportion of meningitis cases attributable to each pathogen were informed by data from Asia. We stratified global data for pathogen-specific meningitis CFR by child mortality setting (i.e. <30 deaths per 1 000 livebirths, 30 to <75 deaths, and ≥75 deaths), and applied them to each province in China based on their child mortality settings.

We applied the proportion of meningitis deaths caused by pneumococcus and Hib to modelled provincial all-cause deaths of meningitis aged 1–59 months from 2010 to 2017 prepared by GBD in China to estimate pathogen-specific meningitis deaths for each province, which were divided by pathogen-specific meningitis CFR estimates to derive pneumococcal and Hib meningitis morbidity estimates.

### Pathogen-specific invasive non-pneumonia, non-meningitis model

Pathogen-specific morbidity from NPNM invasive syndromes (eg, sepsis) were estimated by applying to the meningitis case estimates the pathogen-specific NPNM to meningitis case ratios, obtained from meta-estimates of published studies globally, stratified by high or very high (i.e. >75 deaths per 1 000 live births) and low or medium (i.e. <75 deaths per 1 000 live births) all-cause child mortality, as previously described (Figure 1,C. NPNM).^1^^,^^17^ We stratified pneumococcal NPNM cases into severe and non-severe cases. For each pathogen, we estimated NPNM deaths by multiplying NPNM severe cases by the meningitis CFR and the NPNM-to-meningitis CFR ratio obtained from a meta-estimate of published literature.

### Population data

We described sources of demographic and population data for each province in Table 1. Under-five population data were retrieved from two sources: National Statistical Bureau of China and Chinese CDC. National Statistical Bureau of China collected population information in the 2010 census and in a 1% population sample survey in 2015. Chinese CDC also collected the number of children age-eligible for vaccination for each NIP vaccine from all counties and districts in China (approximately 3 000) annually in 2010–17. Provincial data for each age group were used in this study. Under-five child mortality rates in 2010–17 were obtained from the GBD in China.^13^

### Adjustment for vaccine use

Pathogen-specific pneumococcal and Hib disease burden was initially estimated assuming no vaccine use, and then adjusted to account for provincial vaccine coverage and the impact of Hib vaccine and PCV use with dose-dependent vaccine efficacy against invasive Hib diseases^10^ and serotype-specific invasive pneumococcal diseases^1^^,^^18^^,^^19^, respectively. Chinese CDC does not collect vaccine coverage of Hib vaccine and PCV as they are not in China's NIP, but each county and/or district in China (i.e. approximately 3 000 counties and districts) is required to report the number of doses for all vaccines administered to Chinese CDC, including Hib-containing vaccines, PCV7 and PCV13. We aggregated the number of Hib vaccine and PCV doses used in each county and/or district to the provincial level. In 2019, we conducted a nationally representative survey in ten provinces by collecting vaccination records of more than 6 000 children.^20^ In the vaccination records, the number of Hib vaccine and PCV doses received by each respondent was clearly written or printed. We then used dose-specific vaccine efficacy data^10^^,^^18^^,^^19^^,^^21^ to aggregate the Hib vaccine and PCV coverage by the number of doses as an equivalent three doses of these vaccines. We did a sensitivity analysis by dividing the total number of vaccine doses by three to obtain a three-doses’ coverage of Hib vaccine and PCV respectively with an assumption that all children received three doses of each vaccine (Webappendix 14). We also applied the Hib vaccine and PCV coverage rates in 2010 to calculating the disease burden in 2017, and compared the disease burden with that under the real-world coverage rates in 2017 (Webappendix 15).

### Reporting

We reported pathogen-specific mortality and incidence per 100 000 children aged 1–59 months for each year by province and region. China has 34 provinces or province-equivalent administrative regions: 31 are in Mainland China (i.e. 22 provinces, 5 autonomous regions, and 4 municipalities directly under administrative control of the central government); the other three are Taiwan, Hong Kong Special Administrative Region, and Macau Special Administrative Region, which all have different pneumococcal and Hib disease patterns and Hib vaccine and PCV policies. The present study focused on the disease burden in 31 provinces in mainland China and the term “China” refers to mainland China. Similarly, “province” refers to all province-equivalent areas, including autonomous regions or municipalities, directly administrated under the central government. To show the diversity of disease burden geographically, we used three geographically contiguous and socioeconomically distinct regions: east, central, and west according to National Statistical Bureau of China (Table 2).

**Table 2:** Chinese (mainland) provinces and characteristics by region in 2017.

Region	Province	Under 5 years population (million)	Hib vaccine coverage	PCV coverage
China		81.4	33.4	1.3
East		26.5	38.1	2.5
East	Beijing	0.9	38.6	9.9
East	Fujian	2.5	29.5	1.2
East	Guangdong	7.5	50.3	2.1
East	Jiangsu	3.8	13.2	1.7
East	Liaoning	1.4	23.6	2.0
East	Shandong	6.1	33.8	0.4
East	Shanghai	1.0	75.8	10.2
East	Tianjin	0.6	56.3	3.0
East	Zhejiang	2.7	45.2	5.6
Central		31.8	34.3	0.6
Central	Anhui	3.9	36.1	0.5
Central	Hainan	0.7	29.5	1.4
Central	Hebei	5.2	23.3	0.3
Central	Heilongjiang	1.1	32.1	1.4
Central	Henan	7.4	42.1	0.7
Central	Hubei	3.2	51.2	1.0
Central	Hunan	4.4	27.8	0.8
Central	Jiangxi	3.1	38.1	0.5
Central	Jilin	1.0	23.4	0.4
Central	Shanxi	1.8	15.8	0.2
West		23.2	26.2	0.7
West	Chongqing	1.6	47.3	2.1
West	Gansu	1.5	6.2	0.1
West	Guangxi	4.0	32.0	0.3
West	Guizhou	2.8	22.9	0.4
West	Inner Mongolia	1.0	11.4	0.0
West	Ningxia	0.5	7.5	0.4
West	Qinghai	0.4	5.0	0.0
West	Shaanxi	2.0	15.8	1.0
West	Sichuan	4.2	50.6	1.5
West	Tibet	0.3	2.4	0.0
West	Xinjiang	2.0	2.1	0.3
West	Yunnan	2.8	24.8	0.8

### Role of the funding source

The sponsor of this study had no role in study design, data collection, data analysis, data interpretation, writing of the report, or the decision to submit for publication. All authors had full access to all the data used in the study and the corresponding author had final responsibility for the decision to submit for publication.

## Results

Population-based surveillance at five sites in China to detect bacterial meningitis caused by pneumococcus, Hib, and *Neisseria meningitidis* has been implemented since 2006 by the Chinese CDC.^22^ These data were used in the model. In addition, a literature review identified eight additional studies at other Chinese sites which covered east, central and west regions in China and 29 studies from other countries in Asia that reported the distribution of pathogens among children under five years old with meningitis. Three studies in China reported pneumococcal meningitis case fatality ratios, but no study in China reported Hib meningitis case fatality ratios. Additional studies from low and medium mortality settings contributed data to estimate pathogen-specific meningitis case fatality. We did not identify any studies in China reporting pneumococcal or Hib NPNM cases or CFR multipliers; therefore, data from other countries in the global study were used.^17^ All studies are listed in Webappendices 3–4.

Hib vaccine and PCV coverage at the national level in 2017 was estimated to be 33·4% and 1·3% respectively, but varied greatly by region and province (Webappendix 14). Hib vaccine coverage was estimated to be 50% or greater mostly in economically developed areas with higher per capita Gross Regional Domestic Product rankings in 2017 (eg, Guangdong, Hubei, Shanghai, and Tianjin), but as low as 2–7% in five provinces, all in western China. Shanghai had the highest Hib vaccine coverage with 75·8% of age-eligible children in 2017 receiving three vaccine doses. PCV coverage was low in all provinces, ranging from 3·0% to 10·2% in four developed provinces and no more than 2·1% in other areas.

We estimated that the number of pneumococcal deaths in Chinese children aged 1–59 months fell from 15 600 (UR: 10 800–17 300) in 2010 (Webappendix 8) to 8 000 (5 500–8 900) in 2017 (Table 3), and Hib deaths fell from 6 500 (4 500–8 800) in 2010 (Webappendix 9) to 2 900 (2 000–3 900) in 2017 (Table 4). In 2017, the national mortality rates per 100 000 children aged 1–59 months were 10 (7–11) for pneumococcal infection and 4 (2–5) for Hib infection (Tables 3 and 4), and pneumococcus and Hib accounted for 4% and 1% of all deaths, respectively.

**Table 3:** *Streptococcus pneumoniae* mortality in Chinese children aged 1–59 months in 2017.

Province	*Streptococcus pneumoniae* deaths						*Streptococcus pneumoniae* pneumonia deaths						*Streptococcus pneumoniae* meningitis deaths						*Streptococcus pneumoniae* severe NPNM deaths					
	Number			Rate per 100 000			Number			Rate per 100 000			Number			Rate per 100 000			Number			Rate per 100 000		
	Mean	UR		Mean	UR		Mean	UR		Mean	UR		Mean	UR		Mean	UR		Mean	UR		Mean	UR	
Anhui	277	190	310	7	5	8	208	147	217	5	4	6	37	23	49	1	1	1	33	20	44	1	1	1
Beijing	45	31	50	5	3	5	36	25	37	4	3	4	5	3	7	1	0	1	4	3	6	0	0	1
Chongqing	107	74	119	7	5	7	84	60	88	5	4	5	12	8	16	1	0	1	11	7	15	1	0	1
Fujian	149	103	165	6	4	7	115	82	120	5	3	5	18	11	24	1	0	1	16	10	21	1	0	1
Gansu	284	195	318	19	13	22	211	150	220	14	10	15	39	24	52	3	2	4	35	21	46	2	1	3
Guangdong	580	399	642	8	5	9	451	320	470	6	4	6	68	42	91	1	1	1	60	37	81	1	0	1
Guangxi	290	200	319	7	5	8	234	166	243	6	4	6	30	18	40	1	0	1	26	16	36	1	0	1
Guizhou	217	151	235	8	5	8	184	131	192	7	5	7	17	11	23	1	0	1	15	9	21	1	0	1
Hainan	127	88	139	19	13	21	107	76	111	16	11	17	11	7	15	2	1	2	10	6	13	1	1	2
Hebei	583	395	666	11	8	13	386	274	402	7	5	8	104	64	140	2	1	3	93	57	124	2	1	2
Heilongjiang	101	68	115	9	6	10	67	47	69	6	4	6	18	11	24	2	1	2	16	10	22	1	1	2
Henan	461	314	523	6	4	7	318	226	332	4	3	4	76	47	101	1	1	1	67	42	90	1	1	1
Hubei	154	107	169	5	3	5	125	89	131	4	3	4	15	9	21	0	0	1	14	8	18	0	0	1
Hunan	151	105	166	3	2	4	123	87	128	3	2	3	15	9	20	0	0	0	13	8	18	0	0	0
Inner Mongolia	156	106	176	15	10	17	110	78	115	10	7	11	24	15	32	2	1	3	21	13	29	2	1	3
Jiangsu	90	61	101	2	2	3	64	46	67	2	1	2	13	8	18	0	0	0	12	7	16	0	0	0
Jiangxi	514	354	568	16	11	18	405	287	422	13	9	14	57	36	77	2	1	2	51	32	69	2	1	2
Jilin	116	79	133	12	8	14	77	54	80	8	6	8	21	13	28	2	1	3	19	11	25	2	1	3
Liaoning	47	32	53	3	2	4	32	23	34	2	2	2	8	5	10	1	0	1	7	4	9	0	0	1
Ningxia	79	54	87	17	12	19	63	45	66	14	10	15	8	5	11	2	1	2	7	5	10	2	1	2
Qinghai	135	93	149	36	25	40	107	76	112	28	20	30	15	9	20	4	2	5	13	8	18	3	2	5
Shaanxi	328	225	366	16	11	18	244	173	254	12	9	13	44	27	59	2	1	3	39	24	53	2	1	3
Shandong	158	107	181	3	2	3	100	71	105	2	1	2	30	19	41	0	0	1	27	17	36	0	0	1
Shanghai	50	34	55	5	4	6	39	28	41	4	3	4	6	3	7	1	0	1	5	3	7	1	0	1
Shanxi	269	185	302	15	10	16	198	140	206	11	8	11	38	24	51	2	1	3	34	21	45	2	1	2
Sichuan	610	424	665	15	10	16	515	365	536	12	9	13	51	31	68	1	1	2	45	28	60	1	1	1
Tianjin	48	33	53	8	6	9	38	27	40	7	5	7	5	3	7	1	1	1	5	3	6	1	1	1
Tibet	157	108	173	57	40	64	139	99	145	51	36	53	9	5	15	3	2	5	8	4	13	3	2	5
Xinjiang	782	560	879	38	28	43	676	479	704	33	24	35	56	43	93	3	2	5	50	38	83	2	2	4
Yunnan	821	571	890	29	20	31	704	499	734	25	18	26	61	38	83	2	1	3	55	34	73	2	1	3
Zhejiang	127	87	142	5	3	5	92	65	96	3	2	4	18	11	24	1	0	1	16	10	22	1	0	1
Central	2753	1886	3091	9	6	10	2013	1427	2098	6	4	7	392	242	526	1	1	2	349	216	468	1	1	1
East	1292	887	1443	5	3	5	969	687	1010	4	3	4	171	106	229	1	0	1	152	94	204	1	0	1
West	3965	2763	4377	17	12	19	3271	2320	3409	14	10	15	367	234	512	2	1	2	326	209	456	1	1	2
National	8010	5535	8912	10	7	11	6253	4434	6517	8	5	8	929	582	1267	1	1	2	827	519	1128	1	1	1

**Table 4:** *Haemophilus influenzae* type b mortality in Chinese children aged 1–59 months in 2017.

Province	Hib deaths						Hib pneumonia deaths						Hib meningitis deaths						Hib severe NPNM deaths					
	Number			Rate per 100 000			Number			Rate per 100 000			Number			Rate per 100 000			Number			Rate per 100 000		
	Mean	UR		Mean	UR		Mean	UR		Mean	UR		Mean	UR		Mean	UR		Mean	UR		Mean	UR	
Anhui	87	59	119	2	2	3	79	55	104	2	1	3	8	4	15	0	0	0	0	0	0	0	0	0
Beijing	14	10	19	2	1	2	13	9	17	1	1	2	1	1	2	0	0	0	0	0	0	0	0	0
Chongqing	30	20	40	2	1	2	27	19	36	2	1	2	2	1	4	0	0	0	0	0	0	0	0	0
Fujian	50	34	68	2	1	3	46	32	60	2	1	2	4	2	8	0	0	0	0	0	0	0	0	0
Gansu	133	90	181	9	6	12	119	84	156	8	6	11	14	7	25	1	0	2	0	0	0	0	0	0
Guangdong	136	93	184	2	1	2	125	88	164	2	1	2	11	5	20	0	0	0	0	0	0	0	0	0
Guangxi	100	69	135	2	2	3	93	65	122	2	2	3	7	3	13	0	0	0	0	0	0	0	0	0
Guizhou	86	60	116	3	2	4	82	57	107	3	2	4	5	2	8	0	0	0	0	0	0	0	0	0
Hainan	47	32	63	7	5	10	44	31	58	7	5	9	3	1	5	0	0	1	0	0	0	0	0	0
Hebei	201	135	278	4	3	5	172	121	226	3	2	4	28	13	51	1	0	1	0	0	0	0	0	0
Heilongjiang	31	21	42	3	2	4	26	19	35	2	2	3	4	2	8	0	0	1	0	0	0	0	0	0
Henan	127	86	175	2	1	2	111	78	146	1	1	2	16	8	29	0	0	0	0	0	0	0	0	0
Hubei	40	28	55	1	1	2	38	26	49	1	1	2	3	1	5	0	0	0	0	0	0	0	0	0
Hunan	56	38	75	1	1	2	52	36	68	1	1	2	4	2	7	0	0	0	0	0	0	0	0	0
Inner Mongolia	63	43	86	6	4	8	55	39	73	5	4	7	7	4	14	1	0	1	0	0	0	0	0	0
Jiangsu	35	24	48	1	1	1	31	22	41	1	1	1	4	2	7	0	0	0	0	0	0	0	0	0
Jiangxi	162	111	220	5	4	7	149	105	196	5	3	6	13	6	24	0	0	1	0	0	0	0	0	0
Jilin	38	26	53	4	3	6	33	23	43	3	2	5	5	3	10	1	0	1	0	0	0	0	0	0
Liaoning	17	11	23	1	1	2	14	10	19	1	1	1	2	1	4	0	0	0	0	0	0	0	0	0
Ningxia	36	25	49	8	6	11	34	24	44	7	5	10	3	1	5	1	0	1	0	0	0	0	0	0
Qinghai	63	43	85	17	11	22	58	41	76	15	11	20	5	2	9	1	1	2	0	0	0	0	0	0
Shaanxi	133	91	182	7	5	9	120	84	158	6	4	8	13	6	24	1	0	1	0	0	0	0	0	0
Shandong	46	31	64	1	1	1	39	27	51	1	0	1	7	3	13	0	0	0	0	0	0	0	0	0
Shanghai	7	5	9	1	0	1	6	4	8	1	0	1	1	0	1	0	0	0	0	0	0	0	0	0
Shanxi	112	76	154	6	4	8	100	70	132	5	4	7	12	6	22	1	0	1	0	0	0	0	0	0
Sichuan	164	113	220	4	3	5	154	108	203	4	3	5	9	4	17	0	0	0	0	0	0	0	0	0
Tianjin	11	7	15	2	1	3	10	7	13	2	1	2	1	0	2	0	0	0	0	0	0	0	0	0
Tibet	84	57	112	31	21	41	77	54	102	28	20	37	7	3	10	2	1	4	0	0	0	0	0	0
Xinjiang	423	285	554	21	14	27	388	273	510	19	13	25	35	11	44	2	1	2	0	0	0	0	0	0
Yunnan	321	222	430	11	8	15	305	215	401	11	8	14	16	8	29	1	0	1	0	0	0	0	0	0
Zhejiang	34	23	46	1	1	2	30	21	39	1	1	1	4	2	6	0	0	0	0	0	0	0	0	0
Central	902	612	1233	3	2	4	804	565	1056	3	2	3	97	46	175	0	0	1	1	0	1	0	0	0
East	349	238	476	1	1	2	314	221	413	1	1	2	35	16	62	0	0	0	0	0	0	0	0	0
West	1636	1117	2191	7	5	9	1512	1064	1987	7	5	9	123	53	202	1	0	1	1	0	2	0	0	0
National	2888	1966	3900	4	2	5	2631	1850	3457	3	2	4	255	115	440	0	0	1	2	1	4	0	0	0

**Figure 2:**
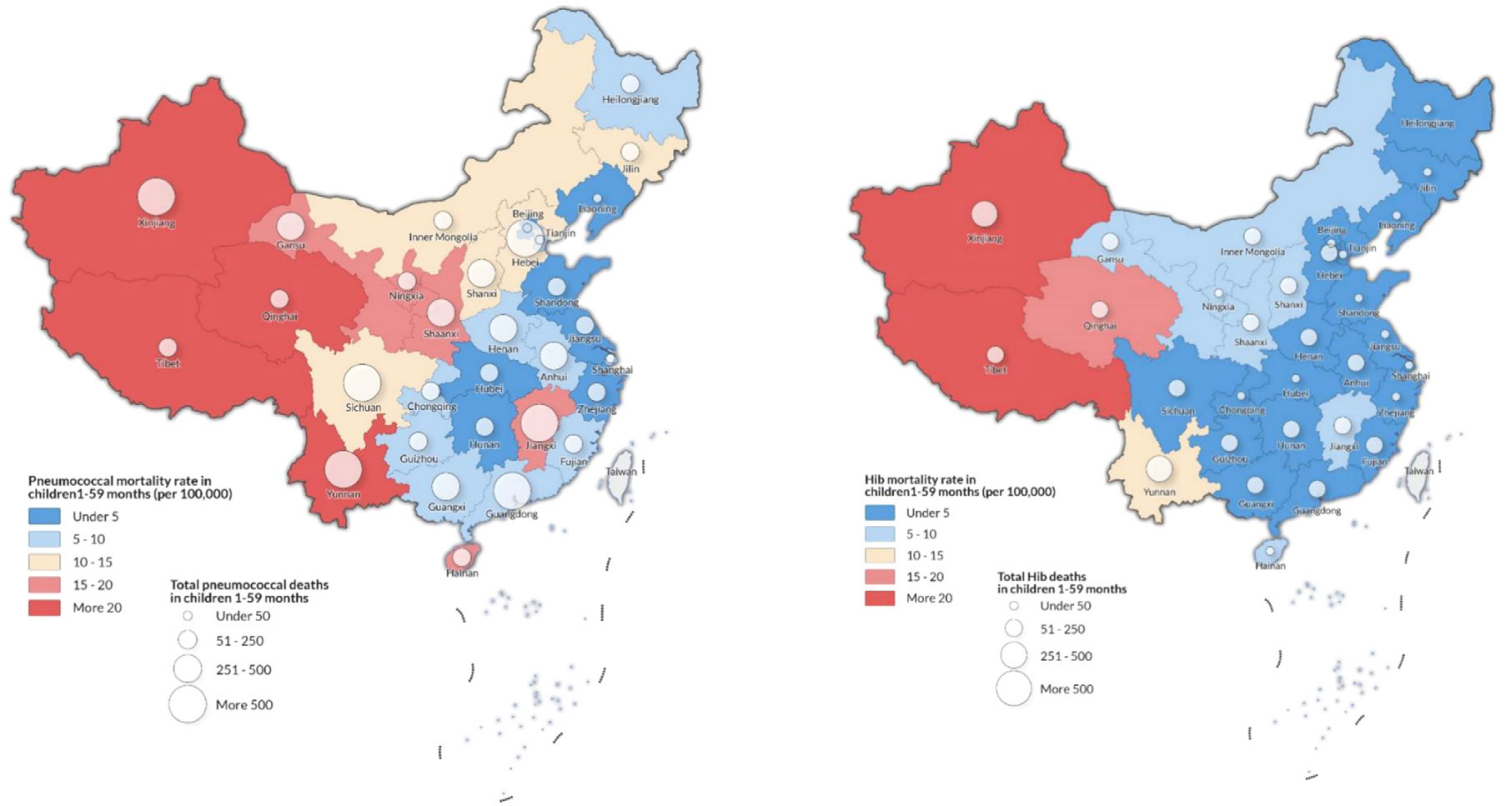
Estimated *Streptococcus pneumoniae* (A) and *Haemophilus influenzae* type b (B) deaths and mortality in 2017. Size of bubble indicates absolute number of pathogen-specific deaths.

**Figure 3:**
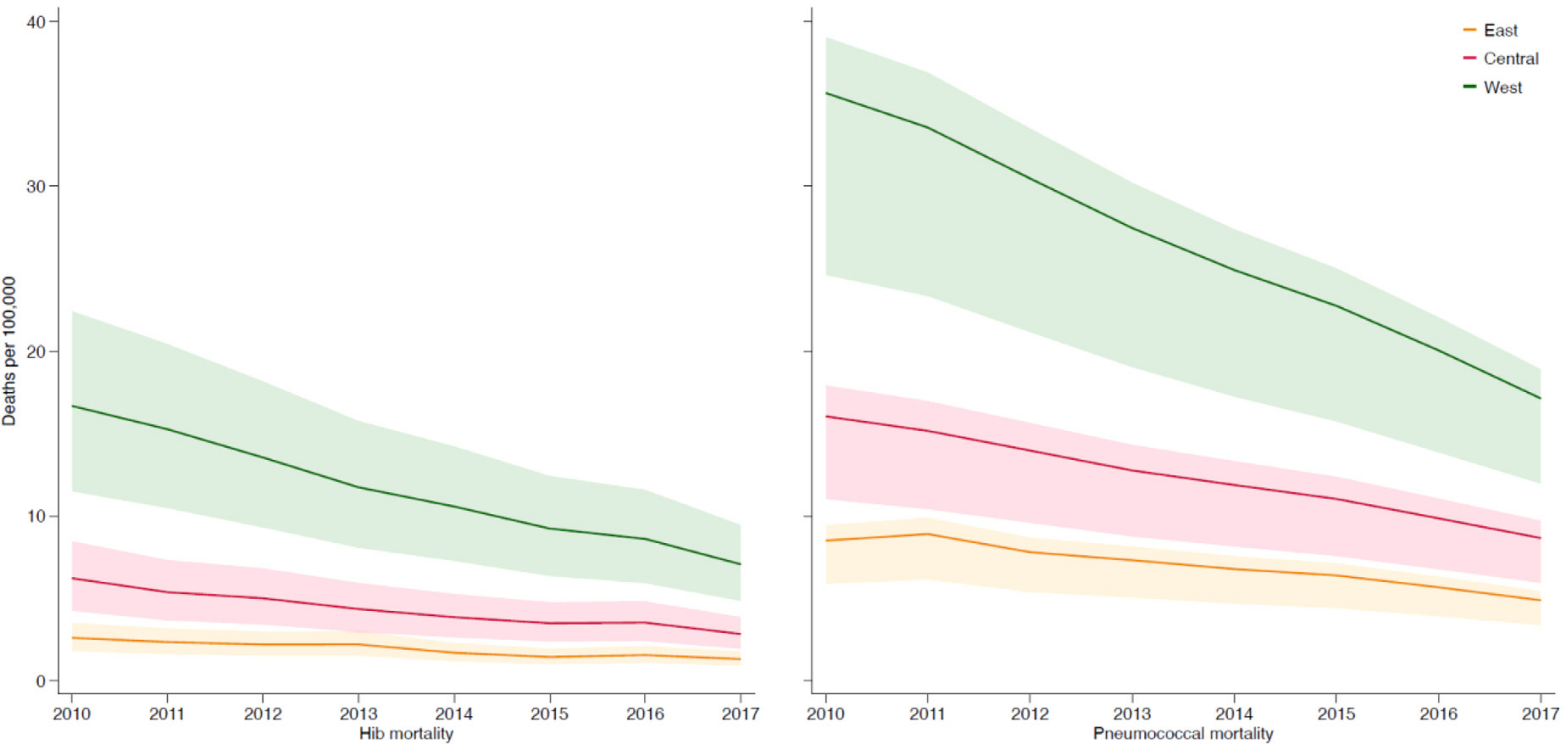
Mortality rates for *Streptococcus pneumoniae* and *Haemophilus influenzae* type b for 2010–2017, by region.

Pneumococcal and Hib mortality in China differed by region. In 2017, we estimated that 49% of pneumococcal deaths (3 600 [UR: 2 500–4 000]) and 67% of Hib deaths (1 200 [800–1 600]) occurred in the west region, which accounted for only 28% of the child population in that year (Table 3). The west region had the highest estimated pneumococcal mortality in 2017, with approximately 17 (12–19) deaths per 100 000 children aged 1–59 months, more than two times the pneumococcal mortality in the rest of China (8 [5–9]) (Table 3, Figure 2). Although the west region had the largest reduction of pneumococcal and Hib deaths from 2010 to 2017 (pneumococcal mortality declined from 36 [25–39] per 100 000 in 2010 to 17 [12–19] in 2017 and Hib mortality declined from 17 [11–22] to 7 [5–9]), the west region still had an estimated higher pneumococcal and Hib mortality in 2017 compared to other regions (Figure 3).

We reported provincial-level pneumococcal and Hib deaths and mortality rates in 2017 in Table 3 and Figure 2. Yunan province, with only 4% of under-five population in China, had the largest number of pneumococcal deaths (800 [UR: 600–900]) and the second largest Hib deaths (300 [200–400]), representing 10% and 11% of all pneumococcal and Hib deaths in China, respectively. Tibet had the highest pneumococcal mortality rate in China with 57 [UR: 40–64] per 100 000 children aged 1–59 months. Qinghai and Xinjiang also had pneumococcal mortality rates more than 30 per 100 000 aged 1–59 months. Hib deaths and mortality rates in 2017 followed the similar pattern as those for pneumococcus. In 2017, six provinces with the lowest Hib coverage in China were all in the west region: Xinjiang, Tibet, Qinghai, Gansu, Ningxia, and Inner Mongolia. Together, these provinces accounted for 28% of Hib deaths in China in 2017 despite only accounting for 7% of under-five population.

Pneumonia accounted for 78% and 91% of pneumococcal and Hib mortality, respectively, among all the syndromes associated with these two pathogens. Pneumonia was the most common syndrome associated with pneumococcus and Hib in each province between 2000 and 2017.

Pneumococcal cases and incidence rates were reported in Table 5 and Webappendix 10. Clinical pneumococcal pneumonia cases in China decreased from 633 400 (UR: 546 500–753 000) in 2010 to 553 000 (477 100–657 400) in 2017. In 2017, Guangdong province had the largest number of pneumococcal pneumonia cases of 66 500 (57 400–79 100), and Tibet had the highest pneumococcal pneumonia incidence rate of 1 000 (900–1 200) per 100 000 children aged 1–59 months.

**Table 5:** *Streptococcus pneumoniae* cases in Chinese children aged 1–59 months in 2017.

Province	*Streptococcus pneumoniae* pneumonia cases						*Streptococcus pneumoniae* severe pneumonia cases						*Streptococcus pneumoniae* meningitis cases^a^						*Streptococcus pneumoniae* severe NPNM cases					
	Number			Rate per 100 000			Number			Rate per 100 000			Number			Rate per 100 000			Number			Rate per 100 000		
	Mean	UR		Mean	UR		Mean	UR		Mean	UR		Mean	UR		Mean	UR		Mean	UR		Mean	UR	
Anhui	23379	20172	27791	605	522	719	8572	6422	9777	222	166	253	293	181	393	8	5	10	333	206	446	9	5	12
Beijing	7600	6558	9035	812	700	965	2787	2088	3178	298	223	339	39	24	53	4	3	6	45	28	60	5	3	6
Chongqing	11714	10107	13925	714	616	849	4295	3218	4899	262	196	298	98	61	132	6	4	8	111	69	150	7	4	9
Fujian	14825	12791	17622	598	516	710	5435	4072	6199	219	164	250	141	87	189	6	4	8	160	99	215	6	4	9
Gansu	10968	9463	13038	745	643	885	4021	3013	4587	273	205	311	310	192	417	21	13	28	353	218	473	24	15	32
Guangdong	66523	57398	79077	885	764	1052	24390	18273	27819	324	243	370	542	335	727	7	4	10	615	381	826	8	5	11
Guangxi	24189	20870	28753	601	518	714	8868	6644	10115	220	165	251	238	147	319	6	4	8	270	167	362	7	4	9
Guizhou	15883	13704	18880	562	485	668	5823	4363	6642	206	154	235	137	85	184	5	3	7	156	96	209	6	3	7
Hainan	4410	3805	5243	669	577	795	1617	1211	1844	245	184	280	87	54	117	13	8	18	99	61	133	15	9	20
Hebei	34605	29858	41135	669	577	795	12687	9505	14471	245	184	280	831	515	1116	16	10	22	944	584	1267	18	11	24
Heilongjiang	9173	7914	10904	835	720	992	3363	2520	3836	306	229	349	144	89	193	13	8	18	163	101	219	15	9	20
Henan	36801	31753	43746	496	428	590	13493	10109	15390	182	136	207	604	374	810	8	5	11	686	424	920	9	6	12
Hubei	18328	15813	21786	568	490	676	6720	5034	7664	208	156	238	122	76	164	4	2	5	139	86	186	4	3	6
Hunan	23108	19938	27469	521	449	619	8472	6347	9663	191	143	218	119	74	160	3	2	4	135	84	181	3	2	4
Inner Mongolia	8772	7569	10427	835	721	993	3216	2409	3668	306	229	349	193	119	258	18	11	25	219	135	293	21	13	28
Jiangsu	23698	20447	28170	619	534	736	8688	6509	9910	227	170	259	107	66	144	3	2	4	122	75	163	3	2	4
Jiangxi	19089	16471	22692	611	527	727	6999	5243	7983	224	168	256	458	284	615	15	9	20	521	322	699	17	10	22
Jilin	8762	7560	10416	918	792	1091	3213	2407	3664	336	252	384	166	103	223	17	11	23	189	117	254	20	12	27
Liaoning	12264	10582	14579	872	753	1037	4497	3369	5129	320	240	365	60	37	81	4	3	6	69	42	92	5	3	7
Ningxia	3103	2678	3689	688	594	818	1138	852	1298	252	189	288	66	41	89	15	9	20	75	47	101	17	10	22
Qinghai	2968	2561	3528	788	680	937	1088	815	1241	289	216	330	118	73	159	31	19	42	134	83	180	36	22	48
Shaanxi	13118	11319	15594	656	566	780	4810	3603	5486	240	180	274	353	218	473	18	11	24	401	248	538	20	12	27
Shandong	31314	27019	37224	515	445	613	11481	8601	13095	189	142	216	242	150	324	4	2	5	274	170	368	5	3	6
Shanghai	7853	6776	9335	822	710	978	2879	2157	3284	301	226	344	44	27	60	5	3	6	50	31	68	5	3	7
Shanxi	12895	11126	15329	698	602	829	4728	3542	5392	256	192	292	304	188	408	16	10	22	345	214	463	19	12	25
Sichuan	35875	30954	42645	863	745	1026	13153	9854	15002	316	237	361	403	250	541	10	6	13	458	284	615	11	7	15
Tianjin	4743	4092	5638	823	710	978	1739	1303	1983	302	226	344	43	27	58	7	5	10	49	30	65	8	5	11
Tibet	2738	2362	3255	1003	865	1192	1004	752	1145	368	276	419	33	18	53	12	7	19	37	20	60	14	7	22
Xinjiang	14296	12335	16994	703	607	836	5241	3927	5978	258	193	294	291	222	481	14	11	24	330	252	546	16	12	27
Yunnan	27765	23956	33005	976	842	1160	10180	7626	11611	358	268	408	491	304	659	17	11	23	558	345	749	20	12	26
Zhejiang	22278	19222	26482	829	715	986	8168	6119	9316	304	228	347	146	90	195	5	3	7	165	102	222	6	4	8
Central	190550	164411	226510	599	517	712	69862	52340	79684	220	165	251	3129	1936	4198	10	6	13	3554	2199	4768	11	7	15
East	191099	164884	227163	722	623	858	70063	52491	79914	265	198	302	1364	844	1831	5	3	7	1550	959	2079	6	4	8
West	171388	147877	203732	740	639	880	62837	47077	71671	271	203	310	2731	1729	3764	12	7	16	3102	1964	4275	13	8	18
National	553037	477172	657405	679	586	807	202762	151908	231270	249	187	284	7225	4510	9793	9	6	12	8206	5123	11123	10	6	14

a All *Streptococcus pneumoniae* meningitis cases are severe.

We estimated 218 200 (UR: 161 500–252 200) cases of severe pneumococcus (i.e. severe pneumococcal pneumonia, pneumococcal meningitis, and severe pneumococcal NPNM) in 2017, and severe pneumococcal pneumonia accounted for 93% of all the severe pneumococcal cases. Guangdong (25 500 [19 000–29 400]), Henan (14 800 [10 900–17 100]), and Hebei (14 500 [10 600–16 900]) had the highest number of estimated severe pneumococcal cases for children aged 1–59 months in 2017, and these three provinces also had relatively larger provincial population in China.

Hib cases and incidence rates were reported in Table 6 and Webappendix 11. We estimated that the number of Hib pneumonia cases decreased from 325 400 (UR: 297 100–531 600) in 2010 to 245 100 (223 800–400 500) in 2017. The number of severe Hib cases, including severe Hib pneumonia, Hib meningitis, and severe Hib NPNM, was estimated to decrease from 70 500 (40 600–139 400) in 2010 to 49 900 (29 000–99 100) in 2017, with a reduction of 29%. In 2017, severe Hib pneumonia accounted for 86% of all severe Hib cases.

**Table 6:** *Haemophilus influenzae* type b cases in Chinese children aged 1–59 months in 2017.

Province	Hib pneumonia cases						Hib severe pneumonia cases						Hib meningitis cases^a^						Hib severe NPNM cases^b^					
	Number			Rate per 100 000			Number			Rate per 100 000			Number			Rate per 100 000			Number			Rate per 100 000		
	Mean	UR		Mean	UR		Mean	UR		Mean	UR		Mean	UR		Mean	UR		Mean	UR		Mean	UR	
Anhui	9966	9099	16283	258	236	421	1751	1048	3533	45	27	91	189	89	342	5	2	9	64	30	115	2	1	3
Beijing	3276	2991	5352	350	319	572	575	345	1161	61	37	124	26	12	47	3	1	5	9	4	16	1	0	2
Chongqing	4227	3859	6907	258	235	421	743	445	1499	45	27	91	55	26	99	3	2	6	19	9	34	1	1	2
Fujian	6952	6348	11359	280	256	458	1221	731	2465	49	29	99	95	45	171	4	2	7	32	15	58	1	1	2
Gansu	6685	6103	10922	454	415	742	1174	703	2370	80	48	161	314	148	567	21	10	39	106	50	191	7	3	13
Guangdong	22747	20769	37167	303	276	494	3996	2392	8065	53	32	107	254	119	458	3	2	6	86	40	155	1	1	2
Guangxi	10905	9957	17818	271	247	442	1916	1147	3866	48	28	96	162	76	293	4	2	7	55	26	99	1	1	2
Guizhou	8060	7359	13170	285	261	466	1416	848	2858	50	30	101	104	49	189	4	2	7	35	17	64	1	1	2
Hainan	2070	1890	3381	314	286	513	364	218	734	55	33	111	63	29	113	9	4	17	21	10	38	3	2	6
Hebei	17463	15944	28533	337	308	551	3068	1837	6191	59	35	120	640	302	1157	12	6	22	216	102	390	4	2	8
Heilongjiang	4157	3795	6792	378	345	618	730	437	1474	66	40	134	98	46	177	9	4	16	33	16	60	3	1	5
Henan	14348	13100	23444	193	177	316	2520	1509	5087	34	20	69	362	170	653	5	2	9	122	57	220	2	1	3
Hubei	6126	5593	10009	190	173	310	1076	644	2172	33	20	67	64	30	115	2	1	4	21	10	39	1	0	1
Hunan	11036	10076	18032	249	227	406	1939	1161	3913	44	26	88	85	40	154	2	1	3	29	14	52	1	0	1
Inner Mongolia	5060	4620	8267	482	440	787	889	532	1794	85	51	171	169	80	305	16	8	29	57	27	103	5	3	10
Jiangsu	13543	12365	22128	354	323	578	2379	1424	4802	62	37	125	86	40	155	2	1	4	29	14	52	1	0	1
Jiangxi	7900	7213	12908	253	231	413	1388	831	2801	44	27	90	295	139	533	9	4	17	99	47	180	3	1	6
Jilin	4419	4034	7219	463	423	756	776	465	1567	81	49	164	123	58	221	13	6	23	41	19	75	4	2	8
Liaoning	6228	5687	10176	443	404	724	1094	655	2208	78	47	157	46	22	84	3	2	6	16	7	28	1	1	2
Ningxia	1869	1707	3054	414	378	677	328	197	663	73	44	147	61	29	110	14	6	24	21	10	37	5	2	8
Qinghai	1829	1670	2988	486	443	793	321	192	648	85	51	172	109	51	197	29	14	52	37	17	67	10	5	18
Shaanxi	7257	6626	11858	363	331	593	1275	763	2573	64	38	129	298	141	539	15	7	27	101	47	182	5	2	9
Shandong	13782	12584	22519	227	207	371	2421	1450	4886	40	24	80	160	75	289	3	1	5	54	25	97	1	0	2
Shanghai	1511	1379	2468	158	144	258	265	159	536	28	17	56	12	6	22	1	1	2	4	2	7	0	0	1
Shanxi	7098	6481	11598	384	351	627	1247	747	2517	67	40	136	274	129	494	15	7	27	92	43	167	5	2	9
Sichuan	12150	11094	19853	292	267	478	2134	1278	4308	51	31	104	213	100	385	5	2	9	72	34	130	2	1	3
Tianjin	1451	1325	2371	252	230	411	255	153	514	44	26	89	20	9	36	3	2	6	7	3	12	1	1	2
Tibet	1732	1581	2829	634	579	1037	304	182	614	111	67	225	31	14	48	11	5	18	10	5	16	4	2	6
Xinjiang	9075	8285	14827	446	407	729	1594	954	3217	78	47	158	289	95	366	14	5	18	97	32	123	5	2	6
Yunnan	13772	12574	22501	484	442	791	2419	1448	4883	85	51	172	362	170	653	13	6	23	122	57	220	4	2	8
Zhejiang	8433	7700	13779	314	287	513	1481	887	2990	55	33	111	80	38	145	3	1	5	27	13	49	1	0	2
Central	84582	77225	138198	266	243	435	14858	8896	29988	47	28	94	2192	1032	3960	7	3	12	739	348	1335	2	1	4
East	77924	71146	127320	294	269	481	13688	8196	27628	52	31	104	779	367	1407	3	1	5	263	124	474	1	0	2
West	82621	75435	134995	357	326	583	14513	8690	29293	63	38	127	2168	980	3753	9	4	16	731	330	1265	3	1	5
National	245127	223806	400514	301	275	492	43059	25781	86909	53	32	107	5139	2379	9120	6	3	11	1732	802	3074	2	1	4

a All Hib meningitis cases are severe.

b All Hib NPNM cases are severe.

Our disease burden estimates for both pathogens would vary with changes in estimates for all-cause pneumonia mortality and the fraction of pneumonia deaths associated with each pathogen. We reported the results of sensitivity analyses in Webappendix 13 using alternative sources of all-cause pneumonia at the national level. Based on these sensitivity analyses, pneumonia deaths could range from 4 700 (UR: 3 300–4 900) to 8 900 (6 300–9 300) for pneumococcus and 2 000 (1 400–2 600) to 5 500 (3 900–7 200) for Hib. In addition, in the probe approach, we used estimates of vaccine-type efficacy against invasive diseases to estimate the proportion of pneumonia deaths attributable to pneumococcus. Our sensitivity analyses using alternative estimates serotype-specific vaccine efficacy indicated that our approach might underestimate the contribution of pneumococcus to deaths from pneumonia. Specifically, we found that there might have been 12 000 (7 300–12 900) pneumococcal pneumonia deaths in 2017.

To quantify the effects of increased vaccine coverage over years, we also calculated the disease burden in 2017 by adopting the same three-dose vaccine coverage rates in 2010 (Webappendix 15). Nationally in the year of 2017, when increasing Hib vaccine coverage from 21.6% (2010) to 33.4% (2017), and PCV coverage from 0.5% (2010) to 1.3% (2017), pneumococcal deaths, Hib deaths, pneumococcal cases and Hib cases would decrease by 0.4% (8 043 to 8 010), 12.9% (3 316 to 2 888), 0.8% (557 241 to 553 037) and 13.7% (284 046 to 245 127), respectively.

## Discussion

The present study estimated national, regional and provincial burden of pneumococcal and Hib pneumonia, meningitis, and NPNM in China in children aged 1–59 months. Even though PCV and Hib vaccine are not currently included in China's NIP, pneumococcal and Hib deaths declined from 2010 to 2017. These declines are likely attributable to the scale-up and effective delivery of maternal and child health programmes. Our pathogen-specific morbidity and mortality estimates can be used to further monitor maternal and child programmes in China.

In 2010–17, estimated Hib vaccine coverage in China increased from 21.6% to 33.4%, and Hib deaths and cases reduced by 56% and 26%, respectively. Pneumococcal deaths and cases declined by 49% and 14% in 2010–17, while PCV coverage rate did not increase substantially, its effect on pneumococcal mortality and morbidity was marginal.

The reduction in deaths attributable to Hib and pneumococcus reflects overall child survival trends in China during 2010–17. China's socioeconomic development (i.e. GDP growth, female education, improved access to healthcare, health insurance coverage) and China's maternal and health interventions and programmes (i.e. equal access to basic public health services, free health checks for children aged 0–6 years) contributed to the decline of child mortality, including pneumonia.^23^ Vaccination is seldom taken into account in such models, so its contribution to mortality decline has not been recognized until now.

We estimated that a disproportionate number of pneumococcal and Hib deaths occurred in some regions. The west region had more pneumococcal and Hib deaths and higher mortality rates compared to the east and central regions, despite it only accounts for 28% of the population in 2017. The west region is less developed, with poorer socioeconomic development indicators, and lower PCV and Hib vaccine coverage in the private sector. Qinghai, Xinjiang, and Tibet had the highest Hib mortality rates per 100 000 children in 2017 in China, and these three provinces also had the lowest Hib vaccine coverage. However, many of the government-launched maternal and child health programmes have focused on this region in the past two decades. These efforts might have contributed to the largest reduction of child mortality between 2010 and 2017 in China. The west region will achieve even higher child mortality reductions if Hib vaccine and PCV are included in the NIP.

China has a large population and there are substantial socioeconomic, cultural, and geographic differences across the country. Our subnational model enables us to assess province-level differences in vaccine coverage and their effects on disease outcomes. In 2008, China expanded its NIP from 6 vaccines to 15 vaccines. However, Hib vaccine and PCV were not included in part due to their high costs. Since then, China has not added any new vaccines into its NIP. Our estimates of pneumococcal and Hib disease burden provide new evidence to support the introduction of these vaccines into China's NIP. Provincial governments in China are empowered to include specific vaccines into local immunization programmes. For example, seasonal flu vaccine and pneumococcal polysaccharide vaccine (PPSV23) have been provided to the elderly for free in several provinces and municipalities.^24^^,^^25^ Our provincial estimates could therefore inform local policies regarding Hib vaccine and PCV.

As China is the most populous country in the world, the reported Hib and pneumococcal disease burden in this country is also of important reference value, especially for nearby areas with similar epidemiological characteristics. China is also the only country which has not included Hib vaccine in its NIP despite recommendations from the WHO to include in all infant immunization programs.^26^ The findings can help ensure all countries in the region are using this lifesaving vaccine. Moreover, some countries in the Western Pacific region have not yet included PCV in their NIPs, including Nauru, Tonga, Tuvalu, Vanuatu and Viet Nam. The present analysis in China might help decision-making related to vaccination in these countries and therefore support global and regional efforts to reduce disease burden due to pneumococcus.

Wahl et al. estimated that China had 7 400 (5 000–8 400) pneumococcal deaths, 3 400 (2 200–4 600) Hib deaths, 214 800 (158 200–249 500) severe pneumococcal cases, and 77 500 (44 900–150 600) severe Hib cases at the national level in 2015.^1^ The present study estimated that there were 10 200 (UR: 7 000–11 400) pneumococcal deaths, 3 600 (2 400–4 800) Hib deaths, 230 500 (170 100–267 100) severe pneumococcal cases, and 50 100 (28 900–99 200) severe Hib cases at the national level in 2015 (reported in Webappendix 10 and 11). The estimation approach adopted by Wahl et al. and the present study are similar; however, the differences in these estimates may lie in the inclusion of vaccine coverage data, subnational estimates, and new estimates of all-cause pneumonia and meningitis mortality.

Additionally, in the present study, we provide estimates of disease burden for Hib and pneumococcus for 2010–2017. Provincial-level all-cause pneumonia and meningitis disease burden data after 2017 were not available. We further calculated the national-level pathogen-specific disease burden for 2018 and 2019 using available national-level data from GBD IHME in Webappendix 16, which may provide more up-to-date references despite the lack of provincial-level estimates.

Our analysis has several limitations, many of which have been described in detail elsewhere.^1^ First, the pathogen-specific pneumonia models used data from vaccine clinical trials conducted in several countries around the world, none of which were done in China and only one was from the Western Pacific Region.^27^ Applying these proportions derived from studies done in other settings could result in biased estimates. In addition, we used vaccine-type invasive pneumococcal disease efficacy as a proxy for vaccine-type pneumococcal pneumonia efficacy when estimating the proportion of pneumococcus pneumonia deaths, which might underestimate the contribution of pneumococcus to pneumonia deaths. To address this limitation, we conducted a sensitivity analysis and found that pneumococcal pneumonia deaths could be roughly twice the estimates from our base case model. Another limitation is that we were unable to account for some underlying risk factors, including HIV infection and sickle cell; however, given their low overall prevalence in China, the impact on our models would likely be modest. Our models also did not account for outbreaks of pathogen-specific meningitis, which might underestimate Hib and pneumococcal meningitis disease burden. Last, there were no data from China on pathogen-specific NPNM, so data from studies done in geographically and epidemiologically relevant settings were used. It would help improve disease burden estimates if additional pathogen-specific data were available from observational studies in China.

According to our subnational estimates, China has made substantial progress in reducing mortality and morbidity caused by pneumococcus and Hib in 2010–17, but there are still severe regional and provincial disparities. These achievements were made with other childhood interventions, while the impacts of PCV and Hib vaccine was limited, even if both have been available in the private market for a long time. Introducing PCV and Hib vaccine into NIP or specific provinces has the potential to substantially accelerate the reduction of childhood pneumococcal and Hib morbidity and mortality towards meeting the Sustainable Development Goal child survival targets by 2030.

## Contributors

XL, BW, and WY did the analyses for the pneumococcal and Hib mortality and morbidity estimates and wrote the first draft of the manuscript. BW, YQ, ZY, MDK, and HF designed the project and oversaw the analysis and manuscript writing. TX, HZ, and CG prepared provincial vaccine coverage and population data in China. YG had oversight responsibility for the project. All coauthors provided feedback during the design and interpretation of the project. They also contributed to revisions of the manuscript. HF supervised the entire project. XL, BW, and WY contributed equally as the co-first authors. ZY, MDK, and HF contributed equally as the senior authors.

### Data sharing statement

Detailed model code and results are available from online open access database. Some model inputs are also available from the online open access database. Individuals wishing to obtain complete model inputs should submit requests to Brian Wahl (bwahl@jhu.edu).

### Editor note

The Lancet Group takes a neutral position with respect to territorial claims in published maps and institutional affiliations.

## Declaration of interests

HF reports grants from the Bill & Melinda Gates Foundation and Sanofi Pasteur. BW reports grants from the Bill & Melinda Gates Foundation. MDK reports grants from the Bill & Melinda Gates Foundation, Pfizer and Gavi Alliance, and personal fees from Merck. CG reports grants from the Bill & Melinda Gates Foundation and Pfizer, and personal fees from Merck. All other authors declare no competing interests.

## Appendix Supplementary materials

Image, application 1
