# Supplementary material for: National, regional, and provincial disease burden attributed to Streptococcus pneumoniae and Haemophilus influenzae type b in children in China: Modelled estimates for 2010–17
Source: Lancet Reg Health West Pac. 2022 Mar 16;22:100430. doi: 10.1016/j.lanwpc.2022.100430 (PMC8928075; doi:10.1016/j.lanwpc.2022.100430)
Supplement: Supplementary file 1 [file mmc1.docx]

**Supplementary appendix for**

**National, regional, and provincial disease burden attributed to *Streptococcus pneumoniae* and *Haemophilus influenzae* type b in children in China: modelled estimates for 2010–17**

**Authors:** Xiaozhen Lai,^1,2*^ Brian Wahl,^3,4,5*^ Wenzhou Yu,^6*^ Tingting Xu,^7^ Haijun Zhang,^1,2^ Cristina Garcia,^4,5^ Ying Qin,^8^ Yan Guo,^2^ Zundong Yin,^6†^ Maria Deloria Knoll,^4,5†^ Hai Fang^1,9,10†^

^1^ China Center for Health Development Studies, Peking University, Beijing, China

^2^ Department of Health Policy and Management, School of Public Health, Peking University, Beijing, China

^3^ Johns Hopkins India, Lucknow, India

^4^ Department of International Health, Johns Hopkins Bloomberg School of Public Health, Baltimore, USA

^5^ International Vaccine Access Center, Johns Hopkins Bloomberg School of Public Health, Baltimore, USA

^6^ National Immunization Programme, Chinese Center for Disease Control and Prevention, Beijing, China

^7^ Department of Health Policy and Management, School of Public Health, Capital Medical University, Beijing, China

^8^ Division of Infectious Diseases, Chinese Center for Disease Control and Prevention, Beijing, China

^9^ Peking University Health Science Center-Chinese Center for Disease Control and Prevention Joint Research Center for Vaccine Economics, Peking University, Beijing, China

^10^ Key Laboratory of Reproductive Health, National Health Commission of the People’s Republic of China, Beijing, China

^*^ contributed equally

^†^ contributed equally

**Keywords:** Immunization, *Streptococcus pneumoniae*, *Haemophilus influenzae* type b, China

**Correspondence:**

Zundong Yin, National Immunization Programme, Chinese Center for Disease Control and Prevention, Beijing, China, E-mail: [yinzd@chinacdc.cn](mailto:yinzd@chinacdc.cn), Phone: +86 13520056303

Maria Deloria Knoll, International Vaccine Access Center, Johns Hopkins Bloomberg School of Public Health, Baltimore, USA, [mknoll2@jhu.edu](mailto:mknoll2@jhu.edu), Phone: +1 443-287-4832

Hai Fang, China Center for Health Development Studies, Peking University, Beijing, China, E-mail: [hfang@hsc.pku.edu.cn](mailto:hfang@hsc.pku.edu.cn), Phone: +86 1082805702

**Funding:** Bill & Melinda Gates Foundation.

**补充附录**

**中国儿童国家、地区和省级肺炎链球菌和流感嗜血杆菌疾病负担：2010–17模型估计**

**作者:** 来晓真,^1,2*^ Brian Wahl,^3,4,5*^ 余文周,^6*^ 徐婷婷,^7^ 张海军,^1,2^ Cristina Garcia,^4,5^ 秦颖,^8^ 郭岩,^2^ 尹遵栋,^6†^ Maria Deloria Knoll,^4,5†^ 方海^1,9,10†^

^1^ 北京大学中国卫生发展研究中心，北京，中国

^2^ 北京大学公共卫生学院卫生政策与管理系，北京，中国

^3^ 约翰斯·霍普金斯大学印度，勒克瑙，印度

^4^ 约翰斯·霍普金斯大学布隆伯格公共卫生学院国际卫生系，巴尔的摩，美国

^5^ 约翰斯·霍普金斯大学布隆伯格公共卫生学院国际疫苗获取中心，巴尔的摩，美国

^6^ 中国疾病预防控制中心免疫规划中心，北京，中国

^7^ 首都医科大学公共卫生学院卫生管理与政策系，北京，中国

^8^ 中国疾病预防控制中心传染病预防控制处，北京，中国

^9^ 北京大学医学部-中国疾病预防控制中心疫苗经济学联合研究中心，北京，中国

^10^ 卫生部生育健康重点实验室，北京，中国

^*^ 贡献等同

^†^ 贡献等同

**关键词**：接种；肺炎链球菌，流感嗜血杆菌，中国

**通讯作者：**

尹遵栋，中国疾病预防控制中心免疫规划中心，北京，中国，电子邮箱：[yinzd@chinacdc.cn](mailto:yinzd@chinacdc.cn)，电话：+86 13520056303

Maria Deloria Knoll，约翰斯·霍普金斯大学布隆伯格公共卫生学院国际疫苗获取中心，巴尔的摩，美国，电子邮箱：[mknoll2@jhu.edu](mailto:mknoll2@jhu.edu)，电话：+1 443-287-4832

方海，北京大学中国卫生发展研究中心，北京，中国，电子邮箱：[hfang@hsc.pku.edu.cn](mailto:hfang@hsc.pku.edu.cn)，电话：+86 1082805702

**中文摘要**

**背景**

目前，中国尚未将肺炎结合疫苗和流感嗜血杆菌疫苗纳入到国家免疫规划中。我们估计了2010–17年中国儿童在国家、地区和省级层面由于肺炎链球菌和流感嗜血杆菌导致的死亡和发病负担，希望为中国免疫规划政策提供建议。

**方法**

我们使用疫苗临床实验数据、细菌性脑膜炎发病和具体病原体病死情况监测数据来估计肺炎链球菌和流感嗜血杆菌所致的肺炎、脑膜炎死亡和发病的比例。在模型中，使用这些比例估计中国1–59月龄儿童省级全因肺炎死亡和发病数、全因脑膜炎死亡数和病死率，同时考虑疫苗的接种情况。非肺炎非脑膜炎发病数使用具体病原体非肺炎非脑膜炎发病数与脑膜炎发病数的比例进行估计。

**结果**

据估计，2010–17年，中国1–59月龄儿童肺炎链球菌和流感嗜血杆菌导致的死亡数分别下降了49%和56%，肺炎链球菌所致死亡数从2010年的15 600（不确定区间UR 10 800–17 300）下降到2017年的8 000（5 500–8900），流感嗜血杆菌所致死亡数由2010年的6 500（4 500–8 800）下降到2017年的2 900（2 000–3 900）。在2017年，估计严重肺炎链球菌和流感嗜血杆菌病例数分别下降到218 200（161 500–252 200）和49 900（29 000–99 100），下降率为16%和29%，估计2017年全国3针肺炎结合疫苗和流感嗜血杆菌疫苗接种率分别为1.3%和33.4%。中国西部疾病负担最高，但两种疫苗的接种率最低。

**解释**

中国儿童肺炎链球菌和流感嗜血杆菌导致的死亡和病例均有所下降，若是提高肺炎结合疫苗和流感嗜血杆菌疫苗的接种率，相关疾病负担将会进一步下降，尤其是在中国西部省份。

**基金项目**

比尔及梅琳达·盖茨基金会

**Webappendix List**

- Webappendix 1: GATHER checklist
- Webappendix 2: Literature review methodology
- Webappendix 3: Data sources of studies contributing to pathogen-specific meningitis case fatality ratio (CFR) estimates stratified by child mortality settings
- Webappendix 4: Data sources of studies contributing to the distribution of meningitis cases by etiology from Asia
- Webappendix 5: Summary estimates of pathogen-specific pneumonia morbidity and mortality
- Webappendix 6: Estimates of pathogen-specific meningitis morbidity and mortality.
- Webappendix 7: Methods used to estimate pathogen-specific pneumonia, meningitis and non-pneumonia, non-meningitis (NPNM) cases and deaths in China
- Webappendix 8: Pneumococcal deaths in China by region, province, and year
- Webappendix 9: Hib deaths in China by region, province, and year
- Webappendix 10: Pneumococcal cases in China by region, province, and year
- Webappendix 11: Hib cases in China by region, province, and year
- Webappendix 12: Sensitivity results of pneumococcal pneumonia mortality by different vaccine efficacy in China by region, province, and year
- Webappendix 13: Sensitivity results of pneumococcal pneumonia mortality by different sources of all-cause pneumonia in China in 2017
- Webappendix 14: PCV and Hib vaccine coverage by alternative estimation methods in China in 2017
- Webappendix 15: Sensitivity results of disease burden in 2017 by adopting the same vaccine coverage rates in 2010
- Webappendix 16: Pathogen-specific disease burden in Chinese children aged 1–59 months in 2018 and 2019 at the national level

**Webappendix 1: GATHER checklist**

| **Item #** | **Checklist item** | **Reported** |
| --- | --- | --- |
| **Objectives and funding** | | |
| **1** | Define the indicator(s), populations (including age, sex, and geographic entities), and time period(s) for which estimates were made. | Indicators, populations, and time periods provided in methods section. |
| **2** | List the funding sources for the work. | Funding source provided in summary section. |
| **Data Inputs** | | |
| ***For all data inputs from multiple sources that are synthesized as part of the study:*** | | |
| **3** | Describe how the data were identified and how the data were accessed. | Data identification and collection approaches provided in methods section and in previous publications.^1 2^ |
| **4** | Specify the inclusion and exclusion criteria. Identify all ad-hoc exclusions. | Inclusion and exclusion criteria provided in webappendix and in previous publications.^1 2^ |
| **5** | Provide information on all included data sources and their main characteristics. For each data source used, report reference information or contact name/institution, population represented, data collection method, year(s) of data collection, sex and age range, diagnostic criteria or measurement method, and sample size, as relevant. | Data sources and characteristics provided in webappendix and in online open access database. |
| **6** | Identify and describe any categories of input data that have potentially important biases (e.g., based on characteristics listed in item 5). | Potentially important biases provided in discussion section. |
| ***For data inputs that contribute to the analysis but were not synthesized as part of the study:*** | | |
| **7** | Describe and give sources for any other data inputs. | Other sources of data described in methods section. |
| ***For all data inputs:*** | | |
| **8** | Provide all data inputs in a file format from which data can be efficiently extracted (e.g., a spreadsheet rather than a PDF), including all relevant meta-data listed in item 5. For any data inputs that cannot be shared because of ethical or legal reasons, such as third-party ownership, provide a contact name or the name of the institution that retains the right to the data. | Data inputs available in Excel spreadsheet in online open access database when appropriate. Data inputs that cannot be shared will be described in the webappendix with institutional contacts for potentially obtaining these data. |
| **Data analysis** | | |
| **9** | Provide a conceptual overview of the data analysis method. A diagram may be helpful. | Conceptual model provided in Figure 1 of the present study and Figure 1 of previous publication.^3^ |
| **10** | Provide a detailed description of all steps of the analysis, including mathematical formulae. This description should cover, as relevant, data cleaning, data pre-processing, data adjustments and weighting of data sources, and mathematical or statistical model(s). | Detailed description of analysis provided in methods section, webappendix, and previous publications.^1 3–5^ |
| **11** | Describe how candidate models were evaluated and how the final model(s) were selected. | Discussion of candidate model considerations provided in methods section and discussed in detail in previous publication.^3^ |
| **12** | Provide the results of an evaluation of model performance, if done, as well as the results of any relevant sensitivity analysis. | Results of model performance at national, regional, and provincial levels provided in results section and in webappendix. |
| **13** | Describe methods for calculating uncertainty of the estimates. State which sources of uncertainty were, and were not, accounted for in the uncertainty analysis. | Uncertainty methods and sources provided in methods section. |
| **14** | State how analytic or statistical source code used to generate estimates can be accessed. | Access to source code provided in online open access database. |
| **Results and Discussion** | | |
| **15** | Provide published estimates in a file format from which data can be efficiently extracted. | Results in Excel spreadsheet provided in online open access database. |
| **16** | Report a quantitative measure of the uncertainty of the estimates (e.g. uncertainty intervals). | Uncertainty intervals provided in the manuscript, webappendix, and online open access database. |
| **17** | Interpret results in light of existing evidence. If updating a previous set of estimates, describe the reasons for changes in estimates. | Discussion of results relevant to existing research provided in research in context, results, and discussion sections. |
| **18** | Discuss limitations of the estimates. Include a discussion of any modelling assumptions or data limitations that affect interpretation of the estimates. | Limitations of data and models provided in discussion section. |

**Webappendix 2: Literature review methodology**

**2.1 Data sources**

For the pneumonia models, we identified PCV and Hib vaccine trials and effectiveness studies from a systematic review of the literature and a Cochrane review of PCV efficacy.^6^ We also updated a systematic review of pneumococcal and Hib invasive disease from 1980-2005^2^ with published and unpublished literature through 2014, including data on pathogen-specific meningitis case fatality ratio (CFR), the etiologic distribution of bacterial meningitis cases, and pathogen-specific meningitis. We searched six global databases (i.e. PubMed, Embase, Biosis, Cochrane, Global Health, Pascal), five regional databases (i.e. IMEMR, IMSEAR, LILACS, WHOLIS, WPRIM), and three Chinese-language databases (i.e. CNKI, Wanfang, CQVIP) followed the same quality assessment criteria described in the previously published literature review.^2^ We also used unpublished data from the Hib Rapid Assessment Tool and WHO Invasive Bacterial Disease surveillance network.

**2.2 Literature review methods and criteria**

The literature review was conducted in several phases. First, researchers collected articles using concept groups from the above mentioned data sources. Next, two reviewers screened each article based on titles and abstracts, and the full text of studies meeting the inclusion criteria based on title and abstract were screened again by two reviewers. Finally, two reviewers reviewed studies one additional time, extracted relevant data, assessed quality for those that met the inclusion criteria, and applied exclusion criteria. Two lead reviewers adjudicated conflicting reviews.

Detailed inclusion and exclusion criteria can be found in the previous publications.^2^ In brief, inclusion criteria included: (1) randomized controlled trial to test the effectiveness of Hib or pneumococcal vaccine. (2) includes data on Hib or pneumococcal disease reduction following widespread use of the vaccine. (3) reports data about the general study population (under-five children). Exclusion criteria included: (1) with “substantial use” (defined as coverage > 25%) of Hib or Spn conjugate vaccines in the study population either before or during the study period, or lacks such data. (2) with cases occurring prior to 1980. (3) involves less than 12 months of case ascertainment; (4) focuses on a specific sub-population of children (e.g. distinct racial/ethnic background, shared medical condition believed to change the risk of disease such as HIV infection) only. (5) with children not grouped into any of the three following age categories: 1 (±1 month) to 11 month olds (±1 month); 1 (±1 month) to 23 month olds (±1 month); or 1 (±1 month) to 59 month olds (±1 year).

Quality assessments for studies reporting incidence, case fatality ratio, and the distribution of bacterial meningitis cases by etiology were based on two subjective criteria: (1) the likelihood of the study missing cases and (2) the reliability of the diagnostic methods for laboratory isolation. These criteria were assessed independently by two data reviewers during the data abstraction phase. Based on these criteria, studies were classified into one of three categories: "A" papers where both reviewers judged both criteria to have been met; "B" papers where only one reviewer judged each criterion to have been met; "C" papers where both reviewers judged that neither criterion had been met or data was not available to allow a judgment. Category “C” studies, as well as all included studies from Asia and Africa, underwent a third quality assessment before a decision was made about inclusion in the final data set for meningitis.

**2.3 PubMed Search Strategy**

**Concept 1: Streptococcus pneumoniae**

"Streptococcus pneumoniae"[Mesh] OR "streptococcus pneumoniae"[all fields] OR "streptococcal pneumonia"[all fields] OR "diplococcus pneumoniae"[all fields] OR "pneumococcus"[all fields] OR "pneumococcic pneumonia"[all fields] OR "s pneumoniae"[all fields] OR "pneumococci"[all fields] OR "pneumococcal"[all fields] OR "Pneumococcal Vaccines"[Mesh] OR "prevnar"[all fields] OR "pnu-imune 23"[all fields] OR " 7-valent pncompc vaccine"[all fields] OR "pncrm7"[all fields] OR "mnc-crm197"[all fields] OR "mncc"[all fields] OR "pneumovax"[all fields] OR "pnu-imune vaccine"[all fields] OR “pcv 7"[all fields] OR “pcv 10"[all fields] OR “pcv 13"[all fields] OR "prevenar"[all fields] OR "prevnar"[all fields] OR "streptorix"[all fields] OR "synflorix"[all fields]

**Concept 2: Haemophilus influenzae type b (Hib)**

“Haemophilus influenzae”[Mesh] OR “haemophilus influenzae type b”[Mesh] OR haemophilus influenzae”[Mesh] OR “diphtheria pertussis poliomyelitis tetanus haemophilus influenzae type b hepatitis b vaccine”[all fields] OR “diphtheria pertussis tetanus haemophilus influenzae type b vaccine”[all fields] OR “diphtheria pertussis tetanus haemophilus influenzae type b vaccine”[all fields] OR “haemophilus influenzae type b dtp vaccine”[all fields] OR “haemophilus influenzae type b hepatitis b vaccine”[all fields] OR “haemophilus influenzae type b vaccine”[all fields] OR “hepatitis b haemophilus influenzae type b vaccine”[all fields] OR “hib ompc”[all fields] OR “hib vax”[all fields] OR “h influenzae”[all fields] OR “hib disease”[all fields] OR “hib infection”[all fields] OR “hib immuni”[all fields] OR “hemophilius influ”[all fields] OR “hemophilus influenzae”[all fields]

**Concept 3: Disease**

“invasive pneumococcal disease”[all fields] OR “pneumococcal invasive disease”[all fields] OR “Streptococcal Infections”[Mesh] OR “Streptococcal Infections”[all fields] OR “Streptococcal Infection”[all fields] OR “streptococcus infection” OR “Meningitis, Pneumococcal”[Mesh] OR “pneumococcal bacteraemia” OR “Meningitis, Bacterial”[Mesh] OR “Bacterial Infections”[Mesh] OR “bacterial infection” OR “bacterial infections” OR “Meningitis”[Mesh] OR “meningitis” OR “meningitides” OR “meningeal” OR “perimeningeal” OR “Respiratory Tract Diseases”[Mesh] OR “respiratory tract disease”[all fields] OR “respiratory tract diseases”[all fields] OR “airway disease”[all fields] OR “airway diseases”[all fields] OR “airway disorder”[all fields] OR “airway disorders”[all fields] OR “respiratory disease”[all fields] OR “respiratory disorder”[all fields] OR “respiratory disorders”[all fields] OR “respiratory illness”[all fields] OR “respiratory tract diseases”[all fields] OR “respiratory tract disease”[all fields] OR “respiratory tract disorder”[all fields] OR “respiratory tract disorders”[all fields] OR “lower respiratory tract infection”[all fields] OR “lower respiratory tract infections”[all fields] OR “lower respiration tract infections”[all fields] OR “lung infection”[all fields] OR “lung infections”[all fields] OR “pulmonary infection”[all fields] OR “pulmonary infections”[all fields] OR “Pneumonia, Bacterial”[Mesh] OR “pneumonia”[mesh] OR “pneumonia”[all fields] OR “pneumonic”[all fields] OR “Pneumonias”[all fields] OR “Pneumonitis”[all fields] OR “Pneumonitides”[all fields] OR “pleuropneumonia”[all fields] OR “pleuropneumonitis”[all fields] OR “Pulmonary Inflammation”[all fields] OR “alri”[all fields] OR “peripneumonia”[all fields] OR “pulmonary inflammation”[all fields] OR “Bacteremia”[Mesh] OR “bacteremia”[all fields] OR “bacteremias”[all fields] OR “septicemia”[all fields] OR “septicaemia”[all fields] OR “Sepsis”[Mesh] OR “sepsis”[all fields] OR “Pyemia”[all fields] OR “Pyemias”[all fields] OR “Pyohemia”[all fields] OR “Pyaemia”[all fields] OR “infectious pneumopath”[all fields]

**Concept 4: Age group**

"child"[mesh] OR "child"[all fields] OR "children"[all fields] OR youth* OR "young people"[all fields] OR "baby"[all fields] OR "babies"[all fields] OR "infant"[mesh] OR infant* OR "childhood"[all fields] OR toddler* OR "kid"[all fields] OR "kids"[all fields] OR "young patient"[all fields] OR "young patients"[all fields] OR "boy"[all fields] OR "boys"[all fields] OR girl* OR "young age"[all fields] OR "child mortality"[mesh] OR "pediatric"[all fields] OR "Child, Preschool"[Mesh] OR “pre-schooler"[all fields] OR “preschooler"[all fields] OR “under 5"[all fields] OR “under five"[all fields] OR “under fives"[all fields] OR "less than five"[all fields]

**Concept 5: Exclusions**

NOT "Case Reports" [Publication Type] OR "case report"[all fields] OR "case reports"[all fields] NOT ("animals"[mh] NOT ("animals"[mh] AND "humans"[mh]))

**Webappendix 3: Data sources of studies contributing to pathogen-specific meningitis case fatality ratio (CFR) estimates stratified by child mortality settings**

**Webappendix 3.1 Streptococcus pneumoniae**

**Low child mortality settings (<30 deaths per 1 000 live births): 39 studies**

- Andrade AL, Oliveira R, Vieira MA, et al. Population-based surveillance for invasive pneumococcal disease and pneumonia in infants and young children in Goiania, Brazil. *Vaccine* 2012; **30**: 1901–9.
- Awaidy STA, Obeidani IA, Busaidy SSA, Mahrouqi SA. Epidemiology of invasive pneumococcal infection among hospitalized children aged less than 5 years in Oman. *Vaccine* 2012; **30**: G7–10.
- Ayed MSA, Hawan AA. Retrospective review of invasive pediatric pneumococcal diseases in a military hospital in the southern region of Saudi Arabia. *Ann Saudi Med* 2011; **31**: 469–72.
- Batuwanthudawe R, Karunarathne K, Dassanayake M, et al. Surveillance of invasive pneumococcal disease in Colombo, Sri Lanka. *Clin Infect Dis* 2009; **48**: S136–40.
- Benavides JA, Ovalle OO, Salvador GR, et al. Population-based surveillance for invasive pneumococcal disease and pneumonia in infants and young children in Bogota, Colombia. *Vaccine* 2012; **30**: 5886–92.
- Biaukula V, Mulholland EK, Tikoduadua L, et al. Meningitis in children in Fiji: etiology, epidemiology, and neurological sequelae. *Int J Infect Dis* 2012; **16**: e289–95.
- Boehme C, Soto L, Rodríguez G, Serra J, Illesca V, Reydet P. Three years of acute bacterial meningitis in the pediatric service at the Temuco Regional Hospital. *Rev Med Chil* 1993; **121**: 633–8.
- Camou T, Palacio R, Fabio JLD, Hortal M. Invasive pneumococcal diseases in Uruguayan children: comparison between serotype distribution and conjugate vaccine formulations. *Vaccine* 2003; **21**: 2093–6.
- Chan IM, Ng DK, Miu T, et al. Invasive pneumococcal disease in Hong Kong children. *J Pediatr Respirology Critic Care* 2013; **9**: 4–8.
- Chavez PA, Rojas AC, Rakela RS, Chadid SJ, Fischer SC. Meningitis bacteriana aguda: experiencia de 10 anos. *Rev Chil Infectol* 1994; **11**: 92–8.
- Meneses F, Pérez Rodríguez A. Las meningoencefalitis bacterianas en la poblacion infantil cubana: 1998-2000. *Rev Cubana Pediatr* 2002; **74**: 106–14.
- Michael D, Parkinson AJ, Bulkow LR, Anne FM, Helen P, Parks DJ. The Epidemiology of Invasive Pneumococcal Disease in Alaska, 1986-1990 Ethnic Differences and Opportunities for Prevention. *J Infect Dis* 1994; **170**: 368–76.
- Musawi A, Muna. A retrospective epidemiological study of invasive pneumococcal infections in children aged 0-5 years in Bahrain from 1 January 1999 to 31 December 2003. *Vaccine* 2012; **30**: G2–6.
- Fagan RL, Hanna JN, Messer RD, Brookes DL, Murphy DM. The epidemiology of invasive pneumococcal disease in children in Far North Queensland. *J Paediatr Child Health* 2001; **37**: 571–5.
- Fortnum HM, Davis AC. Epidemiology of bacterial meningitis. Arch Dis Child 1993; 68: 763–7.
- Grzesiowski P, Skoczynska A, Albrecht P, et al. Invasive pneumococcal disease in children up to 5 years of age in Poland. *Eur J Clin Microbiol Infect Dis* 2008; **27**: 883–5.
- Hanna JN, Wild BE. Bacterial meningitis in children under five years of age in Western Australia. *Med J Aust* 1991; **155**: 160–4.
- Harboe ZB, Thomsen RW, Riis A, et al. Pneumococcal serotypes and mortality following invasive pneumococcal disease: a population-based cohort study. *PLoS Med* 2009; **6**: e1000081.
- Ishikawa T, Asano Y, Morishima T, et al. Epidemiology of bacterial meningitis in children: Aichi prefecture, Japan, 1984-1993. *Pediatr Neurol* 1996; **14**: 244–50.
- Jansen AGSC, Rodenburg GD, Greeff SCD, et al. Invasive pneumococcal disease in the Netherlands: Syndromes, outcome and potential vaccine benefits. *Vaccine* 2009; **27**: 2394–401.
- Johnson AP, Waight P, Andrews N, Pebody R, George RC, Miller E. Morbidity and mortality of pneumococcal meningitis and serotypes of causative strains prior to introduction of the 7-valent conjugant pneumococcal vaccine in England. *J Infect* 2007; **55**: 394–9.
- Kojouharova M, Gatcheva N, Setchanova L, Robertson SE, Wenger JD. Epidemiology of meningitis due to Haemophilus influenzae type B in children in Bulgaria: A prospective, population-based surveillance study. *Bull World Health Organ* 2002; **80**: 690–5.
- Lagos ZR, San Martín BO, Erazo LA, Avendaño BA, Levine M. Epidemiologia de las enfermedades invasoras causadas por Streptococcus pneumoniae en ninos chilenos: proyecciones clinicas y de salud publica. *Rev Chilena Infectol* 2001; **18**: 15–21.
- Lim LH, Lee WS, Parasakthi N. Childhood invasive pneumococcal disease: a hospital-based study from Malaysia. *J Paediatr Child Health* 2007; **43**: 366–9.
- Linares-Prez N. Private communication (unpublished data from Cuba).
- Lucey JM, Gavin P, Cafferkey M, Butler KM. Pneumococcal meningitis: clinical outcomes in a pre-vaccine era at a Dublin paediatric hospital, 1999–2007. *Ir J Med Sci* 2011; **180**: 47–50.
- McIntyre PB, Macintyre CR, Gilmour R, Wang H. A population based study of the impact of corticosteroid therapy and delayed diagnosis on the outcome of childhood pneumococcal meningitis. *Arch Dis Child* 2005; **90**: 391–6.
- Memish ZA, El-Saed A, Al-Otaibi B, Shaalan MA, Alola SA, Thaqafi AO. Epidemiology of invasive pneumococcal infection in children aged five years and under in Saudi Arabia: a five-year retrospective surveillance study. *Int J Infect Dis* 2010; **14**: e708–12.
- Miller E, Waight P, Efstratiou A, Brisson M, Johnson A, George R. Epidemiology of invasive and other pneumococcal disease in children in England and Wales 1996–1998. *Acta Paediatr* 2000; **89**: 11–6.
- Namani S, Koci R, Dedushi K, Raka L. Causative pathogens of bacterial meningitis in children and their susceptibility to antibiotics. *The Internet JInfects Dis* 2010; **9**: 1–6.
- Peng Q, Liao H, Tang J. Clinical analysis of purulent meningitis caused by streptococcus pneumoniae in 12 children. *Chinese Pediatric Emergency Medicine* 2013; **20**: 169–71. In Chinese
- Rendi-Wagner P, Georgopoulos A, Kundi M, et al. Prospective surveillance of incidence, serotypes and antimicrobial susceptibility of invasive Streptococcus pneumoniae among hospitalized children in Austria. *J Antimicrob Chemother* 2004; **53**: 826–31.
- R'Ios AM, Hoz F, Leal AL, Castillo O, Castaeda E. Impacto de la resistencia a antimicrobianos y de serotipos de Streptococcus pneumoniae en la mortalidad de ninos menores de 5 anos con enfermedad invasora. *Rev Panam Salud Publica* 1999; **5**: 69–76.
- Robinson KA, Baughman W, Rothrock G, et al. Epidemiology of invasive Streptococcus pneumoniae infections in the United States, 1995-1998: Opportunities for prevention in the conjugate vaccine era. *JAMA* 2001; **285**: 1729–35.
- Rosanna L, Muñoz A, San MO, et al. Age- and serotype-specific pediatric invasive pneumococcal disease: insights from systematic surveillance in Santiago, Chile, 1994-2007. *J Infect Dis* 2008; **198**: 1809–17.
- Shi X, Zhang B, Li Y, et al. Analysis of clinical manifestation and serotype of 39 children with meningitis caused by Streptococcus pneumoniae in Suzhou. *Journal of Nantong University (Medical Sciences)* 2018; **38**: 408–12. In Chinese
- Trotman H, Olugbuyi O, Barton M, Mcgregor D, Thomas S. Pneumococcal meningitis in Jamaican children. *West Indian Med J* 2009; **58**: 585–8.
- Von Kries R, Hermann M, Hachmeister A, et al. Prediction of the potential benefit of different pneumococcal conjugate vaccines on invasive pneumococcal disease in German children. *Pediatr Infect Dis J* 2002; **21**: 1017–23.
- Voss L, Lennon D, Okesene-Gafa K, Ameratunga S, Martin D. Invasive pneumococcal disease in a pediatric population, Auckland, New Zealand. *Pediatr Infect Dis J* 1994; **13**: 873–8.

**Medium child mortality settings (30-<75 deaths per 1 000 live births)：14 studies.**

- Abucejo-Ladesma E, Simoes EAF, Lupisan SP, et al. Serious community-acquired paediatric infections in rural Asia (Bohol Island, Philippines): bacterial meningitis in children less than 5 years of age. *Scand J Infect Dis* 2007; **39**: 983–9.
- Asturias EJ, Soto M, Menendez R, et al. Meningitis and pneumonia in Guatemalan children: the importance of Haemophilus influenzae type b and Streptococcus pneumoniae. *Rev Panam Salud Publica* 2003; **14**: 377–84.
- Berkley JA, Lowe BS, Mwangi I, et al. Bacteremia among children admitted to a rural hospital in Kenya. *N Engl J Med* 2005; **352**: 39–47.
- Capeding MR, Bravo L, Santos J, Kilgore PE, Moscariello M. Prospective surveillance study of invasive pneumococcal disease among urban children in the Philippines. *Pediatr Infect Dis J* 2013; **32**: e383–9.
- Chhetri UD, Shrestha S, Pradhan R, et al. Clinical profile of invasive pneumococcal disease in Patan Hospital, Nepal. *Kathmandu Univ Med J* 2012; **9**: 45–9.
- El Mdaghri N, Jilali N, Belabbes H, Jouhadi Z, Lahssoune M, Zaid S. Epidemiological profile of invasive bacterial diseases in children in Casablanca, Morocco: antimicrobial susceptibilities and serotype distribution. *East Mediterr Health J* 2012; **18**: 1097–101.
- Flanner. Private communication (unpublished data from Brazil).
- Gomez E, Peguero M, Sanchez J, et al. Population-based surveillance for bacterial meningitis in the Dominican Republic: Implications for control by vaccination. *Epidemiol Infect* 2000; **125**: 549–54.
- Lin M, Dong B, Tang Z, et al. Epidemiological features of bacterial meningitis among children under 5 years old in Nanning. *South China Journal of Preventive Medicine* 2004; **30**: 30–3. In Chinese
- Lovera D, Arbo A. Risk factors for mortality in Paraguayan children with pneumococcal bacterial meningitis. *Trop Med Int Health* 2005; **10**: 1235–41.
- Reis JN, Cordeiro SM, Coppola SJ, et al. Population-based survey of antimicrobial susceptibility and serotype distribution of Streptococcus pneumoniae from meningitis patients in Salvador, Brazil. *J Clin Microbiol* 2002; **40**: 275–7.
- Shah AS, Deloria Knoll M, Sharma PR, et al. Invasive pneumococcal disease in Kanti Children's Hospital, Nepal, as observed by the South Asian Pneumococcal Alliance network. *Clin Infect Dis 2009*; **48**: S123–8.
- Unpublished data from Bangladesh.
- Williams EJ, Thorson S, Maskey M, et al. Hospital-based surveillance of invasive pneumococcal disease among young children in urban Nepal. *Clin Infect Dis* 2009; **48**: S114–22.

**Webappendix 3.2 Haemophilus influenzae type b (Hib)**

**Low child mortality settings (<30 deaths per 1 000 live births): 31 studies**

- Almuneef M, Memish Z, Khan Y, Kagallwala A, Alshaalan M. Childhood bacterial meningitis in saudi arabia. *J Infect* 1998; **36**: 157–60.
- Anh DD, Kilgore PE, Kennedy WA, Nyambat B, Long HT. Haemophilus influenzae type B meningitis among children in Hanoi, Vietnam: epidemiologic patterns and estimates of H. Influenzae type B disease burden. *Am J Trop Med Hyg* 2006; **74**: 509–15.
- Anh DD, Kilgore PE, Slack MPE, et al. Surveillance of pneumococcal-associated disease among hospitalized children in Khanh Hoa Province, Vietnam. *Clin Infect Dis* 2009; **48**: S57–64.
- Barton-Forbes MA, Samms-Vaughan M, Irons B. Epidemiology of Haemophilus influenzae invasive disease in Jamaica, 1990-1993. *West Indian Med J* 2000; **49**: 200–4.
- Batuwanthudawe R, Karunarathne K, Dassanayake M, et al. Surveillance of invasive pneumococcal disease in Colombo, Sri Lanka. *Clin Infect Dis* 2009; **48**: S136–40.
- Batuwanthudawe R, Rajapakse L, Somaratne P, Dassanayake M, Abeysinghe N. Incidence of childhood Haemophilus influenzae type b meningitis in Sri Lanka. *Int J Infect Dis* 2010; **14**: e372–6.
- Boehme C, Soto L, Rodríguez G, Serra J, Illesca V, Reydet P. Three years of acute bacterial meningitis in the pediatric service at the Temuco Regional Hospital. *Rev Med Chil* 1993; **121**: 633–8.
- Booy R, Hodgson SA, Slack MPE, Anderson EC, Moxon ER. Invasive Haemophilus influenzae type b disease in the Oxford region (1985-91). *Arch Dis Child* 1993; **69**: 225–8.
- Bower C, Payne J, Condon R, Hendrie D, Harris A, Henderson R. Sequelae of Haemophilus influenzae type b meningitis in Aboriginal and non-Aboriginal children under 5 years of age. *J Paediatr Child Health* 1994; **30**: 393–7.
- Butsashvili M, Kandelaki G, Eloshvili M, Chlikadze R, Imnadze P, Avaliani N. Surveillance of bacterial meningitis in the country of Georgia, 2006-2010. *J Community Health* 2013; **38**: 724–6.
- Chavez PA, Rojas AC, Rakela RS, Chadid SJ, Fischer SC. Meningitis bacteriana aguda: experiencia de 10 anos. *Rev Chil Infectol* 1994; **11**: 92–8.
- Chen MK, Wang CC, Chu ML, Pan TM. Prospective surveillance of children with invasive Haemophilus influenzae disease in Taiwan. *J Microbiol Immunol Infect* 1999; **32**: 257–60.
- Dagan R, Isaachson M, Lang R, Karpuch J, Block C, Amir J. Epidemiology of pediatric meningitis caused by Haemophilus influenzae type b, Streptococcus pneumoniae, and Neisseria meningitidis in Israel: a 3-year nationwide prospective study. *J Infect Dis* 1994; **169**: 912–6.
- De Jonghe M, Glaesener G. Type B Haemophilus influenzae infections. Experience at the pediatric hospital of Luxembourg. *Bull Soc Sci Med Grand Duche Luxemb* 1995; **132**: 17–20.
- Félix Orlando DM, Antonio Esteban PR. Las meningoencefalitis bacterianas en la poblacion infantil cubana: 1998-2000. *Rev Cubana Pediatr* 2002; **74**: 106–14.
- Fortnum HM, Davis AC. Epidemiology of bacterial meningitis. *Arch Dis Child* 1993; 68: 763–7.
- Gilbert GL, Johnson PD, Clements DA. Clinical manifestations and outcome of Haemophilus influenzae type b disease. *J Paediatr Child Health* 1995; **31**: 99–104.
- Halfon-Yaniv I, Dagan R. Epidemiology of invasive Haemophilus influenzae type b infections in Bedouins and Jews in Southern Israel. *Pediatr Infect Dis J* 1990; **9**: 321–5.
- Ishikawa T, Asano Y, Morishima T, et al. Epidemiology of bacterial meningitis in children: Aichi Prefecture, Japan, 1984–1993. *Pediatr Neurol* 1996; **14**: 244–50.
- Kamiya H, Uehara S, Kato T, et al. Childhood bacterial meningitis in Japan. *Pediatr Infect Dis J* 1998; **17**: S183–5.
- Kristensen K, Kaaber K, Rnne T, Larsen SO, Henrichsen J. Epidemiology of Haemophilus influenzae type b infections among children in Denmark in 1985 and 1986. *Acta Paediatr* 1990; **79**: 587–92.
- Lin MC, Chiu NC, Chi H, Ho CS, Huang FY. Evolving trends of neonatal and childhood bacterial meningitis in northern Taiwan. *J Microbiol Immunol Infect* 2015; **48**: 296–301.
- Mcintyre P, Jepson R, Leeder S, Irwig L. The outcome of childhood Haemophilus influenzae meningitis. A population based study. *Med J Aust* 1992; **159**: 766–72.
- Mcintyre P, Leeder S, Irwig L. Invasive Haemophilus influenzae type b disease in Sydney children 1985-1987: a population-based study. *Med J Aust* 1991; **154**: 832–7.
- Muangchana C, Chunsuttiwat S, Rerks-Ngarm S, Kunasol P. Bacterial meningitis incidence in Thai children estimated by a rapid assessment tool. *Southeast Asian J Trop Med Public Health* 2009; **40**: 553–62.
- Namani S, Koci R, Dedushi K, Raka L. Causative pathogens of bacterial meningitis in children and their susceptibility to antibiotics. *J Infect Dis* 2010; **9**: 1–6.
- Rauter L, Mutz I. Haemophilus influenzae meningitis 1983 to 1992--epidemiology and sequelae of the disease. *Wien Klin Wochenschr* 1993; **106**: 187–92.
- Reinert P, Liwartowski A, Dabernat H, Guyot C, Boucher J, Carrere C. Epidemiology of Haemophilus influenzae type b disease in France. *Vaccine* 1993; **11**: S38–42.
- Schoendorf KC, Adams WG, Kiely JL, Wenger JD. National trends in Haemophilus influenzae meningitis mortality and hospitalization among children, 1980 through 1991. *Pediatrics* 1994; **93**: 663–8.
- Unpublished data from Samoa via Rapid Assessment Tool.
- Zaki M, Daoud AS, ElSaleh Q, West PW. Childhood bacterial meningitis in Kuwait. *J Trop Med Hyg* 1990; **93**: 7–11.

**Medium child mortality settings (30-<75 deaths per 1 000 live births): 18 studies.**

- Abucejo-Ladesma E, Simoes EA, Lupisan SP, et al. Serious community-acquired pediatric infections in rural Asia (Bohol Island, Philippines): bacterial meningitis in children less than 5 years of age. *Scand J Infect Dis* 2007; **39**: 983–9.
- Aida T, Adriana B, Luis S, Ana T. Infecciones invasivas por Haemophilus influenzae tipo b (Hib) en Tucuman-Argentina. *Arch Argent Pediatr* 1995; **93**: 238–44.
- Ansari I, Pokhrel Y. Culture proven bacterial meningitis in children: agents, clinical profile and outcome. *Kathmandu Univ Med J* 2011; **9**: 36–40.
- Asturias EJ, Soto M, Menendez R, et al. Meningitis and pneumonia in Guatemalan children: the importance of Haemophilus influenzae type b and Streptococcus pneumoniae. *Rev Panam Salud Publica* 2003; **14**: 377–84.
- Basualdo W, Arbo A. Invasive Haemophilus influenzae type b infections in children in Paraguay. *Arch Med Res* 2004; **35**: 126–33.
- El Mdaghri N, Jilali N, Belabbes H, Jouhadi Z, Lahssoune M, Zaid S. Epidemiological profile of invasive bacterial diseases in children in Casablanca, Morocco: antimicrobial susceptibilities and serotype distribution. *East Mediterr Health J* 2012; **18**: 1097–101.
- Flanner. Private communication (unpublished data from Brazil).
- Hussey G, Hitchcock J, Schaaf H, et al. Epidemiology of invasive Haemophilus influenzae infections in Cape Town, South Africa. *Ann Trop Paediatr* 1994; **14**: 97–103.
- Limcangco MR. Salole EG, Armour CL, et al. Epidemiology of Haemophilus influenzae type b meningitis in Manila, Philippines, 1994 to 1996. *Pediatr Infect Dis J* 2000; **19**: 7–11.
- Luby SP, Halder AK, Saha SK, et al. A low-cost approach to measure the burden of vaccine preventable diseases in urban areas. *Vaccine* 2010; **28**: 4903–12.
- Mendsaikhan J, Watt JP, Mansoor O, et al. Childhood bacterial meningitis in Ulaanbaatar, Mongolia, 2002–2004. *Clin Infect Dis* 2009; **48**: S141–6.
- Minz S, Balraj V, Lalitha MK, Murali N, Steinhoff MC. Incidence of Haemophilus influenzae type b meningitis in India. *Indian J Med Res* 2008; **128**: 57–64.
- Ramachandran P, Fitzwater SP, Aneja S, et al. Prospective multi-centre sentinel surveillance for Haemophilus influenzae type b & other bacterial meningitis in Indian children. *Indian J Med Res* 2013; **137**: 712–20.
- Ribeiro GS, Reis JN, Cordeiro SM, et al. Prevention of Haemophilus influenzae type b (Hib) meningitis and emergence of serotype replacement with type a strains after introduction of Hib immunization in Brazil. *J Infect Dis* 2003; **187**: 109–16.
- Saha SK, Rikitomi N, Ruhulamin M, et al. The increasing burden of disease in Bangladeshi children due to Haemophilus influenzae type b meningitis. *Ann Trop Paediatr* 1997; **17**: 5–8.
- Shah AS, Rikitomi N, Ruhulamin M, et al. Invasive pneumococcal disease in Kanti Children's Hospital, Nepal, as observed by the South Asian Pneumococcal Alliance network. *Clin Infect Dis* 2009; **48**: S123–8.
- Simoes LLP, Andrade ALSS, Laval CA, et al. Impact of Haemophilus influenzae b (Hib) vaccination on meningitis in Central Brazil. *Rev Saude Publica* 2004; **38**: 664–70.
- Weiss DP, Coplan P, Guess H. Epidemiology of bacterial meningitis among children in Brazil, 1997-1998. *Rev Saude Publica* 2001; **35**: 249–55.

**Webappendix 4: Data sources of studies contributing to the distribution of meningitis cases by etiology from Asia: 38 studies**

- Abanamy A, Shuja M, Khaleel M, et al. Childhood bacterial meningitis in Riyadh. *Ann Saudi Med* 1991; **11**: 628–32.
- Abomelha A, Uduman S, Saleh MF, Al-Rajeh S, Sibai MS, Al-Agib A. Childhood bacterial meningitis. *Ann Saudi Med* 1988; **8**: 274–8.
- al-Jurayyan NA, al Mazyad AS, al-Nasser MN, et al. Childhood bacterial meningitis in Al-Baha province, Saudi Arabia. *J Trop Med and Hyg* 1992; **95**: 180–5.
- Al Khorasani A, Banajehet S. Bacterial profile and clinical outcome of childhood meningitis in rural Yemen: a 2-year hospital-based study. *J Infect* 2006; **53**: 228–34.
- Al-Mazrou YY, Al-Jeffri MH, Al-Haggar SH, Musa EK, Mohamed OM, Abdalla MN. Haemophilus type B meningitis in Saudi children under 5 years old. *J Trop Paediatr* 2004; **50**: 131–6.
- Almuneef M, Memish Z, Khan Y, Kagallwala A, Alshaalan M. Childhood bacterial meningitis in Saudi Arabia. *J Infect* 1998; **36**: 157–60.
- Ansari I, Pokhrel Y. Culture proven bacterial meningitis in children: agents, clinical profile and outcome. *Kathmandu Univ Med J* 2012; **9**: 36–40.
- Azubuike JC. Childhood bacterial meningitis in Tabuk, Saudi Arabia. *Ann Saudi Med* 1990; **10**: 145–8.
- Batuwanthudawe R, Karunarathne K, Dassanayake M, et al. Surveillance of invasive pneumococcal disease in Colombo, Sri Lanka. *Clin Infect Dis* 2009; **48**: S136–40.
- Batuwanthudawe R, Rajapakse L, Somaratne P, Dassanayake M, Abeysinghe N. Incidence of childhood Haemophilus influenzae type b meningitis in Sri Lanka. *Int J Infect Dis* 2010; **14**: e372–6.
- Ceyhan M, Yildirim I, Balmeret P, et al. A prospective study of etiology of childhood acute bacterial meningitis, Turkey. *Emerg Infect Dis* 2008; **14**: 1089–96.
- Chinchankar N, Mane M, Bhave S, et al. Diagnosis and outcome of acute bacterial meningitis in early childhood. *Indian Pediatr* 2002; **39**: 914–21.
- Chinese CDC. Population-based surveillance database for bacterial meningitis in China, 2006-2017. Unpublished data.
- Choo KE, Ariffin WA, Ahmad T, Lim WL, Gururaj AK. Pyogenic meningitis in hospitalized children in Kelantan, Malaysia. *Ann Trop Paediatr* 1990; **10**: 89–98.
- Dagan R, Fraser D, Roitman M, et al. Effectiveness of a nationwide infant immunization program against Haemophilus influenzae b. The Israeli Pediatric Bacteremia and Meningitis Group. *Vaccine* 1999; **17**: 134–41.
- Du L, Han H, Shi K, et al. Pathogen distributions and drug resistance of children's bacterial meningitis in Taiyuan. *Journal of Clinical Medical Literatures* 2014; **11**: 2068–70. In Chinese
- Fujii R, Hiraiwa M, Nonaka C, Kobayashi Y. Trends in childhood bacterial meningitis in Japan (1979-1984).(Part 1). On the causative organisms. *Kansenshogaku Zasshi* 1986; **60**: 592–601.
- Gao J, Yi Z, Zhong J. The etiology and antibiotic resistance patterns of childhood purulent meningitis: a report of 164 cases. *Journal of Pediatric Pharmacy* 2013; **19**: 35–8. In Chinese
- Gratten M, Montgomery J. The Bacteriology of Acute Pneumonia and Meningitis in Children in Papua New Guinea: Assumptions, Facts and Technical Strategies. *P N G Med J* 2005; **48**: 73–86.
- Gurley ES, Hossain MJ, Montgomery SP, et al. Etiologies of bacterial meningitis in Bangladesh: results from a hospital-based study. *Am J Trop Med Hyg* 2009; **81**: 475–83.
- Kim KH, Sohn YM, Kang JH, et al. The Causative Organisms of Bacterial Meningitis in Korean Children, 1986-1995. *J Korean Med Sci* 1998; **13**: 60–4.
- Kim SA, Kim DW, Dong BQ, Kim JS, Anh DD, Kilgore PE. An expanded age range for meningococcal meningitis: molecular diagnostic evidence from population-based surveillance in Asia. *BMC Infect Dis* 2012; **12**: 310.
- Lehmann D, Yeka W, Rongap T, et al. Aetiology and clinical signs of bacterial meningitis in children admitted to Goroka Base Hospital, Papua New Guinea, 1989-1992. *Ann Trop Paediatr* 1999; **19**: 21–32.
- Lin M, Dong B, Tang Z, et al. Epidemiological features of bacterial meningitis among children under 5 years old in Nanning. *South China Journal of Preventive Medicine* 2004; **30**: 30–3. In Chinese
- Liu CC, Chen JS, Lin CH, Chen YJ, Huang CC. Bacterial meningitis in infants and children in southern Taiwan: emphasis on Haemophilus influenzae type B infection. *J Formos Med Assoc* 1993; **92**: 884–8.
- Mahmood YA, Alsaadi AA. Neonatal and childhood bacterial meningitis at Saddam general hospital in Tikrit. *Med J Tikrit Univ* 1997; **3**: 39–45.
- Mahmoud R, Mahmoud M, Badrinath P, Sheek-Hussein M, Alwash R, Nicol AG. Pattern of meningitis in Al-Ain medical district, United Arab Emirates—a decadal experience (1990–99). *J Infect* 2002; **44**: 22–5.
- Mani R, Pradhan S, Nagarathna S, Wasiulla R, Chandramuki A. Bacteriological profile of community acquired acute bacterial meningitis: a ten-year retrospective study in a tertiary neurocare centre in South India. *Indian J Med Microbiol* 2007; **25**: 108.
- Mao F, Wang J, Li J, Yu X. Aetiological spectrum and antibiotic susceptibility pattern of bacterial meningitis in infants and children in Hangzhou, China. *Acta Paediatr* 2005; **94**: 1162–3.
- Mendsaikhan J, Watt JP, Mansoor O, et al. Childhood bacterial meningitis in Ulaanbaatar, Mongolia, 2002–2004. *Clin Infect Dis* 2009; **48**: S141–6.
- Moïsi JC, Saha SK, Falade AG, et al. Enhanced diagnosis of pneumococcal meningitis with use of the Binax NOW immunochromatographic test of Streptococcus pneumoniae antigen: a multisite study. *Clin Infect Dis* 2009; **48**: S49–56.
- Saha SK, Rikitomi N, Ruhulamin M, et al. The increasing burden of disease in Bangladeshi children due to Haemophilus influenzae type b meningitis. *Ann Trop Paediatr* 1997; **17**: 5–8.
- Sakata H, Maruyama S. A study of bacterial meningitis in Hokkaido between 1994 and 1998. *Kansenshogaku zasshi* 2000; **74**: 339–44.
- Sung RYT, Senok AC, Ho A, Oppenheimer SJ, Davies DP. Meningitis in Hong Kong children, with special reference to the infrequency of haemophilus and meningococcal infection. *J Paediatr Child Health* 1997; **33**: 296–9.
- Sutinen J, Sombrero L, Paladin FJE, et al. Etiology of central nervous system infections in the Philippines and the role of serum C-reactive protein in excluding acute bacterial meningitis. *Int J Infect Dis* 1999; **3**: 88–93.
- Wang L, Tian F, Chen G, et al. Distribution of pathogenic bacteria and analysis of drug resistance of purulent meningitis in Chongqing Area from 2009 to 2013. *Journal of Pediatric Pharmacy* 2013; **19**: 37–42. In Chinese
- Yang Y, Leng Z, Shen X, et al. Acute bacterial meningitis in children in Hefei, China 1990-1992. *Chin Med J* 1996; **109**: 385–8.
- Zaidi AKM, Hassan K, Razzaq L, Waheed M, Group SM. Surveillance of pneumococcal meningitis among children in Sindh, southern Pakistan. *Clin Infect Dis* 2009; **48**: S129–35.

**Webappendix 5: Summary estimates of pathogen-specific pneumonia morbidity and mortality**

*Probe approach*

We used the probe approach to estimate the proportion of pneumonia cases and deaths attributable to pneumococcus.^3,7^ We first identified efficacy values from PCV and Hib vaccine randomized control trials (RCT) and effectiveness results from case-control studies with random or systematic allocation of vaccine. We used data from trials and studies that used case definitions that aligned closely with WHO pneumonia case definitions.^8–10^ Since vaccine trials and studies did not assess efficacy against pneumonia mortality, we used values of efficacy and effectiveness against radiography-confirmed, primary end-point pneumonia (i.e. consolidation),^10^ with relevant adjustments, as a proxy for the proportion of pneumonia deaths caused by each pathogen. Arithmetically inherent in using this proxy is that the pneumococcal pneumonia case fatality ratio (CFR) is equivalent to the Hib pneumonia CFR, and they are both equal to the CFR for non-pneumococcal, non-Hib pneumonia cases. The direction of the bias introduced by using this proxy has been described previously—it most likely underestimates pneumococcal and Hib deaths in high-mortality settings and may overestimate deaths in low-mortality settings.^3^ We used values of PCV and Hib vaccine efficacy and effectiveness against WHO-defined clinical and severe pneumonia^8,9^ to estimate the proportions of clinical and severe pneumonia cases associated with each pathogen.

*Adjustments*

PCV and Hib vaccine efficacy and effectiveness values were adjusted to account for imperfect efficacy against vaccine-type (VT) pneumococcal pneumonia. Since true efficacy values for these parameters are not known, we used as a proxy the observed efficacy against VT invasive pneumococcal disease (IPD) on a trial-by-trial basis. We estimated the degree of bias introduced by this proxy measure for pneumococcus using two sensitivity analyses described previously.^3^ PCV efficacy values were also adjusted to account for the proportion of pneumonia that was VT in the control group. As this value is not available, we used as a proxy the proportion of IPD cases that were VT from the control group. For pneumococcus, PCV efficacy values were also adjusted to account for the proportion of pneumonia cases caused by Hib, since all PCV trials were conducted in the context of Hib vaccine use. Below are the formulas for calculating the proportion of deaths and cases due to pneumococcus and Hib:

$${\% Pneumonia cases}_{Spn}= \frac{{VE}_{Pneumonia, PCV}}{\left( {VE}_{IPD, PCV} \right) \times\left( {VT}_{Control} \right) \times\left( {1-\% Pneumonia cases}_{Hib} \right)}$$

$${\% Pneumonia cases}_{Hib}= \frac{{VE}_{Pneumonia, Hib}}{{VE}_{IHD, Hib}}$$

Where $VE$ is PCV or Hib vaccine efficacy against a pneumonia or invasive disease, $IPD$ is invasive pneumococcal disease, $IHD$ is invasive Hib disease, ${VT}_{Control}$ is the proportion of pneumococcal disease caused by vaccine serotypes in the control group of the RCT.

*Summary Estimates*

Adjusted PCV and Hib vaccine efficacy and effectiveness estimates were each combined in a random effects meta-analysis to determine the pathogen-specific fraction of pneumonia cases for each pneumonia case definition (i.e. clinical, severe and radiography-confirmed pneumonia). We used the delta method to calculate standard errors for each trial. Random effects meta-analyses were used throughout the pathogen-specific pneumonia modeling because fixed effects models do not incorporate between study variability. In addition, fixed effects models produce biased results in most cases.^11^ The value of random effects meta-analysis methods has been called into question. However, we determined that this was the most appropriate way to combine information from different studies into a single input parameter value with associated uncertainty.

*Uncertainty*

The primary sources of uncertainty in the pneumonia models were the between-study variation in vaccine efficacy estimates. For pneumococcal pneumonia, the Gambia trial^12^ significantly pushes the meta-analysis upward. In contrast, the Lombok trial^13^ pulls the overall estimate toward the null for Hib pneumonia. As we assume all Hib pneumonia deaths occur only in children less than two years, the upper limit uncertainty bounds, reflect the possibility that there may be deaths in older children. Because of this between-study variation, we chose to use a jackknife, leave-one-study-out approach for the upper and lower bounds of the pneumococcal and Hib pneumonia fractions. A limitation of using the jackknife method with small sample sizes is that standard errors are not reliable, which is why we chose to use the upper and lower bounds for estimating uncertainty. The uncertainty bounds account for both sampling and non-sampling error. Assessing non-sampling error has been a limitation of disease burden models. This approach underscores the uncertainty arising from limited sample sizes, population variability, and the paucity of data that is usually available to estimate disease burden. We were unable to account these sources of uncertainty as these values (e.g., vaccine coverage, population estimates) were not available. Nevertheless, excluding all sources of model uncertainty leads to uncertainty ranges that are narrower than the true uncertainty.

**Webappendix figure 1: Proportion of radiography-confirmed pneumonia attributable to pneumococcus**

**Webappendix figure 2: Proportion of severe pneumonia attributable to pneumococcus**

**Webappendix figure 3: Proportion of clinical pneumonia attributable to pneumococcus**

**Webappendix figure 4: Proportion of radiography-confirmed pneumonia attributable to Hib**

**Webappendix figure 5: Proportion of severe pneumonia attributable to Hib**

**Webappendix figure 6: Proportion of clinical pneumonia attributable to Hib**

**Webappendix 6: Estimates of pathogen-specific meningitis morbidity and mortality**

We used data from studies reporting on the distribution of meningitis cases and adjusted these estimates by the relative pathogen-specific meningitis CFR to calculate the proportion of meningitis deaths due to pneumococcus and Hib. We constrained the total contribution of the most common causes of bacterial meningitis (i.e. pneumococcus, Hib, and *Neisseria meningitidis*) to all-cause meningitis cases and deaths to 83%, based on an analysis of studies reporting on multiple meningitis etiologies using diagnostic techniques with equivalent sensitivity for all pathogens. To derive the proportion of all-cause meningitis deaths attributable to each pathogen, we used the following equation.

$${\% Meningitis deaths}_{x}= \frac{{Meningitis CFR}_{x} \times{\% Meningitis cases}_{x}}{\sum_{i}^{z} ({Meningitis CFR}_{i} \times{\% Meningitis cases}_{i})}$$

where ${Meningitis CFR}_{x}$ is the case fatality ratio (CFR) for pathogen $x$, ${\% Meningitis cases}_{x}$ is the proportion of meningitis cases attributable to pathogen $x$, and the denominator on the right-hand side of the equation is the sum of the products for each pathogen (i.e. $i$ ranges over each of the three pathogens of interest—pneumococcus, Hib, and *Neisseria meningitidis*).

The proportion of meningitis cases attributable to pneumococcus and Hib in the equation above was determined by using data from observational studies identified from the literature rather than the vaccine probe approach was used for estimating pathogen-specific pneumonia cases and deaths. The basis for this decision was that the etiologic diagnostic yield from investigations of meningitis cases is high when conducted with appropriate clinical and microbiologic methods, unlike pneumonia. We included only surveillance studies lasting more than 12 months and conducted in the absence of conjugate vaccine. Data from studies reporting on etiologically-confirmed bacterial meningitis cases attributable to the most common causes of bacterial meningitis (i.e. pneumococcus, Hib, and *Neisseria meningitidis*) were combined in random effects meta-analyses to develop region-specific summary estimates of the relative distribution of meningitis cases by these pathogens.

We estimated pathogen-specific meningitis CFR by random effects meta-analyses of data from the systematic literature review, stratified by child mortality setting: low (<30 deaths per 1 000 live births), medium (30-<75), high (75-<150), and very high (≥150). Because reported CFR values reflect mortality in children who access care at a study site or health facility, we adjusted them to account for the higher CFR (i.e. 90% for all pathogens) assumed for those not accessing care. There is no measure of care seeking for children with meningitis in standardized surveys from China; therefore, we used province-specific values for the proportion of children seeking care for minor illnesses (such as fever or diarrhea) from China Family Panel Studies as a proxy. We assumed 100% access to care in provinces with child mortality below 30 deaths per 1 000 live births.

**Webappendix 7: Methods used to estimate pathogen-specific pneumonia, meningitis and non-pneumonia, non-meningitis (NPNM) cases and deaths in China**

In the present study, we estimated the disease burden separately for the three clinical syndromes associated with pneumococcus and Hib: pneumonia, meningitis, and invasive non-pneumonia, non-meningitis disease (NPNM). We explained the three models used separately for the three syndromes in the manuscript, and the following are some additional information to explain how we calibrated each model from a global one^3^ to the China scenarios.

**Pathogen-specific pneumonia model**

1. Pathogen-specific pneumonia deaths and cases were prepared by applying estimates of the proportion of pneumonia deaths and cases attributable to each pathogen to all-cause pneumonia mortality and morbidity estimates. We obtained annual all-cause pneumonia deaths and cases from GBD IHME, and the data were derived from the Disease Surveillance Point System maintained by the Chinese CDC, the Maternal and Child Health Surveillance System maintained by the National Office for Maternal and Child Health Surveillance of China, and various surveys, cancer registries and censuses in China. The data sources we applied were different from those used in previous global publication,^3^ which used country-specific all-cause pneumonia deaths and cases prepared by WHO and the Maternal and Child Epidemiology Estimation (MCEE) collaboration. We changed the data source because the GBD IHME data combined several reliable data sources in China in a relatively scientific way, which could better represent the situations in China.
2. Efficacy against pneumonia endpoints from randomized controlled PCV and Hib vaccine trials were used to estimate the attributable fraction for each pathogen. We adopted the meta-analysis results of aetiological fraction allocation for pneumonia cases, and could only obtain global estimates due to very limited number of domestic vaccine probe studies. In other words, we used the same literature and efficacy estimates for pneumonia cases as the previous global publication.^3^ Globally, we meta-analysed efficacy and effectiveness results from PCV and Hib vaccine randomised controlled trials and case-control studies with random or systematic allocation of treatment with PCV and Hib vaccine and all-cause pneumonia case definitions. None of the included studies were conducted in China, but some were done in economically, geographically or epidemiologically relevant settings (i.e. Latin American, Indonesia, Philippines). However, due to economic and ethical constraints, further vaccine probe studies to inform the pneumonia estimates are unlikely to be conducted worldwide, and it seems infeasible to get domestic pneumonia aetiological fraction allocation estimates, for no available studies were conducted in China, and there won’t be more domestic studies due to the above-mentioned constraints.
3. We also adjusted for vaccine use in China, which has been mentioned for many times in the manuscript, since the previous global publication assumed that the national Hib vaccine and PCV coverage in China was 0% for the lack of reliable vaccine coverage data.^3^ Our study estimated Hib vaccine and PCV coverage in China using the number of vaccine doses from the Chinese CDC, and proved the importance of vaccine adjustment in China. The same adjustments were done for pathogen-specific meningitis and NPNM, and we did not repeat this in the description of other two models.

**Pathogen-specific meningitis model**

1. Similar to pathogen-specific pneumonia, we used the proportion of meningitis cases attributable to each pathogen derived from literature and all-cause meningitis deaths to estimate pathogen-specific meningitis deaths in China. We adopted the meta-analysis results of aetiological fraction allocation from a global overview due to the lack of vaccine probe studies in China. We also obtained annual all-cause meningitis deaths from GBD IHME.
2. Different from pathogen-specific pneumonia, to calculate pathogen-specific meningitis cases, we divided pathogen-specific meningitis deaths by country-specific and pathogen-specific meningitis case-fatality ratio (CFR) estimates, instead of directly obtaining meningitis cases by multiplying pathogen-specific proportions and all-cause cases. Note that since no studies were identified to report the distribution of meningitis deaths by pathogen, we used studies reporting the distribution of meningitis cases and adjusted them by the relative pathogen-specific meningitis CFR to calculate the proportion of pathogen-specific meningitis deaths. For each province, we prepared summary estimates for the required parameters by meta-analyzing data reported in literature from Asia after adding more domestic studies in Chinese.

**Pathogen-specific invasive non-pneumonia, non-meningitis model**

1. Based on the calculated meningitis cases, we estimated pathogen-specific morbidity due to other invasive syndromes (e.g., sepsis) from studies reporting on the ratio of pathogen-specific NPNM cases to pathogen-specific meningitis cases. These ratios were combined using random effects meta-analyses, stratifying the data first by child mortality setting in a global setting. For pneumococcus, we stratified NPNM cases by severe and non-severe cases. NPNM deaths were estimated by multiplying country-specific NPNM cases (severe cases for pneumococcus) by country-specific NPNM CFR. The latter was derived by estimating a mortality setting-specific ratio of pathogen-specific NPNM CFR and pathogen-specific meningitis CFR, and applying that to the province-specific pneumococcal meningitis CFR.
2. Note that there are very few studies globally reporting on NPNM CFR (5 each for pneumococcus and Hib) and only two of these studies for pneumococcus were conducted in epidemiologically relevant settings (None in China). Because of the paucity of data for pathogen-specific NPNM from relevant settings, we chose to use the ratio from other settings and then anchor our province-specific pathogen NPNM CFR estimates on the province-specific pathogen meningitis CFR estimates. Because we chose to anchor our pathogen NPNM estimates on the pathogen meningitis estimates, we used the pathogen meningitis uncertainty as the primary source of uncertainty in our estimates.

**References**

1 Wolfson L, O’Brien K, Watt J, et al. Methods to estimate the global burden of disease due to Haemophilus influenzae type b and Streptococcus pneumoniae in children less than 5 years of age. *Lancet Web annex* 2009; **374**: 893–902.

2 Knoll M, O’Brien K, Henkle E, et al. Global literature review of Haemophilus influenzae type b and Streptococcus pneumoniae invasive disease among children less than five years of age, 1980-2005. *Geneva, Switzerland: World Health Organization* 2009.

3 Wahl B, O'Brien KL, Greenbaum A, et al. Burden of Streptococcus pneumoniae and Haemophilus influenzae type b disease in children in the era of conjugate vaccines: global, regional, and national estimates for 2000-15. *Lancet Glob Health* 2018; **6**: e744–57.

4 O'Brien KL, Wolfson LJ, Watt JP, et al. Burden of disease caused by Streptococcus pneumoniae in children younger than 5 years: global estimates. *Lancet* 2009; **374**: 893–902.

5 Watt JP, Wolfson LJ, O'Brien KL, et al. Burden of disease caused by Haemophilus influenzae type b in children younger than 5 years: global estimates. *Lancet* 2009; **374**: 903–11.

6 Lucero MG, Dulalia VE, Nillos LT, et al. Pneumococcal conjugate vaccines for preventing vaccine-type invasive pneumococcal disease and X-ray defined pneumonia in children less than two years of age. *The Cochrane database of systematic reviews* 2009; **4**: CD004977.

7 Mulholland EK. Use of vaccine trials to estimate burden of disease. *J Health Popul Nutr* 2004; **22**: 257–67.

8 World Health Organization. Programme for the control of acute respiratory infections: technical bases for the WHO recommendations on the management of pneumonia in children at first level health facilities. *Wkly Epidemiol Rec* 1991; **66**: 233–6.

9 World Health Organization. Handbook IMCI: Integrated management of childhood illness. *Geneva, Switzerland: World Health Organization* 2005.

10 Cherian T, Mulholland EK, Carlin JB, et al. Standardized interpretation of pediatric chest radiographs for the diagnosis of pneumonia in epidemiological studies. *Bull World Health Organ* 2005; **83**: 353–9.

11 Schmidt FL, Oh IS, Hayes TL. Fixed-versus random-effects models in meta-analysis: model properties and an empirical comparison of differences in results. *Br J Math Stat Psychol* 2009; **62**: 97–128.

12 Cutts FT, Zaman SM, Enwere G, et al. Efficacy of nine-valent pneumococcal conjugate vaccine against pneumonia and invasive pneumococcal disease in The Gambia: randomised, double-blind, placebo-controlled trial. *Lancet* 2005; **365**: 1139–46.

13 Gessner BD, Sutanto A, Linehan M, et al. Incidences of vaccine-preventable Haemophilus influenzae type b pneumonia and meningitis in Indonesian children: hamlet-randomised vaccine-probe trial. *Lancet* 2005; **365**: 43–52.

**Webappendix 8: Pneumococcal deaths in China by region, province, and year**

| **Year** | **Province** | **Pneumococcal deaths** | | | | | | **Pneumococcal pneumonia deaths** | | | | | | **Pneumococcal meningitis deaths** | | | | | | **Pneumococcal severe NPNM deaths** | | | | | |
| --- | --- | --- | --- | --- | --- | --- | --- | --- | --- | --- | --- | --- | --- | --- | --- | --- | --- | --- | --- | --- | --- | --- | --- | --- | --- |
|  |  | **Number** | | | **Rate per 100 000** | | | **Number** | | | **Rate per 100 000** | | | **Number** | | | **Rate per 100 000** | | | **Number** | | | **Rate per 100 000** | | |
|  |  | **Mean** | **UR** | | **Mean** | **UR** | | **Mean** | **UR** | | **Mean** | **UR** | | **Mean** | **UR** | | **Mean** | **UR** | | **Mean** | **UR** | | **Mean** | **UR** | |
| 2010 | Anhui | 699 | 480 | 781 | 19 | 13 | 21 | 525 | 373 | 548 | 14 | 10 | 15 | 92 | 57 | 123 | 2 | 2 | 3 | 82 | 51 | 110 | 2 | 1 | 3 |
| 2011 | Anhui | 622 | 427 | 696 | 17 | 12 | 19 | 464 | 329 | 483 | 13 | 9 | 13 | 84 | 52 | 112 | 2 | 1 | 3 | 74 | 46 | 100 | 2 | 1 | 3 |
| 2012 | Anhui | 548 | 376 | 613 | 15 | 10 | 17 | 407 | 289 | 424 | 11 | 8 | 12 | 75 | 46 | 100 | 2 | 1 | 3 | 66 | 41 | 89 | 2 | 1 | 2 |
| 2013 | Anhui | 481 | 330 | 538 | 13 | 9 | 15 | 356 | 253 | 371 | 10 | 7 | 10 | 66 | 41 | 88 | 2 | 1 | 2 | 59 | 36 | 79 | 2 | 1 | 2 |
| 2014 | Anhui | 429 | 294 | 479 | 12 | 8 | 13 | 320 | 227 | 333 | 9 | 6 | 9 | 58 | 36 | 77 | 2 | 1 | 2 | 51 | 32 | 69 | 1 | 1 | 2 |
| 2015 | Anhui | 378 | 260 | 423 | 10 | 7 | 12 | 283 | 201 | 295 | 8 | 6 | 8 | 51 | 31 | 68 | 1 | 1 | 2 | 45 | 28 | 60 | 1 | 1 | 2 |
| 2016 | Anhui | 324 | 222 | 362 | 9 | 6 | 10 | 242 | 172 | 253 | 6 | 5 | 7 | 43 | 27 | 58 | 1 | 1 | 2 | 38 | 24 | 52 | 1 | 1 | 1 |
| 2017 | Anhui | 277 | 190 | 310 | 7 | 5 | 8 | 208 | 147 | 217 | 5 | 4 | 6 | 37 | 23 | 49 | 1 | 1 | 1 | 33 | 20 | 44 | 1 | 1 | 1 |
| 2010 | Beijing | 43 | 30 | 47 | 5 | 4 | 6 | 34 | 24 | 35 | 4 | 3 | 4 | 5 | 3 | 6 | 1 | 0 | 1 | 4 | 3 | 5 | 0 | 0 | 1 |
| 2011 | Beijing | 43 | 30 | 48 | 7 | 5 | 8 | 34 | 24 | 36 | 6 | 4 | 6 | 5 | 3 | 6 | 1 | 0 | 1 | 4 | 3 | 6 | 1 | 0 | 1 |
| 2012 | Beijing | 43 | 30 | 48 | 5 | 4 | 6 | 34 | 24 | 36 | 4 | 3 | 4 | 5 | 3 | 6 | 1 | 0 | 1 | 4 | 3 | 6 | 1 | 0 | 1 |
| 2013 | Beijing | 45 | 31 | 50 | 5 | 4 | 6 | 36 | 25 | 37 | 4 | 3 | 5 | 5 | 3 | 7 | 1 | 0 | 1 | 4 | 3 | 6 | 1 | 0 | 1 |
| 2014 | Beijing | 48 | 33 | 53 | 6 | 4 | 6 | 38 | 27 | 39 | 4 | 3 | 5 | 5 | 3 | 7 | 1 | 0 | 1 | 5 | 3 | 6 | 1 | 0 | 1 |
| 2015 | Beijing | 49 | 34 | 54 | 6 | 4 | 6 | 39 | 28 | 40 | 5 | 3 | 5 | 5 | 3 | 7 | 1 | 0 | 1 | 5 | 3 | 7 | 1 | 0 | 1 |
| 2016 | Beijing | 49 | 34 | 54 | 6 | 4 | 6 | 39 | 27 | 40 | 4 | 3 | 5 | 5 | 3 | 7 | 1 | 0 | 1 | 5 | 3 | 6 | 1 | 0 | 1 |
| 2017 | Beijing | 45 | 31 | 50 | 5 | 3 | 5 | 36 | 25 | 37 | 4 | 3 | 4 | 5 | 3 | 7 | 1 | 0 | 1 | 4 | 3 | 6 | 0 | 0 | 1 |
| 2010 | Chongqing | 259 | 179 | 285 | 18 | 12 | 19 | 210 | 149 | 219 | 14 | 10 | 15 | 26 | 16 | 35 | 2 | 1 | 2 | 23 | 14 | 31 | 2 | 1 | 2 |
| 2011 | Chongqing | 235 | 162 | 259 | 16 | 11 | 17 | 188 | 133 | 196 | 13 | 9 | 13 | 25 | 15 | 33 | 2 | 1 | 2 | 22 | 14 | 29 | 1 | 1 | 2 |
| 2012 | Chongqing | 209 | 145 | 231 | 13 | 9 | 15 | 167 | 118 | 174 | 11 | 7 | 11 | 23 | 14 | 30 | 1 | 1 | 2 | 20 | 13 | 27 | 1 | 1 | 2 |
| 2013 | Chongqing | 188 | 129 | 207 | 12 | 8 | 13 | 148 | 105 | 154 | 10 | 7 | 10 | 21 | 13 | 28 | 1 | 1 | 2 | 19 | 12 | 25 | 1 | 1 | 2 |
| 2014 | Chongqing | 169 | 117 | 188 | 11 | 7 | 12 | 133 | 94 | 138 | 8 | 6 | 9 | 19 | 12 | 26 | 1 | 1 | 2 | 17 | 11 | 23 | 1 | 1 | 1 |
| 2015 | Chongqing | 150 | 103 | 166 | 9 | 7 | 11 | 117 | 83 | 122 | 7 | 5 | 8 | 17 | 11 | 23 | 1 | 1 | 1 | 15 | 10 | 21 | 1 | 1 | 1 |
| 2016 | Chongqing | 127 | 88 | 141 | 8 | 6 | 9 | 99 | 70 | 104 | 6 | 5 | 7 | 15 | 9 | 20 | 1 | 1 | 1 | 13 | 8 | 18 | 1 | 1 | 1 |
| 2017 | Chongqing | 107 | 74 | 119 | 7 | 5 | 7 | 84 | 60 | 88 | 5 | 4 | 5 | 12 | 8 | 16 | 1 | 0 | 1 | 11 | 7 | 15 | 1 | 0 | 1 |
| 2010 | Fujian | 325 | 225 | 357 | 13 | 9 | 14 | 263 | 186 | 274 | 11 | 8 | 11 | 33 | 20 | 44 | 1 | 1 | 2 | 29 | 18 | 39 | 1 | 1 | 2 |
| 2011 | Fujian | 296 | 205 | 326 | 14 | 10 | 15 | 237 | 168 | 247 | 11 | 8 | 12 | 31 | 19 | 42 | 1 | 1 | 2 | 28 | 17 | 37 | 1 | 1 | 2 |
| 2012 | Fujian | 267 | 184 | 295 | 12 | 8 | 13 | 212 | 150 | 221 | 9 | 7 | 10 | 29 | 18 | 39 | 1 | 1 | 2 | 26 | 16 | 35 | 1 | 1 | 2 |
| 2013 | Fujian | 241 | 166 | 267 | 10 | 7 | 11 | 189 | 134 | 197 | 8 | 6 | 8 | 27 | 17 | 37 | 1 | 1 | 2 | 24 | 15 | 33 | 1 | 1 | 1 |
| 2014 | Fujian | 222 | 153 | 246 | 9 | 6 | 10 | 173 | 123 | 180 | 7 | 5 | 7 | 26 | 16 | 35 | 1 | 1 | 1 | 23 | 14 | 31 | 1 | 1 | 1 |
| 2015 | Fujian | 199 | 137 | 220 | 8 | 6 | 9 | 154 | 109 | 161 | 6 | 5 | 7 | 24 | 15 | 32 | 1 | 1 | 1 | 21 | 13 | 28 | 1 | 1 | 1 |
| 2016 | Fujian | 172 | 119 | 191 | 7 | 5 | 8 | 133 | 95 | 139 | 5 | 4 | 6 | 21 | 13 | 28 | 1 | 1 | 1 | 18 | 11 | 25 | 1 | 0 | 1 |
| 2017 | Fujian | 149 | 103 | 165 | 6 | 4 | 7 | 115 | 82 | 120 | 5 | 3 | 5 | 18 | 11 | 24 | 1 | 0 | 1 | 16 | 10 | 21 | 1 | 0 | 1 |
| 2010 | Gansu | 706 | 465 | 778 | 44 | 29 | 49 | 571 | 405 | 596 | 36 | 25 | 37 | 71 | 32 | 97 | 4 | 2 | 6 | 64 | 28 | 86 | 4 | 2 | 5 |
| 2011 | Gansu | 631 | 443 | 714 | 42 | 30 | 48 | 498 | 353 | 519 | 33 | 24 | 35 | 70 | 48 | 103 | 5 | 3 | 7 | 62 | 42 | 92 | 4 | 3 | 6 |
| 2012 | Gansu | 559 | 390 | 631 | 40 | 28 | 45 | 433 | 307 | 452 | 31 | 22 | 32 | 67 | 44 | 95 | 5 | 3 | 7 | 59 | 39 | 85 | 4 | 3 | 6 |
| 2013 | Gansu | 493 | 341 | 554 | 35 | 24 | 39 | 374 | 265 | 389 | 27 | 19 | 28 | 63 | 40 | 87 | 4 | 3 | 6 | 56 | 36 | 78 | 4 | 3 | 6 |
| 2014 | Gansu | 445 | 305 | 498 | 30 | 21 | 34 | 331 | 235 | 345 | 23 | 16 | 23 | 60 | 37 | 81 | 4 | 3 | 6 | 54 | 33 | 72 | 4 | 2 | 5 |
| 2015 | Gansu | 389 | 267 | 436 | 27 | 18 | 30 | 288 | 204 | 300 | 20 | 14 | 21 | 54 | 33 | 72 | 4 | 2 | 5 | 48 | 30 | 64 | 3 | 2 | 4 |
| 2016 | Gansu | 332 | 228 | 372 | 23 | 16 | 25 | 245 | 174 | 256 | 17 | 12 | 17 | 46 | 28 | 62 | 3 | 2 | 4 | 41 | 25 | 55 | 3 | 2 | 4 |
| 2017 | Gansu | 284 | 195 | 318 | 19 | 13 | 22 | 211 | 150 | 220 | 14 | 10 | 15 | 39 | 24 | 52 | 3 | 2 | 4 | 35 | 21 | 46 | 2 | 1 | 3 |
| 2010 | Guangdong | 964 | 666 | 1062 | 17 | 12 | 19 | 772 | 547 | 805 | 14 | 10 | 14 | 102 | 63 | 136 | 2 | 1 | 2 | 91 | 56 | 122 | 2 | 1 | 2 |
| 2011 | Guangdong | 886 | 612 | 979 | 17 | 11 | 18 | 702 | 498 | 731 | 13 | 9 | 14 | 98 | 60 | 131 | 2 | 1 | 2 | 87 | 54 | 117 | 2 | 1 | 2 |
| 2012 | Guangdong | 820 | 565 | 907 | 13 | 9 | 14 | 644 | 457 | 671 | 10 | 7 | 10 | 93 | 57 | 125 | 1 | 1 | 2 | 83 | 51 | 111 | 1 | 1 | 2 |
| 2013 | Guangdong | 772 | 532 | 856 | 12 | 8 | 13 | 602 | 427 | 627 | 9 | 7 | 10 | 90 | 56 | 121 | 1 | 1 | 2 | 80 | 50 | 107 | 1 | 1 | 2 |
| 2014 | Guangdong | 745 | 514 | 826 | 11 | 8 | 12 | 580 | 411 | 604 | 9 | 6 | 9 | 87 | 54 | 117 | 1 | 1 | 2 | 78 | 48 | 105 | 1 | 1 | 2 |
| 2015 | Guangdong | 705 | 486 | 782 | 10 | 7 | 12 | 548 | 389 | 571 | 8 | 6 | 8 | 83 | 51 | 111 | 1 | 1 | 2 | 74 | 46 | 99 | 1 | 1 | 1 |
| 2016 | Guangdong | 643 | 443 | 714 | 9 | 6 | 10 | 500 | 354 | 521 | 7 | 5 | 8 | 76 | 47 | 102 | 1 | 1 | 1 | 68 | 42 | 91 | 1 | 1 | 1 |
| 2017 | Guangdong | 580 | 399 | 642 | 8 | 5 | 9 | 451 | 320 | 470 | 6 | 4 | 6 | 68 | 42 | 91 | 1 | 1 | 1 | 60 | 37 | 81 | 1 | 0 | 1 |
| 2010 | Guangxi | 488 | 338 | 536 | 14 | 10 | 15 | 397 | 282 | 414 | 11 | 8 | 12 | 48 | 30 | 64 | 1 | 1 | 2 | 43 | 26 | 57 | 1 | 1 | 2 |
| 2011 | Guangxi | 460 | 318 | 506 | 13 | 9 | 14 | 372 | 264 | 388 | 10 | 7 | 11 | 47 | 29 | 63 | 1 | 1 | 2 | 41 | 26 | 56 | 1 | 1 | 2 |
| 2012 | Guangxi | 431 | 298 | 474 | 12 | 8 | 13 | 347 | 246 | 361 | 9 | 7 | 10 | 44 | 27 | 60 | 1 | 1 | 2 | 40 | 24 | 53 | 1 | 1 | 1 |
| 2013 | Guangxi | 401 | 277 | 441 | 11 | 7 | 12 | 321 | 228 | 335 | 8 | 6 | 9 | 42 | 26 | 56 | 1 | 1 | 1 | 37 | 23 | 50 | 1 | 1 | 1 |
| 2014 | Guangxi | 384 | 266 | 424 | 10 | 7 | 11 | 308 | 218 | 321 | 8 | 6 | 8 | 41 | 25 | 54 | 1 | 1 | 1 | 36 | 22 | 48 | 1 | 1 | 1 |
| 2015 | Guangxi | 360 | 249 | 397 | 9 | 6 | 10 | 289 | 205 | 301 | 8 | 5 | 8 | 38 | 23 | 51 | 1 | 1 | 1 | 34 | 21 | 45 | 1 | 1 | 1 |
| 2016 | Guangxi | 325 | 225 | 358 | 8 | 6 | 9 | 261 | 185 | 273 | 7 | 5 | 7 | 34 | 21 | 45 | 1 | 1 | 1 | 30 | 19 | 40 | 1 | 0 | 1 |
| 2017 | Guangxi | 290 | 200 | 319 | 7 | 5 | 8 | 234 | 166 | 243 | 6 | 4 | 6 | 30 | 18 | 40 | 1 | 0 | 1 | 26 | 16 | 36 | 1 | 0 | 1 |
| 2010 | Guizhou | 615 | 428 | 667 | 25 | 17 | 27 | 526 | 373 | 549 | 21 | 15 | 22 | 47 | 29 | 63 | 2 | 1 | 3 | 42 | 26 | 56 | 2 | 1 | 2 |
| 2011 | Guizhou | 533 | 371 | 579 | 21 | 15 | 23 | 453 | 321 | 472 | 18 | 13 | 18 | 42 | 26 | 57 | 2 | 1 | 2 | 37 | 23 | 50 | 1 | 1 | 2 |
| 2012 | Guizhou | 460 | 320 | 501 | 18 | 13 | 20 | 390 | 277 | 406 | 15 | 11 | 16 | 37 | 23 | 50 | 1 | 1 | 2 | 33 | 21 | 45 | 1 | 1 | 2 |
| 2013 | Guizhou | 399 | 278 | 435 | 16 | 11 | 17 | 337 | 239 | 351 | 13 | 9 | 14 | 33 | 20 | 44 | 1 | 1 | 2 | 29 | 18 | 39 | 1 | 1 | 2 |
| 2014 | Guizhou | 352 | 245 | 384 | 14 | 9 | 15 | 297 | 211 | 310 | 11 | 8 | 12 | 29 | 18 | 39 | 1 | 1 | 2 | 26 | 16 | 35 | 1 | 1 | 1 |
| 2015 | Guizhou | 303 | 211 | 330 | 12 | 8 | 13 | 256 | 181 | 267 | 10 | 7 | 10 | 25 | 15 | 33 | 1 | 1 | 1 | 22 | 14 | 30 | 1 | 1 | 1 |
| 2016 | Guizhou | 255 | 178 | 278 | 10 | 7 | 11 | 216 | 153 | 225 | 9 | 6 | 9 | 21 | 13 | 28 | 1 | 1 | 1 | 18 | 11 | 25 | 1 | 0 | 1 |
| 2017 | Guizhou | 217 | 151 | 235 | 8 | 5 | 8 | 184 | 131 | 192 | 7 | 5 | 7 | 17 | 11 | 23 | 1 | 0 | 1 | 15 | 9 | 21 | 1 | 0 | 1 |
| 2010 | Hainan | 188 | 131 | 205 | 29 | 20 | 32 | 158 | 112 | 164 | 25 | 17 | 26 | 16 | 10 | 22 | 3 | 2 | 3 | 14 | 9 | 19 | 2 | 1 | 3 |
| 2011 | Hainan | 179 | 124 | 195 | 29 | 20 | 31 | 149 | 106 | 156 | 24 | 17 | 25 | 16 | 10 | 21 | 3 | 2 | 3 | 14 | 9 | 19 | 2 | 1 | 3 |
| 2012 | Hainan | 169 | 117 | 185 | 26 | 18 | 29 | 141 | 100 | 147 | 22 | 16 | 23 | 15 | 9 | 20 | 2 | 1 | 3 | 13 | 8 | 18 | 2 | 1 | 3 |
| 2013 | Hainan | 161 | 112 | 176 | 25 | 17 | 27 | 134 | 95 | 140 | 21 | 15 | 22 | 14 | 9 | 19 | 2 | 1 | 3 | 13 | 8 | 17 | 2 | 1 | 3 |
| 2014 | Hainan | 157 | 109 | 172 | 24 | 17 | 26 | 131 | 93 | 136 | 20 | 14 | 21 | 14 | 9 | 19 | 2 | 1 | 3 | 12 | 8 | 17 | 2 | 1 | 3 |
| 2015 | Hainan | 151 | 104 | 164 | 23 | 16 | 25 | 126 | 89 | 131 | 19 | 14 | 20 | 13 | 8 | 18 | 2 | 1 | 3 | 12 | 7 | 16 | 2 | 1 | 2 |
| 2016 | Hainan | 141 | 98 | 154 | 22 | 15 | 24 | 118 | 83 | 123 | 18 | 13 | 19 | 12 | 8 | 16 | 2 | 1 | 3 | 11 | 7 | 15 | 2 | 1 | 2 |
| 2017 | Hainan | 127 | 88 | 139 | 19 | 13 | 21 | 107 | 76 | 111 | 16 | 11 | 17 | 11 | 7 | 15 | 2 | 1 | 2 | 10 | 6 | 13 | 1 | 1 | 2 |
| 2010 | Hebei | 869 | 589 | 994 | 17 | 12 | 20 | 572 | 406 | 596 | 11 | 8 | 12 | 157 | 97 | 210 | 3 | 2 | 4 | 140 | 86 | 187 | 3 | 2 | 4 |
| 2011 | Hebei | 835 | 566 | 956 | 17 | 12 | 20 | 546 | 387 | 569 | 11 | 8 | 12 | 153 | 94 | 205 | 3 | 2 | 4 | 136 | 84 | 182 | 3 | 2 | 4 |
| 2012 | Hebei | 794 | 538 | 910 | 16 | 11 | 18 | 519 | 368 | 541 | 10 | 7 | 11 | 145 | 90 | 195 | 3 | 2 | 4 | 130 | 80 | 174 | 3 | 2 | 3 |
| 2013 | Hebei | 752 | 510 | 862 | 15 | 10 | 17 | 491 | 348 | 512 | 10 | 7 | 10 | 138 | 85 | 185 | 3 | 2 | 4 | 123 | 76 | 165 | 2 | 1 | 3 |
| 2014 | Hebei | 732 | 496 | 838 | 14 | 10 | 16 | 479 | 340 | 499 | 9 | 7 | 10 | 134 | 83 | 179 | 3 | 2 | 3 | 119 | 74 | 160 | 2 | 1 | 3 |
| 2015 | Hebei | 697 | 473 | 798 | 14 | 9 | 16 | 458 | 325 | 477 | 9 | 6 | 9 | 127 | 78 | 170 | 3 | 2 | 3 | 113 | 70 | 151 | 2 | 1 | 3 |
| 2016 | Hebei | 646 | 438 | 739 | 13 | 9 | 15 | 426 | 302 | 444 | 8 | 6 | 9 | 116 | 72 | 156 | 2 | 1 | 3 | 104 | 64 | 139 | 2 | 1 | 3 |
| 2017 | Hebei | 583 | 395 | 666 | 11 | 8 | 13 | 386 | 274 | 402 | 7 | 5 | 8 | 104 | 64 | 140 | 2 | 1 | 3 | 93 | 57 | 124 | 2 | 1 | 2 |
| 2010 | Heilongjiang | 197 | 134 | 225 | 10 | 7 | 11 | 131 | 93 | 136 | 6 | 5 | 7 | 35 | 22 | 47 | 2 | 1 | 2 | 31 | 19 | 42 | 2 | 1 | 2 |
| 2011 | Heilongjiang | 186 | 126 | 213 | 12 | 8 | 13 | 123 | 87 | 128 | 8 | 5 | 8 | 34 | 21 | 45 | 2 | 1 | 3 | 30 | 18 | 40 | 2 | 1 | 3 |
| 2012 | Heilongjiang | 171 | 116 | 196 | 13 | 9 | 14 | 113 | 80 | 117 | 8 | 6 | 9 | 31 | 19 | 41 | 2 | 1 | 3 | 27 | 17 | 37 | 2 | 1 | 3 |
| 2013 | Heilongjiang | 156 | 106 | 179 | 12 | 8 | 14 | 103 | 73 | 107 | 8 | 6 | 8 | 28 | 17 | 38 | 2 | 1 | 3 | 25 | 16 | 34 | 2 | 1 | 3 |
| 2014 | Heilongjiang | 144 | 98 | 165 | 11 | 7 | 13 | 95 | 67 | 99 | 7 | 5 | 7 | 26 | 16 | 35 | 2 | 1 | 3 | 23 | 14 | 31 | 2 | 1 | 2 |
| 2015 | Heilongjiang | 129 | 88 | 148 | 10 | 7 | 11 | 85 | 60 | 89 | 7 | 5 | 7 | 23 | 14 | 31 | 2 | 1 | 2 | 21 | 13 | 28 | 2 | 1 | 2 |
| 2016 | Heilongjiang | 114 | 77 | 131 | 9 | 6 | 10 | 75 | 53 | 78 | 6 | 4 | 6 | 21 | 13 | 28 | 2 | 1 | 2 | 18 | 11 | 25 | 1 | 1 | 2 |
| 2017 | Heilongjiang | 101 | 68 | 115 | 9 | 6 | 10 | 67 | 47 | 69 | 6 | 4 | 6 | 18 | 11 | 24 | 2 | 1 | 2 | 16 | 10 | 22 | 1 | 1 | 2 |
| 2010 | Henan | 861 | 588 | 972 | 13 | 9 | 15 | 613 | 435 | 639 | 9 | 7 | 10 | 131 | 81 | 176 | 2 | 1 | 3 | 117 | 72 | 157 | 2 | 1 | 2 |
| 2011 | Henan | 807 | 550 | 912 | 11 | 8 | 13 | 567 | 402 | 591 | 8 | 6 | 8 | 127 | 78 | 170 | 2 | 1 | 2 | 113 | 70 | 151 | 2 | 1 | 2 |
| 2012 | Henan | 748 | 510 | 848 | 10 | 7 | 11 | 522 | 370 | 544 | 7 | 5 | 7 | 120 | 74 | 161 | 2 | 1 | 2 | 107 | 66 | 143 | 1 | 1 | 2 |
| 2013 | Henan | 692 | 471 | 785 | 9 | 6 | 11 | 477 | 338 | 497 | 6 | 5 | 7 | 113 | 70 | 152 | 2 | 1 | 2 | 101 | 63 | 136 | 1 | 1 | 2 |
| 2014 | Henan | 651 | 443 | 740 | 9 | 6 | 10 | 447 | 317 | 466 | 6 | 4 | 6 | 108 | 67 | 145 | 1 | 1 | 2 | 96 | 60 | 129 | 1 | 1 | 2 |
| 2015 | Henan | 597 | 406 | 678 | 8 | 6 | 9 | 409 | 290 | 427 | 6 | 4 | 6 | 99 | 61 | 133 | 1 | 1 | 2 | 88 | 55 | 118 | 1 | 1 | 2 |
| 2016 | Henan | 527 | 359 | 598 | 7 | 5 | 8 | 362 | 257 | 377 | 5 | 3 | 5 | 87 | 54 | 117 | 1 | 1 | 2 | 78 | 48 | 104 | 1 | 1 | 1 |
| 2017 | Henan | 461 | 314 | 523 | 6 | 4 | 7 | 318 | 226 | 332 | 4 | 3 | 4 | 76 | 47 | 101 | 1 | 1 | 1 | 67 | 42 | 90 | 1 | 1 | 1 |
| 2010 | Hubei | 319 | 222 | 349 | 9 | 6 | 10 | 267 | 189 | 278 | 8 | 5 | 8 | 28 | 17 | 37 | 1 | 0 | 1 | 25 | 15 | 33 | 1 | 0 | 1 |
| 2011 | Hubei | 299 | 207 | 326 | 10 | 7 | 11 | 248 | 176 | 258 | 8 | 6 | 8 | 27 | 17 | 36 | 1 | 1 | 1 | 24 | 15 | 32 | 1 | 0 | 1 |
| 2012 | Hubei | 273 | 190 | 299 | 9 | 6 | 10 | 225 | 160 | 235 | 8 | 5 | 8 | 25 | 16 | 34 | 1 | 1 | 1 | 23 | 14 | 30 | 1 | 0 | 1 |
| 2013 | Hubei | 248 | 172 | 272 | 8 | 6 | 9 | 203 | 144 | 211 | 7 | 5 | 7 | 24 | 15 | 32 | 1 | 0 | 1 | 21 | 13 | 28 | 1 | 0 | 1 |
| 2014 | Hubei | 228 | 158 | 251 | 7 | 5 | 8 | 186 | 132 | 194 | 6 | 4 | 6 | 22 | 14 | 30 | 1 | 0 | 1 | 20 | 12 | 26 | 1 | 0 | 1 |
| 2015 | Hubei | 205 | 142 | 225 | 7 | 5 | 7 | 167 | 118 | 174 | 5 | 4 | 6 | 20 | 12 | 27 | 1 | 0 | 1 | 18 | 11 | 24 | 1 | 0 | 1 |
| 2016 | Hubei | 178 | 123 | 196 | 6 | 4 | 6 | 145 | 103 | 151 | 5 | 3 | 5 | 18 | 11 | 24 | 1 | 0 | 1 | 16 | 10 | 21 | 1 | 0 | 1 |
| 2017 | Hubei | 154 | 107 | 169 | 5 | 3 | 5 | 125 | 89 | 131 | 4 | 3 | 4 | 15 | 9 | 21 | 0 | 0 | 1 | 14 | 8 | 18 | 0 | 0 | 1 |
| 2010 | Hunan | 331 | 229 | 362 | 8 | 6 | 9 | 272 | 193 | 283 | 7 | 5 | 7 | 31 | 19 | 42 | 1 | 0 | 1 | 28 | 17 | 37 | 1 | 0 | 1 |
| 2011 | Hunan | 306 | 212 | 336 | 7 | 5 | 8 | 250 | 178 | 261 | 6 | 4 | 6 | 29 | 18 | 39 | 1 | 0 | 1 | 26 | 16 | 35 | 1 | 0 | 1 |
| 2012 | Hunan | 278 | 193 | 305 | 7 | 5 | 7 | 227 | 161 | 237 | 5 | 4 | 6 | 27 | 17 | 36 | 1 | 0 | 1 | 24 | 15 | 32 | 1 | 0 | 1 |
| 2013 | Hunan | 250 | 173 | 274 | 6 | 4 | 7 | 203 | 144 | 212 | 5 | 3 | 5 | 25 | 15 | 33 | 1 | 0 | 1 | 22 | 14 | 29 | 1 | 0 | 1 |
| 2014 | Hunan | 229 | 159 | 252 | 5 | 4 | 6 | 186 | 132 | 194 | 4 | 3 | 5 | 23 | 14 | 31 | 1 | 0 | 1 | 20 | 13 | 27 | 0 | 0 | 1 |
| 2015 | Hunan | 204 | 141 | 224 | 5 | 3 | 5 | 165 | 117 | 172 | 4 | 3 | 4 | 20 | 13 | 27 | 0 | 0 | 1 | 18 | 11 | 24 | 0 | 0 | 1 |
| 2016 | Hunan | 175 | 121 | 192 | 4 | 3 | 4 | 142 | 101 | 148 | 3 | 2 | 3 | 17 | 11 | 23 | 0 | 0 | 1 | 16 | 10 | 21 | 0 | 0 | 0 |
| 2017 | Hunan | 151 | 105 | 166 | 3 | 2 | 4 | 123 | 87 | 128 | 3 | 2 | 3 | 15 | 9 | 20 | 0 | 0 | 0 | 13 | 8 | 18 | 0 | 0 | 0 |
| 2010 | Inner Mongolia | 259 | 177 | 292 | 18 | 12 | 20 | 187 | 132 | 195 | 13 | 9 | 14 | 38 | 24 | 51 | 3 | 2 | 4 | 34 | 21 | 46 | 2 | 1 | 3 |
| 2011 | Inner Mongolia | 244 | 167 | 275 | 20 | 14 | 23 | 174 | 124 | 182 | 14 | 10 | 15 | 37 | 23 | 49 | 3 | 2 | 4 | 33 | 20 | 44 | 3 | 2 | 4 |
| 2012 | Inner Mongolia | 228 | 156 | 257 | 20 | 14 | 23 | 162 | 115 | 169 | 14 | 10 | 15 | 35 | 22 | 47 | 3 | 2 | 4 | 31 | 19 | 42 | 3 | 2 | 4 |
| 2013 | Inner Mongolia | 213 | 145 | 241 | 19 | 13 | 22 | 150 | 107 | 157 | 13 | 10 | 14 | 33 | 21 | 45 | 3 | 2 | 4 | 30 | 18 | 40 | 3 | 2 | 4 |
| 2014 | Inner Mongolia | 204 | 139 | 231 | 18 | 12 | 20 | 144 | 102 | 150 | 13 | 9 | 13 | 32 | 20 | 43 | 3 | 2 | 4 | 28 | 18 | 38 | 2 | 2 | 3 |
| 2015 | Inner Mongolia | 191 | 130 | 216 | 17 | 12 | 19 | 135 | 96 | 140 | 12 | 9 | 13 | 30 | 18 | 40 | 3 | 2 | 4 | 27 | 16 | 36 | 2 | 1 | 3 |
| 2016 | Inner Mongolia | 174 | 119 | 196 | 16 | 11 | 18 | 123 | 87 | 128 | 11 | 8 | 11 | 27 | 17 | 36 | 2 | 2 | 3 | 24 | 15 | 32 | 2 | 1 | 3 |
| 2017 | Inner Mongolia | 156 | 106 | 176 | 15 | 10 | 17 | 110 | 78 | 115 | 10 | 7 | 11 | 24 | 15 | 32 | 2 | 1 | 3 | 21 | 13 | 29 | 2 | 1 | 3 |
| 2010 | Jiangsu | 182 | 125 | 203 | 4 | 3 | 5 | 136 | 97 | 142 | 3 | 2 | 3 | 24 | 15 | 32 | 1 | 0 | 1 | 22 | 13 | 29 | 0 | 0 | 1 |
| 2011 | Jiangsu | 168 | 115 | 188 | 4 | 3 | 5 | 124 | 88 | 129 | 3 | 2 | 3 | 23 | 14 | 31 | 1 | 0 | 1 | 21 | 13 | 28 | 1 | 0 | 1 |
| 2012 | Jiangsu | 153 | 105 | 172 | 4 | 3 | 4 | 112 | 80 | 117 | 3 | 2 | 3 | 22 | 13 | 29 | 1 | 0 | 1 | 19 | 12 | 26 | 0 | 0 | 1 |
| 2013 | Jiangsu | 141 | 97 | 159 | 4 | 2 | 4 | 102 | 73 | 107 | 3 | 2 | 3 | 20 | 13 | 27 | 1 | 0 | 1 | 18 | 11 | 24 | 0 | 0 | 1 |
| 2014 | Jiangsu | 132 | 90 | 148 | 3 | 2 | 4 | 95 | 67 | 99 | 2 | 2 | 3 | 19 | 12 | 26 | 0 | 0 | 1 | 17 | 11 | 23 | 0 | 0 | 1 |
| 2015 | Jiangsu | 119 | 82 | 134 | 3 | 2 | 3 | 86 | 61 | 89 | 2 | 2 | 2 | 18 | 11 | 24 | 0 | 0 | 1 | 16 | 10 | 21 | 0 | 0 | 1 |
| 2016 | Jiangsu | 104 | 71 | 117 | 3 | 2 | 3 | 74 | 53 | 77 | 2 | 1 | 2 | 16 | 10 | 21 | 0 | 0 | 1 | 14 | 9 | 19 | 0 | 0 | 0 |
| 2017 | Jiangsu | 90 | 61 | 101 | 2 | 2 | 3 | 64 | 46 | 67 | 2 | 1 | 2 | 13 | 8 | 18 | 0 | 0 | 0 | 12 | 7 | 16 | 0 | 0 | 0 |
| 2010 | Jiangxi | 1074 | 743 | 1181 | 36 | 25 | 39 | 866 | 614 | 903 | 29 | 20 | 30 | 110 | 68 | 147 | 4 | 2 | 5 | 98 | 61 | 131 | 3 | 2 | 4 |
| 2011 | Jiangxi | 966 | 667 | 1064 | 30 | 21 | 33 | 773 | 548 | 806 | 24 | 17 | 25 | 102 | 63 | 137 | 3 | 2 | 4 | 91 | 56 | 122 | 3 | 2 | 4 |
| 2012 | Jiangxi | 866 | 598 | 955 | 25 | 17 | 28 | 690 | 489 | 719 | 20 | 14 | 21 | 93 | 57 | 124 | 3 | 2 | 4 | 83 | 51 | 111 | 2 | 1 | 3 |
| 2013 | Jiangxi | 779 | 538 | 860 | 23 | 16 | 25 | 617 | 437 | 643 | 18 | 13 | 19 | 86 | 53 | 115 | 3 | 2 | 3 | 76 | 47 | 102 | 2 | 1 | 3 |
| 2014 | Jiangxi | 719 | 496 | 795 | 21 | 15 | 24 | 567 | 402 | 591 | 17 | 12 | 18 | 81 | 50 | 108 | 2 | 1 | 3 | 72 | 44 | 96 | 2 | 1 | 3 |
| 2015 | Jiangxi | 650 | 449 | 719 | 20 | 14 | 22 | 512 | 363 | 533 | 15 | 11 | 16 | 73 | 45 | 99 | 2 | 1 | 3 | 65 | 40 | 88 | 2 | 1 | 3 |
| 2016 | Jiangxi | 581 | 400 | 642 | 18 | 12 | 20 | 456 | 324 | 476 | 14 | 10 | 14 | 66 | 41 | 88 | 2 | 1 | 3 | 59 | 36 | 79 | 2 | 1 | 2 |
| 2017 | Jiangxi | 514 | 354 | 568 | 16 | 11 | 18 | 405 | 287 | 422 | 13 | 9 | 14 | 57 | 36 | 77 | 2 | 1 | 2 | 51 | 32 | 69 | 2 | 1 | 2 |
| 2010 | Jilin | 215 | 147 | 245 | 15 | 10 | 17 | 148 | 105 | 154 | 10 | 7 | 11 | 36 | 22 | 48 | 2 | 2 | 3 | 32 | 20 | 43 | 2 | 1 | 3 |
| 2011 | Jilin | 200 | 136 | 228 | 17 | 11 | 19 | 135 | 96 | 141 | 11 | 8 | 12 | 34 | 21 | 46 | 3 | 2 | 4 | 30 | 19 | 41 | 3 | 2 | 3 |
| 2012 | Jilin | 184 | 125 | 210 | 17 | 12 | 20 | 123 | 87 | 128 | 12 | 8 | 12 | 32 | 20 | 43 | 3 | 2 | 4 | 29 | 18 | 38 | 3 | 2 | 4 |
| 2013 | Jilin | 171 | 116 | 195 | 16 | 11 | 19 | 113 | 80 | 118 | 11 | 8 | 11 | 30 | 19 | 41 | 3 | 2 | 4 | 27 | 17 | 36 | 3 | 2 | 3 |
| 2014 | Jilin | 162 | 110 | 185 | 16 | 11 | 18 | 107 | 76 | 112 | 10 | 7 | 11 | 29 | 18 | 39 | 3 | 2 | 4 | 26 | 16 | 35 | 2 | 2 | 3 |
| 2015 | Jilin | 149 | 101 | 171 | 15 | 10 | 17 | 98 | 70 | 103 | 10 | 7 | 10 | 27 | 17 | 36 | 3 | 2 | 4 | 24 | 15 | 32 | 2 | 1 | 3 |
| 2016 | Jilin | 132 | 89 | 151 | 13 | 9 | 15 | 87 | 62 | 90 | 9 | 6 | 9 | 24 | 15 | 32 | 2 | 1 | 3 | 21 | 13 | 28 | 2 | 1 | 3 |
| 2017 | Jilin | 116 | 79 | 133 | 12 | 8 | 14 | 77 | 54 | 80 | 8 | 6 | 8 | 21 | 13 | 28 | 2 | 1 | 3 | 19 | 11 | 25 | 2 | 1 | 3 |
| 2010 | Liaoning | 86 | 58 | 97 | 4 | 3 | 5 | 60 | 43 | 63 | 3 | 2 | 3 | 14 | 8 | 18 | 1 | 0 | 1 | 12 | 7 | 16 | 1 | 0 | 1 |
| 2011 | Liaoning | 79 | 54 | 90 | 4 | 3 | 5 | 55 | 39 | 57 | 3 | 2 | 3 | 13 | 8 | 17 | 1 | 0 | 1 | 11 | 7 | 15 | 1 | 0 | 1 |
| 2012 | Liaoning | 73 | 50 | 82 | 5 | 3 | 5 | 50 | 36 | 52 | 3 | 2 | 3 | 12 | 7 | 16 | 1 | 0 | 1 | 11 | 7 | 14 | 1 | 0 | 1 |
| 2013 | Liaoning | 68 | 46 | 77 | 4 | 3 | 5 | 47 | 33 | 49 | 3 | 2 | 3 | 11 | 7 | 15 | 1 | 0 | 1 | 10 | 6 | 13 | 1 | 0 | 1 |
| 2014 | Liaoning | 64 | 44 | 73 | 4 | 3 | 5 | 44 | 31 | 46 | 3 | 2 | 3 | 11 | 7 | 14 | 1 | 0 | 1 | 9 | 6 | 13 | 1 | 0 | 1 |
| 2015 | Liaoning | 59 | 40 | 67 | 4 | 3 | 4 | 41 | 29 | 43 | 3 | 2 | 3 | 10 | 6 | 13 | 1 | 0 | 1 | 9 | 5 | 12 | 1 | 0 | 1 |
| 2016 | Liaoning | 53 | 36 | 60 | 3 | 2 | 4 | 36 | 26 | 38 | 2 | 2 | 3 | 9 | 5 | 12 | 1 | 0 | 1 | 8 | 5 | 10 | 1 | 0 | 1 |
| 2017 | Liaoning | 47 | 32 | 53 | 3 | 2 | 4 | 32 | 23 | 34 | 2 | 2 | 2 | 8 | 5 | 10 | 1 | 0 | 1 | 7 | 4 | 9 | 0 | 0 | 1 |
| 2010 | Ningxia | 159 | 110 | 174 | 36 | 25 | 39 | 130 | 92 | 135 | 29 | 21 | 31 | 15 | 9 | 21 | 3 | 2 | 5 | 14 | 8 | 18 | 3 | 2 | 4 |
| 2011 | Ningxia | 142 | 98 | 156 | 32 | 22 | 36 | 116 | 82 | 121 | 27 | 19 | 28 | 14 | 8 | 18 | 3 | 2 | 4 | 12 | 8 | 16 | 3 | 2 | 4 |
| 2012 | Ningxia | 128 | 89 | 141 | 29 | 20 | 32 | 104 | 74 | 108 | 24 | 17 | 25 | 13 | 8 | 17 | 3 | 2 | 4 | 11 | 7 | 15 | 3 | 2 | 4 |
| 2013 | Ningxia | 115 | 80 | 127 | 26 | 18 | 29 | 93 | 66 | 97 | 21 | 15 | 22 | 12 | 7 | 16 | 3 | 2 | 4 | 11 | 7 | 14 | 2 | 2 | 3 |
| 2014 | Ningxia | 107 | 74 | 118 | 23 | 16 | 25 | 86 | 61 | 90 | 18 | 13 | 19 | 11 | 7 | 15 | 2 | 1 | 3 | 10 | 6 | 13 | 2 | 1 | 3 |
| 2015 | Ningxia | 98 | 68 | 108 | 22 | 15 | 24 | 79 | 56 | 82 | 17 | 12 | 18 | 10 | 6 | 14 | 2 | 1 | 3 | 9 | 6 | 12 | 2 | 1 | 3 |
| 2016 | Ningxia | 89 | 61 | 98 | 20 | 14 | 22 | 71 | 50 | 74 | 16 | 11 | 16 | 9 | 6 | 13 | 2 | 1 | 3 | 8 | 5 | 11 | 2 | 1 | 2 |
| 2017 | Ningxia | 79 | 54 | 87 | 17 | 12 | 19 | 63 | 45 | 66 | 14 | 10 | 15 | 8 | 5 | 11 | 2 | 1 | 2 | 7 | 5 | 10 | 2 | 1 | 2 |
| 2010 | Qinghai | 268 | 180 | 298 | 73 | 49 | 82 | 224 | 159 | 233 | 61 | 44 | 64 | 23 | 11 | 34 | 6 | 3 | 9 | 21 | 10 | 30 | 6 | 3 | 8 |
| 2011 | Qinghai | 246 | 175 | 279 | 68 | 48 | 77 | 203 | 144 | 211 | 56 | 40 | 58 | 23 | 16 | 36 | 6 | 5 | 10 | 20 | 15 | 32 | 6 | 4 | 9 |
| 2012 | Qinghai | 226 | 159 | 254 | 63 | 44 | 71 | 184 | 130 | 191 | 51 | 36 | 53 | 22 | 15 | 33 | 6 | 4 | 9 | 20 | 14 | 30 | 6 | 4 | 8 |
| 2013 | Qinghai | 208 | 145 | 233 | 59 | 41 | 66 | 167 | 118 | 174 | 48 | 34 | 50 | 22 | 14 | 31 | 6 | 4 | 9 | 20 | 13 | 28 | 6 | 4 | 8 |
| 2014 | Qinghai | 196 | 135 | 216 | 53 | 36 | 58 | 155 | 110 | 162 | 42 | 30 | 43 | 22 | 13 | 29 | 6 | 4 | 8 | 19 | 12 | 26 | 5 | 3 | 7 |
| 2015 | Qinghai | 177 | 122 | 196 | 48 | 33 | 53 | 141 | 100 | 147 | 38 | 27 | 40 | 19 | 12 | 25 | 5 | 3 | 7 | 17 | 10 | 23 | 5 | 3 | 6 |
| 2016 | Qinghai | 155 | 107 | 171 | 41 | 29 | 46 | 123 | 87 | 128 | 33 | 23 | 34 | 17 | 11 | 23 | 5 | 3 | 6 | 15 | 9 | 20 | 4 | 3 | 5 |
| 2017 | Qinghai | 135 | 93 | 149 | 36 | 25 | 40 | 107 | 76 | 112 | 28 | 20 | 30 | 15 | 9 | 20 | 4 | 2 | 5 | 13 | 8 | 18 | 3 | 2 | 5 |
| 2010 | Shaanxi | 618 | 426 | 685 | 27 | 19 | 30 | 480 | 340 | 500 | 21 | 15 | 22 | 73 | 45 | 98 | 3 | 2 | 4 | 65 | 40 | 87 | 3 | 2 | 4 |
| 2011 | Shaanxi | 563 | 387 | 626 | 27 | 19 | 30 | 433 | 307 | 451 | 21 | 15 | 22 | 69 | 43 | 93 | 3 | 2 | 5 | 61 | 38 | 82 | 3 | 2 | 4 |
| 2012 | Shaanxi | 514 | 354 | 573 | 26 | 18 | 29 | 391 | 278 | 408 | 20 | 14 | 21 | 65 | 40 | 87 | 3 | 2 | 4 | 58 | 36 | 78 | 3 | 2 | 4 |
| 2013 | Shaanxi | 470 | 323 | 525 | 24 | 16 | 26 | 354 | 251 | 369 | 18 | 13 | 19 | 61 | 38 | 82 | 3 | 2 | 4 | 55 | 34 | 73 | 3 | 2 | 4 |
| 2014 | Shaanxi | 442 | 304 | 495 | 21 | 15 | 24 | 331 | 235 | 345 | 16 | 11 | 17 | 59 | 37 | 79 | 3 | 2 | 4 | 53 | 33 | 71 | 3 | 2 | 3 |
| 2015 | Shaanxi | 410 | 282 | 459 | 20 | 14 | 22 | 306 | 217 | 318 | 15 | 11 | 16 | 55 | 34 | 74 | 3 | 2 | 4 | 49 | 31 | 66 | 2 | 1 | 3 |
| 2016 | Shaanxi | 370 | 254 | 414 | 18 | 13 | 20 | 275 | 195 | 287 | 14 | 10 | 14 | 50 | 31 | 67 | 2 | 2 | 3 | 45 | 28 | 60 | 2 | 1 | 3 |
| 2017 | Shaanxi | 328 | 225 | 366 | 16 | 11 | 18 | 244 | 173 | 254 | 12 | 9 | 13 | 44 | 27 | 59 | 2 | 1 | 3 | 39 | 24 | 53 | 2 | 1 | 3 |
| 2010 | Shandong | 286 | 194 | 328 | 5 | 3 | 5 | 186 | 132 | 194 | 3 | 2 | 3 | 53 | 33 | 71 | 1 | 1 | 1 | 47 | 29 | 63 | 1 | 0 | 1 |
| 2011 | Shandong | 272 | 184 | 312 | 5 | 3 | 6 | 175 | 124 | 183 | 3 | 2 | 3 | 51 | 32 | 69 | 1 | 1 | 1 | 45 | 28 | 61 | 1 | 0 | 1 |
| 2012 | Shandong | 254 | 172 | 292 | 5 | 3 | 5 | 163 | 115 | 170 | 3 | 2 | 3 | 48 | 30 | 65 | 1 | 1 | 1 | 43 | 27 | 58 | 1 | 0 | 1 |
| 2013 | Shandong | 237 | 160 | 273 | 4 | 3 | 5 | 151 | 107 | 157 | 3 | 2 | 3 | 46 | 28 | 61 | 1 | 1 | 1 | 41 | 25 | 55 | 1 | 0 | 1 |
| 2014 | Shandong | 225 | 152 | 259 | 4 | 3 | 5 | 143 | 101 | 149 | 3 | 2 | 3 | 43 | 27 | 58 | 1 | 0 | 1 | 39 | 24 | 52 | 1 | 0 | 1 |
| 2015 | Shandong | 206 | 139 | 238 | 4 | 2 | 4 | 131 | 93 | 136 | 2 | 2 | 2 | 40 | 25 | 54 | 1 | 0 | 1 | 36 | 22 | 48 | 1 | 0 | 1 |
| 2016 | Shandong | 181 | 122 | 208 | 3 | 2 | 4 | 115 | 81 | 119 | 2 | 1 | 2 | 35 | 22 | 47 | 1 | 0 | 1 | 31 | 19 | 42 | 1 | 0 | 1 |
| 2017 | Shandong | 158 | 107 | 181 | 3 | 2 | 3 | 100 | 71 | 105 | 2 | 1 | 2 | 30 | 19 | 41 | 0 | 0 | 1 | 27 | 17 | 36 | 0 | 0 | 1 |
| 2010 | Shanghai | 52 | 36 | 58 | 6 | 4 | 7 | 42 | 30 | 44 | 5 | 3 | 5 | 5 | 3 | 7 | 1 | 0 | 1 | 5 | 3 | 6 | 1 | 0 | 1 |
| 2011 | Shanghai | 54 | 37 | 60 | 6 | 4 | 7 | 44 | 31 | 45 | 5 | 4 | 5 | 6 | 3 | 8 | 1 | 0 | 1 | 5 | 3 | 7 | 1 | 0 | 1 |
| 2012 | Shanghai | 54 | 38 | 60 | 6 | 4 | 7 | 44 | 31 | 45 | 5 | 3 | 5 | 6 | 4 | 8 | 1 | 0 | 1 | 5 | 3 | 7 | 1 | 0 | 1 |
| 2013 | Shanghai | 56 | 39 | 62 | 6 | 4 | 7 | 44 | 31 | 46 | 5 | 3 | 5 | 6 | 4 | 8 | 1 | 0 | 1 | 5 | 3 | 7 | 1 | 0 | 1 |
| 2014 | Shanghai | 57 | 39 | 63 | 6 | 4 | 7 | 45 | 32 | 47 | 5 | 3 | 5 | 6 | 4 | 8 | 1 | 0 | 1 | 6 | 3 | 8 | 1 | 0 | 1 |
| 2015 | Shanghai | 56 | 39 | 62 | 6 | 4 | 7 | 44 | 31 | 46 | 5 | 3 | 5 | 6 | 4 | 8 | 1 | 0 | 1 | 6 | 3 | 8 | 1 | 0 | 1 |
| 2016 | Shanghai | 55 | 38 | 61 | 6 | 4 | 6 | 43 | 31 | 45 | 5 | 3 | 5 | 6 | 4 | 8 | 1 | 0 | 1 | 6 | 3 | 7 | 1 | 0 | 1 |
| 2017 | Shanghai | 50 | 34 | 55 | 5 | 4 | 6 | 39 | 28 | 41 | 4 | 3 | 4 | 6 | 3 | 7 | 1 | 0 | 1 | 5 | 3 | 7 | 1 | 0 | 1 |
| 2010 | Shanxi | 420 | 288 | 471 | 20 | 14 | 22 | 312 | 221 | 325 | 15 | 10 | 15 | 57 | 36 | 77 | 3 | 2 | 4 | 51 | 32 | 69 | 2 | 1 | 3 |
| 2011 | Shanxi | 397 | 272 | 445 | 20 | 14 | 22 | 293 | 207 | 305 | 15 | 10 | 15 | 55 | 34 | 74 | 3 | 2 | 4 | 49 | 31 | 66 | 2 | 2 | 3 |
| 2012 | Shanxi | 374 | 256 | 419 | 20 | 14 | 23 | 274 | 194 | 286 | 15 | 11 | 15 | 53 | 33 | 71 | 3 | 2 | 4 | 47 | 29 | 63 | 3 | 2 | 3 |
| 2013 | Shanxi | 352 | 241 | 395 | 19 | 13 | 21 | 257 | 182 | 268 | 14 | 10 | 14 | 50 | 31 | 67 | 3 | 2 | 4 | 45 | 28 | 60 | 2 | 1 | 3 |
| 2014 | Shanxi | 341 | 233 | 383 | 18 | 12 | 20 | 249 | 176 | 259 | 13 | 9 | 13 | 49 | 30 | 65 | 3 | 2 | 3 | 43 | 27 | 58 | 2 | 1 | 3 |
| 2015 | Shanxi | 324 | 222 | 364 | 17 | 12 | 19 | 237 | 168 | 247 | 12 | 9 | 13 | 46 | 29 | 62 | 2 | 1 | 3 | 41 | 26 | 55 | 2 | 1 | 3 |
| 2016 | Shanxi | 299 | 204 | 335 | 16 | 11 | 18 | 218 | 155 | 227 | 11 | 8 | 12 | 43 | 26 | 57 | 2 | 1 | 3 | 38 | 23 | 51 | 2 | 1 | 3 |
| 2017 | Shanxi | 269 | 185 | 302 | 15 | 10 | 16 | 198 | 140 | 206 | 11 | 8 | 11 | 38 | 24 | 51 | 2 | 1 | 3 | 34 | 21 | 45 | 2 | 1 | 2 |
| 2010 | Sichuan | 1608 | 1120 | 1742 | 35 | 24 | 38 | 1387 | 984 | 1446 | 30 | 21 | 31 | 117 | 72 | 157 | 3 | 2 | 3 | 104 | 64 | 140 | 2 | 1 | 3 |
| 2011 | Sichuan | 1411 | 983 | 1531 | 33 | 23 | 36 | 1210 | 858 | 1261 | 28 | 20 | 30 | 107 | 66 | 143 | 3 | 2 | 3 | 95 | 59 | 127 | 2 | 1 | 3 |
| 2012 | Sichuan | 1227 | 854 | 1333 | 29 | 20 | 32 | 1047 | 742 | 1091 | 25 | 18 | 26 | 96 | 59 | 128 | 2 | 1 | 3 | 85 | 53 | 114 | 2 | 1 | 3 |
| 2013 | Sichuan | 1070 | 744 | 1164 | 26 | 18 | 28 | 907 | 643 | 945 | 22 | 16 | 23 | 86 | 53 | 116 | 2 | 1 | 3 | 77 | 48 | 103 | 2 | 1 | 2 |
| 2014 | Sichuan | 955 | 664 | 1040 | 23 | 16 | 25 | 806 | 572 | 840 | 20 | 14 | 20 | 79 | 49 | 106 | 2 | 1 | 3 | 70 | 43 | 94 | 2 | 1 | 2 |
| 2015 | Sichuan | 839 | 583 | 914 | 20 | 14 | 22 | 707 | 501 | 737 | 17 | 12 | 18 | 70 | 43 | 94 | 2 | 1 | 2 | 62 | 39 | 84 | 2 | 1 | 2 |
| 2016 | Sichuan | 719 | 499 | 783 | 17 | 12 | 19 | 605 | 429 | 630 | 15 | 10 | 15 | 60 | 37 | 81 | 1 | 1 | 2 | 54 | 33 | 72 | 1 | 1 | 2 |
| 2017 | Sichuan | 610 | 424 | 665 | 15 | 10 | 16 | 515 | 365 | 536 | 12 | 9 | 13 | 51 | 31 | 68 | 1 | 1 | 2 | 45 | 28 | 60 | 1 | 1 | 1 |
| 2010 | Tianjin | 50 | 35 | 56 | 8 | 5 | 9 | 40 | 28 | 41 | 6 | 4 | 7 | 6 | 3 | 8 | 1 | 1 | 1 | 5 | 3 | 7 | 1 | 0 | 1 |
| 2011 | Tianjin | 51 | 35 | 56 | 10 | 7 | 11 | 40 | 28 | 41 | 8 | 6 | 8 | 6 | 4 | 8 | 1 | 1 | 2 | 5 | 3 | 7 | 1 | 1 | 1 |
| 2012 | Tianjin | 51 | 35 | 56 | 10 | 7 | 11 | 40 | 28 | 41 | 8 | 5 | 8 | 6 | 4 | 8 | 1 | 1 | 1 | 5 | 3 | 7 | 1 | 1 | 1 |
| 2013 | Tianjin | 51 | 35 | 57 | 10 | 7 | 11 | 40 | 29 | 42 | 7 | 5 | 8 | 6 | 4 | 8 | 1 | 1 | 1 | 5 | 3 | 7 | 1 | 1 | 1 |
| 2014 | Tianjin | 53 | 37 | 59 | 9 | 6 | 10 | 42 | 30 | 43 | 7 | 5 | 8 | 6 | 4 | 8 | 1 | 1 | 1 | 5 | 3 | 7 | 1 | 1 | 1 |
| 2015 | Tianjin | 53 | 37 | 59 | 10 | 7 | 11 | 42 | 30 | 44 | 8 | 5 | 8 | 6 | 4 | 8 | 1 | 1 | 1 | 5 | 3 | 7 | 1 | 1 | 1 |
| 2016 | Tianjin | 52 | 36 | 57 | 9 | 6 | 10 | 41 | 29 | 42 | 7 | 5 | 7 | 6 | 4 | 8 | 1 | 1 | 1 | 5 | 3 | 7 | 1 | 1 | 1 |
| 2017 | Tianjin | 48 | 33 | 53 | 8 | 6 | 9 | 38 | 27 | 40 | 7 | 5 | 7 | 5 | 3 | 7 | 1 | 1 | 1 | 5 | 3 | 6 | 1 | 1 | 1 |
| 2010 | Tibet | 224 | 154 | 248 | 116 | 80 | 128 | 199 | 141 | 207 | 103 | 73 | 107 | 13 | 7 | 22 | 7 | 4 | 11 | 12 | 6 | 19 | 6 | 3 | 10 |
| 2011 | Tibet | 218 | 151 | 242 | 114 | 79 | 126 | 193 | 137 | 201 | 101 | 71 | 105 | 13 | 7 | 21 | 7 | 4 | 11 | 12 | 7 | 19 | 6 | 3 | 10 |
| 2012 | Tibet | 211 | 146 | 234 | 90 | 62 | 100 | 187 | 132 | 194 | 80 | 56 | 83 | 13 | 7 | 21 | 6 | 3 | 9 | 12 | 6 | 18 | 5 | 3 | 8 |
| 2013 | Tibet | 203 | 140 | 225 | 100 | 69 | 110 | 179 | 127 | 186 | 88 | 62 | 92 | 13 | 7 | 20 | 6 | 3 | 10 | 11 | 6 | 18 | 6 | 3 | 9 |
| 2014 | Tibet | 200 | 138 | 221 | 81 | 56 | 90 | 177 | 126 | 185 | 72 | 51 | 75 | 12 | 7 | 19 | 5 | 3 | 8 | 11 | 6 | 17 | 4 | 2 | 7 |
| 2015 | Tibet | 191 | 132 | 211 | 77 | 53 | 85 | 169 | 120 | 176 | 68 | 48 | 71 | 12 | 6 | 18 | 5 | 3 | 7 | 10 | 6 | 16 | 4 | 2 | 7 |
| 2016 | Tibet | 174 | 120 | 193 | 67 | 46 | 74 | 155 | 110 | 161 | 60 | 42 | 62 | 10 | 6 | 17 | 4 | 2 | 6 | 9 | 5 | 15 | 4 | 2 | 6 |
| 2017 | Tibet | 157 | 108 | 173 | 57 | 40 | 64 | 139 | 99 | 145 | 51 | 36 | 53 | 9 | 5 | 15 | 3 | 2 | 5 | 8 | 4 | 13 | 3 | 2 | 5 |
| 2010 | Xinjiang | 1274 | 876 | 1421 | 75 | 51 | 83 | 1118 | 793 | 1165 | 66 | 46 | 68 | 83 | 44 | 135 | 5 | 3 | 8 | 73 | 39 | 121 | 4 | 2 | 7 |
| 2011 | Xinjiang | 1209 | 834 | 1347 | 71 | 49 | 80 | 1058 | 750 | 1103 | 63 | 44 | 65 | 80 | 44 | 129 | 5 | 3 | 8 | 71 | 39 | 115 | 4 | 2 | 7 |
| 2012 | Xinjiang | 1139 | 784 | 1265 | 63 | 44 | 70 | 996 | 706 | 1038 | 55 | 39 | 58 | 76 | 41 | 120 | 4 | 2 | 7 | 67 | 37 | 107 | 4 | 2 | 6 |
| 2013 | Xinjiang | 1065 | 732 | 1182 | 58 | 40 | 65 | 930 | 659 | 969 | 51 | 36 | 53 | 71 | 38 | 113 | 4 | 2 | 6 | 64 | 34 | 100 | 3 | 2 | 5 |
| 2014 | Xinjiang | 1024 | 703 | 1134 | 55 | 38 | 61 | 894 | 634 | 932 | 48 | 34 | 50 | 69 | 36 | 107 | 4 | 2 | 6 | 61 | 32 | 95 | 3 | 2 | 5 |
| 2015 | Xinjiang | 957 | 657 | 1059 | 51 | 35 | 56 | 834 | 591 | 869 | 44 | 31 | 46 | 65 | 35 | 101 | 3 | 2 | 5 | 58 | 31 | 90 | 3 | 2 | 5 |
| 2016 | Xinjiang | 870 | 595 | 963 | 45 | 31 | 50 | 757 | 537 | 789 | 39 | 28 | 41 | 60 | 31 | 92 | 3 | 2 | 5 | 54 | 28 | 82 | 3 | 1 | 4 |
| 2017 | Xinjiang | 782 | 560 | 879 | 38 | 28 | 43 | 676 | 479 | 704 | 33 | 24 | 35 | 56 | 43 | 93 | 3 | 2 | 5 | 50 | 38 | 83 | 2 | 2 | 4 |
| 2010 | Yunnan | 1751 | 1222 | 1888 | 58 | 41 | 63 | 1538 | 1091 | 1603 | 51 | 36 | 53 | 113 | 70 | 151 | 4 | 2 | 5 | 100 | 62 | 135 | 3 | 2 | 4 |
| 2011 | Yunnan | 1591 | 1110 | 1719 | 55 | 38 | 59 | 1387 | 983 | 1445 | 48 | 34 | 50 | 108 | 67 | 145 | 4 | 2 | 5 | 96 | 59 | 129 | 3 | 2 | 4 |
| 2012 | Yunnan | 1425 | 993 | 1542 | 49 | 34 | 53 | 1235 | 876 | 1287 | 43 | 30 | 45 | 101 | 62 | 135 | 3 | 2 | 5 | 90 | 56 | 120 | 3 | 2 | 4 |
| 2013 | Yunnan | 1277 | 890 | 1384 | 44 | 31 | 48 | 1099 | 779 | 1145 | 38 | 27 | 40 | 94 | 58 | 126 | 3 | 2 | 4 | 84 | 52 | 113 | 3 | 2 | 4 |
| 2014 | Yunnan | 1177 | 820 | 1277 | 41 | 29 | 44 | 1009 | 715 | 1051 | 35 | 25 | 37 | 89 | 55 | 120 | 3 | 2 | 4 | 79 | 49 | 106 | 3 | 2 | 4 |
| 2015 | Yunnan | 1058 | 736 | 1149 | 37 | 26 | 40 | 905 | 642 | 943 | 32 | 23 | 33 | 81 | 50 | 109 | 3 | 2 | 4 | 72 | 45 | 97 | 3 | 2 | 3 |
| 2016 | Yunnan | 938 | 653 | 1019 | 33 | 23 | 36 | 803 | 569 | 837 | 28 | 20 | 29 | 72 | 44 | 96 | 3 | 2 | 3 | 64 | 39 | 86 | 2 | 1 | 3 |
| 2017 | Yunnan | 821 | 571 | 890 | 29 | 20 | 31 | 704 | 499 | 734 | 25 | 18 | 26 | 61 | 38 | 83 | 2 | 1 | 3 | 55 | 34 | 73 | 2 | 1 | 3 |
| 2010 | Zhejiang | 242 | 166 | 270 | 8 | 5 | 9 | 181 | 128 | 189 | 6 | 4 | 6 | 32 | 20 | 43 | 1 | 1 | 1 | 29 | 18 | 38 | 1 | 1 | 1 |
| 2011 | Zhejiang | 226 | 155 | 253 | 9 | 6 | 10 | 168 | 119 | 176 | 7 | 5 | 7 | 30 | 19 | 41 | 1 | 1 | 2 | 27 | 17 | 36 | 1 | 1 | 1 |
| 2012 | Zhejiang | 207 | 142 | 232 | 8 | 5 | 9 | 153 | 108 | 159 | 6 | 4 | 6 | 29 | 18 | 38 | 1 | 1 | 1 | 25 | 16 | 34 | 1 | 1 | 1 |
| 2013 | Zhejiang | 193 | 132 | 217 | 7 | 5 | 8 | 141 | 100 | 147 | 5 | 4 | 6 | 27 | 17 | 37 | 1 | 1 | 1 | 24 | 15 | 33 | 1 | 1 | 1 |
| 2014 | Zhejiang | 183 | 125 | 205 | 7 | 5 | 8 | 133 | 95 | 139 | 5 | 4 | 5 | 26 | 16 | 35 | 1 | 1 | 1 | 23 | 14 | 31 | 1 | 1 | 1 |
| 2015 | Zhejiang | 167 | 114 | 188 | 6 | 4 | 7 | 122 | 86 | 127 | 5 | 3 | 5 | 24 | 15 | 32 | 1 | 1 | 1 | 21 | 13 | 29 | 1 | 1 | 1 |
| 2016 | Zhejiang | 147 | 100 | 165 | 5 | 4 | 6 | 106 | 75 | 111 | 4 | 3 | 4 | 21 | 13 | 29 | 1 | 0 | 1 | 19 | 12 | 25 | 1 | 0 | 1 |
| 2017 | Zhejiang | 127 | 87 | 142 | 5 | 3 | 5 | 92 | 65 | 96 | 3 | 2 | 4 | 18 | 11 | 24 | 1 | 0 | 1 | 16 | 10 | 22 | 1 | 0 | 1 |
| 2010 | Central | 5174 | 3550 | 5784 | 16 | 11 | 18 | 3863 | 2739 | 4026 | 12 | 8 | 12 | 693 | 429 | 930 | 2 | 1 | 3 | 617 | 382 | 828 | 2 | 1 | 3 |
| 2011 | Central | 4795 | 3288 | 5371 | 15 | 10 | 17 | 3549 | 2516 | 3698 | 11 | 8 | 12 | 660 | 408 | 885 | 2 | 1 | 3 | 587 | 363 | 788 | 2 | 1 | 2 |
| 2012 | Central | 4405 | 3019 | 4939 | 14 | 10 | 16 | 3242 | 2299 | 3379 | 10 | 7 | 11 | 615 | 381 | 825 | 2 | 1 | 3 | 548 | 339 | 735 | 2 | 1 | 2 |
| 2013 | Central | 4040 | 2767 | 4536 | 13 | 9 | 14 | 2954 | 2095 | 3078 | 9 | 7 | 10 | 575 | 356 | 771 | 2 | 1 | 2 | 512 | 317 | 687 | 2 | 1 | 2 |
| 2014 | Central | 3793 | 2597 | 4260 | 12 | 8 | 13 | 2767 | 1962 | 2883 | 9 | 6 | 9 | 543 | 336 | 728 | 2 | 1 | 2 | 483 | 299 | 649 | 2 | 1 | 2 |
| 2015 | Central | 3485 | 2386 | 3915 | 11 | 8 | 12 | 2540 | 1801 | 2647 | 8 | 6 | 8 | 500 | 309 | 671 | 2 | 1 | 2 | 445 | 275 | 597 | 1 | 1 | 2 |
| 2016 | Central | 3115 | 2133 | 3500 | 10 | 7 | 11 | 2271 | 1610 | 2367 | 7 | 5 | 7 | 447 | 276 | 599 | 1 | 1 | 2 | 398 | 246 | 534 | 1 | 1 | 2 |
| 2017 | Central | 2753 | 1886 | 3091 | 9 | 6 | 10 | 2013 | 1427 | 2098 | 6 | 4 | 7 | 392 | 242 | 526 | 1 | 1 | 2 | 349 | 216 | 468 | 1 | 1 | 1 |
| 2010 | East | 2230 | 1535 | 2478 | 9 | 6 | 9 | 1715 | 1216 | 1787 | 7 | 5 | 7 | 272 | 169 | 366 | 1 | 1 | 1 | 243 | 150 | 325 | 1 | 1 | 1 |
| 2011 | East | 2075 | 1427 | 2311 | 9 | 6 | 10 | 1579 | 1120 | 1646 | 7 | 5 | 7 | 262 | 162 | 352 | 1 | 1 | 2 | 234 | 145 | 313 | 1 | 1 | 1 |
| 2012 | East | 1922 | 1321 | 2144 | 8 | 5 | 9 | 1452 | 1030 | 1513 | 6 | 4 | 6 | 249 | 154 | 334 | 1 | 1 | 1 | 221 | 137 | 297 | 1 | 1 | 1 |
| 2013 | East | 1805 | 1239 | 2016 | 7 | 5 | 8 | 1353 | 959 | 1410 | 5 | 4 | 6 | 239 | 148 | 321 | 1 | 1 | 1 | 213 | 132 | 285 | 1 | 1 | 1 |
| 2014 | East | 1728 | 1186 | 1932 | 7 | 5 | 8 | 1293 | 917 | 1348 | 5 | 4 | 5 | 230 | 143 | 309 | 1 | 1 | 1 | 205 | 127 | 275 | 1 | 0 | 1 |
| 2015 | East | 1615 | 1108 | 1805 | 6 | 4 | 7 | 1207 | 856 | 1258 | 5 | 3 | 5 | 216 | 133 | 289 | 1 | 1 | 1 | 192 | 119 | 258 | 1 | 0 | 1 |
| 2016 | East | 1455 | 998 | 1626 | 6 | 4 | 6 | 1087 | 771 | 1133 | 4 | 3 | 4 | 195 | 120 | 261 | 1 | 0 | 1 | 173 | 107 | 232 | 1 | 0 | 1 |
| 2017 | East | 1292 | 887 | 1443 | 5 | 3 | 5 | 969 | 687 | 1010 | 4 | 3 | 4 | 171 | 106 | 229 | 1 | 0 | 1 | 152 | 94 | 204 | 1 | 0 | 1 |
| 2010 | West | 8229 | 5676 | 9015 | 36 | 25 | 39 | 6968 | 4941 | 7262 | 30 | 21 | 31 | 667 | 389 | 927 | 3 | 2 | 4 | 594 | 346 | 826 | 3 | 2 | 4 |
| 2011 | West | 7484 | 5200 | 8232 | 34 | 23 | 37 | 6287 | 4458 | 6552 | 28 | 20 | 29 | 633 | 392 | 889 | 3 | 2 | 4 | 564 | 349 | 792 | 3 | 2 | 4 |
| 2012 | West | 6758 | 4687 | 7436 | 30 | 21 | 34 | 5641 | 4000 | 5879 | 25 | 18 | 27 | 591 | 363 | 824 | 3 | 2 | 4 | 526 | 323 | 733 | 2 | 1 | 3 |
| 2013 | West | 6103 | 4224 | 6718 | 27 | 19 | 30 | 5059 | 3587 | 5272 | 23 | 16 | 24 | 552 | 337 | 765 | 2 | 2 | 3 | 492 | 300 | 681 | 2 | 1 | 3 |
| 2014 | West | 5658 | 3909 | 6225 | 25 | 17 | 27 | 4671 | 3312 | 4867 | 21 | 15 | 21 | 522 | 316 | 718 | 2 | 1 | 3 | 465 | 281 | 639 | 2 | 1 | 3 |
| 2015 | West | 5124 | 3540 | 5640 | 23 | 16 | 25 | 4225 | 2996 | 4403 | 19 | 13 | 20 | 476 | 288 | 655 | 2 | 1 | 3 | 424 | 256 | 583 | 2 | 1 | 3 |
| 2016 | West | 4530 | 3127 | 4987 | 20 | 14 | 22 | 3733 | 2647 | 3891 | 17 | 12 | 17 | 421 | 254 | 580 | 2 | 1 | 3 | 375 | 226 | 516 | 2 | 1 | 2 |
| 2017 | West | 3965 | 2763 | 4377 | 17 | 12 | 19 | 3271 | 2320 | 3409 | 14 | 10 | 15 | 367 | 234 | 512 | 2 | 1 | 2 | 326 | 209 | 456 | 1 | 1 | 2 |
| 2010 | National | 15632 | 10761 | 17277 | 19 | 13 | 21 | 12546 | 8896 | 13075 | 15 | 11 | 16 | 1633 | 986 | 2223 | 2 | 1 | 3 | 1453 | 878 | 1979 | 2 | 1 | 2 |
| 2011 | National | 14354 | 9914 | 15915 | 19 | 13 | 21 | 11414 | 8094 | 11896 | 15 | 10 | 15 | 1555 | 963 | 2126 | 2 | 1 | 3 | 1385 | 857 | 1893 | 2 | 1 | 2 |
| 2012 | National | 13086 | 9026 | 14519 | 17 | 12 | 19 | 10335 | 7329 | 10771 | 13 | 9 | 14 | 1455 | 898 | 1983 | 2 | 1 | 3 | 1295 | 799 | 1765 | 2 | 1 | 2 |
| 2013 | National | 11948 | 8230 | 13270 | 15 | 10 | 17 | 9366 | 6641 | 9760 | 12 | 8 | 12 | 1366 | 841 | 1857 | 2 | 1 | 2 | 1216 | 748 | 1653 | 2 | 1 | 2 |
| 2014 | National | 11179 | 7692 | 12417 | 14 | 10 | 15 | 8730 | 6191 | 9098 | 11 | 8 | 11 | 1295 | 794 | 1755 | 2 | 1 | 2 | 1153 | 707 | 1563 | 1 | 1 | 2 |
| 2015 | National | 10223 | 7034 | 11360 | 13 | 9 | 14 | 7972 | 5653 | 8308 | 10 | 7 | 10 | 1191 | 731 | 1614 | 2 | 1 | 2 | 1060 | 650 | 1437 | 1 | 1 | 2 |
| 2016 | National | 9100 | 6258 | 10113 | 11 | 8 | 13 | 7091 | 5029 | 7390 | 9 | 6 | 9 | 1063 | 651 | 1440 | 1 | 1 | 2 | 946 | 579 | 1282 | 1 | 1 | 2 |
| 2017 | National | 8010 | 5535 | 8912 | 10 | 7 | 11 | 6253 | 4434 | 6517 | 8 | 5 | 8 | 929 | 582 | 1267 | 1 | 1 | 2 | 827 | 519 | 1128 | 1 | 1 | 1 |

**Webappendix 9: Hib deaths in China by region, province, and year**

| **Year** | **Province** | **Hib deaths** | | | | | | **Hib pneumonia deaths** | | | | | | **Hib meningitis deaths** | | | | | | **Hib severe NPNM deaths** | | | | | |
| --- | --- | --- | --- | --- | --- | --- | --- | --- | --- | --- | --- | --- | --- | --- | --- | --- | --- | --- | --- | --- | --- | --- | --- | --- | --- |
|  |  | **Number** | | | **Rate per 100 000** | | | **Number** | | | **Rate per 100 000** | | | **Number** | | | **Rate per 100 000** | | | **Number** | | | **Rate per 100 000** | | |
|  |  | **Mean** | **UR** | | **Mean** | **UR** | | **Mean** | **UR** | | **Mean** | **UR** | | **Mean** | **UR** | | **Mean** | **UR** | | **Mean** | **UR** | | **Mean** | **UR** | |
| 2010 | Anhui | 255 | 174 | 347 | 7 | 5 | 9 | 231 | 162 | 303 | 6 | 4 | 8 | 24 | 11 | 44 | 1 | 0 | 1 | 0 | 0 | 0 | 0 | 0 | 0 |
| 2011 | Anhui | 201 | 137 | 274 | 6 | 4 | 8 | 181 | 128 | 238 | 5 | 4 | 7 | 20 | 9 | 36 | 1 | 0 | 1 | 0 | 0 | 0 | 0 | 0 | 0 |
| 2012 | Anhui | 183 | 124 | 249 | 5 | 3 | 7 | 165 | 116 | 216 | 5 | 3 | 6 | 18 | 9 | 33 | 0 | 0 | 1 | 0 | 0 | 0 | 0 | 0 | 0 |
| 2013 | Anhui | 138 | 94 | 188 | 4 | 3 | 5 | 124 | 87 | 163 | 3 | 2 | 5 | 14 | 6 | 25 | 0 | 0 | 1 | 0 | 0 | 0 | 0 | 0 | 0 |
| 2014 | Anhui | 124 | 84 | 169 | 3 | 2 | 5 | 112 | 79 | 147 | 3 | 2 | 4 | 12 | 6 | 22 | 0 | 0 | 1 | 0 | 0 | 0 | 0 | 0 | 0 |
| 2015 | Anhui | 103 | 70 | 140 | 3 | 2 | 4 | 93 | 65 | 122 | 3 | 2 | 3 | 10 | 5 | 18 | 0 | 0 | 1 | 0 | 0 | 0 | 0 | 0 | 0 |
| 2016 | Anhui | 111 | 75 | 151 | 3 | 2 | 4 | 100 | 70 | 131 | 3 | 2 | 4 | 11 | 5 | 19 | 0 | 0 | 1 | 0 | 0 | 0 | 0 | 0 | 0 |
| 2017 | Anhui | 87 | 59 | 119 | 2 | 2 | 3 | 79 | 55 | 104 | 2 | 1 | 3 | 8 | 4 | 15 | 0 | 0 | 0 | 0 | 0 | 0 | 0 | 0 | 0 |
| 2010 | Beijing | 15 | 11 | 21 | 2 | 1 | 2 | 14 | 10 | 19 | 2 | 1 | 2 | 1 | 1 | 2 | 0 | 0 | 0 | 0 | 0 | 0 | 0 | 0 | 0 |
| 2011 | Beijing | 13 | 9 | 18 | 2 | 2 | 3 | 12 | 9 | 16 | 2 | 1 | 3 | 1 | 0 | 2 | 0 | 0 | 0 | 0 | 0 | 0 | 0 | 0 | 0 |
| 2012 | Beijing | 14 | 9 | 19 | 2 | 1 | 2 | 13 | 9 | 17 | 2 | 1 | 2 | 1 | 1 | 2 | 0 | 0 | 0 | 0 | 0 | 0 | 0 | 0 | 0 |
| 2013 | Beijing | 15 | 10 | 20 | 2 | 1 | 2 | 14 | 10 | 18 | 2 | 1 | 2 | 1 | 1 | 2 | 0 | 0 | 0 | 0 | 0 | 0 | 0 | 0 | 0 |
| 2014 | Beijing | 18 | 12 | 24 | 2 | 1 | 3 | 16 | 11 | 21 | 2 | 1 | 2 | 1 | 1 | 3 | 0 | 0 | 0 | 0 | 0 | 0 | 0 | 0 | 0 |
| 2015 | Beijing | 13 | 9 | 17 | 2 | 1 | 2 | 12 | 8 | 15 | 1 | 1 | 2 | 1 | 0 | 2 | 0 | 0 | 0 | 0 | 0 | 0 | 0 | 0 | 0 |
| 2016 | Beijing | 15 | 11 | 21 | 2 | 1 | 2 | 14 | 10 | 19 | 2 | 1 | 2 | 1 | 1 | 2 | 0 | 0 | 0 | 0 | 0 | 0 | 0 | 0 | 0 |
| 2017 | Beijing | 14 | 10 | 19 | 2 | 1 | 2 | 13 | 9 | 17 | 1 | 1 | 2 | 1 | 1 | 2 | 0 | 0 | 0 | 0 | 0 | 0 | 0 | 0 | 0 |
| 2010 | Chongqing | 93 | 64 | 125 | 6 | 4 | 9 | 86 | 61 | 113 | 6 | 4 | 8 | 7 | 3 | 12 | 0 | 0 | 1 | 0 | 0 | 0 | 0 | 0 | 0 |
| 2011 | Chongqing | 77 | 53 | 104 | 5 | 4 | 7 | 71 | 50 | 93 | 5 | 3 | 6 | 6 | 3 | 10 | 0 | 0 | 1 | 0 | 0 | 0 | 0 | 0 | 0 |
| 2012 | Chongqing | 60 | 41 | 81 | 4 | 3 | 5 | 55 | 39 | 73 | 4 | 2 | 5 | 5 | 2 | 8 | 0 | 0 | 1 | 0 | 0 | 0 | 0 | 0 | 0 |
| 2013 | Chongqing | 53 | 36 | 72 | 3 | 2 | 5 | 49 | 34 | 64 | 3 | 2 | 4 | 4 | 2 | 8 | 0 | 0 | 0 | 0 | 0 | 0 | 0 | 0 | 0 |
| 2014 | Chongqing | 46 | 31 | 62 | 3 | 2 | 4 | 42 | 30 | 55 | 3 | 2 | 3 | 4 | 2 | 7 | 0 | 0 | 0 | 0 | 0 | 0 | 0 | 0 | 0 |
| 2015 | Chongqing | 34 | 23 | 46 | 2 | 1 | 3 | 31 | 22 | 41 | 2 | 1 | 3 | 3 | 1 | 5 | 0 | 0 | 0 | 0 | 0 | 0 | 0 | 0 | 0 |
| 2016 | Chongqing | 37 | 26 | 51 | 2 | 2 | 3 | 34 | 24 | 45 | 2 | 2 | 3 | 3 | 1 | 6 | 0 | 0 | 0 | 0 | 0 | 0 | 0 | 0 | 0 |
| 2017 | Chongqing | 30 | 20 | 40 | 2 | 1 | 2 | 27 | 19 | 36 | 2 | 1 | 2 | 2 | 1 | 4 | 0 | 0 | 0 | 0 | 0 | 0 | 0 | 0 | 0 |
| 2010 | Fujian | 115 | 79 | 155 | 5 | 3 | 6 | 107 | 75 | 140 | 4 | 3 | 6 | 8 | 4 | 14 | 0 | 0 | 1 | 0 | 0 | 0 | 0 | 0 | 0 |
| 2011 | Fujian | 94 | 64 | 126 | 4 | 3 | 6 | 87 | 61 | 114 | 4 | 3 | 5 | 7 | 3 | 12 | 0 | 0 | 1 | 0 | 0 | 0 | 0 | 0 | 0 |
| 2012 | Fujian | 97 | 67 | 131 | 4 | 3 | 6 | 90 | 63 | 118 | 4 | 3 | 5 | 7 | 3 | 13 | 0 | 0 | 1 | 0 | 0 | 0 | 0 | 0 | 0 |
| 2013 | Fujian | 62 | 43 | 84 | 3 | 2 | 4 | 57 | 40 | 75 | 2 | 2 | 3 | 5 | 2 | 9 | 0 | 0 | 0 | 0 | 0 | 0 | 0 | 0 | 0 |
| 2014 | Fujian | 63 | 43 | 85 | 3 | 2 | 4 | 58 | 41 | 76 | 2 | 2 | 3 | 5 | 2 | 9 | 0 | 0 | 0 | 0 | 0 | 0 | 0 | 0 | 0 |
| 2015 | Fujian | 52 | 35 | 70 | 2 | 1 | 3 | 47 | 33 | 62 | 2 | 1 | 3 | 4 | 2 | 8 | 0 | 0 | 0 | 0 | 0 | 0 | 0 | 0 | 0 |
| 2016 | Fujian | 58 | 40 | 79 | 2 | 2 | 3 | 53 | 37 | 70 | 2 | 2 | 3 | 5 | 2 | 9 | 0 | 0 | 0 | 0 | 0 | 0 | 0 | 0 | 0 |
| 2017 | Fujian | 50 | 34 | 68 | 2 | 1 | 3 | 46 | 32 | 60 | 2 | 1 | 2 | 4 | 2 | 8 | 0 | 0 | 0 | 0 | 0 | 0 | 0 | 0 | 0 |
| 2010 | Gansu | 378 | 262 | 521 | 24 | 16 | 33 | 336 | 237 | 442 | 21 | 15 | 28 | 41 | 25 | 79 | 3 | 2 | 5 | 0 | 0 | 1 | 0 | 0 | 0 |
| 2011 | Gansu | 326 | 218 | 434 | 22 | 15 | 29 | 291 | 205 | 382 | 19 | 14 | 26 | 35 | 13 | 51 | 2 | 1 | 3 | 0 | 0 | 0 | 0 | 0 | 0 |
| 2012 | Gansu | 280 | 188 | 376 | 20 | 13 | 27 | 250 | 176 | 328 | 18 | 12 | 23 | 30 | 12 | 47 | 2 | 1 | 3 | 0 | 0 | 0 | 0 | 0 | 0 |
| 2013 | Gansu | 239 | 161 | 323 | 17 | 11 | 23 | 213 | 150 | 280 | 15 | 11 | 20 | 25 | 11 | 43 | 2 | 1 | 3 | 0 | 0 | 0 | 0 | 0 | 0 |
| 2014 | Gansu | 211 | 143 | 288 | 14 | 10 | 20 | 189 | 133 | 248 | 13 | 9 | 17 | 22 | 10 | 39 | 1 | 1 | 3 | 0 | 0 | 0 | 0 | 0 | 0 |
| 2015 | Gansu | 181 | 123 | 248 | 12 | 8 | 17 | 162 | 114 | 213 | 11 | 8 | 15 | 19 | 9 | 35 | 1 | 1 | 2 | 0 | 0 | 0 | 0 | 0 | 0 |
| 2016 | Gansu | 157 | 107 | 215 | 11 | 7 | 15 | 140 | 99 | 184 | 10 | 7 | 13 | 17 | 8 | 30 | 1 | 1 | 2 | 0 | 0 | 0 | 0 | 0 | 0 |
| 2017 | Gansu | 133 | 90 | 181 | 9 | 6 | 12 | 119 | 84 | 156 | 8 | 6 | 11 | 14 | 7 | 25 | 1 | 0 | 2 | 0 | 0 | 0 | 0 | 0 | 0 |
| 2010 | Guangdong | 250 | 172 | 338 | 4 | 3 | 6 | 232 | 163 | 305 | 4 | 3 | 5 | 18 | 9 | 33 | 0 | 0 | 1 | 0 | 0 | 0 | 0 | 0 | 0 |
| 2011 | Guangdong | 183 | 125 | 248 | 3 | 2 | 5 | 169 | 119 | 222 | 3 | 2 | 4 | 14 | 7 | 25 | 0 | 0 | 0 | 0 | 0 | 0 | 0 | 0 | 0 |
| 2012 | Guangdong | 189 | 129 | 256 | 3 | 2 | 4 | 174 | 122 | 229 | 3 | 2 | 4 | 15 | 7 | 27 | 0 | 0 | 0 | 0 | 0 | 0 | 0 | 0 | 0 |
| 2013 | Guangdong | 241 | 165 | 326 | 4 | 3 | 5 | 221 | 156 | 291 | 3 | 2 | 4 | 20 | 9 | 36 | 0 | 0 | 1 | 0 | 0 | 0 | 0 | 0 | 0 |
| 2014 | Guangdong | 156 | 107 | 211 | 2 | 2 | 3 | 143 | 100 | 188 | 2 | 1 | 3 | 13 | 6 | 23 | 0 | 0 | 0 | 0 | 0 | 0 | 0 | 0 | 0 |
| 2015 | Guangdong | 136 | 93 | 184 | 2 | 1 | 3 | 124 | 88 | 164 | 2 | 1 | 2 | 11 | 5 | 20 | 0 | 0 | 0 | 0 | 0 | 0 | 0 | 0 | 0 |
| 2016 | Guangdong | 155 | 106 | 210 | 2 | 2 | 3 | 142 | 100 | 187 | 2 | 1 | 3 | 13 | 6 | 23 | 0 | 0 | 0 | 0 | 0 | 0 | 0 | 0 | 0 |
| 2017 | Guangdong | 136 | 93 | 184 | 2 | 1 | 2 | 125 | 88 | 164 | 2 | 1 | 2 | 11 | 5 | 20 | 0 | 0 | 0 | 0 | 0 | 0 | 0 | 0 | 0 |
| 2010 | Guangxi | 191 | 131 | 257 | 5 | 4 | 7 | 178 | 125 | 234 | 5 | 4 | 7 | 13 | 6 | 23 | 0 | 0 | 1 | 0 | 0 | 0 | 0 | 0 | 0 |
| 2011 | Guangxi | 179 | 123 | 241 | 5 | 3 | 7 | 166 | 117 | 218 | 5 | 3 | 6 | 13 | 6 | 23 | 0 | 0 | 1 | 0 | 0 | 0 | 0 | 0 | 0 |
| 2012 | Guangxi | 159 | 109 | 215 | 4 | 3 | 6 | 148 | 104 | 194 | 4 | 3 | 5 | 11 | 5 | 21 | 0 | 0 | 1 | 0 | 0 | 0 | 0 | 0 | 0 |
| 2013 | Guangxi | 139 | 95 | 187 | 4 | 3 | 5 | 129 | 90 | 169 | 3 | 2 | 4 | 10 | 5 | 18 | 0 | 0 | 0 | 0 | 0 | 0 | 0 | 0 | 0 |
| 2014 | Guangxi | 133 | 91 | 180 | 3 | 2 | 5 | 123 | 87 | 162 | 3 | 2 | 4 | 10 | 5 | 18 | 0 | 0 | 0 | 0 | 0 | 0 | 0 | 0 | 0 |
| 2015 | Guangxi | 115 | 79 | 156 | 3 | 2 | 4 | 107 | 75 | 140 | 3 | 2 | 4 | 8 | 4 | 15 | 0 | 0 | 0 | 0 | 0 | 0 | 0 | 0 | 0 |
| 2016 | Guangxi | 115 | 79 | 156 | 3 | 2 | 4 | 107 | 75 | 141 | 3 | 2 | 4 | 8 | 4 | 15 | 0 | 0 | 0 | 0 | 0 | 0 | 0 | 0 | 0 |
| 2017 | Guangxi | 100 | 69 | 135 | 2 | 2 | 3 | 93 | 65 | 122 | 2 | 2 | 3 | 7 | 3 | 13 | 0 | 0 | 0 | 0 | 0 | 0 | 0 | 0 | 0 |
| 2010 | Guizhou | 297 | 205 | 398 | 12 | 8 | 16 | 282 | 198 | 370 | 11 | 8 | 15 | 15 | 7 | 27 | 1 | 0 | 1 | 0 | 0 | 0 | 0 | 0 | 0 |
| 2011 | Guizhou | 244 | 169 | 327 | 10 | 7 | 13 | 231 | 163 | 304 | 9 | 6 | 12 | 13 | 6 | 23 | 1 | 0 | 1 | 0 | 0 | 0 | 0 | 0 | 0 |
| 2012 | Guizhou | 200 | 138 | 268 | 8 | 5 | 11 | 189 | 133 | 248 | 7 | 5 | 10 | 11 | 5 | 20 | 0 | 0 | 1 | 0 | 0 | 0 | 0 | 0 | 0 |
| 2013 | Guizhou | 166 | 115 | 223 | 7 | 5 | 9 | 157 | 110 | 206 | 6 | 4 | 8 | 9 | 4 | 17 | 0 | 0 | 1 | 0 | 0 | 0 | 0 | 0 | 0 |
| 2014 | Guizhou | 142 | 98 | 191 | 5 | 4 | 7 | 134 | 94 | 176 | 5 | 4 | 7 | 8 | 4 | 14 | 0 | 0 | 1 | 0 | 0 | 0 | 0 | 0 | 0 |
| 2015 | Guizhou | 113 | 78 | 152 | 4 | 3 | 6 | 107 | 75 | 140 | 4 | 3 | 5 | 6 | 3 | 11 | 0 | 0 | 0 | 0 | 0 | 0 | 0 | 0 | 0 |
| 2016 | Guizhou | 105 | 72 | 140 | 4 | 3 | 6 | 99 | 69 | 130 | 4 | 3 | 5 | 6 | 3 | 10 | 0 | 0 | 0 | 0 | 0 | 0 | 0 | 0 | 0 |
| 2017 | Guizhou | 86 | 60 | 116 | 3 | 2 | 4 | 82 | 57 | 107 | 3 | 2 | 4 | 5 | 2 | 8 | 0 | 0 | 0 | 0 | 0 | 0 | 0 | 0 | 0 |
| 2010 | Hainan | 86 | 59 | 115 | 13 | 9 | 18 | 81 | 57 | 106 | 13 | 9 | 17 | 5 | 2 | 9 | 1 | 0 | 1 | 0 | 0 | 0 | 0 | 0 | 0 |
| 2011 | Hainan | 70 | 49 | 95 | 11 | 8 | 15 | 66 | 47 | 87 | 11 | 8 | 14 | 4 | 2 | 8 | 1 | 0 | 1 | 0 | 0 | 0 | 0 | 0 | 0 |
| 2012 | Hainan | 63 | 44 | 85 | 10 | 7 | 13 | 60 | 42 | 78 | 9 | 6 | 12 | 4 | 2 | 7 | 1 | 0 | 1 | 0 | 0 | 0 | 0 | 0 | 0 |
| 2013 | Hainan | 57 | 39 | 77 | 9 | 6 | 12 | 54 | 38 | 70 | 8 | 6 | 11 | 4 | 2 | 6 | 1 | 0 | 1 | 0 | 0 | 0 | 0 | 0 | 0 |
| 2014 | Hainan | 59 | 41 | 80 | 9 | 6 | 12 | 56 | 39 | 73 | 9 | 6 | 11 | 4 | 2 | 7 | 1 | 0 | 1 | 0 | 0 | 0 | 0 | 0 | 0 |
| 2015 | Hainan | 53 | 36 | 71 | 8 | 6 | 11 | 49 | 35 | 65 | 8 | 5 | 10 | 3 | 1 | 6 | 0 | 0 | 1 | 0 | 0 | 0 | 0 | 0 | 0 |
| 2016 | Hainan | 57 | 39 | 77 | 9 | 6 | 12 | 54 | 38 | 70 | 8 | 6 | 11 | 3 | 2 | 6 | 1 | 0 | 1 | 0 | 0 | 0 | 0 | 0 | 0 |
| 2017 | Hainan | 47 | 32 | 63 | 7 | 5 | 10 | 44 | 31 | 58 | 7 | 5 | 9 | 3 | 1 | 5 | 0 | 0 | 1 | 0 | 0 | 0 | 0 | 0 | 0 |
| 2010 | Hebei | 323 | 217 | 448 | 6 | 4 | 9 | 277 | 195 | 364 | 6 | 4 | 7 | 46 | 22 | 83 | 1 | 0 | 2 | 0 | 0 | 1 | 0 | 0 | 0 |
| 2011 | Hebei | 290 | 194 | 402 | 6 | 4 | 8 | 248 | 174 | 326 | 5 | 4 | 7 | 42 | 20 | 76 | 1 | 0 | 2 | 0 | 0 | 1 | 0 | 0 | 0 |
| 2012 | Hebei | 272 | 182 | 377 | 5 | 4 | 8 | 232 | 163 | 305 | 5 | 3 | 6 | 39 | 19 | 71 | 1 | 0 | 1 | 0 | 0 | 1 | 0 | 0 | 0 |
| 2013 | Hebei | 255 | 170 | 353 | 5 | 3 | 7 | 217 | 153 | 285 | 4 | 3 | 6 | 37 | 17 | 67 | 1 | 0 | 1 | 0 | 0 | 1 | 0 | 0 | 0 |
| 2014 | Hebei | 251 | 168 | 348 | 5 | 3 | 7 | 214 | 151 | 281 | 4 | 3 | 5 | 36 | 17 | 66 | 1 | 0 | 1 | 0 | 0 | 1 | 0 | 0 | 0 |
| 2015 | Hebei | 240 | 160 | 332 | 5 | 3 | 7 | 205 | 144 | 269 | 4 | 3 | 5 | 35 | 16 | 62 | 1 | 0 | 1 | 0 | 0 | 0 | 0 | 0 | 0 |
| 2016 | Hebei | 242 | 162 | 336 | 5 | 3 | 7 | 207 | 146 | 273 | 4 | 3 | 5 | 35 | 16 | 63 | 1 | 0 | 1 | 0 | 0 | 0 | 0 | 0 | 0 |
| 2017 | Hebei | 201 | 135 | 278 | 4 | 3 | 5 | 172 | 121 | 226 | 3 | 2 | 4 | 28 | 13 | 51 | 1 | 0 | 1 | 0 | 0 | 0 | 0 | 0 | 0 |
| 2010 | Heilongjiang | 76 | 51 | 105 | 4 | 3 | 5 | 65 | 46 | 86 | 3 | 2 | 4 | 11 | 5 | 19 | 1 | 0 | 1 | 0 | 0 | 0 | 0 | 0 | 0 |
| 2011 | Heilongjiang | 67 | 45 | 92 | 4 | 3 | 6 | 57 | 40 | 75 | 4 | 3 | 5 | 9 | 4 | 17 | 1 | 0 | 1 | 0 | 0 | 0 | 0 | 0 | 0 |
| 2012 | Heilongjiang | 56 | 38 | 78 | 4 | 3 | 6 | 48 | 34 | 64 | 4 | 3 | 5 | 8 | 4 | 14 | 1 | 0 | 1 | 0 | 0 | 0 | 0 | 0 | 0 |
| 2013 | Heilongjiang | 50 | 33 | 69 | 4 | 3 | 5 | 43 | 30 | 56 | 3 | 2 | 4 | 7 | 3 | 13 | 1 | 0 | 1 | 0 | 0 | 0 | 0 | 0 | 0 |
| 2014 | Heilongjiang | 46 | 31 | 64 | 4 | 2 | 5 | 40 | 28 | 52 | 3 | 2 | 4 | 7 | 3 | 12 | 1 | 0 | 1 | 0 | 0 | 0 | 0 | 0 | 0 |
| 2015 | Heilongjiang | 40 | 27 | 55 | 3 | 2 | 4 | 34 | 24 | 45 | 3 | 2 | 3 | 6 | 3 | 10 | 0 | 0 | 1 | 0 | 0 | 0 | 0 | 0 | 0 |
| 2016 | Heilongjiang | 40 | 27 | 55 | 3 | 2 | 4 | 34 | 24 | 45 | 3 | 2 | 4 | 6 | 3 | 10 | 0 | 0 | 1 | 0 | 0 | 0 | 0 | 0 | 0 |
| 2017 | Heilongjiang | 31 | 21 | 42 | 3 | 2 | 4 | 26 | 19 | 35 | 2 | 2 | 3 | 4 | 2 | 8 | 0 | 0 | 1 | 0 | 0 | 0 | 0 | 0 | 0 |
| 2010 | Henan | 318 | 215 | 436 | 5 | 3 | 7 | 281 | 198 | 370 | 4 | 3 | 6 | 36 | 17 | 65 | 1 | 0 | 1 | 0 | 0 | 1 | 0 | 0 | 0 |
| 2011 | Henan | 273 | 185 | 375 | 4 | 3 | 5 | 241 | 169 | 316 | 3 | 2 | 4 | 32 | 15 | 58 | 0 | 0 | 1 | 0 | 0 | 0 | 0 | 0 | 0 |
| 2012 | Henan | 234 | 158 | 321 | 3 | 2 | 4 | 205 | 144 | 269 | 3 | 2 | 4 | 28 | 13 | 51 | 0 | 0 | 1 | 0 | 0 | 0 | 0 | 0 | 0 |
| 2013 | Henan | 208 | 140 | 286 | 3 | 2 | 4 | 182 | 128 | 239 | 2 | 2 | 3 | 26 | 12 | 47 | 0 | 0 | 1 | 0 | 0 | 0 | 0 | 0 | 0 |
| 2014 | Henan | 185 | 125 | 255 | 3 | 2 | 3 | 162 | 114 | 212 | 2 | 2 | 3 | 24 | 11 | 43 | 0 | 0 | 1 | 0 | 0 | 0 | 0 | 0 | 0 |
| 2015 | Henan | 163 | 110 | 225 | 2 | 2 | 3 | 142 | 100 | 187 | 2 | 1 | 3 | 21 | 10 | 38 | 0 | 0 | 1 | 0 | 0 | 0 | 0 | 0 | 0 |
| 2016 | Henan | 166 | 112 | 229 | 2 | 2 | 3 | 145 | 102 | 190 | 2 | 1 | 3 | 21 | 10 | 38 | 0 | 0 | 1 | 0 | 0 | 0 | 0 | 0 | 0 |
| 2017 | Henan | 127 | 86 | 175 | 2 | 1 | 2 | 111 | 78 | 146 | 1 | 1 | 2 | 16 | 8 | 29 | 0 | 0 | 0 | 0 | 0 | 0 | 0 | 0 | 0 |
| 2010 | Hubei | 109 | 75 | 146 | 3 | 2 | 4 | 102 | 72 | 134 | 3 | 2 | 4 | 7 | 3 | 12 | 0 | 0 | 0 | 0 | 0 | 0 | 0 | 0 | 0 |
| 2011 | Hubei | 89 | 61 | 120 | 3 | 2 | 4 | 84 | 59 | 110 | 3 | 2 | 4 | 6 | 3 | 10 | 0 | 0 | 0 | 0 | 0 | 0 | 0 | 0 | 0 |
| 2012 | Hubei | 72 | 50 | 98 | 2 | 2 | 3 | 68 | 48 | 89 | 2 | 2 | 3 | 5 | 2 | 8 | 0 | 0 | 0 | 0 | 0 | 0 | 0 | 0 | 0 |
| 2013 | Hubei | 62 | 43 | 84 | 2 | 1 | 3 | 58 | 41 | 77 | 2 | 1 | 3 | 4 | 2 | 8 | 0 | 0 | 0 | 0 | 0 | 0 | 0 | 0 | 0 |
| 2014 | Hubei | 59 | 40 | 79 | 2 | 1 | 3 | 55 | 38 | 72 | 2 | 1 | 2 | 4 | 2 | 7 | 0 | 0 | 0 | 0 | 0 | 0 | 0 | 0 | 0 |
| 2015 | Hubei | 50 | 35 | 68 | 2 | 1 | 2 | 47 | 33 | 62 | 2 | 1 | 2 | 3 | 2 | 6 | 0 | 0 | 0 | 0 | 0 | 0 | 0 | 0 | 0 |
| 2016 | Hubei | 50 | 35 | 68 | 2 | 1 | 2 | 47 | 33 | 61 | 2 | 1 | 2 | 4 | 2 | 6 | 0 | 0 | 0 | 0 | 0 | 0 | 0 | 0 | 0 |
| 2017 | Hubei | 40 | 28 | 55 | 1 | 1 | 2 | 38 | 26 | 49 | 1 | 1 | 2 | 3 | 1 | 5 | 0 | 0 | 0 | 0 | 0 | 0 | 0 | 0 | 0 |
| 2010 | Hunan | 150 | 104 | 203 | 4 | 3 | 5 | 141 | 99 | 185 | 3 | 2 | 4 | 10 | 5 | 18 | 0 | 0 | 0 | 0 | 0 | 0 | 0 | 0 | 0 |
| 2011 | Hunan | 120 | 82 | 161 | 3 | 2 | 4 | 112 | 79 | 147 | 3 | 2 | 3 | 8 | 4 | 14 | 0 | 0 | 0 | 0 | 0 | 0 | 0 | 0 | 0 |
| 2012 | Hunan | 108 | 74 | 146 | 3 | 2 | 4 | 101 | 71 | 133 | 2 | 2 | 3 | 7 | 3 | 13 | 0 | 0 | 0 | 0 | 0 | 0 | 0 | 0 | 0 |
| 2013 | Hunan | 89 | 61 | 120 | 2 | 1 | 3 | 83 | 58 | 109 | 2 | 1 | 3 | 6 | 3 | 11 | 0 | 0 | 0 | 0 | 0 | 0 | 0 | 0 | 0 |
| 2014 | Hunan | 83 | 57 | 111 | 2 | 1 | 3 | 77 | 54 | 101 | 2 | 1 | 2 | 6 | 3 | 10 | 0 | 0 | 0 | 0 | 0 | 0 | 0 | 0 | 0 |
| 2015 | Hunan | 72 | 49 | 97 | 2 | 1 | 2 | 67 | 47 | 88 | 2 | 1 | 2 | 5 | 2 | 9 | 0 | 0 | 0 | 0 | 0 | 0 | 0 | 0 | 0 |
| 2016 | Hunan | 68 | 47 | 92 | 2 | 1 | 2 | 63 | 45 | 83 | 1 | 1 | 2 | 5 | 2 | 8 | 0 | 0 | 0 | 0 | 0 | 0 | 0 | 0 | 0 |
| 2017 | Hunan | 56 | 38 | 75 | 1 | 1 | 2 | 52 | 36 | 68 | 1 | 1 | 2 | 4 | 2 | 7 | 0 | 0 | 0 | 0 | 0 | 0 | 0 | 0 | 0 |
| 2010 | Inner Mongolia | 106 | 72 | 145 | 7 | 5 | 10 | 94 | 66 | 124 | 7 | 5 | 9 | 12 | 6 | 21 | 1 | 0 | 1 | 0 | 0 | 0 | 0 | 0 | 0 |
| 2011 | Inner Mongolia | 97 | 66 | 133 | 8 | 5 | 11 | 86 | 60 | 113 | 7 | 5 | 9 | 11 | 5 | 20 | 1 | 0 | 2 | 0 | 0 | 0 | 0 | 0 | 0 |
| 2012 | Inner Mongolia | 85 | 57 | 116 | 8 | 5 | 10 | 75 | 53 | 98 | 7 | 5 | 9 | 10 | 5 | 18 | 1 | 0 | 2 | 0 | 0 | 0 | 0 | 0 | 0 |
| 2013 | Inner Mongolia | 78 | 53 | 107 | 7 | 5 | 10 | 69 | 48 | 90 | 6 | 4 | 8 | 9 | 4 | 17 | 1 | 0 | 2 | 0 | 0 | 0 | 0 | 0 | 0 |
| 2014 | Inner Mongolia | 78 | 53 | 107 | 7 | 5 | 9 | 69 | 48 | 90 | 6 | 4 | 8 | 9 | 4 | 17 | 1 | 0 | 1 | 0 | 0 | 0 | 0 | 0 | 0 |
| 2015 | Inner Mongolia | 73 | 49 | 101 | 7 | 4 | 9 | 64 | 45 | 85 | 6 | 4 | 8 | 9 | 4 | 16 | 1 | 0 | 1 | 0 | 0 | 0 | 0 | 0 | 0 |
| 2016 | Inner Mongolia | 73 | 49 | 100 | 7 | 4 | 9 | 64 | 45 | 84 | 6 | 4 | 8 | 9 | 4 | 16 | 1 | 0 | 1 | 0 | 0 | 0 | 0 | 0 | 0 |
| 2017 | Inner Mongolia | 63 | 43 | 86 | 6 | 4 | 8 | 55 | 39 | 73 | 5 | 4 | 7 | 7 | 4 | 14 | 1 | 0 | 1 | 0 | 0 | 0 | 0 | 0 | 0 |
| 2010 | Jiangsu | 60 | 41 | 82 | 1 | 1 | 2 | 55 | 38 | 72 | 1 | 1 | 2 | 6 | 3 | 10 | 0 | 0 | 0 | 0 | 0 | 0 | 0 | 0 | 0 |
| 2011 | Jiangsu | 54 | 37 | 73 | 1 | 1 | 2 | 48 | 34 | 64 | 1 | 1 | 2 | 5 | 2 | 10 | 0 | 0 | 0 | 0 | 0 | 0 | 0 | 0 | 0 |
| 2012 | Jiangsu | 53 | 36 | 72 | 1 | 1 | 2 | 48 | 34 | 63 | 1 | 1 | 2 | 5 | 3 | 10 | 0 | 0 | 0 | 0 | 0 | 0 | 0 | 0 | 0 |
| 2013 | Jiangsu | 50 | 34 | 68 | 1 | 1 | 2 | 45 | 31 | 59 | 1 | 1 | 2 | 5 | 2 | 9 | 0 | 0 | 0 | 0 | 0 | 0 | 0 | 0 | 0 |
| 2014 | Jiangsu | 47 | 32 | 64 | 1 | 1 | 2 | 42 | 29 | 55 | 1 | 1 | 1 | 5 | 2 | 9 | 0 | 0 | 0 | 0 | 0 | 0 | 0 | 0 | 0 |
| 2015 | Jiangsu | 42 | 28 | 57 | 1 | 1 | 1 | 37 | 26 | 49 | 1 | 1 | 1 | 5 | 2 | 8 | 0 | 0 | 0 | 0 | 0 | 0 | 0 | 0 | 0 |
| 2016 | Jiangsu | 40 | 27 | 55 | 1 | 1 | 1 | 36 | 25 | 47 | 1 | 1 | 1 | 4 | 2 | 8 | 0 | 0 | 0 | 0 | 0 | 0 | 0 | 0 | 0 |
| 2017 | Jiangsu | 35 | 24 | 48 | 1 | 1 | 1 | 31 | 22 | 41 | 1 | 1 | 1 | 4 | 2 | 7 | 0 | 0 | 0 | 0 | 0 | 0 | 0 | 0 | 0 |
| 2010 | Jiangxi | 408 | 280 | 551 | 14 | 9 | 18 | 379 | 266 | 498 | 13 | 9 | 16 | 29 | 14 | 53 | 1 | 0 | 2 | 0 | 0 | 0 | 0 | 0 | 0 |
| 2011 | Jiangxi | 338 | 231 | 456 | 10 | 7 | 14 | 312 | 220 | 410 | 10 | 7 | 13 | 25 | 12 | 45 | 1 | 0 | 1 | 0 | 0 | 0 | 0 | 0 | 0 |
| 2012 | Jiangxi | 357 | 245 | 483 | 10 | 7 | 14 | 330 | 232 | 433 | 10 | 7 | 13 | 27 | 13 | 49 | 1 | 0 | 1 | 0 | 0 | 0 | 0 | 0 | 0 |
| 2013 | Jiangxi | 309 | 212 | 418 | 9 | 6 | 12 | 284 | 200 | 374 | 8 | 6 | 11 | 24 | 11 | 44 | 1 | 0 | 1 | 0 | 0 | 0 | 0 | 0 | 0 |
| 2014 | Jiangxi | 222 | 152 | 301 | 7 | 5 | 9 | 204 | 144 | 268 | 6 | 4 | 8 | 18 | 8 | 32 | 1 | 0 | 1 | 0 | 0 | 0 | 0 | 0 | 0 |
| 2015 | Jiangxi | 194 | 133 | 263 | 6 | 4 | 8 | 178 | 125 | 234 | 5 | 4 | 7 | 16 | 7 | 29 | 0 | 0 | 1 | 0 | 0 | 0 | 0 | 0 | 0 |
| 2016 | Jiangxi | 200 | 137 | 271 | 6 | 4 | 8 | 183 | 129 | 241 | 6 | 4 | 7 | 16 | 8 | 30 | 0 | 0 | 1 | 0 | 0 | 0 | 0 | 0 | 0 |
| 2017 | Jiangxi | 162 | 111 | 220 | 5 | 4 | 7 | 149 | 105 | 196 | 5 | 3 | 6 | 13 | 6 | 24 | 0 | 0 | 1 | 0 | 0 | 0 | 0 | 0 | 0 |
| 2010 | Jilin | 81 | 55 | 112 | 6 | 4 | 8 | 71 | 50 | 93 | 5 | 3 | 6 | 10 | 5 | 19 | 1 | 0 | 1 | 0 | 0 | 0 | 0 | 0 | 0 |
| 2011 | Jilin | 71 | 47 | 97 | 6 | 4 | 8 | 61 | 43 | 80 | 5 | 4 | 7 | 9 | 4 | 17 | 1 | 0 | 1 | 0 | 0 | 0 | 0 | 0 | 0 |
| 2012 | Jilin | 63 | 42 | 87 | 6 | 4 | 8 | 54 | 38 | 71 | 5 | 4 | 7 | 9 | 4 | 15 | 1 | 0 | 1 | 0 | 0 | 0 | 0 | 0 | 0 |
| 2013 | Jilin | 56 | 37 | 77 | 5 | 4 | 7 | 48 | 34 | 63 | 5 | 3 | 6 | 8 | 4 | 14 | 1 | 0 | 1 | 0 | 0 | 0 | 0 | 0 | 0 |
| 2014 | Jilin | 53 | 35 | 73 | 5 | 3 | 7 | 45 | 32 | 60 | 4 | 3 | 6 | 8 | 4 | 14 | 1 | 0 | 1 | 0 | 0 | 0 | 0 | 0 | 0 |
| 2015 | Jilin | 47 | 31 | 65 | 5 | 3 | 6 | 40 | 28 | 53 | 4 | 3 | 5 | 7 | 3 | 12 | 1 | 0 | 1 | 0 | 0 | 0 | 0 | 0 | 0 |
| 2016 | Jilin | 48 | 32 | 67 | 5 | 3 | 7 | 41 | 29 | 54 | 4 | 3 | 5 | 7 | 3 | 12 | 1 | 0 | 1 | 0 | 0 | 0 | 0 | 0 | 0 |
| 2017 | Jilin | 38 | 26 | 53 | 4 | 3 | 6 | 33 | 23 | 43 | 3 | 2 | 5 | 5 | 3 | 10 | 1 | 0 | 1 | 0 | 0 | 0 | 0 | 0 | 0 |
| 2010 | Liaoning | 36 | 24 | 49 | 2 | 1 | 2 | 31 | 22 | 41 | 1 | 1 | 2 | 4 | 2 | 8 | 0 | 0 | 0 | 0 | 0 | 0 | 0 | 0 | 0 |
| 2011 | Liaoning | 31 | 21 | 43 | 2 | 1 | 2 | 27 | 19 | 36 | 2 | 1 | 2 | 4 | 2 | 7 | 0 | 0 | 0 | 0 | 0 | 0 | 0 | 0 | 0 |
| 2012 | Liaoning | 28 | 19 | 39 | 2 | 1 | 3 | 24 | 17 | 32 | 2 | 1 | 2 | 4 | 2 | 6 | 0 | 0 | 0 | 0 | 0 | 0 | 0 | 0 | 0 |
| 2013 | Liaoning | 26 | 18 | 36 | 2 | 1 | 2 | 23 | 16 | 30 | 1 | 1 | 2 | 3 | 2 | 6 | 0 | 0 | 0 | 0 | 0 | 0 | 0 | 0 | 0 |
| 2014 | Liaoning | 24 | 16 | 33 | 2 | 1 | 2 | 21 | 15 | 28 | 1 | 1 | 2 | 3 | 1 | 6 | 0 | 0 | 0 | 0 | 0 | 0 | 0 | 0 | 0 |
| 2015 | Liaoning | 21 | 14 | 29 | 1 | 1 | 2 | 19 | 13 | 24 | 1 | 1 | 2 | 3 | 1 | 5 | 0 | 0 | 0 | 0 | 0 | 0 | 0 | 0 | 0 |
| 2016 | Liaoning | 20 | 13 | 27 | 1 | 1 | 2 | 17 | 12 | 23 | 1 | 1 | 2 | 2 | 1 | 5 | 0 | 0 | 0 | 0 | 0 | 0 | 0 | 0 | 0 |
| 2017 | Liaoning | 17 | 11 | 23 | 1 | 1 | 2 | 14 | 10 | 19 | 1 | 1 | 1 | 2 | 1 | 4 | 0 | 0 | 0 | 0 | 0 | 0 | 0 | 0 | 0 |
| 2010 | Ningxia | 75 | 51 | 100 | 17 | 12 | 23 | 70 | 49 | 91 | 16 | 11 | 21 | 5 | 2 | 9 | 1 | 1 | 2 | 0 | 0 | 0 | 0 | 0 | 0 |
| 2011 | Ningxia | 65 | 45 | 87 | 15 | 10 | 20 | 61 | 43 | 80 | 14 | 10 | 18 | 4 | 2 | 8 | 1 | 0 | 2 | 0 | 0 | 0 | 0 | 0 | 0 |
| 2012 | Ningxia | 59 | 40 | 79 | 14 | 9 | 18 | 55 | 38 | 72 | 13 | 9 | 16 | 4 | 2 | 7 | 1 | 0 | 2 | 0 | 0 | 0 | 0 | 0 | 0 |
| 2013 | Ningxia | 52 | 36 | 71 | 12 | 8 | 16 | 49 | 34 | 64 | 11 | 8 | 15 | 4 | 2 | 7 | 1 | 0 | 2 | 0 | 0 | 0 | 0 | 0 | 0 |
| 2014 | Ningxia | 47 | 32 | 64 | 10 | 7 | 14 | 44 | 31 | 57 | 9 | 7 | 12 | 3 | 2 | 6 | 1 | 0 | 1 | 0 | 0 | 0 | 0 | 0 | 0 |
| 2015 | Ningxia | 45 | 31 | 60 | 10 | 7 | 13 | 41 | 29 | 54 | 9 | 6 | 12 | 3 | 2 | 6 | 1 | 0 | 1 | 0 | 0 | 0 | 0 | 0 | 0 |
| 2016 | Ningxia | 42 | 29 | 57 | 9 | 6 | 13 | 39 | 27 | 51 | 9 | 6 | 11 | 3 | 1 | 6 | 1 | 0 | 1 | 0 | 0 | 0 | 0 | 0 | 0 |
| 2017 | Ningxia | 36 | 25 | 49 | 8 | 6 | 11 | 34 | 24 | 44 | 7 | 5 | 10 | 3 | 1 | 5 | 1 | 0 | 1 | 0 | 0 | 0 | 0 | 0 | 0 |
| 2010 | Qinghai | 140 | 96 | 191 | 38 | 26 | 52 | 126 | 89 | 166 | 35 | 24 | 45 | 14 | 7 | 25 | 4 | 2 | 7 | 0 | 0 | 0 | 0 | 0 | 0 |
| 2011 | Qinghai | 125 | 84 | 166 | 34 | 23 | 46 | 114 | 80 | 149 | 31 | 22 | 41 | 12 | 4 | 16 | 3 | 1 | 4 | 0 | 0 | 0 | 0 | 0 | 0 |
| 2012 | Qinghai | 110 | 74 | 147 | 31 | 21 | 41 | 100 | 71 | 132 | 28 | 20 | 37 | 10 | 4 | 15 | 3 | 1 | 4 | 0 | 0 | 0 | 0 | 0 | 0 |
| 2013 | Qinghai | 99 | 68 | 133 | 28 | 19 | 38 | 91 | 64 | 119 | 26 | 18 | 34 | 8 | 4 | 14 | 2 | 1 | 4 | 0 | 0 | 0 | 0 | 0 | 0 |
| 2014 | Qinghai | 90 | 62 | 122 | 24 | 17 | 33 | 83 | 58 | 109 | 22 | 16 | 29 | 7 | 3 | 13 | 2 | 1 | 3 | 0 | 0 | 0 | 0 | 0 | 0 |
| 2015 | Qinghai | 79 | 54 | 107 | 21 | 15 | 29 | 73 | 52 | 96 | 20 | 14 | 26 | 6 | 3 | 11 | 2 | 1 | 3 | 0 | 0 | 0 | 0 | 0 | 0 |
| 2016 | Qinghai | 72 | 49 | 98 | 19 | 13 | 26 | 66 | 47 | 87 | 18 | 12 | 23 | 6 | 3 | 10 | 1 | 1 | 3 | 0 | 0 | 0 | 0 | 0 | 0 |
| 2017 | Qinghai | 63 | 43 | 85 | 17 | 11 | 22 | 58 | 41 | 76 | 15 | 11 | 20 | 5 | 2 | 9 | 1 | 1 | 2 | 0 | 0 | 0 | 0 | 0 | 0 |
| 2010 | Shaanxi | 293 | 200 | 397 | 13 | 9 | 17 | 268 | 189 | 352 | 12 | 8 | 15 | 25 | 12 | 44 | 1 | 1 | 2 | 0 | 0 | 0 | 0 | 0 | 0 |
| 2011 | Shaanxi | 268 | 183 | 364 | 13 | 9 | 18 | 244 | 172 | 321 | 12 | 8 | 16 | 23 | 11 | 42 | 1 | 1 | 2 | 0 | 0 | 0 | 0 | 0 | 0 |
| 2012 | Shaanxi | 246 | 168 | 335 | 13 | 9 | 17 | 224 | 157 | 294 | 12 | 8 | 15 | 22 | 11 | 40 | 1 | 1 | 2 | 0 | 0 | 0 | 0 | 0 | 0 |
| 2013 | Shaanxi | 201 | 137 | 274 | 10 | 7 | 14 | 182 | 128 | 239 | 9 | 6 | 12 | 19 | 9 | 34 | 1 | 0 | 2 | 0 | 0 | 0 | 0 | 0 | 0 |
| 2014 | Shaanxi | 185 | 126 | 252 | 9 | 6 | 12 | 167 | 117 | 219 | 8 | 6 | 11 | 18 | 9 | 33 | 1 | 0 | 2 | 0 | 0 | 0 | 0 | 0 | 0 |
| 2015 | Shaanxi | 167 | 114 | 228 | 8 | 6 | 11 | 151 | 106 | 198 | 7 | 5 | 10 | 17 | 8 | 30 | 1 | 0 | 1 | 0 | 0 | 0 | 0 | 0 | 0 |
| 2016 | Shaanxi | 160 | 109 | 219 | 8 | 5 | 11 | 144 | 101 | 190 | 7 | 5 | 9 | 16 | 8 | 29 | 1 | 0 | 1 | 0 | 0 | 0 | 0 | 0 | 0 |
| 2017 | Shaanxi | 133 | 91 | 182 | 7 | 5 | 9 | 120 | 84 | 158 | 6 | 4 | 8 | 13 | 6 | 24 | 1 | 0 | 1 | 0 | 0 | 0 | 0 | 0 | 0 |
| 2010 | Shandong | 91 | 61 | 127 | 2 | 1 | 2 | 78 | 55 | 102 | 1 | 1 | 2 | 13 | 6 | 24 | 0 | 0 | 0 | 0 | 0 | 0 | 0 | 0 | 0 |
| 2011 | Shandong | 76 | 51 | 106 | 1 | 1 | 2 | 65 | 46 | 85 | 1 | 1 | 2 | 11 | 5 | 21 | 0 | 0 | 0 | 0 | 0 | 0 | 0 | 0 | 0 |
| 2012 | Shandong | 66 | 44 | 91 | 1 | 1 | 2 | 55 | 39 | 73 | 1 | 1 | 1 | 10 | 5 | 18 | 0 | 0 | 0 | 0 | 0 | 0 | 0 | 0 | 0 |
| 2013 | Shandong | 58 | 39 | 81 | 1 | 1 | 1 | 49 | 35 | 65 | 1 | 1 | 1 | 9 | 4 | 16 | 0 | 0 | 0 | 0 | 0 | 0 | 0 | 0 | 0 |
| 2014 | Shandong | 56 | 37 | 77 | 1 | 1 | 1 | 47 | 33 | 62 | 1 | 1 | 1 | 9 | 4 | 16 | 0 | 0 | 0 | 0 | 0 | 0 | 0 | 0 | 0 |
| 2015 | Shandong | 46 | 31 | 64 | 1 | 1 | 1 | 39 | 27 | 51 | 1 | 0 | 1 | 7 | 3 | 13 | 0 | 0 | 0 | 0 | 0 | 0 | 0 | 0 | 0 |
| 2016 | Shandong | 54 | 36 | 75 | 1 | 1 | 1 | 45 | 32 | 59 | 1 | 1 | 1 | 8 | 4 | 15 | 0 | 0 | 0 | 0 | 0 | 0 | 0 | 0 | 0 |
| 2017 | Shandong | 46 | 31 | 64 | 1 | 1 | 1 | 39 | 27 | 51 | 1 | 0 | 1 | 7 | 3 | 13 | 0 | 0 | 0 | 0 | 0 | 0 | 0 | 0 | 0 |
| 2010 | Shanghai | 15 | 11 | 21 | 2 | 1 | 2 | 14 | 10 | 19 | 2 | 1 | 2 | 1 | 0 | 2 | 0 | 0 | 0 | 0 | 0 | 0 | 0 | 0 | 0 |
| 2011 | Shanghai | 15 | 11 | 21 | 2 | 1 | 2 | 14 | 10 | 19 | 2 | 1 | 2 | 1 | 1 | 2 | 0 | 0 | 0 | 0 | 0 | 0 | 0 | 0 | 0 |
| 2012 | Shanghai | 18 | 12 | 24 | 2 | 1 | 3 | 17 | 12 | 22 | 2 | 1 | 2 | 1 | 1 | 2 | 0 | 0 | 0 | 0 | 0 | 0 | 0 | 0 | 0 |
| 2013 | Shanghai | 17 | 12 | 23 | 2 | 1 | 2 | 16 | 11 | 21 | 2 | 1 | 2 | 1 | 1 | 2 | 0 | 0 | 0 | 0 | 0 | 0 | 0 | 0 | 0 |
| 2014 | Shanghai | 8 | 5 | 11 | 1 | 1 | 1 | 7 | 5 | 10 | 1 | 1 | 1 | 1 | 0 | 1 | 0 | 0 | 0 | 0 | 0 | 0 | 0 | 0 | 0 |
| 2015 | Shanghai | 6 | 4 | 8 | 1 | 0 | 1 | 6 | 4 | 7 | 1 | 0 | 1 | 0 | 0 | 1 | 0 | 0 | 0 | 0 | 0 | 0 | 0 | 0 | 0 |
| 2016 | Shanghai | 8 | 5 | 10 | 1 | 1 | 1 | 7 | 5 | 9 | 1 | 1 | 1 | 1 | 0 | 1 | 0 | 0 | 0 | 0 | 0 | 0 | 0 | 0 | 0 |
| 2017 | Shanghai | 7 | 5 | 9 | 1 | 0 | 1 | 6 | 4 | 8 | 1 | 0 | 1 | 1 | 0 | 1 | 0 | 0 | 0 | 0 | 0 | 0 | 0 | 0 | 0 |
| 2010 | Shanxi | 200 | 136 | 273 | 9 | 6 | 13 | 179 | 126 | 235 | 8 | 6 | 11 | 21 | 10 | 37 | 1 | 0 | 2 | 0 | 0 | 0 | 0 | 0 | 0 |
| 2011 | Shanxi | 182 | 123 | 248 | 9 | 6 | 12 | 162 | 114 | 213 | 8 | 6 | 11 | 19 | 9 | 35 | 1 | 0 | 2 | 0 | 0 | 0 | 0 | 0 | 0 |
| 2012 | Shanxi | 169 | 115 | 231 | 9 | 6 | 13 | 151 | 106 | 198 | 8 | 6 | 11 | 18 | 9 | 33 | 1 | 0 | 2 | 0 | 0 | 0 | 0 | 0 | 0 |
| 2013 | Shanxi | 155 | 105 | 212 | 8 | 6 | 11 | 138 | 97 | 182 | 7 | 5 | 10 | 17 | 8 | 30 | 1 | 0 | 2 | 0 | 0 | 0 | 0 | 0 | 0 |
| 2014 | Shanxi | 150 | 102 | 205 | 8 | 5 | 11 | 133 | 94 | 175 | 7 | 5 | 9 | 16 | 8 | 30 | 1 | 0 | 2 | 0 | 0 | 0 | 0 | 0 | 0 |
| 2015 | Shanxi | 141 | 95 | 192 | 7 | 5 | 10 | 125 | 88 | 164 | 7 | 5 | 9 | 15 | 7 | 28 | 1 | 0 | 1 | 0 | 0 | 0 | 0 | 0 | 0 |
| 2016 | Shanxi | 134 | 91 | 183 | 7 | 5 | 10 | 119 | 84 | 156 | 6 | 4 | 8 | 15 | 7 | 26 | 1 | 0 | 1 | 0 | 0 | 0 | 0 | 0 | 0 |
| 2017 | Shanxi | 112 | 76 | 154 | 6 | 4 | 8 | 100 | 70 | 132 | 5 | 4 | 7 | 12 | 6 | 22 | 1 | 0 | 1 | 0 | 0 | 0 | 0 | 0 | 0 |
| 2010 | Sichuan | 610 | 422 | 817 | 13 | 9 | 18 | 580 | 408 | 762 | 13 | 9 | 17 | 30 | 14 | 54 | 1 | 0 | 1 | 0 | 0 | 0 | 0 | 0 | 0 |
| 2011 | Sichuan | 533 | 368 | 714 | 13 | 9 | 17 | 505 | 355 | 664 | 12 | 8 | 16 | 27 | 13 | 50 | 1 | 0 | 1 | 0 | 0 | 0 | 0 | 0 | 0 |
| 2012 | Sichuan | 427 | 295 | 573 | 10 | 7 | 14 | 404 | 284 | 531 | 10 | 7 | 13 | 23 | 11 | 41 | 1 | 0 | 1 | 0 | 0 | 0 | 0 | 0 | 0 |
| 2013 | Sichuan | 354 | 244 | 474 | 9 | 6 | 11 | 334 | 235 | 439 | 8 | 6 | 11 | 20 | 9 | 35 | 0 | 0 | 1 | 0 | 0 | 0 | 0 | 0 | 0 |
| 2014 | Sichuan | 303 | 209 | 406 | 7 | 5 | 10 | 285 | 201 | 375 | 7 | 5 | 9 | 17 | 8 | 31 | 0 | 0 | 1 | 0 | 0 | 0 | 0 | 0 | 0 |
| 2015 | Sichuan | 232 | 160 | 312 | 6 | 4 | 8 | 219 | 154 | 287 | 5 | 4 | 7 | 13 | 6 | 24 | 0 | 0 | 1 | 0 | 0 | 0 | 0 | 0 | 0 |
| 2016 | Sichuan | 227 | 157 | 305 | 5 | 4 | 7 | 214 | 150 | 281 | 5 | 4 | 7 | 13 | 6 | 24 | 0 | 0 | 1 | 0 | 0 | 0 | 0 | 0 | 0 |
| 2017 | Sichuan | 164 | 113 | 220 | 4 | 3 | 5 | 154 | 108 | 203 | 4 | 3 | 5 | 9 | 4 | 17 | 0 | 0 | 0 | 0 | 0 | 0 | 0 | 0 | 0 |
| 2010 | Tianjin | 19 | 13 | 25 | 3 | 2 | 4 | 17 | 12 | 22 | 3 | 2 | 4 | 1 | 1 | 3 | 0 | 0 | 0 | 0 | 0 | 0 | 0 | 0 | 0 |
| 2011 | Tianjin | 14 | 9 | 18 | 3 | 2 | 4 | 12 | 9 | 16 | 2 | 2 | 3 | 1 | 1 | 2 | 0 | 0 | 0 | 0 | 0 | 0 | 0 | 0 | 0 |
| 2012 | Tianjin | 14 | 10 | 19 | 3 | 2 | 4 | 13 | 9 | 17 | 3 | 2 | 3 | 1 | 1 | 2 | 0 | 0 | 0 | 0 | 0 | 0 | 0 | 0 | 0 |
| 2013 | Tianjin | 11 | 8 | 15 | 2 | 1 | 3 | 10 | 7 | 13 | 2 | 1 | 2 | 1 | 0 | 2 | 0 | 0 | 0 | 0 | 0 | 0 | 0 | 0 | 0 |
| 2014 | Tianjin | 11 | 8 | 15 | 2 | 1 | 3 | 10 | 7 | 14 | 2 | 1 | 2 | 1 | 0 | 2 | 0 | 0 | 0 | 0 | 0 | 0 | 0 | 0 | 0 |
| 2015 | Tianjin | 10 | 7 | 13 | 2 | 1 | 2 | 9 | 6 | 12 | 2 | 1 | 2 | 1 | 0 | 1 | 0 | 0 | 0 | 0 | 0 | 0 | 0 | 0 | 0 |
| 2016 | Tianjin | 14 | 10 | 20 | 2 | 2 | 3 | 13 | 9 | 17 | 2 | 2 | 3 | 1 | 1 | 2 | 0 | 0 | 0 | 0 | 0 | 0 | 0 | 0 | 0 |
| 2017 | Tianjin | 11 | 7 | 15 | 2 | 1 | 3 | 10 | 7 | 13 | 2 | 1 | 2 | 1 | 0 | 2 | 0 | 0 | 0 | 0 | 0 | 0 | 0 | 0 | 0 |
| 2010 | Tibet | 121 | 83 | 162 | 63 | 43 | 84 | 111 | 78 | 146 | 57 | 40 | 76 | 10 | 4 | 15 | 5 | 2 | 8 | 0 | 0 | 0 | 0 | 0 | 0 |
| 2011 | Tibet | 118 | 81 | 157 | 61 | 42 | 82 | 108 | 76 | 142 | 56 | 40 | 74 | 10 | 5 | 15 | 5 | 2 | 8 | 0 | 0 | 0 | 0 | 0 | 0 |
| 2012 | Tibet | 114 | 78 | 152 | 49 | 33 | 65 | 104 | 73 | 137 | 45 | 31 | 59 | 9 | 5 | 15 | 4 | 2 | 6 | 0 | 0 | 0 | 0 | 0 | 0 |
| 2013 | Tibet | 110 | 75 | 146 | 54 | 37 | 72 | 100 | 71 | 132 | 49 | 35 | 65 | 9 | 5 | 15 | 5 | 2 | 7 | 0 | 0 | 0 | 0 | 0 | 0 |
| 2014 | Tibet | 109 | 75 | 145 | 44 | 30 | 59 | 100 | 70 | 131 | 40 | 28 | 53 | 9 | 5 | 14 | 4 | 2 | 6 | 0 | 0 | 0 | 0 | 0 | 0 |
| 2015 | Tibet | 104 | 71 | 138 | 42 | 29 | 56 | 95 | 67 | 125 | 38 | 27 | 51 | 8 | 4 | 13 | 3 | 2 | 5 | 0 | 0 | 0 | 0 | 0 | 0 |
| 2016 | Tibet | 96 | 65 | 128 | 37 | 25 | 49 | 88 | 62 | 115 | 34 | 24 | 44 | 8 | 4 | 12 | 3 | 1 | 5 | 0 | 0 | 0 | 0 | 0 | 0 |
| 2017 | Tibet | 84 | 57 | 112 | 31 | 21 | 41 | 77 | 54 | 102 | 28 | 20 | 37 | 7 | 3 | 10 | 2 | 1 | 4 | 0 | 0 | 0 | 0 | 0 | 0 |
| 2010 | Xinjiang | 705 | 480 | 944 | 41 | 28 | 55 | 641 | 451 | 842 | 38 | 26 | 49 | 64 | 29 | 101 | 4 | 2 | 6 | 1 | 0 | 1 | 0 | 0 | 0 |
| 2011 | Xinjiang | 667 | 455 | 891 | 39 | 27 | 53 | 606 | 426 | 796 | 36 | 25 | 47 | 61 | 29 | 94 | 4 | 2 | 6 | 0 | 0 | 1 | 0 | 0 | 0 |
| 2012 | Xinjiang | 627 | 429 | 838 | 35 | 24 | 47 | 570 | 401 | 749 | 32 | 22 | 42 | 57 | 28 | 89 | 3 | 2 | 5 | 0 | 0 | 1 | 0 | 0 | 0 |
| 2013 | Xinjiang | 585 | 401 | 783 | 32 | 22 | 43 | 532 | 374 | 698 | 29 | 20 | 38 | 53 | 26 | 84 | 3 | 1 | 5 | 0 | 0 | 1 | 0 | 0 | 0 |
| 2014 | Xinjiang | 564 | 387 | 756 | 30 | 21 | 40 | 513 | 361 | 674 | 27 | 19 | 36 | 51 | 26 | 81 | 3 | 1 | 4 | 0 | 0 | 1 | 0 | 0 | 0 |
| 2015 | Xinjiang | 521 | 357 | 698 | 28 | 19 | 37 | 475 | 334 | 624 | 25 | 18 | 33 | 46 | 23 | 74 | 2 | 1 | 4 | 0 | 0 | 1 | 0 | 0 | 0 |
| 2016 | Xinjiang | 479 | 328 | 644 | 25 | 17 | 33 | 438 | 308 | 575 | 23 | 16 | 30 | 41 | 20 | 69 | 2 | 1 | 4 | 0 | 0 | 1 | 0 | 0 | 0 |
| 2017 | Xinjiang | 423 | 285 | 554 | 21 | 14 | 27 | 388 | 273 | 510 | 19 | 13 | 25 | 35 | 11 | 44 | 2 | 1 | 2 | 0 | 0 | 0 | 0 | 0 | 0 |
| 2010 | Yunnan | 840 | 583 | 1121 | 28 | 19 | 37 | 805 | 566 | 1057 | 27 | 19 | 35 | 35 | 17 | 63 | 1 | 1 | 2 | 0 | 0 | 1 | 0 | 0 | 0 |
| 2011 | Yunnan | 704 | 488 | 941 | 24 | 17 | 32 | 673 | 473 | 884 | 23 | 16 | 30 | 31 | 15 | 56 | 1 | 1 | 2 | 0 | 0 | 0 | 0 | 0 | 0 |
| 2012 | Yunnan | 638 | 442 | 853 | 22 | 15 | 30 | 608 | 428 | 799 | 21 | 15 | 28 | 30 | 14 | 53 | 1 | 0 | 2 | 0 | 0 | 0 | 0 | 0 | 0 |
| 2013 | Yunnan | 532 | 368 | 712 | 18 | 13 | 25 | 506 | 356 | 665 | 18 | 12 | 23 | 26 | 12 | 47 | 1 | 0 | 2 | 0 | 0 | 0 | 0 | 0 | 0 |
| 2014 | Yunnan | 493 | 341 | 660 | 17 | 12 | 23 | 468 | 329 | 615 | 16 | 11 | 21 | 25 | 12 | 45 | 1 | 0 | 2 | 0 | 0 | 0 | 0 | 0 | 0 |
| 2015 | Yunnan | 416 | 288 | 557 | 15 | 10 | 20 | 395 | 278 | 518 | 14 | 10 | 18 | 21 | 10 | 38 | 1 | 0 | 1 | 0 | 0 | 0 | 0 | 0 | 0 |
| 2016 | Yunnan | 381 | 264 | 511 | 13 | 9 | 18 | 362 | 255 | 476 | 13 | 9 | 17 | 19 | 9 | 35 | 1 | 0 | 1 | 0 | 0 | 0 | 0 | 0 | 0 |
| 2017 | Yunnan | 321 | 222 | 430 | 11 | 8 | 15 | 305 | 215 | 401 | 11 | 8 | 14 | 16 | 8 | 29 | 1 | 0 | 1 | 0 | 0 | 0 | 0 | 0 | 0 |
| 2010 | Zhejiang | 81 | 55 | 111 | 3 | 2 | 4 | 73 | 52 | 97 | 2 | 2 | 3 | 8 | 4 | 14 | 0 | 0 | 0 | 0 | 0 | 0 | 0 | 0 | 0 |
| 2011 | Zhejiang | 67 | 46 | 92 | 3 | 2 | 4 | 61 | 43 | 80 | 2 | 2 | 3 | 7 | 3 | 12 | 0 | 0 | 0 | 0 | 0 | 0 | 0 | 0 | 0 |
| 2012 | Zhejiang | 64 | 43 | 87 | 2 | 2 | 3 | 57 | 40 | 75 | 2 | 2 | 3 | 6 | 3 | 12 | 0 | 0 | 0 | 0 | 0 | 0 | 0 | 0 | 0 |
| 2013 | Zhejiang | 63 | 43 | 86 | 2 | 2 | 3 | 57 | 40 | 74 | 2 | 2 | 3 | 7 | 3 | 12 | 0 | 0 | 0 | 0 | 0 | 0 | 0 | 0 | 0 |
| 2014 | Zhejiang | 51 | 35 | 70 | 2 | 1 | 3 | 46 | 32 | 61 | 2 | 1 | 2 | 5 | 3 | 10 | 0 | 0 | 0 | 0 | 0 | 0 | 0 | 0 | 0 |
| 2015 | Zhejiang | 40 | 27 | 55 | 2 | 1 | 2 | 36 | 25 | 47 | 1 | 1 | 2 | 4 | 2 | 8 | 0 | 0 | 0 | 0 | 0 | 0 | 0 | 0 | 0 |
| 2016 | Zhejiang | 37 | 25 | 51 | 1 | 1 | 2 | 33 | 23 | 44 | 1 | 1 | 2 | 4 | 2 | 7 | 0 | 0 | 0 | 0 | 0 | 0 | 0 | 0 | 0 |
| 2017 | Zhejiang | 34 | 23 | 46 | 1 | 1 | 2 | 30 | 21 | 39 | 1 | 1 | 1 | 4 | 2 | 6 | 0 | 0 | 0 | 0 | 0 | 0 | 0 | 0 | 0 |
| 2010 | Central | 2007 | 1365 | 2736 | 6 | 4 | 8 | 1807 | 1271 | 2374 | 6 | 4 | 7 | 198 | 93 | 358 | 1 | 0 | 1 | 2 | 1 | 3 | 0 | 0 | 0 |
| 2011 | Central | 1701 | 1155 | 2321 | 5 | 4 | 7 | 1525 | 1072 | 2003 | 5 | 3 | 6 | 175 | 82 | 316 | 1 | 0 | 1 | 1 | 1 | 3 | 0 | 0 | 0 |
| 2012 | Central | 1578 | 1072 | 2155 | 5 | 3 | 7 | 1413 | 994 | 1857 | 4 | 3 | 6 | 164 | 77 | 295 | 1 | 0 | 1 | 1 | 1 | 2 | 0 | 0 | 0 |
| 2013 | Central | 1379 | 936 | 1885 | 4 | 3 | 6 | 1231 | 866 | 1617 | 4 | 3 | 5 | 147 | 69 | 265 | 0 | 0 | 1 | 1 | 1 | 2 | 0 | 0 | 0 |
| 2014 | Central | 1232 | 835 | 1685 | 4 | 3 | 5 | 1097 | 772 | 1442 | 3 | 2 | 5 | 134 | 63 | 242 | 0 | 0 | 1 | 1 | 1 | 2 | 0 | 0 | 0 |
| 2015 | Central | 1103 | 747 | 1509 | 3 | 2 | 5 | 981 | 690 | 1289 | 3 | 2 | 4 | 121 | 57 | 218 | 0 | 0 | 1 | 1 | 0 | 2 | 0 | 0 | 0 |
| 2016 | Central | 1116 | 756 | 1526 | 4 | 2 | 5 | 993 | 699 | 1305 | 3 | 2 | 4 | 122 | 57 | 220 | 0 | 0 | 1 | 1 | 0 | 2 | 0 | 0 | 0 |
| 2017 | Central | 902 | 612 | 1233 | 3 | 2 | 4 | 804 | 565 | 1056 | 3 | 2 | 3 | 97 | 46 | 175 | 0 | 0 | 1 | 1 | 0 | 1 | 0 | 0 | 0 |
| 2010 | East | 683 | 466 | 928 | 3 | 2 | 4 | 622 | 437 | 817 | 2 | 2 | 3 | 61 | 29 | 110 | 0 | 0 | 0 | 0 | 0 | 1 | 0 | 0 | 0 |
| 2011 | East | 548 | 373 | 745 | 2 | 2 | 3 | 496 | 349 | 652 | 2 | 1 | 3 | 51 | 24 | 93 | 0 | 0 | 0 | 0 | 0 | 1 | 0 | 0 | 0 |
| 2012 | East | 543 | 370 | 739 | 2 | 2 | 3 | 491 | 345 | 645 | 2 | 1 | 3 | 51 | 24 | 92 | 0 | 0 | 0 | 0 | 0 | 1 | 0 | 0 | 0 |
| 2013 | East | 545 | 371 | 741 | 2 | 2 | 3 | 492 | 346 | 646 | 2 | 1 | 3 | 52 | 25 | 94 | 0 | 0 | 0 | 0 | 0 | 1 | 0 | 0 | 0 |
| 2014 | East | 433 | 295 | 591 | 2 | 1 | 2 | 390 | 274 | 512 | 2 | 1 | 2 | 43 | 20 | 78 | 0 | 0 | 0 | 0 | 0 | 1 | 0 | 0 | 0 |
| 2015 | East | 366 | 249 | 499 | 1 | 1 | 2 | 329 | 231 | 432 | 1 | 1 | 2 | 37 | 17 | 66 | 0 | 0 | 0 | 0 | 0 | 1 | 0 | 0 | 0 |
| 2016 | East | 402 | 273 | 548 | 2 | 1 | 2 | 361 | 254 | 475 | 1 | 1 | 2 | 40 | 19 | 72 | 0 | 0 | 0 | 0 | 0 | 1 | 0 | 0 | 0 |
| 2017 | East | 349 | 238 | 476 | 1 | 1 | 2 | 314 | 221 | 413 | 1 | 1 | 2 | 35 | 16 | 62 | 0 | 0 | 0 | 0 | 0 | 0 | 0 | 0 | 0 |
| 2010 | West | 3848 | 2649 | 5178 | 17 | 11 | 22 | 3577 | 2516 | 4701 | 16 | 11 | 20 | 269 | 132 | 474 | 1 | 1 | 2 | 2 | 1 | 4 | 0 | 0 | 0 |
| 2011 | West | 3403 | 2332 | 4559 | 15 | 10 | 20 | 3156 | 2220 | 4147 | 14 | 10 | 19 | 245 | 111 | 409 | 1 | 0 | 2 | 2 | 1 | 3 | 0 | 0 | 0 |
| 2012 | West | 3004 | 2060 | 4032 | 14 | 9 | 18 | 2781 | 1956 | 3655 | 13 | 9 | 16 | 221 | 103 | 374 | 1 | 0 | 2 | 2 | 1 | 3 | 0 | 0 | 0 |
| 2013 | West | 2608 | 1789 | 3506 | 12 | 8 | 16 | 2409 | 1695 | 3166 | 11 | 8 | 14 | 198 | 93 | 338 | 1 | 0 | 2 | 2 | 1 | 3 | 0 | 0 | 0 |
| 2014 | West | 2401 | 1648 | 3233 | 11 | 7 | 14 | 2217 | 1559 | 2913 | 10 | 7 | 13 | 183 | 88 | 318 | 1 | 0 | 1 | 1 | 1 | 3 | 0 | 0 | 0 |
| 2015 | West | 2081 | 1428 | 2803 | 9 | 6 | 12 | 1920 | 1350 | 2522 | 9 | 6 | 11 | 160 | 77 | 279 | 1 | 0 | 1 | 1 | 1 | 2 | 0 | 0 | 0 |
| 2016 | West | 1945 | 1334 | 2622 | 9 | 6 | 12 | 1795 | 1263 | 2359 | 8 | 6 | 10 | 149 | 71 | 262 | 1 | 0 | 1 | 1 | 1 | 2 | 0 | 0 | 0 |
| 2017 | West | 1636 | 1117 | 2191 | 7 | 5 | 9 | 1512 | 1064 | 1987 | 7 | 5 | 9 | 123 | 53 | 202 | 1 | 0 | 1 | 1 | 0 | 2 | 0 | 0 | 0 |
| 2010 | National | 6538 | 4480 | 8841 | 8 | 5 | 11 | 6006 | 4224 | 7891 | 7 | 5 | 10 | 528 | 254 | 943 | 1 | 0 | 1 | 4 | 2 | 8 | 0 | 0 | 0 |
| 2011 | National | 5651 | 3860 | 7625 | 7 | 5 | 10 | 5176 | 3641 | 6801 | 7 | 5 | 9 | 471 | 218 | 817 | 1 | 0 | 1 | 4 | 2 | 7 | 0 | 0 | 0 |
| 2012 | National | 5126 | 3501 | 6925 | 7 | 4 | 9 | 4686 | 3296 | 6157 | 6 | 4 | 8 | 436 | 204 | 762 | 1 | 0 | 1 | 3 | 2 | 6 | 0 | 0 | 0 |
| 2013 | National | 4532 | 3095 | 6133 | 6 | 4 | 8 | 4132 | 2906 | 5429 | 5 | 4 | 7 | 397 | 187 | 698 | 1 | 0 | 1 | 3 | 1 | 6 | 0 | 0 | 0 |
| 2014 | National | 4066 | 2778 | 5509 | 5 | 3 | 7 | 3704 | 2605 | 4867 | 5 | 3 | 6 | 360 | 172 | 637 | 0 | 0 | 1 | 3 | 1 | 5 | 0 | 0 | 0 |
| 2015 | National | 3550 | 2424 | 4811 | 4 | 3 | 6 | 3230 | 2272 | 4244 | 4 | 3 | 5 | 318 | 151 | 563 | 0 | 0 | 1 | 3 | 1 | 4 | 0 | 0 | 0 |
| 2016 | National | 3463 | 2363 | 4696 | 4 | 3 | 6 | 3150 | 2215 | 4138 | 4 | 3 | 5 | 311 | 147 | 554 | 0 | 0 | 1 | 2 | 1 | 4 | 0 | 0 | 0 |
| 2017 | National | 2888 | 1966 | 3900 | 4 | 2 | 5 | 2631 | 1850 | 3457 | 3 | 2 | 4 | 255 | 115 | 440 | 0 | 0 | 1 | 2 | 1 | 4 | 0 | 0 | 0 |

**Webappendix 10: Pneumococcal cases in China by region, province, and year**

| **Year** | **Province** | **Pneumococcal clinical pneumonia cases^1^** | | | | | | **Pneumococcal severe pneumonia cases^1^** | | | | | | **Pneumococcal meningitis cases^2^** | | | | | | **Pneumococcal severe NPNM cases^3^** | | | | | | **Pneumococcal non-severe NPNM cases^3^** | | | | | |
| --- | --- | --- | --- | --- | --- | --- | --- | --- | --- | --- | --- | --- | --- | --- | --- | --- | --- | --- | --- | --- | --- | --- | --- | --- | --- | --- | --- | --- | --- | --- | --- |
|  |  | Number | | | Rate per 100 000 | | | Number | | | Rate per 100 000 | | | Number | | | Rate per 100 000 | | | Number | | | Rate per 100 000 | | | Number | | | Rate per 100 000 | | |
|  |  | Mean | UR | | Mean | UR | | Mean | UR | | Mean | UR | | Mean | UR | | Mean | UR | | Mean | UR | | Mean | UR | | Mean | UR | | Mean | UR | |
| 2010 | Anhui | 28145 | 24285 | 33457 | 754 | 651 | 896 | 10319 | 7731 | 11770 | 276 | 207 | 315 | 734 | 454 | 984 | 20 | 12 | 26 | 833 | 516 | 1118 | 22 | 14 | 30 | 2215 | 1371 | 2972 | 59 | 37 | 80 |
| 2011 | Anhui | 27568 | 23787 | 32771 | 758 | 654 | 901 | 10108 | 7572 | 11529 | 278 | 208 | 317 | 668 | 414 | 897 | 18 | 11 | 25 | 759 | 470 | 1018 | 21 | 13 | 28 | 2018 | 1249 | 2708 | 56 | 34 | 74 |
| 2012 | Anhui | 26880 | 23193 | 31953 | 736 | 635 | 875 | 9855 | 7383 | 11241 | 270 | 202 | 308 | 595 | 368 | 799 | 16 | 10 | 22 | 676 | 418 | 907 | 19 | 11 | 25 | 1797 | 1112 | 2412 | 49 | 30 | 66 |
| 2013 | Anhui | 26365 | 22748 | 31340 | 730 | 629 | 867 | 9666 | 7242 | 11025 | 267 | 200 | 305 | 526 | 325 | 705 | 15 | 9 | 20 | 597 | 369 | 801 | 17 | 10 | 22 | 1587 | 982 | 2129 | 44 | 27 | 59 |
| 2014 | Anhui | 25814 | 22273 | 30686 | 711 | 613 | 845 | 9464 | 7091 | 10795 | 261 | 195 | 297 | 460 | 285 | 617 | 13 | 8 | 17 | 522 | 323 | 701 | 14 | 9 | 19 | 1389 | 860 | 1864 | 38 | 24 | 51 |
| 2015 | Anhui | 24662 | 21279 | 29316 | 682 | 588 | 811 | 9042 | 6774 | 10313 | 250 | 187 | 285 | 404 | 250 | 542 | 11 | 7 | 15 | 459 | 284 | 615 | 13 | 8 | 17 | 1219 | 755 | 1636 | 34 | 21 | 45 |
| 2016 | Anhui | 23969 | 20681 | 28492 | 642 | 554 | 763 | 8788 | 6584 | 10023 | 235 | 176 | 268 | 345 | 214 | 463 | 9 | 6 | 12 | 392 | 243 | 526 | 10 | 6 | 14 | 1042 | 645 | 1398 | 28 | 17 | 37 |
| 2017 | Anhui | 23379 | 20172 | 27791 | 605 | 522 | 719 | 8572 | 6422 | 9777 | 222 | 166 | 253 | 293 | 181 | 393 | 8 | 5 | 10 | 333 | 206 | 446 | 9 | 5 | 12 | 884 | 547 | 1187 | 23 | 14 | 31 |
| 2010 | Beijing | 6142 | 5300 | 7301 | 735 | 634 | 874 | 2252 | 1687 | 2569 | 269 | 202 | 307 | 37 | 23 | 49 | 4 | 3 | 6 | 42 | 26 | 56 | 5 | 3 | 7 | 111 | 69 | 149 | 13 | 8 | 18 |
| 2011 | Beijing | 6376 | 5501 | 7579 | 1070 | 923 | 1272 | 2338 | 1751 | 2666 | 392 | 294 | 447 | 38 | 23 | 51 | 6 | 4 | 8 | 43 | 27 | 58 | 7 | 4 | 10 | 114 | 71 | 153 | 19 | 12 | 26 |
| 2012 | Beijing | 6445 | 5561 | 7661 | 811 | 699 | 964 | 2363 | 1770 | 2695 | 297 | 223 | 339 | 38 | 23 | 51 | 5 | 3 | 6 | 43 | 27 | 58 | 5 | 3 | 7 | 115 | 71 | 154 | 14 | 9 | 19 |
| 2013 | Beijing | 6949 | 5996 | 8260 | 841 | 725 | 999 | 2548 | 1909 | 2906 | 308 | 231 | 352 | 40 | 25 | 54 | 5 | 3 | 7 | 46 | 28 | 61 | 6 | 3 | 7 | 121 | 75 | 163 | 15 | 9 | 20 |
| 2014 | Beijing | 7240 | 6247 | 8606 | 841 | 726 | 1000 | 2654 | 1989 | 3028 | 308 | 231 | 352 | 42 | 26 | 57 | 5 | 3 | 7 | 48 | 30 | 65 | 6 | 3 | 8 | 128 | 79 | 172 | 15 | 9 | 20 |
| 2015 | Beijing | 7365 | 6355 | 8755 | 873 | 754 | 1038 | 2700 | 2023 | 3080 | 320 | 240 | 365 | 44 | 27 | 58 | 5 | 3 | 7 | 49 | 31 | 66 | 6 | 4 | 8 | 132 | 81 | 176 | 16 | 10 | 21 |
| 2016 | Beijing | 7615 | 6570 | 9052 | 874 | 754 | 1039 | 2792 | 2092 | 3184 | 321 | 240 | 366 | 43 | 27 | 58 | 5 | 3 | 7 | 49 | 30 | 66 | 6 | 3 | 8 | 131 | 81 | 175 | 15 | 9 | 20 |
| 2017 | Beijing | 7600 | 6558 | 9035 | 812 | 700 | 965 | 2787 | 2088 | 3178 | 298 | 223 | 339 | 39 | 24 | 53 | 4 | 3 | 6 | 45 | 28 | 60 | 5 | 3 | 6 | 119 | 74 | 160 | 13 | 8 | 17 |
| 2010 | Chongqing | 15008 | 12949 | 17840 | 1024 | 884 | 1217 | 5502 | 4122 | 6276 | 375 | 281 | 428 | 208 | 129 | 280 | 14 | 9 | 19 | 237 | 147 | 318 | 16 | 10 | 22 | 630 | 390 | 845 | 43 | 27 | 58 |
| 2011 | Chongqing | 14531 | 12538 | 17273 | 968 | 835 | 1151 | 5328 | 3991 | 6077 | 355 | 266 | 405 | 197 | 122 | 264 | 13 | 8 | 18 | 223 | 138 | 300 | 15 | 9 | 20 | 594 | 368 | 797 | 40 | 24 | 53 |
| 2012 | Chongqing | 13867 | 11965 | 16484 | 879 | 759 | 1045 | 5084 | 3809 | 5799 | 322 | 242 | 368 | 181 | 112 | 243 | 11 | 7 | 15 | 206 | 127 | 276 | 13 | 8 | 18 | 547 | 339 | 734 | 35 | 21 | 47 |
| 2013 | Chongqing | 13339 | 11509 | 15856 | 863 | 745 | 1026 | 4890 | 3664 | 5578 | 316 | 237 | 361 | 169 | 104 | 226 | 11 | 7 | 15 | 192 | 119 | 257 | 12 | 8 | 17 | 509 | 315 | 683 | 33 | 20 | 44 |
| 2014 | Chongqing | 12826 | 11066 | 15246 | 809 | 698 | 962 | 4702 | 3523 | 5363 | 297 | 222 | 338 | 155 | 96 | 208 | 10 | 6 | 13 | 176 | 109 | 237 | 11 | 7 | 15 | 469 | 290 | 629 | 30 | 18 | 40 |
| 2015 | Chongqing | 12169 | 10499 | 14465 | 768 | 663 | 913 | 4461 | 3343 | 5089 | 282 | 211 | 321 | 139 | 86 | 186 | 9 | 5 | 12 | 158 | 98 | 212 | 10 | 6 | 13 | 419 | 259 | 562 | 26 | 16 | 36 |
| 2016 | Chongqing | 11906 | 10273 | 14153 | 766 | 661 | 910 | 4365 | 3270 | 4979 | 281 | 210 | 320 | 118 | 73 | 158 | 8 | 5 | 10 | 134 | 83 | 180 | 9 | 5 | 12 | 356 | 221 | 478 | 23 | 14 | 31 |
| 2017 | Chongqing | 11714 | 10107 | 13925 | 714 | 616 | 849 | 4295 | 3218 | 4899 | 262 | 196 | 298 | 98 | 61 | 132 | 6 | 4 | 8 | 111 | 69 | 150 | 7 | 4 | 9 | 296 | 183 | 398 | 18 | 11 | 24 |
| 2010 | Fujian | 20111 | 17353 | 23907 | 811 | 700 | 964 | 7374 | 5524 | 8410 | 297 | 223 | 339 | 261 | 162 | 351 | 11 | 7 | 14 | 297 | 184 | 398 | 12 | 7 | 16 | 789 | 488 | 1059 | 32 | 20 | 43 |
| 2011 | Fujian | 19804 | 17087 | 23541 | 940 | 811 | 1117 | 7261 | 5440 | 8282 | 345 | 258 | 393 | 249 | 154 | 335 | 12 | 7 | 16 | 283 | 175 | 380 | 13 | 8 | 18 | 753 | 466 | 1010 | 36 | 22 | 48 |
| 2012 | Fujian | 19198 | 16564 | 22820 | 834 | 719 | 991 | 7038 | 5273 | 8028 | 306 | 229 | 349 | 233 | 144 | 313 | 10 | 6 | 14 | 265 | 164 | 356 | 12 | 7 | 15 | 704 | 436 | 945 | 31 | 19 | 41 |
| 2013 | Fujian | 18643 | 16086 | 22162 | 798 | 689 | 949 | 6835 | 5121 | 7796 | 293 | 219 | 334 | 219 | 135 | 294 | 9 | 6 | 13 | 248 | 154 | 333 | 11 | 7 | 14 | 661 | 409 | 886 | 28 | 18 | 38 |
| 2014 | Fujian | 17914 | 15457 | 21295 | 739 | 638 | 879 | 6568 | 4921 | 7492 | 271 | 203 | 309 | 207 | 128 | 277 | 9 | 5 | 11 | 235 | 145 | 315 | 10 | 6 | 13 | 624 | 386 | 837 | 26 | 16 | 35 |
| 2015 | Fujian | 16695 | 14405 | 19845 | 696 | 600 | 827 | 6121 | 4586 | 6981 | 255 | 191 | 291 | 188 | 116 | 252 | 8 | 5 | 11 | 213 | 132 | 286 | 9 | 6 | 12 | 567 | 351 | 761 | 24 | 15 | 32 |
| 2016 | Fujian | 15749 | 13589 | 18721 | 645 | 557 | 767 | 5774 | 4326 | 6586 | 237 | 177 | 270 | 164 | 102 | 221 | 7 | 4 | 9 | 187 | 116 | 251 | 8 | 5 | 10 | 497 | 307 | 666 | 20 | 13 | 27 |
| 2017 | Fujian | 14825 | 12791 | 17622 | 598 | 516 | 710 | 5435 | 4072 | 6199 | 219 | 164 | 250 | 141 | 87 | 189 | 6 | 4 | 8 | 160 | 99 | 215 | 6 | 4 | 9 | 426 | 264 | 572 | 17 | 11 | 23 |
| 2010 | Gansu | 13695 | 11816 | 16279 | 859 | 741 | 1021 | 5021 | 3762 | 5727 | 315 | 236 | 359 | 431 | 191 | 583 | 27 | 12 | 37 | 489 | 217 | 662 | 31 | 14 | 41 | 1301 | 577 | 1759 | 82 | 36 | 110 |
| 2011 | Gansu | 13386 | 11550 | 15912 | 893 | 770 | 1061 | 4908 | 3677 | 5598 | 327 | 245 | 373 | 471 | 320 | 693 | 31 | 21 | 46 | 535 | 363 | 788 | 36 | 24 | 53 | 1423 | 966 | 2094 | 95 | 64 | 140 |
| 2012 | Gansu | 13003 | 11219 | 15457 | 919 | 793 | 1092 | 4767 | 3572 | 5438 | 337 | 252 | 384 | 481 | 316 | 685 | 34 | 22 | 48 | 547 | 359 | 778 | 39 | 25 | 55 | 1454 | 954 | 2067 | 103 | 67 | 146 |
| 2013 | Gansu | 12681 | 10941 | 15074 | 900 | 776 | 1070 | 4649 | 3483 | 5303 | 330 | 247 | 376 | 480 | 306 | 663 | 34 | 22 | 47 | 545 | 347 | 753 | 39 | 25 | 53 | 1450 | 924 | 2003 | 103 | 66 | 142 |
| 2014 | Gansu | 12328 | 10637 | 14655 | 839 | 724 | 997 | 4520 | 3386 | 5155 | 308 | 230 | 351 | 481 | 298 | 645 | 33 | 20 | 44 | 546 | 338 | 733 | 37 | 23 | 50 | 1453 | 899 | 1949 | 99 | 61 | 133 |
| 2015 | Gansu | 11661 | 10061 | 13862 | 799 | 689 | 950 | 4275 | 3203 | 4876 | 293 | 219 | 334 | 428 | 265 | 574 | 29 | 18 | 39 | 486 | 301 | 652 | 33 | 21 | 45 | 1293 | 800 | 1734 | 89 | 55 | 119 |
| 2016 | Gansu | 11270 | 9724 | 13397 | 769 | 663 | 914 | 4132 | 3096 | 4713 | 282 | 211 | 321 | 368 | 227 | 493 | 25 | 16 | 34 | 417 | 258 | 560 | 28 | 18 | 38 | 1110 | 687 | 1489 | 76 | 47 | 102 |
| 2017 | Gansu | 10968 | 9463 | 13038 | 745 | 643 | 885 | 4021 | 3013 | 4587 | 273 | 205 | 311 | 310 | 192 | 417 | 21 | 13 | 28 | 353 | 218 | 473 | 24 | 15 | 32 | 937 | 580 | 1258 | 64 | 39 | 85 |
| 2010 | Guangdong | 79895 | 68935 | 94972 | 1402 | 1209 | 1666 | 29292 | 21946 | 33411 | 514 | 385 | 586 | 812 | 503 | 1090 | 14 | 9 | 19 | 923 | 571 | 1238 | 16 | 10 | 22 | 2453 | 1518 | 3292 | 43 | 27 | 58 |
| 2011 | Guangdong | 78296 | 67555 | 93072 | 1465 | 1264 | 1741 | 28706 | 21506 | 32742 | 537 | 402 | 613 | 780 | 483 | 1046 | 15 | 9 | 20 | 886 | 548 | 1188 | 17 | 10 | 22 | 2355 | 1457 | 3160 | 44 | 27 | 59 |
| 2012 | Guangdong | 75702 | 65317 | 89988 | 1160 | 1001 | 1379 | 27755 | 20794 | 31657 | 425 | 319 | 485 | 742 | 459 | 995 | 11 | 7 | 15 | 842 | 521 | 1130 | 13 | 8 | 17 | 2239 | 1386 | 3004 | 34 | 21 | 46 |
| 2013 | Guangdong | 74295 | 64104 | 88316 | 1137 | 981 | 1352 | 27239 | 20407 | 31069 | 417 | 312 | 476 | 718 | 445 | 964 | 11 | 7 | 15 | 816 | 505 | 1095 | 12 | 8 | 17 | 2169 | 1342 | 2910 | 33 | 21 | 45 |
| 2014 | Guangdong | 72496 | 62551 | 86177 | 1064 | 918 | 1265 | 26580 | 19913 | 30317 | 390 | 292 | 445 | 699 | 432 | 938 | 10 | 6 | 14 | 794 | 491 | 1065 | 12 | 7 | 16 | 2110 | 1306 | 2831 | 31 | 19 | 42 |
| 2015 | Guangdong | 69471 | 59941 | 82581 | 1024 | 884 | 1217 | 25470 | 19082 | 29051 | 375 | 281 | 428 | 664 | 411 | 891 | 10 | 6 | 13 | 754 | 467 | 1011 | 11 | 7 | 15 | 2004 | 1240 | 2689 | 30 | 18 | 40 |
| 2016 | Guangdong | 67816 | 58513 | 80614 | 992 | 856 | 1179 | 24864 | 18628 | 28359 | 364 | 272 | 415 | 608 | 376 | 815 | 9 | 5 | 12 | 690 | 427 | 926 | 10 | 6 | 14 | 1835 | 1135 | 2461 | 27 | 17 | 36 |
| 2017 | Guangdong | 66523 | 57398 | 79077 | 885 | 764 | 1052 | 24390 | 18273 | 27819 | 324 | 243 | 370 | 542 | 335 | 727 | 7 | 4 | 10 | 615 | 381 | 826 | 8 | 5 | 11 | 1636 | 1013 | 2195 | 22 | 13 | 29 |
| 2010 | Guangxi | 25262 | 21797 | 30030 | 716 | 618 | 852 | 9262 | 6939 | 10564 | 263 | 197 | 300 | 382 | 237 | 513 | 11 | 7 | 15 | 434 | 269 | 582 | 12 | 8 | 17 | 1154 | 714 | 1548 | 33 | 20 | 44 |
| 2011 | Guangxi | 25250 | 21786 | 30015 | 697 | 601 | 829 | 9257 | 6936 | 10559 | 256 | 191 | 292 | 372 | 230 | 499 | 10 | 6 | 14 | 423 | 262 | 567 | 12 | 7 | 16 | 1124 | 695 | 1508 | 31 | 19 | 42 |
| 2012 | Guangxi | 25121 | 21675 | 29862 | 686 | 592 | 816 | 9210 | 6900 | 10505 | 252 | 189 | 287 | 354 | 219 | 476 | 10 | 6 | 13 | 403 | 249 | 540 | 11 | 7 | 15 | 1070 | 662 | 1436 | 29 | 18 | 39 |
| 2013 | Guangxi | 25177 | 21723 | 29928 | 661 | 571 | 786 | 9231 | 6916 | 10528 | 242 | 182 | 277 | 335 | 207 | 449 | 9 | 5 | 12 | 380 | 235 | 510 | 10 | 6 | 13 | 1011 | 626 | 1357 | 27 | 16 | 36 |
| 2014 | Guangxi | 25279 | 21811 | 30050 | 649 | 560 | 772 | 9268 | 6944 | 10571 | 238 | 178 | 271 | 323 | 200 | 434 | 8 | 5 | 11 | 367 | 227 | 493 | 9 | 6 | 13 | 977 | 605 | 1311 | 25 | 16 | 34 |
| 2015 | Guangxi | 24804 | 21401 | 29485 | 644 | 556 | 766 | 9094 | 6813 | 10373 | 236 | 177 | 269 | 301 | 186 | 404 | 8 | 5 | 10 | 342 | 211 | 458 | 9 | 5 | 12 | 908 | 562 | 1219 | 24 | 15 | 32 |
| 2016 | Guangxi | 24557 | 21188 | 29192 | 628 | 542 | 746 | 9003 | 6745 | 10269 | 230 | 172 | 262 | 270 | 167 | 363 | 7 | 4 | 9 | 307 | 190 | 412 | 8 | 5 | 11 | 817 | 505 | 1096 | 21 | 13 | 28 |
| 2017 | Guangxi | 24189 | 20870 | 28753 | 601 | 518 | 714 | 8868 | 6644 | 10115 | 220 | 165 | 251 | 238 | 147 | 319 | 6 | 4 | 8 | 270 | 167 | 362 | 7 | 4 | 9 | 717 | 444 | 962 | 18 | 11 | 24 |
| 2010 | Guizhou | 19462 | 16792 | 23135 | 792 | 683 | 941 | 7135 | 5346 | 8139 | 290 | 217 | 331 | 373 | 231 | 501 | 15 | 9 | 20 | 424 | 262 | 569 | 17 | 11 | 23 | 1127 | 698 | 1513 | 46 | 28 | 62 |
| 2011 | Guizhou | 18808 | 16228 | 22357 | 736 | 635 | 875 | 6896 | 5166 | 7865 | 270 | 202 | 308 | 336 | 208 | 451 | 13 | 8 | 18 | 382 | 236 | 513 | 15 | 9 | 20 | 1016 | 629 | 1363 | 40 | 25 | 53 |
| 2012 | Guizhou | 18278 | 15770 | 21727 | 726 | 627 | 863 | 6701 | 5021 | 7643 | 266 | 199 | 304 | 298 | 184 | 399 | 12 | 7 | 16 | 338 | 209 | 454 | 13 | 8 | 18 | 899 | 556 | 1206 | 36 | 22 | 48 |
| 2013 | Guizhou | 17970 | 15505 | 21362 | 710 | 612 | 843 | 6588 | 4936 | 7515 | 260 | 195 | 297 | 264 | 163 | 354 | 10 | 6 | 14 | 300 | 186 | 402 | 12 | 7 | 16 | 797 | 493 | 1070 | 31 | 19 | 42 |
| 2014 | Guizhou | 17705 | 15276 | 21046 | 685 | 591 | 814 | 6491 | 4863 | 7404 | 251 | 188 | 286 | 232 | 144 | 312 | 9 | 6 | 12 | 264 | 163 | 354 | 10 | 6 | 14 | 702 | 434 | 942 | 27 | 17 | 36 |
| 2015 | Guizhou | 17053 | 14714 | 20271 | 665 | 574 | 791 | 6252 | 4684 | 7131 | 244 | 183 | 278 | 198 | 123 | 266 | 8 | 5 | 10 | 225 | 140 | 302 | 9 | 5 | 12 | 599 | 371 | 804 | 23 | 14 | 31 |
| 2016 | Guizhou | 16545 | 14275 | 19667 | 659 | 569 | 784 | 6066 | 4545 | 6919 | 242 | 181 | 276 | 165 | 102 | 221 | 7 | 4 | 9 | 187 | 116 | 252 | 7 | 5 | 10 | 498 | 308 | 669 | 20 | 12 | 27 |
| 2017 | Guizhou | 15883 | 13704 | 18880 | 562 | 485 | 668 | 5823 | 4363 | 6642 | 206 | 154 | 235 | 137 | 85 | 184 | 5 | 3 | 7 | 156 | 96 | 209 | 6 | 3 | 7 | 414 | 256 | 555 | 15 | 9 | 20 |
| 2010 | Hainan | 5550 | 4789 | 6597 | 867 | 748 | 1031 | 2035 | 1524 | 2321 | 318 | 238 | 363 | 128 | 79 | 172 | 20 | 12 | 27 | 145 | 90 | 195 | 23 | 14 | 30 | 387 | 239 | 519 | 60 | 37 | 81 |
| 2011 | Hainan | 5460 | 4711 | 6491 | 881 | 760 | 1047 | 2002 | 1500 | 2283 | 323 | 242 | 368 | 124 | 77 | 167 | 20 | 12 | 27 | 141 | 87 | 189 | 23 | 14 | 31 | 375 | 232 | 503 | 60 | 37 | 81 |
| 2012 | Hainan | 5308 | 4580 | 6310 | 824 | 711 | 979 | 1946 | 1458 | 2220 | 302 | 226 | 344 | 119 | 73 | 159 | 18 | 11 | 25 | 135 | 83 | 181 | 21 | 13 | 28 | 359 | 222 | 481 | 56 | 34 | 75 |
| 2013 | Hainan | 5176 | 4466 | 6153 | 800 | 690 | 951 | 1898 | 1422 | 2165 | 293 | 220 | 335 | 115 | 71 | 155 | 18 | 11 | 24 | 131 | 81 | 176 | 20 | 13 | 27 | 349 | 216 | 468 | 54 | 33 | 72 |
| 2014 | Hainan | 5018 | 4330 | 5965 | 769 | 664 | 914 | 1840 | 1378 | 2098 | 282 | 211 | 322 | 111 | 69 | 149 | 17 | 11 | 23 | 127 | 78 | 170 | 19 | 12 | 26 | 336 | 208 | 451 | 52 | 32 | 69 |
| 2015 | Hainan | 4729 | 4080 | 5622 | 733 | 632 | 871 | 1734 | 1299 | 1978 | 269 | 201 | 306 | 105 | 65 | 142 | 16 | 10 | 22 | 120 | 74 | 161 | 19 | 11 | 25 | 319 | 197 | 427 | 49 | 31 | 66 |
| 2016 | Hainan | 4559 | 3934 | 5420 | 703 | 607 | 836 | 1672 | 1252 | 1907 | 258 | 193 | 294 | 98 | 60 | 131 | 15 | 9 | 20 | 111 | 69 | 149 | 17 | 11 | 23 | 295 | 182 | 396 | 45 | 28 | 61 |
| 2017 | Hainan | 4410 | 3805 | 5243 | 669 | 577 | 795 | 1617 | 1211 | 1844 | 245 | 184 | 280 | 87 | 54 | 117 | 13 | 8 | 18 | 99 | 61 | 133 | 15 | 9 | 20 | 263 | 163 | 353 | 40 | 25 | 54 |
| 2010 | Hebei | 38470 | 33192 | 45730 | 764 | 659 | 908 | 14104 | 10567 | 16087 | 280 | 210 | 319 | 1253 | 775 | 1681 | 25 | 15 | 33 | 1423 | 880 | 1909 | 28 | 17 | 38 | 3782 | 2341 | 5075 | 75 | 46 | 101 |
| 2011 | Hebei | 38454 | 33179 | 45711 | 800 | 690 | 951 | 14099 | 10563 | 16081 | 293 | 220 | 334 | 1219 | 754 | 1635 | 25 | 16 | 34 | 1384 | 857 | 1857 | 29 | 18 | 39 | 3681 | 2278 | 4938 | 77 | 47 | 103 |
| 2012 | Hebei | 38090 | 32865 | 45278 | 765 | 660 | 909 | 13965 | 10463 | 15928 | 280 | 210 | 320 | 1162 | 719 | 1559 | 23 | 14 | 31 | 1320 | 817 | 1771 | 26 | 16 | 36 | 3508 | 2171 | 4707 | 70 | 44 | 94 |
| 2013 | Hebei | 37787 | 32604 | 44918 | 741 | 639 | 881 | 13854 | 10379 | 15802 | 272 | 204 | 310 | 1103 | 683 | 1480 | 22 | 13 | 29 | 1253 | 775 | 1681 | 25 | 15 | 33 | 3330 | 2061 | 4468 | 65 | 40 | 88 |
| 2014 | Hebei | 37317 | 32198 | 44360 | 716 | 618 | 851 | 13682 | 10250 | 15605 | 263 | 197 | 300 | 1068 | 661 | 1433 | 21 | 13 | 28 | 1213 | 751 | 1628 | 23 | 14 | 31 | 3225 | 1996 | 4327 | 62 | 38 | 83 |
| 2015 | Hebei | 35938 | 31008 | 42720 | 712 | 615 | 847 | 13176 | 9871 | 15028 | 261 | 196 | 298 | 1011 | 626 | 1356 | 20 | 12 | 27 | 1148 | 710 | 1540 | 23 | 14 | 31 | 3052 | 1889 | 4095 | 60 | 37 | 81 |
| 2016 | Hebei | 35134 | 30314 | 41764 | 700 | 604 | 833 | 12881 | 9651 | 14692 | 257 | 192 | 293 | 929 | 575 | 1247 | 19 | 11 | 25 | 1055 | 653 | 1416 | 21 | 13 | 28 | 2805 | 1736 | 3764 | 56 | 35 | 75 |
| 2017 | Hebei | 34605 | 29858 | 41135 | 669 | 577 | 795 | 12687 | 9505 | 14471 | 245 | 184 | 280 | 831 | 515 | 1116 | 16 | 10 | 22 | 944 | 584 | 1267 | 18 | 11 | 24 | 2511 | 1554 | 3369 | 49 | 30 | 65 |
| 2010 | Heilongjiang | 11805 | 10186 | 14033 | 580 | 501 | 690 | 4328 | 3243 | 4937 | 213 | 159 | 243 | 281 | 174 | 376 | 14 | 9 | 18 | 319 | 197 | 427 | 16 | 10 | 21 | 847 | 524 | 1136 | 42 | 26 | 56 |
| 2011 | Heilongjiang | 11423 | 9856 | 13579 | 714 | 616 | 849 | 4188 | 3138 | 4777 | 262 | 196 | 299 | 268 | 166 | 360 | 17 | 10 | 22 | 304 | 188 | 408 | 19 | 12 | 26 | 809 | 501 | 1086 | 51 | 31 | 68 |
| 2012 | Heilongjiang | 10975 | 9469 | 13046 | 814 | 702 | 967 | 4024 | 3015 | 4590 | 298 | 223 | 340 | 246 | 152 | 330 | 18 | 11 | 24 | 279 | 173 | 375 | 21 | 13 | 28 | 743 | 460 | 997 | 55 | 34 | 74 |
| 2013 | Heilongjiang | 10612 | 9157 | 12615 | 817 | 705 | 971 | 3891 | 2915 | 4438 | 299 | 224 | 342 | 225 | 139 | 302 | 17 | 11 | 23 | 256 | 158 | 343 | 20 | 12 | 26 | 680 | 421 | 912 | 52 | 32 | 70 |
| 2014 | Heilongjiang | 10238 | 8834 | 12170 | 775 | 669 | 921 | 3754 | 2812 | 4281 | 284 | 213 | 324 | 209 | 129 | 280 | 16 | 10 | 21 | 237 | 147 | 318 | 18 | 11 | 24 | 630 | 390 | 845 | 48 | 30 | 64 |
| 2015 | Heilongjiang | 9717 | 8384 | 11551 | 752 | 649 | 894 | 3563 | 2669 | 4064 | 276 | 207 | 315 | 187 | 115 | 250 | 14 | 9 | 19 | 212 | 131 | 284 | 16 | 10 | 22 | 564 | 349 | 756 | 44 | 27 | 59 |
| 2016 | Heilongjiang | 9407 | 8117 | 11182 | 739 | 638 | 878 | 3449 | 2584 | 3934 | 271 | 203 | 309 | 164 | 102 | 220 | 13 | 8 | 17 | 187 | 115 | 250 | 15 | 9 | 20 | 496 | 307 | 665 | 39 | 24 | 52 |
| 2017 | Heilongjiang | 9173 | 7914 | 10904 | 835 | 720 | 992 | 3363 | 2520 | 3836 | 306 | 229 | 349 | 144 | 89 | 193 | 13 | 8 | 18 | 163 | 101 | 219 | 15 | 9 | 20 | 434 | 269 | 583 | 40 | 24 | 53 |
| 2010 | Henan | 34275 | 29573 | 40743 | 523 | 451 | 622 | 12566 | 9415 | 14333 | 192 | 144 | 219 | 1049 | 649 | 1407 | 16 | 10 | 21 | 1191 | 737 | 1598 | 18 | 11 | 24 | 3166 | 1959 | 4248 | 48 | 30 | 65 |
| 2011 | Henan | 34639 | 29888 | 41177 | 478 | 413 | 569 | 12700 | 9515 | 14486 | 175 | 131 | 200 | 1010 | 625 | 1356 | 14 | 9 | 19 | 1148 | 710 | 1540 | 16 | 10 | 21 | 3051 | 1888 | 4094 | 42 | 26 | 57 |
| 2012 | Henan | 35105 | 30289 | 41729 | 473 | 408 | 562 | 12871 | 9643 | 14680 | 173 | 130 | 198 | 956 | 592 | 1283 | 13 | 8 | 17 | 1086 | 672 | 1457 | 15 | 9 | 20 | 2888 | 1787 | 3874 | 39 | 24 | 52 |
| 2013 | Henan | 35974 | 31039 | 42762 | 483 | 417 | 574 | 13189 | 9881 | 15043 | 177 | 133 | 202 | 906 | 561 | 1216 | 12 | 8 | 16 | 1029 | 637 | 1381 | 14 | 9 | 19 | 2737 | 1694 | 3672 | 37 | 23 | 49 |
| 2014 | Henan | 36763 | 31720 | 43701 | 498 | 430 | 592 | 13478 | 10098 | 15373 | 183 | 137 | 208 | 863 | 534 | 1158 | 12 | 7 | 16 | 980 | 607 | 1315 | 13 | 8 | 18 | 2606 | 1613 | 3497 | 35 | 22 | 47 |
| 2015 | Henan | 36477 | 31473 | 43361 | 497 | 429 | 591 | 13374 | 10020 | 15254 | 182 | 137 | 208 | 791 | 490 | 1062 | 11 | 7 | 14 | 899 | 556 | 1206 | 12 | 8 | 16 | 2390 | 1479 | 3207 | 33 | 20 | 44 |
| 2016 | Henan | 36710 | 31674 | 43638 | 497 | 429 | 591 | 13459 | 10084 | 15351 | 182 | 137 | 208 | 697 | 431 | 935 | 9 | 6 | 13 | 791 | 490 | 1062 | 11 | 7 | 14 | 2104 | 1302 | 2822 | 28 | 18 | 38 |
| 2017 | Henan | 36801 | 31753 | 43746 | 496 | 428 | 590 | 13493 | 10109 | 15390 | 182 | 136 | 207 | 604 | 374 | 810 | 8 | 5 | 11 | 686 | 424 | 920 | 9 | 6 | 12 | 1823 | 1128 | 2445 | 25 | 15 | 33 |
| 2010 | Hubei | 15976 | 13784 | 18990 | 450 | 388 | 535 | 5857 | 4388 | 6681 | 165 | 124 | 188 | 223 | 138 | 299 | 6 | 4 | 8 | 253 | 157 | 340 | 7 | 4 | 10 | 673 | 417 | 904 | 19 | 12 | 25 |
| 2011 | Hubei | 15995 | 13801 | 19014 | 520 | 449 | 618 | 5864 | 4394 | 6689 | 191 | 143 | 217 | 215 | 133 | 289 | 7 | 4 | 9 | 245 | 151 | 328 | 8 | 5 | 11 | 651 | 403 | 873 | 21 | 13 | 28 |
| 2012 | Hubei | 16138 | 13924 | 19183 | 538 | 464 | 639 | 5917 | 4433 | 6748 | 197 | 148 | 225 | 202 | 125 | 272 | 7 | 4 | 9 | 230 | 142 | 309 | 8 | 5 | 10 | 611 | 378 | 820 | 20 | 13 | 27 |
| 2013 | Hubei | 16575 | 14301 | 19702 | 544 | 470 | 647 | 6077 | 4553 | 6931 | 200 | 150 | 228 | 189 | 117 | 254 | 6 | 4 | 8 | 215 | 133 | 289 | 7 | 4 | 9 | 572 | 354 | 767 | 19 | 12 | 25 |
| 2014 | Hubei | 17115 | 14767 | 20345 | 549 | 474 | 653 | 6275 | 4701 | 7157 | 201 | 151 | 230 | 177 | 110 | 238 | 6 | 4 | 8 | 201 | 125 | 270 | 6 | 4 | 9 | 535 | 331 | 718 | 17 | 11 | 23 |
| 2015 | Hubei | 17295 | 14923 | 20559 | 558 | 481 | 663 | 6341 | 4751 | 7233 | 204 | 153 | 233 | 160 | 99 | 215 | 5 | 3 | 7 | 182 | 113 | 244 | 6 | 4 | 8 | 484 | 300 | 650 | 16 | 10 | 21 |
| 2016 | Hubei | 17809 | 15366 | 21170 | 582 | 502 | 692 | 6530 | 4892 | 7448 | 213 | 160 | 243 | 141 | 87 | 189 | 5 | 3 | 6 | 160 | 99 | 215 | 5 | 3 | 7 | 425 | 263 | 571 | 14 | 9 | 19 |
| 2017 | Hubei | 18328 | 15813 | 21786 | 568 | 490 | 676 | 6720 | 5034 | 7664 | 208 | 156 | 238 | 122 | 76 | 164 | 4 | 2 | 5 | 139 | 86 | 186 | 4 | 3 | 6 | 369 | 228 | 494 | 11 | 7 | 15 |
| 2010 | Hunan | 22022 | 19001 | 26178 | 536 | 462 | 637 | 8074 | 6049 | 9209 | 196 | 147 | 224 | 249 | 154 | 334 | 6 | 4 | 8 | 283 | 175 | 380 | 7 | 4 | 9 | 753 | 466 | 1010 | 18 | 11 | 25 |
| 2011 | Hunan | 22094 | 19063 | 26264 | 524 | 452 | 623 | 8100 | 6069 | 9239 | 192 | 144 | 219 | 235 | 145 | 315 | 6 | 3 | 7 | 267 | 165 | 358 | 6 | 4 | 8 | 709 | 439 | 952 | 17 | 10 | 23 |
| 2012 | Hunan | 22153 | 19114 | 26334 | 535 | 462 | 636 | 8122 | 6085 | 9264 | 196 | 147 | 224 | 216 | 134 | 290 | 5 | 3 | 7 | 245 | 152 | 329 | 6 | 4 | 8 | 652 | 403 | 875 | 16 | 10 | 21 |
| 2013 | Hunan | 22423 | 19347 | 26654 | 534 | 461 | 634 | 8221 | 6159 | 9377 | 196 | 147 | 223 | 197 | 122 | 264 | 5 | 3 | 6 | 224 | 138 | 300 | 5 | 3 | 7 | 595 | 368 | 798 | 14 | 9 | 19 |
| 2014 | Hunan | 22736 | 19617 | 27026 | 530 | 458 | 631 | 8336 | 6245 | 9508 | 194 | 146 | 222 | 182 | 113 | 245 | 4 | 3 | 6 | 207 | 128 | 278 | 5 | 3 | 6 | 550 | 341 | 738 | 13 | 8 | 17 |
| 2015 | Hunan | 22606 | 19505 | 26872 | 526 | 454 | 625 | 8288 | 6209 | 9453 | 193 | 144 | 220 | 162 | 100 | 218 | 4 | 2 | 5 | 184 | 114 | 247 | 4 | 3 | 6 | 490 | 303 | 657 | 11 | 7 | 15 |
| 2016 | Hunan | 22841 | 19708 | 27151 | 531 | 458 | 631 | 8374 | 6274 | 9552 | 195 | 146 | 222 | 139 | 86 | 187 | 3 | 2 | 4 | 158 | 98 | 212 | 4 | 2 | 5 | 421 | 260 | 565 | 10 | 6 | 13 |
| 2017 | Hunan | 23108 | 19938 | 27469 | 521 | 449 | 619 | 8472 | 6347 | 9663 | 191 | 143 | 218 | 119 | 74 | 160 | 3 | 2 | 4 | 135 | 84 | 181 | 3 | 2 | 4 | 359 | 222 | 482 | 8 | 5 | 11 |
| 2010 | Inner Mongolia | 11398 | 9834 | 13549 | 798 | 689 | 949 | 4179 | 3131 | 4766 | 293 | 219 | 334 | 305 | 189 | 409 | 21 | 13 | 29 | 347 | 215 | 465 | 24 | 15 | 33 | 922 | 570 | 1237 | 65 | 40 | 87 |
| 2011 | Inner Mongolia | 11083 | 9563 | 13174 | 920 | 793 | 1093 | 4063 | 3044 | 4635 | 337 | 253 | 385 | 293 | 181 | 393 | 24 | 15 | 33 | 333 | 206 | 446 | 28 | 17 | 37 | 885 | 547 | 1187 | 73 | 45 | 98 |
| 2012 | Inner Mongolia | 10686 | 9220 | 12703 | 955 | 824 | 1135 | 3918 | 2935 | 4469 | 350 | 262 | 399 | 278 | 172 | 373 | 25 | 15 | 33 | 315 | 195 | 423 | 28 | 17 | 38 | 838 | 519 | 1125 | 75 | 46 | 101 |
| 2013 | Inner Mongolia | 10350 | 8930 | 12303 | 926 | 799 | 1101 | 3795 | 2843 | 4328 | 340 | 254 | 387 | 265 | 164 | 356 | 24 | 15 | 32 | 301 | 186 | 404 | 27 | 17 | 36 | 800 | 495 | 1074 | 72 | 44 | 96 |
| 2014 | Inner Mongolia | 9996 | 8625 | 11883 | 876 | 756 | 1041 | 3665 | 2746 | 4180 | 321 | 241 | 366 | 255 | 158 | 342 | 22 | 14 | 30 | 289 | 179 | 388 | 25 | 16 | 34 | 769 | 476 | 1032 | 67 | 42 | 90 |
| 2015 | Inner Mongolia | 9438 | 8143 | 11219 | 840 | 725 | 999 | 3460 | 2592 | 3947 | 308 | 231 | 351 | 239 | 148 | 320 | 21 | 13 | 29 | 271 | 168 | 364 | 24 | 15 | 32 | 721 | 446 | 967 | 64 | 40 | 86 |
| 2016 | Inner Mongolia | 9060 | 7817 | 10770 | 811 | 700 | 965 | 3322 | 2489 | 3789 | 298 | 223 | 339 | 216 | 134 | 290 | 19 | 12 | 26 | 246 | 152 | 330 | 22 | 14 | 30 | 653 | 404 | 876 | 58 | 36 | 78 |
| 2017 | Inner Mongolia | 8772 | 7569 | 10427 | 835 | 721 | 993 | 3216 | 2409 | 3668 | 306 | 229 | 349 | 193 | 119 | 258 | 18 | 11 | 25 | 219 | 135 | 293 | 21 | 13 | 28 | 582 | 360 | 780 | 55 | 34 | 74 |
| 2010 | Jiangsu | 20631 | 17801 | 24524 | 463 | 399 | 550 | 7564 | 5667 | 8627 | 170 | 127 | 194 | 193 | 119 | 259 | 4 | 3 | 6 | 219 | 136 | 294 | 5 | 3 | 7 | 583 | 361 | 782 | 13 | 8 | 18 |
| 2011 | Jiangsu | 20855 | 17994 | 24791 | 538 | 465 | 640 | 7646 | 5729 | 8721 | 197 | 148 | 225 | 185 | 114 | 248 | 5 | 3 | 6 | 210 | 130 | 281 | 5 | 3 | 7 | 557 | 345 | 748 | 14 | 9 | 19 |
| 2012 | Jiangsu | 21086 | 18193 | 25065 | 533 | 460 | 633 | 7731 | 5792 | 8818 | 195 | 146 | 223 | 173 | 107 | 232 | 4 | 3 | 6 | 197 | 122 | 264 | 5 | 3 | 7 | 523 | 324 | 702 | 13 | 8 | 18 |
| 2013 | Jiangsu | 21725 | 18745 | 25825 | 555 | 479 | 660 | 7965 | 5967 | 9085 | 204 | 152 | 232 | 163 | 101 | 219 | 4 | 3 | 6 | 185 | 115 | 249 | 5 | 3 | 6 | 493 | 305 | 661 | 13 | 8 | 17 |
| 2014 | Jiangsu | 22365 | 19297 | 26586 | 566 | 488 | 673 | 8200 | 6143 | 9353 | 207 | 155 | 237 | 155 | 96 | 207 | 4 | 2 | 5 | 176 | 109 | 236 | 4 | 3 | 6 | 467 | 289 | 626 | 12 | 7 | 16 |
| 2015 | Jiangsu | 22574 | 19478 | 26835 | 577 | 497 | 685 | 8277 | 6201 | 9440 | 211 | 158 | 241 | 142 | 88 | 191 | 4 | 2 | 5 | 161 | 100 | 216 | 4 | 3 | 6 | 429 | 265 | 575 | 11 | 7 | 15 |
| 2016 | Jiangsu | 23149 | 19974 | 27518 | 591 | 510 | 703 | 8487 | 6359 | 9681 | 217 | 162 | 247 | 124 | 77 | 167 | 3 | 2 | 4 | 141 | 87 | 189 | 4 | 2 | 5 | 375 | 232 | 504 | 10 | 6 | 13 |
| 2017 | Jiangsu | 23698 | 20447 | 28170 | 619 | 534 | 736 | 8688 | 6509 | 9910 | 227 | 170 | 259 | 107 | 66 | 144 | 3 | 2 | 4 | 122 | 75 | 163 | 3 | 2 | 4 | 323 | 200 | 434 | 8 | 5 | 11 |
| 2010 | Jiangxi | 28489 | 24581 | 33866 | 945 | 815 | 1123 | 10445 | 7825 | 11914 | 346 | 259 | 395 | 877 | 543 | 1177 | 29 | 18 | 39 | 997 | 617 | 1337 | 33 | 20 | 44 | 2649 | 1640 | 3555 | 88 | 54 | 118 |
| 2011 | Jiangxi | 27500 | 23728 | 32690 | 847 | 730 | 1006 | 10082 | 7554 | 11500 | 310 | 233 | 354 | 813 | 503 | 1091 | 25 | 15 | 34 | 924 | 572 | 1240 | 28 | 18 | 38 | 2456 | 1520 | 3296 | 76 | 47 | 101 |
| 2012 | Jiangxi | 26237 | 22638 | 31189 | 762 | 657 | 905 | 9619 | 7207 | 10972 | 279 | 209 | 319 | 740 | 458 | 994 | 21 | 13 | 29 | 841 | 520 | 1128 | 24 | 15 | 33 | 2236 | 1384 | 3000 | 65 | 40 | 87 |
| 2013 | Jiangxi | 24999 | 21570 | 29717 | 736 | 635 | 875 | 9165 | 6867 | 10454 | 270 | 202 | 308 | 685 | 424 | 919 | 20 | 12 | 27 | 778 | 482 | 1044 | 23 | 14 | 31 | 2069 | 1281 | 2776 | 61 | 38 | 82 |
| 2014 | Jiangxi | 23665 | 20418 | 28131 | 707 | 610 | 840 | 8676 | 6500 | 9896 | 259 | 194 | 295 | 645 | 399 | 865 | 19 | 12 | 26 | 732 | 453 | 982 | 22 | 14 | 29 | 1946 | 1204 | 2611 | 58 | 36 | 78 |
| 2015 | Jiangxi | 21743 | 18760 | 25846 | 655 | 565 | 778 | 7972 | 5972 | 9092 | 240 | 180 | 274 | 587 | 363 | 787 | 18 | 11 | 24 | 666 | 412 | 894 | 20 | 12 | 27 | 1771 | 1096 | 2377 | 53 | 33 | 72 |
| 2016 | Jiangxi | 20325 | 17537 | 24161 | 617 | 533 | 734 | 7452 | 5583 | 8500 | 226 | 170 | 258 | 525 | 325 | 704 | 16 | 10 | 21 | 596 | 369 | 800 | 18 | 11 | 24 | 1585 | 981 | 2127 | 48 | 30 | 65 |
| 2017 | Jiangxi | 19089 | 16471 | 22692 | 611 | 527 | 727 | 6999 | 5243 | 7983 | 224 | 168 | 256 | 458 | 284 | 615 | 15 | 9 | 20 | 521 | 322 | 699 | 17 | 10 | 22 | 1384 | 857 | 1857 | 44 | 27 | 59 |
| 2010 | Jilin | 11847 | 10222 | 14083 | 809 | 698 | 962 | 4344 | 3254 | 4954 | 297 | 222 | 339 | 286 | 177 | 383 | 20 | 12 | 26 | 325 | 201 | 435 | 22 | 14 | 30 | 863 | 534 | 1158 | 59 | 36 | 79 |
| 2011 | Jilin | 11464 | 9891 | 13627 | 964 | 832 | 1146 | 4203 | 3149 | 4794 | 354 | 265 | 403 | 273 | 169 | 366 | 23 | 14 | 31 | 310 | 192 | 416 | 26 | 16 | 35 | 824 | 510 | 1106 | 69 | 43 | 93 |
| 2012 | Jilin | 11007 | 9497 | 13084 | 1041 | 898 | 1238 | 4035 | 3023 | 4603 | 382 | 286 | 435 | 256 | 158 | 343 | 24 | 15 | 32 | 291 | 180 | 390 | 27 | 17 | 37 | 773 | 478 | 1037 | 73 | 45 | 98 |
| 2013 | Jilin | 10619 | 9163 | 12623 | 1020 | 880 | 1212 | 3893 | 2917 | 4441 | 374 | 280 | 427 | 243 | 150 | 326 | 23 | 14 | 31 | 276 | 171 | 370 | 26 | 16 | 36 | 733 | 454 | 984 | 70 | 44 | 94 |
| 2014 | Jilin | 10204 | 8804 | 12129 | 980 | 846 | 1165 | 3741 | 2803 | 4267 | 359 | 269 | 410 | 232 | 144 | 312 | 22 | 14 | 30 | 264 | 163 | 354 | 25 | 16 | 34 | 701 | 434 | 941 | 67 | 42 | 90 |
| 2015 | Jilin | 9582 | 8268 | 11391 | 944 | 814 | 1122 | 3513 | 2632 | 4007 | 346 | 259 | 395 | 215 | 133 | 288 | 21 | 13 | 28 | 244 | 151 | 327 | 24 | 15 | 32 | 648 | 401 | 869 | 64 | 39 | 86 |
| 2016 | Jilin | 9136 | 7883 | 10860 | 905 | 781 | 1076 | 3350 | 2509 | 3821 | 332 | 249 | 379 | 191 | 118 | 256 | 19 | 12 | 25 | 216 | 134 | 290 | 21 | 13 | 29 | 575 | 356 | 772 | 57 | 35 | 76 |
| 2017 | Jilin | 8762 | 7560 | 10416 | 918 | 792 | 1091 | 3213 | 2407 | 3664 | 336 | 252 | 384 | 166 | 103 | 223 | 17 | 11 | 23 | 189 | 117 | 254 | 20 | 12 | 27 | 503 | 311 | 674 | 53 | 33 | 71 |
| 2010 | Liaoning | 11670 | 10069 | 13872 | 551 | 475 | 655 | 4278 | 3205 | 4880 | 202 | 151 | 230 | 108 | 67 | 145 | 5 | 3 | 7 | 123 | 76 | 165 | 6 | 4 | 8 | 327 | 202 | 438 | 15 | 10 | 21 |
| 2011 | Liaoning | 11534 | 9952 | 13711 | 636 | 549 | 756 | 4229 | 3168 | 4823 | 233 | 175 | 266 | 102 | 63 | 137 | 6 | 3 | 8 | 116 | 72 | 156 | 6 | 4 | 9 | 309 | 191 | 414 | 17 | 11 | 23 |
| 2012 | Liaoning | 11409 | 9844 | 13562 | 741 | 639 | 880 | 4183 | 3134 | 4771 | 272 | 203 | 310 | 95 | 59 | 127 | 6 | 4 | 8 | 107 | 66 | 144 | 7 | 4 | 9 | 286 | 177 | 383 | 19 | 11 | 25 |
| 2013 | Liaoning | 11519 | 9939 | 13693 | 754 | 651 | 897 | 4223 | 3164 | 4817 | 277 | 207 | 315 | 89 | 55 | 119 | 6 | 4 | 8 | 101 | 63 | 136 | 7 | 4 | 9 | 269 | 166 | 361 | 18 | 11 | 24 |
| 2014 | Liaoning | 11680 | 10077 | 13884 | 752 | 649 | 894 | 4282 | 3208 | 4884 | 276 | 206 | 314 | 84 | 52 | 113 | 5 | 3 | 7 | 95 | 59 | 128 | 6 | 4 | 8 | 254 | 157 | 340 | 16 | 10 | 22 |
| 2015 | Liaoning | 11713 | 10107 | 13924 | 778 | 671 | 924 | 4295 | 3217 | 4898 | 285 | 214 | 325 | 77 | 48 | 104 | 5 | 3 | 7 | 88 | 54 | 118 | 6 | 4 | 8 | 233 | 144 | 313 | 15 | 10 | 21 |
| 2016 | Liaoning | 11978 | 10335 | 14239 | 793 | 684 | 943 | 4392 | 3290 | 5009 | 291 | 218 | 332 | 69 | 42 | 92 | 5 | 3 | 6 | 78 | 48 | 105 | 5 | 3 | 7 | 207 | 128 | 278 | 14 | 8 | 18 |
| 2017 | Liaoning | 12264 | 10582 | 14579 | 872 | 753 | 1037 | 4497 | 3369 | 5129 | 320 | 240 | 365 | 60 | 37 | 81 | 4 | 3 | 6 | 69 | 42 | 92 | 5 | 3 | 7 | 182 | 113 | 245 | 13 | 8 | 17 |
| 2010 | Ningxia | 4226 | 3646 | 5023 | 955 | 824 | 1135 | 1549 | 1161 | 1767 | 350 | 262 | 399 | 123 | 76 | 164 | 28 | 17 | 37 | 139 | 86 | 187 | 31 | 19 | 42 | 370 | 229 | 496 | 84 | 52 | 112 |
| 2011 | Ningxia | 4081 | 3522 | 4852 | 932 | 804 | 1108 | 1496 | 1121 | 1707 | 342 | 256 | 390 | 109 | 68 | 146 | 25 | 15 | 33 | 124 | 77 | 166 | 28 | 18 | 38 | 329 | 204 | 442 | 75 | 47 | 101 |
| 2012 | Ningxia | 3904 | 3369 | 4641 | 898 | 775 | 1068 | 1431 | 1072 | 1633 | 329 | 247 | 376 | 103 | 64 | 138 | 24 | 15 | 32 | 117 | 72 | 157 | 27 | 17 | 36 | 311 | 193 | 418 | 72 | 44 | 96 |
| 2013 | Ningxia | 3744 | 3231 | 4451 | 857 | 740 | 1019 | 1373 | 1028 | 1566 | 314 | 236 | 359 | 95 | 59 | 128 | 22 | 14 | 29 | 108 | 67 | 146 | 25 | 15 | 33 | 288 | 178 | 387 | 66 | 41 | 89 |
| 2014 | Ningxia | 3583 | 3091 | 4259 | 767 | 662 | 912 | 1313 | 984 | 1498 | 281 | 211 | 321 | 90 | 56 | 121 | 19 | 12 | 26 | 102 | 63 | 137 | 22 | 14 | 29 | 271 | 168 | 364 | 58 | 36 | 78 |
| 2015 | Ningxia | 3344 | 2886 | 3976 | 741 | 640 | 881 | 1226 | 919 | 1399 | 272 | 204 | 310 | 83 | 51 | 111 | 18 | 11 | 25 | 94 | 58 | 126 | 21 | 13 | 28 | 250 | 155 | 336 | 56 | 34 | 74 |
| 2016 | Ningxia | 3202 | 2763 | 3807 | 709 | 612 | 843 | 1174 | 880 | 1339 | 260 | 195 | 297 | 75 | 46 | 101 | 17 | 10 | 22 | 85 | 53 | 114 | 19 | 12 | 25 | 227 | 140 | 304 | 50 | 31 | 67 |
| 2017 | Ningxia | 3103 | 2678 | 3689 | 688 | 594 | 818 | 1138 | 852 | 1298 | 252 | 189 | 288 | 66 | 41 | 89 | 15 | 9 | 20 | 75 | 47 | 101 | 17 | 10 | 22 | 200 | 124 | 268 | 44 | 27 | 59 |
| 2010 | Qinghai | 4527 | 3906 | 5381 | 1241 | 1071 | 1475 | 1660 | 1243 | 1893 | 455 | 341 | 519 | 120 | 58 | 178 | 33 | 16 | 49 | 136 | 66 | 202 | 37 | 18 | 55 | 362 | 174 | 537 | 99 | 48 | 147 |
| 2011 | Qinghai | 4346 | 3750 | 5166 | 1194 | 1030 | 1420 | 1593 | 1194 | 1817 | 438 | 328 | 499 | 140 | 101 | 218 | 39 | 28 | 60 | 159 | 114 | 248 | 44 | 31 | 68 | 424 | 304 | 659 | 117 | 83 | 181 |
| 2012 | Qinghai | 4121 | 3556 | 4899 | 1146 | 988 | 1362 | 1511 | 1132 | 1723 | 420 | 315 | 479 | 152 | 104 | 225 | 42 | 29 | 62 | 173 | 118 | 255 | 48 | 33 | 71 | 459 | 313 | 678 | 128 | 87 | 189 |
| 2013 | Qinghai | 3905 | 3370 | 4642 | 1114 | 961 | 1324 | 1432 | 1073 | 1633 | 408 | 306 | 466 | 162 | 105 | 228 | 46 | 30 | 65 | 184 | 120 | 259 | 52 | 34 | 74 | 489 | 318 | 690 | 139 | 91 | 197 |
| 2014 | Qinghai | 3679 | 3174 | 4373 | 989 | 854 | 1176 | 1349 | 1010 | 1538 | 363 | 272 | 414 | 173 | 107 | 232 | 46 | 29 | 62 | 196 | 121 | 263 | 53 | 33 | 71 | 521 | 323 | 699 | 140 | 87 | 188 |
| 2015 | Qinghai | 3361 | 2900 | 3995 | 906 | 782 | 1077 | 1232 | 923 | 1405 | 332 | 249 | 379 | 152 | 94 | 204 | 41 | 25 | 55 | 172 | 107 | 231 | 46 | 29 | 62 | 458 | 284 | 615 | 124 | 76 | 166 |
| 2016 | Qinghai | 3144 | 2713 | 3737 | 838 | 723 | 997 | 1153 | 864 | 1315 | 307 | 230 | 351 | 136 | 84 | 183 | 36 | 23 | 49 | 155 | 96 | 208 | 41 | 26 | 55 | 412 | 255 | 553 | 110 | 68 | 147 |
| 2017 | Qinghai | 2968 | 2561 | 3528 | 788 | 680 | 937 | 1088 | 815 | 1241 | 289 | 216 | 330 | 118 | 73 | 159 | 31 | 19 | 42 | 134 | 83 | 180 | 36 | 22 | 48 | 357 | 221 | 479 | 95 | 59 | 127 |
| 2010 | Shaanxi | 17487 | 15088 | 20787 | 767 | 662 | 912 | 6411 | 4803 | 7313 | 281 | 211 | 321 | 583 | 361 | 782 | 26 | 16 | 34 | 662 | 410 | 888 | 29 | 18 | 39 | 1759 | 1089 | 2360 | 77 | 48 | 104 |
| 2011 | Shaanxi | 17284 | 14913 | 20545 | 842 | 726 | 1001 | 6337 | 4747 | 7228 | 309 | 231 | 352 | 551 | 341 | 739 | 27 | 17 | 36 | 626 | 387 | 840 | 30 | 19 | 41 | 1664 | 1030 | 2232 | 81 | 50 | 109 |
| 2012 | Shaanxi | 16857 | 14545 | 20039 | 868 | 749 | 1031 | 6181 | 4630 | 7049 | 318 | 238 | 363 | 519 | 321 | 696 | 27 | 17 | 36 | 589 | 365 | 791 | 30 | 19 | 41 | 1567 | 970 | 2102 | 81 | 50 | 108 |
| 2013 | Shaanxi | 16398 | 14148 | 19492 | 828 | 714 | 984 | 6012 | 4504 | 6857 | 303 | 227 | 346 | 490 | 303 | 658 | 25 | 15 | 33 | 557 | 345 | 747 | 28 | 17 | 38 | 1480 | 916 | 1986 | 75 | 46 | 100 |
| 2014 | Shaanxi | 15803 | 13636 | 18786 | 764 | 660 | 909 | 5794 | 4341 | 6609 | 280 | 210 | 320 | 472 | 292 | 633 | 23 | 14 | 31 | 536 | 332 | 719 | 26 | 16 | 35 | 1426 | 882 | 1913 | 69 | 43 | 93 |
| 2015 | Shaanxi | 14707 | 12689 | 17482 | 719 | 620 | 854 | 5392 | 4040 | 6150 | 263 | 197 | 301 | 443 | 274 | 594 | 22 | 13 | 29 | 503 | 311 | 675 | 25 | 15 | 33 | 1338 | 828 | 1795 | 65 | 40 | 88 |
| 2016 | Shaanxi | 13912 | 12004 | 16538 | 685 | 591 | 815 | 5101 | 3821 | 5818 | 251 | 188 | 287 | 402 | 249 | 539 | 20 | 12 | 27 | 456 | 282 | 612 | 22 | 14 | 30 | 1213 | 751 | 1627 | 60 | 37 | 80 |
| 2017 | Shaanxi | 13118 | 11319 | 15594 | 656 | 566 | 780 | 4810 | 3603 | 5486 | 240 | 180 | 274 | 353 | 218 | 473 | 18 | 11 | 24 | 401 | 248 | 538 | 20 | 12 | 27 | 1066 | 659 | 1430 | 53 | 33 | 71 |
| 2010 | Shandong | 22725 | 19607 | 27013 | 378 | 326 | 449 | 8332 | 6242 | 9503 | 139 | 104 | 158 | 421 | 261 | 565 | 7 | 4 | 9 | 478 | 296 | 642 | 8 | 5 | 11 | 1272 | 787 | 1707 | 21 | 13 | 28 |
| 2011 | Shandong | 23153 | 19977 | 27522 | 410 | 353 | 487 | 8489 | 6360 | 9682 | 150 | 113 | 171 | 408 | 252 | 547 | 7 | 4 | 10 | 463 | 287 | 622 | 8 | 5 | 11 | 1232 | 762 | 1652 | 22 | 13 | 29 |
| 2012 | Shandong | 23819 | 20551 | 28314 | 439 | 379 | 522 | 8733 | 6543 | 9961 | 161 | 121 | 184 | 386 | 239 | 518 | 7 | 4 | 10 | 438 | 271 | 588 | 8 | 5 | 11 | 1166 | 721 | 1564 | 21 | 13 | 29 |
| 2013 | Shandong | 24994 | 21566 | 29711 | 461 | 397 | 547 | 9164 | 6865 | 10452 | 169 | 127 | 193 | 365 | 226 | 490 | 7 | 4 | 9 | 415 | 257 | 557 | 8 | 5 | 10 | 1103 | 682 | 1480 | 20 | 13 | 27 |
| 2014 | Shandong | 26463 | 22833 | 31457 | 465 | 401 | 552 | 9702 | 7269 | 11066 | 170 | 128 | 194 | 347 | 215 | 466 | 6 | 4 | 8 | 394 | 244 | 529 | 7 | 4 | 9 | 1048 | 648 | 1406 | 18 | 11 | 25 |
| 2015 | Shandong | 27627 | 23837 | 32840 | 485 | 419 | 577 | 10129 | 7589 | 11553 | 178 | 133 | 203 | 319 | 197 | 428 | 6 | 3 | 8 | 362 | 224 | 486 | 6 | 4 | 9 | 962 | 596 | 1291 | 17 | 10 | 23 |
| 2016 | Shandong | 29372 | 25343 | 34915 | 499 | 431 | 594 | 10769 | 8068 | 12283 | 183 | 137 | 209 | 279 | 173 | 374 | 5 | 3 | 6 | 317 | 196 | 425 | 5 | 3 | 7 | 843 | 521 | 1131 | 14 | 9 | 19 |
| 2017 | Shandong | 31314 | 27019 | 37224 | 515 | 445 | 613 | 11481 | 8601 | 13095 | 189 | 142 | 216 | 242 | 150 | 324 | 4 | 2 | 5 | 274 | 170 | 368 | 5 | 3 | 6 | 730 | 451 | 979 | 12 | 7 | 16 |
| 2010 | Shanghai | 7236 | 6243 | 8601 | 833 | 718 | 990 | 2653 | 1987 | 3026 | 305 | 229 | 348 | 43 | 26 | 57 | 5 | 3 | 7 | 48 | 30 | 65 | 6 | 3 | 7 | 128 | 79 | 172 | 15 | 9 | 20 |
| 2011 | Shanghai | 7391 | 6377 | 8786 | 864 | 746 | 1027 | 2710 | 2030 | 3091 | 317 | 237 | 361 | 45 | 28 | 60 | 5 | 3 | 7 | 51 | 31 | 68 | 6 | 4 | 8 | 135 | 84 | 181 | 16 | 10 | 21 |
| 2012 | Shanghai | 7370 | 6359 | 8761 | 807 | 697 | 960 | 2702 | 2024 | 3082 | 296 | 222 | 338 | 46 | 28 | 61 | 5 | 3 | 7 | 52 | 32 | 70 | 6 | 4 | 8 | 138 | 85 | 185 | 15 | 9 | 20 |
| 2013 | Shanghai | 7700 | 6644 | 9153 | 822 | 709 | 977 | 2823 | 2115 | 3220 | 301 | 226 | 344 | 49 | 30 | 65 | 5 | 3 | 7 | 55 | 34 | 74 | 6 | 4 | 8 | 147 | 91 | 197 | 16 | 10 | 21 |
| 2014 | Shanghai | 7861 | 6782 | 9344 | 823 | 710 | 978 | 2882 | 2159 | 3287 | 302 | 226 | 344 | 50 | 31 | 67 | 5 | 3 | 7 | 57 | 35 | 76 | 6 | 4 | 8 | 152 | 94 | 203 | 16 | 10 | 21 |
| 2015 | Shanghai | 7867 | 6787 | 9351 | 840 | 724 | 998 | 2884 | 2161 | 3290 | 308 | 231 | 351 | 50 | 31 | 67 | 5 | 3 | 7 | 57 | 35 | 76 | 6 | 4 | 8 | 152 | 94 | 203 | 16 | 10 | 22 |
| 2016 | Shanghai | 8026 | 6925 | 9541 | 847 | 731 | 1007 | 2943 | 2205 | 3357 | 311 | 233 | 354 | 50 | 31 | 67 | 5 | 3 | 7 | 56 | 35 | 76 | 6 | 4 | 8 | 150 | 93 | 201 | 16 | 10 | 21 |
| 2017 | Shanghai | 7853 | 6776 | 9335 | 822 | 710 | 978 | 2879 | 2157 | 3284 | 301 | 226 | 344 | 44 | 27 | 60 | 5 | 3 | 6 | 50 | 31 | 68 | 5 | 3 | 7 | 134 | 83 | 180 | 14 | 9 | 19 |
| 2010 | Shanxi | 13403 | 11564 | 15933 | 635 | 548 | 755 | 4914 | 3682 | 5605 | 233 | 174 | 265 | 459 | 284 | 615 | 22 | 13 | 29 | 521 | 322 | 699 | 25 | 15 | 33 | 1385 | 857 | 1858 | 66 | 41 | 88 |
| 2011 | Shanxi | 13179 | 11371 | 15666 | 662 | 571 | 787 | 4832 | 3620 | 5511 | 243 | 182 | 277 | 442 | 274 | 593 | 22 | 14 | 30 | 502 | 311 | 674 | 25 | 16 | 34 | 1335 | 826 | 1792 | 67 | 42 | 90 |
| 2012 | Shanxi | 12927 | 11154 | 15366 | 700 | 604 | 832 | 4739 | 3551 | 5406 | 257 | 192 | 293 | 420 | 260 | 564 | 23 | 14 | 31 | 478 | 296 | 641 | 26 | 16 | 35 | 1270 | 786 | 1703 | 69 | 43 | 92 |
| 2013 | Shanxi | 12799 | 11043 | 15214 | 685 | 591 | 814 | 4692 | 3516 | 5352 | 251 | 188 | 286 | 400 | 248 | 537 | 21 | 13 | 29 | 455 | 281 | 610 | 24 | 15 | 33 | 1209 | 748 | 1622 | 65 | 40 | 87 |
| 2014 | Shanxi | 12724 | 10979 | 15125 | 654 | 564 | 777 | 4665 | 3495 | 5321 | 240 | 180 | 273 | 389 | 241 | 522 | 20 | 12 | 27 | 442 | 274 | 593 | 23 | 14 | 30 | 1175 | 727 | 1577 | 60 | 37 | 81 |
| 2015 | Shanxi | 12420 | 10716 | 14763 | 648 | 559 | 771 | 4553 | 3411 | 5194 | 238 | 178 | 271 | 370 | 229 | 496 | 19 | 12 | 26 | 420 | 260 | 564 | 22 | 14 | 29 | 1117 | 691 | 1498 | 58 | 36 | 78 |
| 2016 | Shanxi | 12521 | 10803 | 14884 | 658 | 568 | 783 | 4590 | 3439 | 5236 | 241 | 181 | 275 | 340 | 210 | 456 | 18 | 11 | 24 | 386 | 239 | 518 | 20 | 13 | 27 | 1025 | 635 | 1376 | 54 | 33 | 72 |
| 2017 | Shanxi | 12895 | 11126 | 15329 | 698 | 602 | 829 | 4728 | 3542 | 5392 | 256 | 192 | 292 | 304 | 188 | 408 | 16 | 10 | 22 | 345 | 214 | 463 | 19 | 12 | 25 | 918 | 568 | 1232 | 50 | 31 | 67 |
| 2010 | Sichuan | 51030 | 44030 | 60661 | 1111 | 958 | 1320 | 18709 | 14017 | 21340 | 407 | 305 | 464 | 934 | 578 | 1253 | 20 | 13 | 27 | 1061 | 656 | 1423 | 23 | 14 | 31 | 2820 | 1745 | 3784 | 61 | 38 | 82 |
| 2011 | Sichuan | 49483 | 42695 | 58821 | 1161 | 1002 | 1381 | 18142 | 13592 | 20693 | 426 | 319 | 486 | 851 | 527 | 1142 | 20 | 12 | 27 | 967 | 598 | 1297 | 23 | 14 | 30 | 2571 | 1591 | 3449 | 60 | 37 | 81 |
| 2012 | Sichuan | 47535 | 41014 | 56506 | 1124 | 970 | 1336 | 17428 | 13057 | 19878 | 412 | 309 | 470 | 764 | 473 | 1025 | 18 | 11 | 24 | 868 | 537 | 1164 | 21 | 13 | 28 | 2306 | 1427 | 3094 | 55 | 34 | 73 |
| 2013 | Sichuan | 45945 | 39642 | 54615 | 1113 | 960 | 1323 | 16845 | 12620 | 19213 | 408 | 306 | 465 | 689 | 427 | 925 | 17 | 10 | 22 | 783 | 484 | 1050 | 19 | 12 | 25 | 2081 | 1288 | 2792 | 50 | 31 | 68 |
| 2014 | Sichuan | 44058 | 38014 | 52373 | 1067 | 920 | 1268 | 16153 | 12102 | 18424 | 391 | 293 | 446 | 629 | 389 | 844 | 15 | 9 | 20 | 714 | 442 | 959 | 17 | 11 | 23 | 1899 | 1175 | 2549 | 46 | 28 | 62 |
| 2015 | Sichuan | 40974 | 35354 | 48707 | 998 | 861 | 1186 | 15023 | 11255 | 17135 | 366 | 274 | 417 | 559 | 346 | 750 | 14 | 8 | 18 | 635 | 393 | 851 | 15 | 10 | 21 | 1687 | 1044 | 2264 | 41 | 25 | 55 |
| 2016 | Sichuan | 38582 | 33290 | 45864 | 933 | 805 | 1110 | 14146 | 10598 | 16134 | 342 | 256 | 390 | 481 | 297 | 645 | 12 | 7 | 16 | 546 | 338 | 733 | 13 | 8 | 18 | 1451 | 898 | 1947 | 35 | 22 | 47 |
| 2017 | Sichuan | 35875 | 30954 | 42645 | 863 | 745 | 1026 | 13153 | 9854 | 15002 | 316 | 237 | 361 | 403 | 250 | 541 | 10 | 6 | 13 | 458 | 284 | 615 | 11 | 7 | 15 | 1218 | 754 | 1634 | 29 | 18 | 39 |
| 2010 | Tianjin | 4473 | 3860 | 5318 | 707 | 610 | 840 | 1640 | 1229 | 1871 | 259 | 194 | 296 | 45 | 28 | 60 | 7 | 4 | 10 | 51 | 32 | 69 | 8 | 5 | 11 | 136 | 84 | 183 | 22 | 13 | 29 |
| 2011 | Tianjin | 4537 | 3914 | 5393 | 899 | 776 | 1069 | 1663 | 1246 | 1897 | 330 | 247 | 376 | 46 | 28 | 61 | 9 | 6 | 12 | 52 | 32 | 70 | 10 | 6 | 14 | 138 | 85 | 185 | 27 | 17 | 37 |
| 2012 | Tianjin | 4558 | 3932 | 5418 | 880 | 760 | 1047 | 1671 | 1252 | 1906 | 323 | 242 | 368 | 46 | 28 | 61 | 9 | 5 | 12 | 52 | 32 | 70 | 10 | 6 | 13 | 138 | 85 | 185 | 27 | 17 | 36 |
| 2013 | Tianjin | 4671 | 4031 | 5553 | 866 | 747 | 1030 | 1713 | 1283 | 1954 | 318 | 238 | 362 | 47 | 29 | 63 | 9 | 5 | 12 | 53 | 33 | 71 | 10 | 6 | 13 | 141 | 87 | 189 | 26 | 16 | 35 |
| 2014 | Tianjin | 4729 | 4081 | 5622 | 830 | 716 | 987 | 1734 | 1299 | 1978 | 304 | 228 | 347 | 48 | 30 | 65 | 8 | 5 | 11 | 55 | 34 | 73 | 10 | 6 | 13 | 145 | 90 | 195 | 26 | 16 | 34 |
| 2015 | Tianjin | 4680 | 4038 | 5564 | 848 | 731 | 1008 | 1716 | 1286 | 1957 | 311 | 233 | 354 | 48 | 30 | 65 | 9 | 5 | 12 | 55 | 34 | 73 | 10 | 6 | 13 | 145 | 90 | 195 | 26 | 16 | 35 |
| 2016 | Tianjin | 4715 | 4069 | 5605 | 814 | 702 | 968 | 1729 | 1295 | 1972 | 299 | 224 | 340 | 46 | 29 | 62 | 8 | 5 | 11 | 53 | 33 | 71 | 9 | 6 | 12 | 140 | 87 | 188 | 24 | 15 | 33 |
| 2017 | Tianjin | 4743 | 4092 | 5638 | 823 | 710 | 978 | 1739 | 1303 | 1983 | 302 | 226 | 344 | 43 | 27 | 58 | 7 | 5 | 10 | 49 | 30 | 65 | 8 | 5 | 11 | 130 | 80 | 174 | 23 | 14 | 30 |
| 2010 | Tibet | 3898 | 3363 | 4633 | 2015 | 1739 | 2396 | 1429 | 1071 | 1630 | 739 | 554 | 843 | 47 | 25 | 77 | 24 | 13 | 40 | 54 | 29 | 88 | 28 | 15 | 45 | 142 | 76 | 233 | 74 | 39 | 121 |
| 2011 | Tibet | 3818 | 3294 | 4538 | 1988 | 1715 | 2363 | 1400 | 1049 | 1596 | 729 | 546 | 831 | 49 | 27 | 78 | 25 | 14 | 41 | 55 | 31 | 89 | 29 | 16 | 46 | 147 | 82 | 237 | 76 | 42 | 123 |
| 2012 | Tibet | 3669 | 3165 | 4361 | 1567 | 1352 | 1862 | 1345 | 1008 | 1534 | 574 | 430 | 655 | 49 | 27 | 78 | 21 | 11 | 33 | 55 | 30 | 88 | 24 | 13 | 38 | 148 | 81 | 235 | 63 | 34 | 100 |
| 2013 | Tibet | 3503 | 3022 | 4164 | 1720 | 1484 | 2044 | 1284 | 962 | 1465 | 631 | 472 | 719 | 48 | 26 | 76 | 24 | 13 | 37 | 55 | 30 | 87 | 27 | 15 | 43 | 146 | 79 | 231 | 72 | 39 | 113 |
| 2014 | Tibet | 3320 | 2864 | 3946 | 1348 | 1163 | 1603 | 1217 | 912 | 1388 | 494 | 370 | 564 | 47 | 25 | 74 | 19 | 10 | 30 | 54 | 29 | 84 | 22 | 12 | 34 | 142 | 76 | 223 | 58 | 31 | 91 |
| 2015 | Tibet | 3064 | 2644 | 3642 | 1238 | 1068 | 1472 | 1123 | 842 | 1281 | 454 | 340 | 518 | 43 | 24 | 69 | 17 | 10 | 28 | 49 | 27 | 78 | 20 | 11 | 32 | 130 | 71 | 207 | 53 | 29 | 84 |
| 2016 | Tibet | 2880 | 2485 | 3424 | 1110 | 958 | 1319 | 1056 | 791 | 1205 | 407 | 305 | 464 | 38 | 20 | 61 | 14 | 8 | 24 | 43 | 23 | 69 | 16 | 9 | 27 | 113 | 62 | 184 | 44 | 24 | 71 |
| 2017 | Tibet | 2738 | 2362 | 3255 | 1003 | 865 | 1192 | 1004 | 752 | 1145 | 368 | 276 | 419 | 33 | 18 | 53 | 12 | 7 | 19 | 37 | 20 | 60 | 14 | 7 | 22 | 98 | 54 | 160 | 36 | 20 | 59 |
| 2010 | Xinjiang | 18063 | 15585 | 21472 | 1059 | 913 | 1258 | 6622 | 4962 | 7554 | 388 | 291 | 443 | 296 | 158 | 486 | 17 | 9 | 28 | 336 | 179 | 552 | 20 | 11 | 32 | 894 | 476 | 1466 | 52 | 28 | 86 |
| 2011 | Xinjiang | 17777 | 15339 | 21132 | 1050 | 906 | 1249 | 6518 | 4883 | 7434 | 385 | 289 | 439 | 293 | 163 | 474 | 17 | 10 | 28 | 333 | 185 | 538 | 20 | 11 | 32 | 885 | 492 | 1430 | 52 | 29 | 85 |
| 2012 | Xinjiang | 17319 | 14943 | 20587 | 964 | 832 | 1146 | 6350 | 4757 | 7243 | 353 | 265 | 403 | 284 | 155 | 451 | 16 | 9 | 25 | 322 | 176 | 512 | 18 | 10 | 29 | 856 | 468 | 1362 | 48 | 26 | 76 |
| 2013 | Xinjiang | 16902 | 14583 | 20091 | 925 | 798 | 1099 | 6197 | 4643 | 7068 | 339 | 254 | 387 | 271 | 146 | 428 | 15 | 8 | 23 | 308 | 166 | 486 | 17 | 9 | 27 | 819 | 441 | 1291 | 45 | 24 | 71 |
| 2014 | Xinjiang | 16384 | 14137 | 19476 | 876 | 756 | 1041 | 6007 | 4500 | 6852 | 321 | 241 | 366 | 272 | 144 | 422 | 15 | 8 | 23 | 309 | 163 | 480 | 17 | 9 | 26 | 821 | 433 | 1275 | 44 | 23 | 68 |
| 2015 | Xinjiang | 15384 | 13273 | 18287 | 816 | 704 | 970 | 5640 | 4226 | 6433 | 299 | 224 | 341 | 273 | 145 | 423 | 14 | 8 | 22 | 310 | 165 | 480 | 16 | 9 | 25 | 824 | 439 | 1277 | 44 | 23 | 68 |
| 2016 | Xinjiang | 14771 | 12744 | 17558 | 762 | 657 | 905 | 5415 | 4057 | 6177 | 279 | 209 | 319 | 273 | 140 | 419 | 14 | 7 | 22 | 310 | 159 | 476 | 16 | 8 | 25 | 823 | 424 | 1265 | 42 | 22 | 65 |
| 2017 | Xinjiang | 14296 | 12335 | 16994 | 703 | 607 | 836 | 5241 | 3927 | 5978 | 258 | 193 | 294 | 291 | 222 | 481 | 14 | 11 | 24 | 330 | 252 | 546 | 16 | 12 | 27 | 879 | 669 | 1451 | 43 | 33 | 71 |
| 2010 | Yunnan | 42639 | 36790 | 50685 | 1414 | 1220 | 1680 | 15633 | 11712 | 17831 | 518 | 388 | 591 | 900 | 557 | 1207 | 30 | 18 | 40 | 1022 | 632 | 1371 | 34 | 21 | 45 | 2716 | 1681 | 3644 | 90 | 56 | 121 |
| 2011 | Yunnan | 41007 | 35382 | 48746 | 1408 | 1215 | 1674 | 15035 | 11264 | 17148 | 516 | 387 | 589 | 862 | 534 | 1157 | 30 | 18 | 40 | 979 | 606 | 1314 | 34 | 21 | 45 | 2604 | 1611 | 3493 | 89 | 55 | 120 |
| 2012 | Yunnan | 38930 | 33590 | 46277 | 1347 | 1162 | 1602 | 14273 | 10693 | 16280 | 494 | 370 | 563 | 805 | 498 | 1080 | 28 | 17 | 37 | 914 | 566 | 1227 | 32 | 20 | 42 | 2431 | 1505 | 3262 | 84 | 52 | 113 |
| 2013 | Yunnan | 36920 | 31855 | 43887 | 1283 | 1107 | 1525 | 13536 | 10141 | 15439 | 470 | 352 | 536 | 753 | 466 | 1010 | 26 | 16 | 35 | 855 | 529 | 1147 | 30 | 18 | 40 | 2273 | 1407 | 3050 | 79 | 49 | 106 |
| 2014 | Yunnan | 34802 | 30028 | 41370 | 1210 | 1044 | 1439 | 12760 | 9559 | 14554 | 444 | 332 | 506 | 712 | 440 | 955 | 25 | 15 | 33 | 808 | 500 | 1085 | 28 | 17 | 38 | 2149 | 1330 | 2884 | 75 | 46 | 100 |
| 2015 | Yunnan | 31934 | 27553 | 37960 | 1125 | 971 | 1337 | 11708 | 8772 | 13354 | 412 | 309 | 470 | 647 | 401 | 869 | 23 | 14 | 31 | 735 | 455 | 987 | 26 | 16 | 35 | 1955 | 1210 | 2623 | 69 | 43 | 92 |
| 2016 | Yunnan | 29739 | 25659 | 35351 | 1041 | 898 | 1238 | 10903 | 8169 | 12436 | 382 | 286 | 435 | 572 | 354 | 768 | 20 | 12 | 27 | 650 | 402 | 872 | 23 | 14 | 31 | 1728 | 1069 | 2318 | 60 | 37 | 81 |
| 2017 | Yunnan | 27765 | 23956 | 33005 | 976 | 842 | 1160 | 10180 | 7626 | 11611 | 358 | 268 | 408 | 491 | 304 | 659 | 17 | 11 | 23 | 558 | 345 | 749 | 20 | 12 | 26 | 1483 | 918 | 1990 | 52 | 32 | 70 |
| 2010 | Zhejiang | 23865 | 20591 | 28369 | 768 | 663 | 913 | 8750 | 6555 | 9980 | 282 | 211 | 321 | 256 | 158 | 343 | 8 | 5 | 11 | 290 | 180 | 390 | 9 | 6 | 13 | 772 | 478 | 1036 | 25 | 15 | 33 |
| 2011 | Zhejiang | 23549 | 20319 | 27994 | 919 | 793 | 1092 | 8634 | 6469 | 9848 | 337 | 252 | 384 | 243 | 151 | 326 | 9 | 6 | 13 | 276 | 171 | 371 | 11 | 7 | 14 | 734 | 455 | 985 | 29 | 18 | 38 |
| 2012 | Zhejiang | 22990 | 19836 | 27328 | 864 | 746 | 1028 | 8429 | 6315 | 9614 | 317 | 237 | 362 | 229 | 141 | 307 | 9 | 5 | 12 | 260 | 161 | 348 | 10 | 6 | 13 | 690 | 427 | 926 | 26 | 16 | 35 |
| 2013 | Zhejiang | 22892 | 19752 | 27213 | 870 | 751 | 1035 | 8393 | 6288 | 9573 | 319 | 239 | 364 | 218 | 135 | 293 | 8 | 5 | 11 | 248 | 154 | 333 | 9 | 6 | 13 | 660 | 408 | 885 | 25 | 16 | 34 |
| 2014 | Zhejiang | 22730 | 19612 | 27020 | 852 | 735 | 1013 | 8334 | 6244 | 9505 | 312 | 234 | 356 | 208 | 129 | 279 | 8 | 5 | 10 | 236 | 146 | 316 | 9 | 5 | 12 | 627 | 388 | 841 | 24 | 15 | 32 |
| 2015 | Zhejiang | 22271 | 19216 | 26474 | 848 | 732 | 1008 | 8165 | 6118 | 9313 | 311 | 233 | 355 | 191 | 118 | 256 | 7 | 4 | 10 | 217 | 134 | 291 | 8 | 5 | 11 | 577 | 357 | 774 | 22 | 14 | 29 |
| 2016 | Zhejiang | 22315 | 19254 | 26527 | 835 | 721 | 993 | 8182 | 6130 | 9332 | 306 | 229 | 349 | 170 | 105 | 228 | 6 | 4 | 9 | 193 | 120 | 259 | 7 | 4 | 10 | 514 | 318 | 689 | 19 | 12 | 26 |
| 2017 | Zhejiang | 22278 | 19222 | 26482 | 829 | 715 | 986 | 8168 | 6119 | 9316 | 304 | 228 | 347 | 146 | 90 | 195 | 5 | 3 | 7 | 165 | 102 | 222 | 6 | 4 | 8 | 439 | 272 | 590 | 16 | 10 | 22 |
| 2010 | Central | 209982 | 181177 | 249609 | 651 | 562 | 774 | 76986 | 57678 | 87810 | 239 | 179 | 272 | 5537 | 3427 | 7430 | 17 | 11 | 23 | 6289 | 3892 | 8439 | 20 | 12 | 26 | 16721 | 10348 | 22434 | 52 | 32 | 70 |
| 2011 | Central | 207777 | 179275 | 246989 | 657 | 567 | 781 | 76178 | 57072 | 86889 | 241 | 180 | 275 | 5269 | 3261 | 7069 | 17 | 10 | 22 | 5984 | 3703 | 8029 | 19 | 12 | 25 | 15910 | 9846 | 21347 | 50 | 31 | 68 |
| 2012 | Central | 204819 | 176722 | 243472 | 649 | 560 | 772 | 75094 | 56260 | 85651 | 238 | 178 | 272 | 4913 | 3041 | 6592 | 16 | 10 | 21 | 5581 | 3454 | 7488 | 18 | 11 | 24 | 14837 | 9182 | 19906 | 47 | 29 | 63 |
| 2013 | Central | 203328 | 175436 | 241700 | 642 | 554 | 763 | 74547 | 55850 | 85028 | 235 | 176 | 269 | 4590 | 2841 | 6159 | 14 | 9 | 19 | 5214 | 3227 | 6995 | 16 | 10 | 22 | 13861 | 8578 | 18598 | 44 | 27 | 59 |
| 2014 | Central | 201593 | 173939 | 239638 | 631 | 545 | 750 | 73911 | 55374 | 84303 | 231 | 173 | 264 | 4337 | 2684 | 5818 | 14 | 8 | 18 | 4925 | 3048 | 6609 | 15 | 10 | 21 | 13095 | 8104 | 17570 | 41 | 25 | 55 |
| 2015 | Central | 195169 | 168396 | 232000 | 618 | 533 | 735 | 71555 | 53609 | 81616 | 227 | 170 | 258 | 3992 | 2470 | 5356 | 13 | 8 | 17 | 4534 | 2806 | 6083 | 14 | 9 | 19 | 12053 | 7459 | 16172 | 38 | 24 | 51 |
| 2016 | Central | 192411 | 166016 | 228722 | 609 | 525 | 723 | 70544 | 52851 | 80463 | 223 | 167 | 254 | 3568 | 2208 | 4787 | 11 | 7 | 15 | 4053 | 2508 | 5437 | 13 | 8 | 17 | 10774 | 6668 | 14456 | 34 | 21 | 46 |
| 2017 | Central | 190550 | 164411 | 226510 | 599 | 517 | 712 | 69862 | 52340 | 79684 | 220 | 165 | 251 | 3129 | 1936 | 4198 | 10 | 6 | 13 | 3554 | 2199 | 4768 | 11 | 7 | 15 | 9448 | 5847 | 12677 | 30 | 18 | 40 |
| 2010 | East | 196748 | 169758 | 233878 | 751 | 648 | 892 | 72134 | 54043 | 82276 | 275 | 206 | 314 | 2176 | 1347 | 2920 | 8 | 5 | 11 | 2472 | 1530 | 3316 | 9 | 6 | 13 | 6571 | 4067 | 8817 | 25 | 16 | 34 |
| 2011 | East | 195495 | 168677 | 232388 | 839 | 724 | 997 | 71675 | 53699 | 81752 | 307 | 230 | 351 | 2095 | 1297 | 2811 | 9 | 6 | 12 | 2380 | 1473 | 3193 | 10 | 6 | 14 | 6327 | 3915 | 8489 | 27 | 17 | 36 |
| 2012 | East | 192575 | 166158 | 228917 | 782 | 675 | 929 | 70604 | 52896 | 80531 | 287 | 215 | 327 | 1987 | 1229 | 2665 | 8 | 5 | 11 | 2256 | 1396 | 3027 | 9 | 6 | 12 | 5999 | 3712 | 8049 | 24 | 15 | 33 |
| 2013 | East | 193390 | 166861 | 229886 | 784 | 676 | 932 | 70903 | 53120 | 80872 | 287 | 215 | 328 | 1908 | 1181 | 2561 | 8 | 5 | 10 | 2168 | 1341 | 2908 | 9 | 5 | 12 | 5763 | 3566 | 7732 | 23 | 14 | 31 |
| 2014 | East | 193478 | 166937 | 229991 | 759 | 655 | 902 | 70936 | 53145 | 80909 | 278 | 208 | 317 | 1839 | 1138 | 2468 | 7 | 4 | 10 | 2089 | 1293 | 2803 | 8 | 5 | 11 | 5554 | 3437 | 7452 | 22 | 13 | 29 |
| 2015 | East | 190263 | 164163 | 226170 | 753 | 650 | 895 | 69757 | 52262 | 79565 | 276 | 207 | 315 | 1722 | 1066 | 2311 | 7 | 4 | 9 | 1956 | 1211 | 2625 | 8 | 5 | 10 | 5201 | 3218 | 6978 | 21 | 13 | 28 |
| 2016 | East | 190737 | 164572 | 226732 | 743 | 641 | 884 | 69931 | 52392 | 79763 | 273 | 204 | 311 | 1554 | 961 | 2084 | 6 | 4 | 8 | 1765 | 1092 | 2367 | 7 | 4 | 9 | 4691 | 2903 | 6294 | 18 | 11 | 25 |
| 2017 | East | 191099 | 164884 | 227163 | 722 | 623 | 858 | 70063 | 52491 | 79914 | 265 | 198 | 302 | 1364 | 844 | 1831 | 5 | 3 | 7 | 1550 | 959 | 2079 | 6 | 4 | 8 | 4120 | 2550 | 5528 | 16 | 10 | 21 |
| 2010 | West | 226694 | 195596 | 269475 | 983 | 848 | 1168 | 83114 | 62268 | 94799 | 360 | 270 | 411 | 4702 | 2788 | 6432 | 20 | 12 | 28 | 5340 | 3167 | 7306 | 23 | 14 | 32 | 14197 | 8419 | 19423 | 62 | 36 | 84 |
| 2011 | West | 220852 | 190556 | 262531 | 991 | 855 | 1178 | 80972 | 60664 | 92356 | 363 | 272 | 414 | 4525 | 2821 | 6256 | 20 | 13 | 28 | 5140 | 3204 | 7106 | 23 | 14 | 32 | 13664 | 8518 | 18891 | 61 | 38 | 85 |
| 2012 | West | 213290 | 184031 | 253542 | 962 | 830 | 1143 | 78199 | 58587 | 89194 | 353 | 264 | 402 | 4268 | 2644 | 5868 | 19 | 12 | 26 | 4847 | 3003 | 6665 | 22 | 14 | 30 | 12887 | 7985 | 17720 | 58 | 36 | 80 |
| 2013 | West | 206833 | 178460 | 245866 | 931 | 803 | 1107 | 75832 | 56813 | 86494 | 341 | 256 | 389 | 4022 | 2477 | 5501 | 18 | 11 | 25 | 4568 | 2814 | 6249 | 21 | 13 | 28 | 12144 | 7480 | 16612 | 55 | 34 | 75 |
| 2014 | West | 199763 | 172360 | 237462 | 880 | 759 | 1046 | 73240 | 54871 | 83537 | 323 | 242 | 368 | 3842 | 2349 | 5222 | 17 | 10 | 23 | 4363 | 2668 | 5932 | 19 | 12 | 26 | 11600 | 7092 | 15770 | 51 | 31 | 69 |
| 2015 | West | 187892 | 162117 | 223350 | 834 | 720 | 991 | 68887 | 51610 | 78573 | 306 | 229 | 349 | 3505 | 2142 | 4770 | 16 | 10 | 21 | 3981 | 2433 | 5417 | 18 | 11 | 24 | 10583 | 6468 | 14403 | 47 | 29 | 64 |
| 2016 | West | 179569 | 154936 | 213457 | 794 | 685 | 944 | 65836 | 49324 | 75093 | 291 | 218 | 332 | 3114 | 1896 | 4241 | 14 | 8 | 19 | 3536 | 2153 | 4817 | 16 | 10 | 21 | 9402 | 5724 | 12807 | 42 | 25 | 57 |
| 2017 | West | 171388 | 147877 | 203732 | 740 | 639 | 880 | 62837 | 47077 | 71671 | 271 | 203 | 310 | 2731 | 1729 | 3764 | 12 | 7 | 16 | 3102 | 1964 | 4275 | 13 | 8 | 18 | 8247 | 5222 | 11366 | 36 | 23 | 49 |
| 2010 | National | 633423 | 546531 | 752962 | 777 | 670 | 924 | 232235 | 173989 | 264886 | 285 | 213 | 325 | 12415 | 7562 | 16782 | 15 | 9 | 21 | 14101 | 8588 | 19061 | 17 | 11 | 23 | 37489 | 22833 | 50674 | 46 | 28 | 62 |
| 2011 | National | 624125 | 538508 | 741908 | 808 | 697 | 961 | 228825 | 171435 | 260997 | 296 | 222 | 338 | 11889 | 7378 | 16137 | 15 | 10 | 21 | 13504 | 8380 | 18328 | 17 | 11 | 24 | 35901 | 22279 | 48726 | 46 | 29 | 63 |
| 2012 | National | 610683 | 526911 | 725930 | 779 | 673 | 927 | 223897 | 167743 | 255377 | 286 | 214 | 326 | 11168 | 6914 | 15126 | 14 | 9 | 19 | 12684 | 7853 | 17180 | 16 | 10 | 22 | 33722 | 20879 | 45675 | 43 | 27 | 58 |
| 2013 | National | 603551 | 520757 | 717452 | 768 | 663 | 913 | 221282 | 165784 | 252394 | 282 | 211 | 321 | 10521 | 6499 | 14221 | 13 | 8 | 18 | 11949 | 7382 | 16152 | 15 | 9 | 21 | 31768 | 19625 | 42942 | 40 | 25 | 55 |
| 2014 | National | 594834 | 513236 | 707091 | 742 | 641 | 882 | 218087 | 163389 | 248749 | 272 | 204 | 310 | 10018 | 6171 | 13509 | 13 | 8 | 17 | 11378 | 7009 | 15343 | 14 | 9 | 19 | 30249 | 18633 | 40791 | 38 | 23 | 51 |
| 2015 | National | 573324 | 494676 | 681520 | 722 | 623 | 859 | 210200 | 157481 | 239753 | 265 | 198 | 302 | 9219 | 5678 | 12436 | 12 | 7 | 16 | 10470 | 6449 | 14125 | 13 | 8 | 18 | 27836 | 17146 | 37553 | 35 | 22 | 47 |
| 2016 | National | 562717 | 485524 | 668912 | 704 | 608 | 837 | 206311 | 154567 | 235318 | 258 | 194 | 295 | 8235 | 5065 | 11113 | 10 | 6 | 14 | 9353 | 5753 | 12622 | 12 | 7 | 16 | 24867 | 15295 | 33557 | 31 | 19 | 42 |
| 2017 | National | 553037 | 477172 | 657405 | 679 | 586 | 807 | 202762 | 151908 | 231270 | 249 | 187 | 284 | 7225 | 4510 | 9793 | 9 | 6 | 12 | 8206 | 5123 | 11123 | 10 | 6 | 14 | 21815 | 13619 | 29571 | 27 | 17 | 36 |
| 1. Clinical pneumonia cases include severe pneumonia cases. | | | | | | | | | | | | | | | | | | | | | | | | | | | | | | | |
| 2. All meningitis cases are severe. | | | | | | | | | | | | | | | | | | | | | | | | | | | | | | | |
| 3. Severe and non-severe NPNM cases are mutually exclusive. | | | | | | | | | | | | | | | | | | | | | | | | | | | | | | | |

**Webappendix 11: Hib cases in China by region, province, and year**

| **Year** | **Province** | **Hib clinical pneumonia cases^1^** | | | | | | **Hib severe pneumonia cases^1^** | | | | | | **Hib meningitis cases^2^** | | | | | | **Hib severe NPNM cases^3^** | | | | | |
| --- | --- | --- | --- | --- | --- | --- | --- | --- | --- | --- | --- | --- | --- | --- | --- | --- | --- | --- | --- | --- | --- | --- | --- | --- | --- |
|  |  | Number | | | Rate per 100 000 | | | Number | | | Rate per 100 000 | | | Number | | | Rate per 100 000 | | | Number | | | Rate per 100 000 | | |
|  |  | Mean | UR | | Mean | UR | | Mean | UR | | Mean | UR | | Mean | UR | | Mean | UR | | Mean | UR | | Mean | UR | |
| 2010 | Anhui | 13967 | 12752 | 22820 | 374 | 342 | 611 | 2453 | 1469 | 4952 | 66 | 39 | 133 | 549 | 259 | 992 | 15 | 7 | 27 | 185 | 87 | 334 | 5 | 2 | 9 |
| 2011 | Anhui | 12186 | 11126 | 19911 | 335 | 306 | 548 | 2141 | 1282 | 4321 | 59 | 35 | 119 | 445 | 210 | 805 | 12 | 6 | 22 | 150 | 71 | 271 | 4 | 2 | 7 |
| 2012 | Anhui | 12267 | 11200 | 20044 | 336 | 307 | 549 | 2155 | 1290 | 4349 | 59 | 35 | 119 | 410 | 193 | 740 | 11 | 5 | 20 | 138 | 65 | 249 | 4 | 2 | 7 |
| 2013 | Anhui | 10322 | 9424 | 16865 | 286 | 261 | 467 | 1813 | 1086 | 3660 | 50 | 30 | 101 | 310 | 146 | 561 | 9 | 4 | 16 | 105 | 49 | 189 | 3 | 1 | 5 |
| 2014 | Anhui | 10149 | 9266 | 16583 | 279 | 255 | 457 | 1783 | 1067 | 3598 | 49 | 29 | 99 | 273 | 129 | 494 | 8 | 4 | 14 | 92 | 43 | 167 | 3 | 1 | 5 |
| 2015 | Anhui | 9111 | 8318 | 14886 | 252 | 230 | 412 | 1600 | 958 | 3230 | 44 | 26 | 89 | 226 | 107 | 409 | 6 | 3 | 11 | 76 | 36 | 138 | 2 | 1 | 4 |
| 2016 | Anhui | 11102 | 10137 | 18140 | 297 | 271 | 486 | 1950 | 1168 | 3936 | 52 | 31 | 105 | 243 | 114 | 438 | 6 | 3 | 12 | 82 | 39 | 148 | 2 | 1 | 4 |
| 2017 | Anhui | 9966 | 9099 | 16283 | 258 | 236 | 421 | 1751 | 1048 | 3533 | 45 | 27 | 91 | 189 | 89 | 342 | 5 | 2 | 9 | 64 | 30 | 115 | 2 | 1 | 3 |
| 2010 | Beijing | 2921 | 2666 | 4772 | 349 | 319 | 571 | 513 | 307 | 1035 | 61 | 37 | 124 | 26 | 12 | 48 | 3 | 1 | 6 | 9 | 4 | 16 | 1 | 1 | 2 |
| 2011 | Beijing | 2577 | 2353 | 4211 | 432 | 395 | 707 | 453 | 271 | 914 | 76 | 45 | 153 | 23 | 11 | 42 | 4 | 2 | 7 | 8 | 4 | 14 | 1 | 1 | 2 |
| 2012 | Beijing | 2730 | 2492 | 4460 | 343 | 313 | 561 | 479 | 287 | 968 | 60 | 36 | 122 | 24 | 12 | 44 | 3 | 1 | 6 | 8 | 4 | 15 | 1 | 0 | 2 |
| 2013 | Beijing | 3070 | 2803 | 5016 | 371 | 339 | 607 | 539 | 323 | 1088 | 65 | 39 | 132 | 27 | 13 | 49 | 3 | 2 | 6 | 9 | 4 | 16 | 1 | 1 | 2 |
| 2014 | Beijing | 3530 | 3223 | 5768 | 410 | 374 | 670 | 620 | 371 | 1252 | 72 | 43 | 145 | 32 | 15 | 57 | 4 | 2 | 7 | 11 | 5 | 19 | 1 | 1 | 2 |
| 2015 | Beijing | 2531 | 2311 | 4136 | 300 | 274 | 490 | 445 | 266 | 897 | 53 | 32 | 106 | 23 | 11 | 41 | 3 | 1 | 5 | 8 | 4 | 14 | 1 | 0 | 2 |
| 2016 | Beijing | 3194 | 2917 | 5219 | 367 | 335 | 599 | 561 | 336 | 1133 | 64 | 39 | 130 | 28 | 13 | 50 | 3 | 1 | 6 | 9 | 4 | 17 | 1 | 1 | 2 |
| 2017 | Beijing | 3276 | 2991 | 5352 | 350 | 319 | 572 | 575 | 345 | 1161 | 61 | 37 | 124 | 26 | 12 | 47 | 3 | 1 | 5 | 9 | 4 | 16 | 1 | 0 | 2 |
| 2010 | Chongqing | 6948 | 6343 | 11352 | 474 | 433 | 775 | 1220 | 731 | 2463 | 83 | 50 | 168 | 147 | 69 | 266 | 10 | 5 | 18 | 50 | 23 | 90 | 3 | 2 | 6 |
| 2011 | Chongqing | 6175 | 5638 | 10090 | 411 | 376 | 672 | 1085 | 649 | 2189 | 72 | 43 | 146 | 128 | 60 | 231 | 9 | 4 | 15 | 43 | 20 | 78 | 3 | 1 | 5 |
| 2012 | Chongqing | 5182 | 4731 | 8466 | 329 | 300 | 537 | 910 | 545 | 1837 | 58 | 35 | 116 | 104 | 49 | 188 | 7 | 3 | 12 | 35 | 16 | 63 | 2 | 1 | 4 |
| 2013 | Chongqing | 4954 | 4523 | 8094 | 321 | 293 | 524 | 870 | 521 | 1756 | 56 | 34 | 114 | 96 | 45 | 174 | 6 | 3 | 11 | 32 | 15 | 59 | 2 | 1 | 4 |
| 2014 | Chongqing | 4575 | 4177 | 7475 | 289 | 263 | 471 | 804 | 481 | 1622 | 51 | 30 | 102 | 85 | 40 | 154 | 5 | 3 | 10 | 29 | 14 | 52 | 2 | 1 | 3 |
| 2015 | Chongqing | 3620 | 3305 | 5915 | 229 | 209 | 374 | 636 | 381 | 1284 | 40 | 24 | 81 | 64 | 30 | 115 | 4 | 2 | 7 | 22 | 10 | 39 | 1 | 1 | 2 |
| 2016 | Chongqing | 4595 | 4195 | 7508 | 296 | 270 | 483 | 807 | 483 | 1629 | 52 | 31 | 105 | 71 | 33 | 128 | 5 | 2 | 8 | 24 | 11 | 43 | 2 | 1 | 3 |
| 2017 | Chongqing | 4227 | 3859 | 6907 | 258 | 235 | 421 | 743 | 445 | 1499 | 45 | 27 | 91 | 55 | 26 | 99 | 3 | 2 | 6 | 19 | 9 | 34 | 1 | 1 | 2 |
| 2010 | Fujian | 9609 | 8773 | 15699 | 388 | 354 | 633 | 1688 | 1011 | 3407 | 68 | 41 | 137 | 179 | 84 | 324 | 7 | 3 | 13 | 60 | 28 | 109 | 2 | 1 | 4 |
| 2011 | Fujian | 8513 | 7773 | 13910 | 404 | 369 | 660 | 1495 | 895 | 3018 | 71 | 42 | 143 | 154 | 72 | 278 | 7 | 3 | 13 | 52 | 24 | 94 | 2 | 1 | 4 |
| 2012 | Fujian | 9544 | 8714 | 15594 | 414 | 378 | 677 | 1676 | 1004 | 3384 | 73 | 44 | 147 | 166 | 78 | 300 | 7 | 3 | 13 | 56 | 26 | 101 | 2 | 1 | 4 |
| 2013 | Fujian | 6627 | 6051 | 10828 | 284 | 259 | 464 | 1164 | 697 | 2350 | 50 | 30 | 101 | 112 | 53 | 202 | 5 | 2 | 9 | 38 | 18 | 68 | 2 | 1 | 3 |
| 2014 | Fujian | 7038 | 6425 | 11499 | 290 | 265 | 475 | 1236 | 740 | 2495 | 51 | 31 | 103 | 117 | 55 | 211 | 5 | 2 | 9 | 39 | 19 | 71 | 2 | 1 | 3 |
| 2015 | Fujian | 6041 | 5516 | 9871 | 252 | 230 | 411 | 1061 | 635 | 2142 | 44 | 26 | 89 | 98 | 46 | 177 | 4 | 2 | 7 | 33 | 16 | 60 | 1 | 1 | 2 |
| 2016 | Fujian | 7405 | 6761 | 12100 | 303 | 277 | 496 | 1301 | 779 | 2626 | 53 | 32 | 108 | 111 | 52 | 201 | 5 | 2 | 8 | 37 | 18 | 68 | 2 | 1 | 3 |
| 2017 | Fujian | 6952 | 6348 | 11359 | 280 | 256 | 458 | 1221 | 731 | 2465 | 49 | 29 | 99 | 95 | 45 | 171 | 4 | 2 | 7 | 32 | 15 | 58 | 1 | 1 | 2 |
| 2010 | Gansu | 8727 | 7968 | 14259 | 547 | 500 | 894 | 1533 | 918 | 3094 | 96 | 58 | 194 | 455 | 277 | 876 | 29 | 17 | 55 | 153 | 93 | 295 | 10 | 6 | 19 |
| 2011 | Gansu | 8467 | 7730 | 13834 | 565 | 515 | 922 | 1487 | 890 | 3002 | 99 | 59 | 200 | 494 | 190 | 730 | 33 | 13 | 49 | 167 | 64 | 246 | 11 | 4 | 16 |
| 2012 | Gansu | 8125 | 7419 | 13276 | 574 | 524 | 938 | 1427 | 855 | 2881 | 101 | 60 | 204 | 499 | 206 | 788 | 35 | 15 | 56 | 168 | 69 | 266 | 12 | 5 | 19 |
| 2013 | Gansu | 7841 | 7159 | 12811 | 556 | 508 | 909 | 1377 | 825 | 2780 | 98 | 59 | 197 | 492 | 216 | 829 | 35 | 15 | 59 | 166 | 73 | 279 | 12 | 5 | 20 |
| 2014 | Gansu | 7620 | 6957 | 12450 | 519 | 474 | 847 | 1339 | 801 | 2702 | 91 | 55 | 184 | 493 | 232 | 891 | 34 | 16 | 61 | 166 | 78 | 300 | 11 | 5 | 20 |
| 2015 | Gansu | 7119 | 6500 | 11632 | 488 | 445 | 797 | 1251 | 749 | 2524 | 86 | 51 | 173 | 434 | 204 | 783 | 30 | 14 | 54 | 146 | 69 | 264 | 10 | 5 | 18 |
| 2016 | Gansu | 6970 | 6363 | 11388 | 475 | 434 | 777 | 1224 | 733 | 2471 | 83 | 50 | 169 | 378 | 178 | 682 | 26 | 12 | 47 | 127 | 60 | 230 | 9 | 4 | 16 |
| 2017 | Gansu | 6685 | 6103 | 10922 | 454 | 415 | 742 | 1174 | 703 | 2370 | 80 | 48 | 161 | 314 | 148 | 567 | 21 | 10 | 39 | 106 | 50 | 191 | 7 | 3 | 13 |
| 2010 | Guangdong | 29559 | 26988 | 48297 | 519 | 473 | 847 | 5192 | 3109 | 10480 | 91 | 55 | 184 | 411 | 194 | 743 | 7 | 3 | 13 | 139 | 65 | 250 | 2 | 1 | 4 |
| 2011 | Guangdong | 23239 | 21218 | 37970 | 435 | 397 | 710 | 4082 | 2444 | 8239 | 76 | 46 | 154 | 316 | 149 | 571 | 6 | 3 | 11 | 106 | 50 | 192 | 2 | 1 | 4 |
| 2012 | Guangdong | 25234 | 23039 | 41229 | 387 | 353 | 632 | 4433 | 2654 | 8947 | 68 | 41 | 137 | 337 | 159 | 609 | 5 | 2 | 9 | 114 | 54 | 205 | 2 | 1 | 3 |
| 2013 | Guangdong | 33607 | 30684 | 54911 | 515 | 470 | 841 | 5903 | 3535 | 11915 | 90 | 54 | 182 | 444 | 209 | 803 | 7 | 3 | 12 | 150 | 71 | 271 | 2 | 1 | 4 |
| 2014 | Guangdong | 21962 | 20052 | 35884 | 322 | 294 | 527 | 3858 | 2310 | 7787 | 57 | 34 | 114 | 291 | 137 | 525 | 4 | 2 | 8 | 98 | 46 | 177 | 1 | 1 | 3 |
| 2015 | Guangdong | 19376 | 17690 | 31658 | 286 | 261 | 467 | 3404 | 2038 | 6870 | 50 | 30 | 101 | 255 | 120 | 461 | 4 | 2 | 7 | 86 | 41 | 155 | 1 | 1 | 2 |
| 2016 | Guangdong | 23722 | 21658 | 38759 | 347 | 317 | 567 | 4167 | 2495 | 8410 | 61 | 36 | 123 | 293 | 138 | 529 | 4 | 2 | 8 | 99 | 46 | 178 | 1 | 1 | 3 |
| 2017 | Guangdong | 22747 | 20769 | 37167 | 303 | 276 | 494 | 3996 | 2392 | 8065 | 53 | 32 | 107 | 254 | 119 | 458 | 3 | 2 | 6 | 86 | 40 | 155 | 1 | 1 | 2 |
| 2010 | Guangxi | 12972 | 11844 | 21195 | 368 | 336 | 601 | 2279 | 1364 | 4599 | 65 | 39 | 130 | 291 | 137 | 526 | 8 | 4 | 15 | 98 | 46 | 177 | 3 | 1 | 5 |
| 2011 | Guangxi | 12925 | 11801 | 21119 | 357 | 326 | 583 | 2270 | 1359 | 4583 | 63 | 38 | 127 | 283 | 133 | 511 | 8 | 4 | 14 | 95 | 45 | 172 | 3 | 1 | 5 |
| 2012 | Guangxi | 12248 | 11183 | 20012 | 335 | 306 | 547 | 2151 | 1288 | 4342 | 59 | 35 | 119 | 258 | 121 | 465 | 7 | 3 | 13 | 87 | 41 | 157 | 2 | 1 | 4 |
| 2013 | Guangxi | 11485 | 10486 | 18765 | 302 | 275 | 493 | 2017 | 1208 | 4072 | 53 | 32 | 107 | 229 | 108 | 413 | 6 | 3 | 11 | 77 | 36 | 139 | 2 | 1 | 4 |
| 2014 | Guangxi | 11533 | 10530 | 18845 | 296 | 270 | 484 | 2026 | 1213 | 4089 | 52 | 31 | 105 | 222 | 104 | 401 | 6 | 3 | 10 | 75 | 35 | 135 | 2 | 1 | 3 |
| 2015 | Guangxi | 10388 | 9485 | 16973 | 270 | 246 | 441 | 1825 | 1093 | 3683 | 47 | 28 | 96 | 190 | 90 | 344 | 5 | 2 | 9 | 64 | 30 | 116 | 2 | 1 | 3 |
| 2016 | Guangxi | 11371 | 10382 | 18579 | 291 | 265 | 475 | 1997 | 1196 | 4032 | 51 | 31 | 103 | 190 | 89 | 343 | 5 | 2 | 9 | 64 | 30 | 116 | 2 | 1 | 3 |
| 2017 | Guangxi | 10905 | 9957 | 17818 | 271 | 247 | 442 | 1916 | 1147 | 3866 | 48 | 28 | 96 | 162 | 76 | 293 | 4 | 2 | 7 | 55 | 26 | 99 | 1 | 1 | 2 |
| 2010 | Guizhou | 11996 | 10953 | 19601 | 488 | 446 | 797 | 2107 | 1262 | 4253 | 86 | 51 | 173 | 341 | 160 | 616 | 14 | 7 | 25 | 115 | 54 | 208 | 5 | 2 | 8 |
| 2011 | Guizhou | 11083 | 10119 | 18108 | 434 | 396 | 709 | 1947 | 1166 | 3929 | 76 | 46 | 154 | 293 | 138 | 529 | 11 | 5 | 21 | 99 | 46 | 178 | 4 | 2 | 7 |
| 2012 | Guizhou | 10217 | 9328 | 16693 | 406 | 371 | 663 | 1795 | 1075 | 3622 | 71 | 43 | 144 | 245 | 116 | 443 | 10 | 5 | 18 | 83 | 39 | 149 | 3 | 2 | 6 |
| 2013 | Guizhou | 9688 | 8846 | 15830 | 383 | 349 | 625 | 1702 | 1019 | 3435 | 67 | 40 | 136 | 210 | 99 | 379 | 8 | 4 | 15 | 71 | 33 | 128 | 3 | 1 | 5 |
| 2014 | Guizhou | 9226 | 8423 | 15074 | 357 | 326 | 583 | 1621 | 970 | 3271 | 63 | 38 | 126 | 179 | 84 | 324 | 7 | 3 | 13 | 60 | 28 | 109 | 2 | 1 | 4 |
| 2015 | Guizhou | 8194 | 7481 | 13388 | 320 | 292 | 522 | 1439 | 862 | 2905 | 56 | 34 | 113 | 142 | 67 | 256 | 6 | 3 | 10 | 48 | 23 | 86 | 2 | 1 | 3 |
| 2016 | Guizhou | 8660 | 7907 | 14149 | 345 | 315 | 564 | 1521 | 911 | 3070 | 61 | 36 | 122 | 129 | 61 | 233 | 5 | 2 | 9 | 44 | 21 | 79 | 2 | 1 | 3 |
| 2017 | Guizhou | 8060 | 7359 | 13170 | 285 | 261 | 466 | 1416 | 848 | 2858 | 50 | 30 | 101 | 104 | 49 | 189 | 4 | 2 | 7 | 35 | 17 | 64 | 1 | 1 | 2 |
| 2010 | Hainan | 3224 | 2944 | 5268 | 504 | 460 | 823 | 566 | 339 | 1143 | 89 | 53 | 179 | 113 | 53 | 203 | 18 | 8 | 32 | 38 | 18 | 69 | 6 | 3 | 11 |
| 2011 | Hainan | 2752 | 2513 | 4497 | 444 | 405 | 726 | 483 | 289 | 976 | 78 | 47 | 157 | 95 | 45 | 171 | 15 | 7 | 28 | 32 | 15 | 58 | 5 | 2 | 9 |
| 2012 | Hainan | 2544 | 2323 | 4157 | 395 | 360 | 645 | 447 | 268 | 902 | 69 | 42 | 140 | 86 | 41 | 156 | 13 | 6 | 24 | 29 | 14 | 53 | 5 | 2 | 8 |
| 2013 | Hainan | 2349 | 2144 | 3837 | 363 | 331 | 593 | 413 | 247 | 833 | 64 | 38 | 129 | 80 | 38 | 144 | 12 | 6 | 22 | 27 | 13 | 48 | 4 | 2 | 7 |
| 2014 | Hainan | 2408 | 2198 | 3934 | 369 | 337 | 603 | 423 | 253 | 854 | 65 | 39 | 131 | 81 | 38 | 147 | 12 | 6 | 23 | 27 | 13 | 50 | 4 | 2 | 8 |
| 2015 | Hainan | 2101 | 1919 | 3434 | 326 | 297 | 532 | 369 | 221 | 745 | 57 | 34 | 115 | 72 | 34 | 130 | 11 | 5 | 20 | 24 | 11 | 44 | 4 | 2 | 7 |
| 2016 | Hainan | 2341 | 2138 | 3825 | 361 | 330 | 590 | 411 | 246 | 830 | 63 | 38 | 128 | 77 | 36 | 139 | 12 | 6 | 21 | 26 | 12 | 47 | 4 | 2 | 7 |
| 2017 | Hainan | 2070 | 1890 | 3381 | 314 | 286 | 513 | 364 | 218 | 734 | 55 | 33 | 111 | 63 | 29 | 113 | 9 | 4 | 17 | 21 | 10 | 38 | 3 | 2 | 6 |
| 2010 | Hebei | 21223 | 19377 | 34676 | 421 | 385 | 689 | 3728 | 2232 | 7524 | 74 | 44 | 149 | 1034 | 487 | 1869 | 21 | 10 | 37 | 349 | 164 | 630 | 7 | 3 | 13 |
| 2011 | Hebei | 19841 | 18115 | 32418 | 413 | 377 | 674 | 3485 | 2087 | 7035 | 72 | 43 | 146 | 945 | 445 | 1708 | 20 | 9 | 36 | 319 | 150 | 576 | 7 | 3 | 12 |
| 2012 | Hebei | 19354 | 17671 | 31623 | 389 | 355 | 635 | 3400 | 2036 | 6862 | 68 | 41 | 138 | 890 | 419 | 1609 | 18 | 8 | 32 | 300 | 141 | 542 | 6 | 3 | 11 |
| 2013 | Hebei | 18973 | 17322 | 30999 | 372 | 340 | 608 | 3333 | 1995 | 6727 | 65 | 39 | 132 | 838 | 395 | 1514 | 16 | 8 | 30 | 283 | 133 | 510 | 6 | 3 | 10 |
| 2014 | Hebei | 18887 | 17244 | 30860 | 362 | 331 | 592 | 3318 | 1986 | 6696 | 64 | 38 | 129 | 821 | 387 | 1484 | 16 | 7 | 28 | 277 | 130 | 500 | 5 | 3 | 10 |
| 2015 | Hebei | 18159 | 16579 | 29670 | 360 | 329 | 588 | 3190 | 1910 | 6438 | 63 | 38 | 128 | 780 | 367 | 1409 | 15 | 7 | 28 | 263 | 124 | 475 | 5 | 2 | 9 |
| 2016 | Hebei | 19317 | 17637 | 31562 | 385 | 352 | 629 | 3393 | 2032 | 6849 | 68 | 41 | 137 | 782 | 368 | 1412 | 16 | 7 | 28 | 263 | 124 | 476 | 5 | 2 | 9 |
| 2017 | Hebei | 17463 | 15944 | 28533 | 337 | 308 | 551 | 3068 | 1837 | 6191 | 59 | 35 | 120 | 640 | 302 | 1157 | 12 | 6 | 22 | 216 | 102 | 390 | 4 | 2 | 8 |
| 2010 | Heilongjiang | 6830 | 6236 | 11160 | 336 | 307 | 549 | 1200 | 718 | 2422 | 59 | 35 | 119 | 240 | 113 | 433 | 12 | 6 | 21 | 81 | 38 | 146 | 4 | 2 | 7 |
| 2011 | Heilongjiang | 6137 | 5604 | 10028 | 384 | 350 | 627 | 1078 | 645 | 2176 | 67 | 40 | 136 | 213 | 100 | 385 | 13 | 6 | 24 | 72 | 34 | 130 | 4 | 2 | 8 |
| 2012 | Heilongjiang | 5446 | 4972 | 8898 | 404 | 369 | 660 | 957 | 573 | 1931 | 71 | 42 | 143 | 181 | 85 | 326 | 13 | 6 | 24 | 61 | 29 | 110 | 5 | 2 | 8 |
| 2013 | Heilongjiang | 5103 | 4659 | 8338 | 393 | 359 | 642 | 896 | 537 | 1809 | 69 | 41 | 139 | 160 | 76 | 290 | 12 | 6 | 22 | 54 | 25 | 98 | 4 | 2 | 8 |
| 2014 | Heilongjiang | 4933 | 4504 | 8060 | 373 | 341 | 610 | 867 | 519 | 1749 | 66 | 39 | 132 | 149 | 70 | 270 | 11 | 5 | 20 | 50 | 24 | 91 | 4 | 2 | 7 |
| 2015 | Heilongjiang | 4503 | 4112 | 7358 | 349 | 318 | 569 | 791 | 474 | 1597 | 61 | 37 | 124 | 129 | 61 | 233 | 10 | 5 | 18 | 43 | 20 | 79 | 3 | 2 | 6 |
| 2016 | Heilongjiang | 4883 | 4459 | 7979 | 384 | 350 | 627 | 858 | 514 | 1731 | 67 | 40 | 136 | 128 | 60 | 230 | 10 | 5 | 18 | 43 | 20 | 78 | 3 | 2 | 6 |
| 2017 | Heilongjiang | 4157 | 3795 | 6792 | 378 | 345 | 618 | 730 | 437 | 1474 | 66 | 40 | 134 | 98 | 46 | 177 | 9 | 4 | 16 | 33 | 16 | 60 | 3 | 1 | 5 |
| 2010 | Henan | 17711 | 16170 | 28938 | 270 | 247 | 442 | 3111 | 1863 | 6279 | 47 | 28 | 96 | 816 | 384 | 1474 | 12 | 6 | 22 | 275 | 129 | 497 | 4 | 2 | 8 |
| 2011 | Henan | 16528 | 15091 | 27006 | 228 | 208 | 373 | 2903 | 1738 | 5860 | 40 | 24 | 81 | 728 | 343 | 1316 | 10 | 5 | 18 | 246 | 116 | 444 | 3 | 2 | 6 |
| 2012 | Henan | 15495 | 14147 | 25318 | 209 | 191 | 341 | 2722 | 1630 | 5494 | 37 | 22 | 74 | 640 | 301 | 1156 | 9 | 4 | 16 | 216 | 102 | 390 | 3 | 1 | 5 |
| 2013 | Henan | 15387 | 14048 | 25141 | 206 | 189 | 337 | 2703 | 1618 | 5455 | 36 | 22 | 73 | 589 | 277 | 1065 | 8 | 4 | 14 | 199 | 94 | 359 | 3 | 1 | 5 |
| 2014 | Henan | 14921 | 13623 | 24380 | 202 | 185 | 330 | 2621 | 1569 | 5290 | 36 | 21 | 72 | 534 | 252 | 965 | 7 | 3 | 13 | 180 | 85 | 325 | 2 | 1 | 4 |
| 2015 | Henan | 14203 | 12967 | 23206 | 194 | 177 | 316 | 2495 | 1494 | 5036 | 34 | 20 | 69 | 472 | 222 | 853 | 6 | 3 | 12 | 159 | 75 | 288 | 2 | 1 | 4 |
| 2016 | Henan | 16412 | 14984 | 26815 | 222 | 203 | 363 | 2883 | 1726 | 5819 | 39 | 23 | 79 | 478 | 225 | 864 | 6 | 3 | 12 | 161 | 76 | 291 | 2 | 1 | 4 |
| 2017 | Henan | 14348 | 13100 | 23444 | 193 | 177 | 316 | 2520 | 1509 | 5087 | 34 | 20 | 69 | 362 | 170 | 653 | 5 | 2 | 9 | 122 | 57 | 220 | 2 | 1 | 3 |
| 2010 | Hubei | 6835 | 6240 | 11167 | 192 | 176 | 315 | 1201 | 719 | 2423 | 34 | 20 | 68 | 147 | 69 | 266 | 4 | 2 | 7 | 50 | 23 | 90 | 1 | 1 | 3 |
| 2011 | Hubei | 6029 | 5504 | 9850 | 196 | 179 | 320 | 1059 | 634 | 2137 | 34 | 21 | 69 | 125 | 59 | 226 | 4 | 2 | 7 | 42 | 20 | 76 | 1 | 1 | 2 |
| 2012 | Hubei | 5415 | 4944 | 8847 | 180 | 165 | 295 | 951 | 569 | 1920 | 32 | 19 | 64 | 105 | 49 | 190 | 3 | 2 | 6 | 35 | 17 | 64 | 1 | 1 | 2 |
| 2013 | Hubei | 5312 | 4850 | 8680 | 174 | 159 | 285 | 933 | 559 | 1884 | 31 | 18 | 62 | 94 | 44 | 170 | 3 | 1 | 6 | 32 | 15 | 57 | 1 | 0 | 2 |
| 2014 | Hubei | 5584 | 5099 | 9124 | 179 | 164 | 293 | 981 | 587 | 1980 | 31 | 19 | 64 | 90 | 42 | 162 | 3 | 1 | 5 | 30 | 14 | 55 | 1 | 0 | 2 |
| 2015 | Hubei | 5414 | 4943 | 8845 | 175 | 159 | 285 | 951 | 569 | 1919 | 31 | 18 | 62 | 78 | 37 | 141 | 3 | 1 | 5 | 26 | 12 | 48 | 1 | 0 | 2 |
| 2016 | Hubei | 6405 | 5848 | 10465 | 209 | 191 | 342 | 1125 | 674 | 2271 | 37 | 22 | 74 | 79 | 37 | 143 | 3 | 1 | 5 | 27 | 13 | 48 | 1 | 0 | 2 |
| 2017 | Hubei | 6126 | 5593 | 10009 | 190 | 173 | 310 | 1076 | 644 | 2172 | 33 | 20 | 67 | 64 | 30 | 115 | 2 | 1 | 4 | 21 | 10 | 39 | 1 | 0 | 1 |
| 2010 | Hunan | 12915 | 11791 | 21102 | 314 | 287 | 513 | 2269 | 1358 | 4579 | 55 | 33 | 111 | 220 | 103 | 397 | 5 | 3 | 10 | 74 | 35 | 134 | 2 | 1 | 3 |
| 2011 | Hunan | 11178 | 10206 | 18263 | 265 | 242 | 433 | 1963 | 1176 | 3963 | 47 | 28 | 94 | 178 | 84 | 322 | 4 | 2 | 8 | 60 | 28 | 109 | 1 | 1 | 3 |
| 2012 | Hunan | 11160 | 10190 | 18235 | 270 | 246 | 440 | 1960 | 1174 | 3957 | 47 | 28 | 96 | 163 | 77 | 295 | 4 | 2 | 7 | 55 | 26 | 100 | 1 | 1 | 2 |
| 2013 | Hunan | 10358 | 9457 | 16924 | 247 | 225 | 403 | 1819 | 1089 | 3672 | 43 | 26 | 87 | 137 | 64 | 247 | 3 | 2 | 6 | 46 | 22 | 83 | 1 | 1 | 2 |
| 2014 | Hunan | 10625 | 9701 | 17360 | 248 | 226 | 405 | 1866 | 1118 | 3767 | 44 | 26 | 88 | 128 | 60 | 231 | 3 | 1 | 5 | 43 | 20 | 78 | 1 | 0 | 2 |
| 2015 | Hunan | 10318 | 9420 | 16858 | 240 | 219 | 392 | 1812 | 1085 | 3658 | 42 | 25 | 85 | 111 | 52 | 201 | 3 | 1 | 5 | 38 | 18 | 68 | 1 | 0 | 2 |
| 2016 | Hunan | 11504 | 10504 | 18797 | 267 | 244 | 437 | 2021 | 1210 | 4079 | 47 | 28 | 95 | 105 | 50 | 191 | 2 | 1 | 4 | 36 | 17 | 64 | 1 | 0 | 1 |
| 2017 | Hunan | 11036 | 10076 | 18032 | 249 | 227 | 406 | 1939 | 1161 | 3913 | 44 | 26 | 88 | 85 | 40 | 154 | 2 | 1 | 3 | 29 | 14 | 52 | 1 | 0 | 1 |
| 2010 | Inner Mongolia | 6663 | 6084 | 10887 | 467 | 426 | 762 | 1170 | 701 | 2362 | 82 | 49 | 165 | 266 | 125 | 481 | 19 | 9 | 34 | 90 | 42 | 162 | 6 | 3 | 11 |
| 2011 | Inner Mongolia | 6299 | 5751 | 10292 | 523 | 477 | 854 | 1106 | 662 | 2233 | 92 | 55 | 185 | 249 | 117 | 450 | 21 | 10 | 37 | 84 | 40 | 152 | 7 | 3 | 13 |
| 2012 | Inner Mongolia | 5692 | 5197 | 9301 | 509 | 464 | 831 | 1000 | 599 | 2018 | 89 | 53 | 180 | 222 | 105 | 401 | 20 | 9 | 36 | 75 | 35 | 135 | 7 | 3 | 12 |
| 2013 | Inner Mongolia | 5448 | 4974 | 8901 | 487 | 445 | 796 | 957 | 573 | 1931 | 86 | 51 | 173 | 210 | 99 | 379 | 19 | 9 | 34 | 71 | 33 | 128 | 6 | 3 | 11 |
| 2014 | Inner Mongolia | 5502 | 5023 | 8989 | 482 | 440 | 788 | 966 | 579 | 1951 | 85 | 51 | 171 | 212 | 100 | 383 | 19 | 9 | 34 | 71 | 34 | 129 | 6 | 3 | 11 |
| 2015 | Inner Mongolia | 5172 | 4722 | 8451 | 461 | 420 | 752 | 909 | 544 | 1834 | 81 | 48 | 163 | 199 | 93 | 359 | 18 | 8 | 32 | 67 | 32 | 121 | 6 | 3 | 11 |
| 2016 | Inner Mongolia | 5436 | 4963 | 8881 | 487 | 444 | 795 | 955 | 572 | 1927 | 86 | 51 | 173 | 197 | 93 | 357 | 18 | 8 | 32 | 67 | 31 | 120 | 6 | 3 | 11 |
| 2017 | Inner Mongolia | 5060 | 4620 | 8267 | 482 | 440 | 787 | 889 | 532 | 1794 | 85 | 51 | 171 | 169 | 80 | 305 | 16 | 8 | 29 | 57 | 27 | 103 | 5 | 3 | 10 |
| 2010 | Jiangsu | 9584 | 8751 | 15660 | 215 | 196 | 351 | 1684 | 1008 | 3398 | 38 | 23 | 76 | 128 | 60 | 230 | 3 | 1 | 5 | 43 | 20 | 78 | 1 | 0 | 2 |
| 2011 | Jiangsu | 9474 | 8650 | 15480 | 245 | 223 | 400 | 1664 | 996 | 3359 | 43 | 26 | 87 | 119 | 56 | 215 | 3 | 1 | 6 | 40 | 19 | 72 | 1 | 0 | 2 |
| 2012 | Jiangsu | 10459 | 9550 | 17090 | 264 | 241 | 432 | 1837 | 1100 | 3708 | 46 | 28 | 94 | 121 | 57 | 219 | 3 | 1 | 6 | 41 | 19 | 74 | 1 | 0 | 2 |
| 2013 | Jiangsu | 11094 | 10129 | 18126 | 283 | 259 | 463 | 1949 | 1167 | 3933 | 50 | 30 | 101 | 118 | 55 | 213 | 3 | 1 | 5 | 40 | 19 | 72 | 1 | 0 | 2 |
| 2014 | Jiangsu | 11483 | 10485 | 18763 | 291 | 265 | 475 | 2017 | 1208 | 4071 | 51 | 31 | 103 | 112 | 53 | 203 | 3 | 1 | 5 | 38 | 18 | 68 | 1 | 0 | 2 |
| 2015 | Jiangsu | 11545 | 10541 | 18863 | 295 | 269 | 482 | 2028 | 1214 | 4093 | 52 | 31 | 105 | 103 | 48 | 186 | 3 | 1 | 5 | 35 | 16 | 63 | 1 | 0 | 2 |
| 2016 | Jiangsu | 13104 | 11964 | 21411 | 335 | 306 | 547 | 2302 | 1378 | 4646 | 59 | 35 | 119 | 99 | 47 | 179 | 3 | 1 | 5 | 33 | 16 | 60 | 1 | 0 | 2 |
| 2017 | Jiangsu | 13543 | 12365 | 22128 | 354 | 323 | 578 | 2379 | 1424 | 4802 | 62 | 37 | 125 | 86 | 40 | 155 | 2 | 1 | 4 | 29 | 14 | 52 | 1 | 0 | 1 |
| 2010 | Jiangxi | 14098 | 12872 | 23035 | 467 | 427 | 764 | 2476 | 1483 | 4998 | 82 | 49 | 166 | 661 | 311 | 1195 | 22 | 10 | 40 | 223 | 105 | 403 | 7 | 3 | 13 |
| 2011 | Jiangxi | 12568 | 11475 | 20535 | 387 | 353 | 632 | 2208 | 1322 | 4456 | 68 | 41 | 137 | 568 | 267 | 1025 | 17 | 8 | 32 | 191 | 90 | 346 | 6 | 3 | 11 |
| 2012 | Jiangxi | 14181 | 12948 | 23171 | 412 | 376 | 673 | 2491 | 1492 | 5028 | 72 | 43 | 146 | 613 | 289 | 1107 | 18 | 8 | 32 | 207 | 97 | 373 | 6 | 3 | 11 |
| 2013 | Jiangxi | 13031 | 11897 | 21291 | 384 | 350 | 627 | 2289 | 1371 | 4620 | 67 | 40 | 136 | 549 | 259 | 992 | 16 | 8 | 29 | 185 | 87 | 334 | 5 | 3 | 10 |
| 2014 | Jiangxi | 9626 | 8789 | 15728 | 287 | 262 | 470 | 1691 | 1012 | 3413 | 50 | 30 | 102 | 404 | 190 | 731 | 12 | 6 | 22 | 136 | 64 | 246 | 4 | 2 | 7 |
| 2015 | Jiangxi | 8537 | 7795 | 13949 | 257 | 235 | 420 | 1500 | 898 | 3027 | 45 | 27 | 91 | 357 | 168 | 645 | 11 | 5 | 19 | 120 | 57 | 217 | 4 | 2 | 7 |
| 2016 | Jiangxi | 9182 | 8383 | 15002 | 279 | 255 | 456 | 1613 | 966 | 3255 | 49 | 29 | 99 | 369 | 174 | 666 | 11 | 5 | 20 | 124 | 59 | 225 | 4 | 2 | 7 |
| 2017 | Jiangxi | 7900 | 7213 | 12908 | 253 | 231 | 413 | 1388 | 831 | 2801 | 44 | 27 | 90 | 295 | 139 | 533 | 9 | 4 | 17 | 99 | 47 | 180 | 3 | 1 | 6 |
| 2010 | Jilin | 6669 | 6089 | 10896 | 456 | 416 | 744 | 1171 | 701 | 2364 | 80 | 48 | 162 | 235 | 111 | 425 | 16 | 8 | 29 | 79 | 37 | 143 | 5 | 3 | 10 |
| 2011 | Jilin | 6084 | 5554 | 9940 | 512 | 467 | 836 | 1069 | 640 | 2157 | 90 | 54 | 181 | 212 | 100 | 383 | 18 | 8 | 32 | 71 | 34 | 129 | 6 | 3 | 11 |
| 2012 | Jilin | 5689 | 5194 | 9295 | 538 | 491 | 879 | 999 | 598 | 2017 | 95 | 57 | 191 | 193 | 91 | 349 | 18 | 9 | 33 | 65 | 31 | 118 | 6 | 3 | 11 |
| 2013 | Jilin | 5300 | 4839 | 8660 | 509 | 465 | 832 | 931 | 557 | 1879 | 89 | 54 | 181 | 177 | 84 | 320 | 17 | 8 | 31 | 60 | 28 | 108 | 6 | 3 | 10 |
| 2014 | Jilin | 5080 | 4639 | 8301 | 488 | 446 | 798 | 892 | 534 | 1801 | 86 | 51 | 173 | 169 | 80 | 306 | 16 | 8 | 29 | 57 | 27 | 103 | 5 | 3 | 10 |
| 2015 | Jilin | 4602 | 4202 | 7519 | 453 | 414 | 740 | 808 | 484 | 1632 | 80 | 48 | 161 | 151 | 71 | 273 | 15 | 7 | 27 | 51 | 24 | 92 | 5 | 2 | 9 |
| 2016 | Jilin | 5097 | 4654 | 8328 | 505 | 461 | 825 | 895 | 536 | 1807 | 89 | 53 | 179 | 156 | 73 | 281 | 15 | 7 | 28 | 52 | 25 | 95 | 5 | 2 | 9 |
| 2017 | Jilin | 4419 | 4034 | 7219 | 463 | 423 | 756 | 776 | 465 | 1567 | 81 | 49 | 164 | 123 | 58 | 221 | 13 | 6 | 23 | 41 | 19 | 75 | 4 | 2 | 8 |
| 2010 | Liaoning | 6881 | 6283 | 11243 | 325 | 297 | 531 | 1209 | 724 | 2440 | 57 | 34 | 115 | 98 | 46 | 177 | 5 | 2 | 8 | 33 | 16 | 59 | 2 | 1 | 3 |
| 2011 | Liaoning | 6496 | 5931 | 10614 | 358 | 327 | 585 | 1141 | 683 | 2303 | 63 | 38 | 127 | 88 | 41 | 159 | 5 | 2 | 9 | 30 | 14 | 54 | 2 | 1 | 3 |
| 2012 | Liaoning | 6330 | 5780 | 10343 | 411 | 375 | 671 | 1112 | 666 | 2244 | 72 | 43 | 146 | 80 | 38 | 145 | 5 | 2 | 9 | 27 | 13 | 49 | 2 | 1 | 3 |
| 2013 | Liaoning | 6445 | 5884 | 10530 | 422 | 385 | 690 | 1132 | 678 | 2285 | 74 | 44 | 150 | 76 | 36 | 137 | 5 | 2 | 9 | 26 | 12 | 46 | 2 | 1 | 3 |
| 2014 | Liaoning | 6298 | 5751 | 10291 | 405 | 370 | 662 | 1106 | 662 | 2233 | 71 | 43 | 144 | 69 | 32 | 125 | 4 | 2 | 8 | 23 | 11 | 42 | 1 | 1 | 3 |
| 2015 | Liaoning | 6058 | 5531 | 9897 | 402 | 367 | 657 | 1064 | 637 | 2148 | 71 | 42 | 143 | 61 | 29 | 110 | 4 | 2 | 7 | 20 | 10 | 37 | 1 | 1 | 2 |
| 2016 | Liaoning | 6475 | 5912 | 10579 | 429 | 391 | 700 | 1137 | 681 | 2296 | 75 | 45 | 152 | 56 | 27 | 102 | 4 | 2 | 7 | 19 | 9 | 34 | 1 | 1 | 2 |
| 2017 | Liaoning | 6228 | 5687 | 10176 | 443 | 404 | 724 | 1094 | 655 | 2208 | 78 | 47 | 157 | 46 | 22 | 84 | 3 | 2 | 6 | 16 | 7 | 28 | 1 | 1 | 2 |
| 2010 | Ningxia | 2553 | 2331 | 4172 | 577 | 527 | 943 | 448 | 269 | 905 | 101 | 61 | 205 | 112 | 53 | 202 | 25 | 12 | 46 | 38 | 18 | 68 | 9 | 4 | 15 |
| 2011 | Ningxia | 2405 | 2196 | 3929 | 549 | 501 | 897 | 422 | 253 | 853 | 96 | 58 | 195 | 97 | 46 | 176 | 22 | 10 | 40 | 33 | 15 | 59 | 7 | 4 | 14 |
| 2012 | Ningxia | 2323 | 2121 | 3795 | 534 | 488 | 873 | 408 | 244 | 823 | 94 | 56 | 189 | 93 | 44 | 168 | 21 | 10 | 39 | 31 | 15 | 57 | 7 | 3 | 13 |
| 2013 | Ningxia | 2219 | 2026 | 3626 | 508 | 464 | 830 | 390 | 233 | 787 | 89 | 53 | 180 | 86 | 40 | 155 | 20 | 9 | 36 | 29 | 14 | 52 | 7 | 3 | 12 |
| 2014 | Ningxia | 2051 | 1873 | 3351 | 439 | 401 | 718 | 360 | 216 | 727 | 77 | 46 | 156 | 78 | 37 | 141 | 17 | 8 | 30 | 26 | 12 | 48 | 6 | 3 | 10 |
| 2015 | Ningxia | 1986 | 1813 | 3245 | 440 | 402 | 719 | 349 | 209 | 704 | 77 | 46 | 156 | 75 | 35 | 136 | 17 | 8 | 30 | 25 | 12 | 46 | 6 | 3 | 10 |
| 2016 | Ningxia | 1988 | 1815 | 3248 | 440 | 402 | 720 | 349 | 209 | 705 | 77 | 46 | 156 | 71 | 34 | 129 | 16 | 7 | 29 | 24 | 11 | 43 | 5 | 3 | 10 |
| 2017 | Ningxia | 1869 | 1707 | 3054 | 414 | 378 | 677 | 328 | 197 | 663 | 73 | 44 | 147 | 61 | 29 | 110 | 14 | 6 | 24 | 21 | 10 | 37 | 5 | 2 | 8 |
| 2010 | Qinghai | 2928 | 2673 | 4784 | 803 | 733 | 1311 | 514 | 308 | 1038 | 141 | 84 | 285 | 115 | 59 | 206 | 32 | 16 | 56 | 39 | 20 | 69 | 11 | 5 | 19 |
| 2011 | Qinghai | 2794 | 2551 | 4565 | 768 | 701 | 1254 | 491 | 294 | 991 | 135 | 81 | 272 | 134 | 48 | 186 | 37 | 13 | 51 | 45 | 16 | 63 | 12 | 4 | 17 |
| 2012 | Qinghai | 2583 | 2358 | 4220 | 718 | 656 | 1173 | 454 | 272 | 916 | 126 | 76 | 255 | 142 | 55 | 213 | 39 | 15 | 59 | 48 | 19 | 72 | 13 | 5 | 20 |
| 2013 | Qinghai | 2437 | 2225 | 3982 | 695 | 635 | 1136 | 428 | 256 | 864 | 122 | 73 | 246 | 151 | 64 | 245 | 43 | 18 | 70 | 51 | 22 | 83 | 14 | 6 | 24 |
| 2014 | Qinghai | 2255 | 2059 | 3684 | 606 | 554 | 991 | 396 | 237 | 799 | 107 | 64 | 215 | 159 | 75 | 286 | 43 | 20 | 77 | 53 | 25 | 97 | 14 | 7 | 26 |
| 2015 | Qinghai | 1993 | 1819 | 3256 | 537 | 490 | 878 | 350 | 210 | 707 | 94 | 56 | 190 | 135 | 64 | 245 | 36 | 17 | 66 | 46 | 21 | 82 | 12 | 6 | 22 |
| 2016 | Qinghai | 1944 | 1775 | 3177 | 518 | 473 | 847 | 342 | 205 | 689 | 91 | 55 | 184 | 127 | 60 | 229 | 34 | 16 | 61 | 43 | 20 | 77 | 11 | 5 | 21 |
| 2017 | Qinghai | 1829 | 1670 | 2988 | 486 | 443 | 793 | 321 | 192 | 648 | 85 | 51 | 172 | 109 | 51 | 197 | 29 | 14 | 52 | 37 | 17 | 67 | 10 | 5 | 18 |
| 2010 | Shaanxi | 11085 | 10120 | 18111 | 486 | 444 | 795 | 1947 | 1166 | 3930 | 85 | 51 | 172 | 553 | 261 | 1000 | 24 | 11 | 44 | 187 | 88 | 337 | 8 | 4 | 15 |
| 2011 | Shaanxi | 11071 | 10108 | 18088 | 539 | 492 | 881 | 1945 | 1164 | 3925 | 95 | 57 | 191 | 530 | 250 | 957 | 26 | 12 | 47 | 179 | 84 | 323 | 9 | 4 | 16 |
| 2012 | Shaanxi | 10907 | 9958 | 17821 | 561 | 513 | 917 | 1916 | 1147 | 3867 | 99 | 59 | 199 | 505 | 238 | 913 | 26 | 12 | 47 | 170 | 80 | 308 | 9 | 4 | 16 |
| 2013 | Shaanxi | 9516 | 8688 | 15548 | 480 | 438 | 785 | 1672 | 1001 | 3374 | 84 | 51 | 170 | 430 | 202 | 776 | 22 | 10 | 39 | 145 | 68 | 262 | 7 | 3 | 13 |
| 2014 | Shaanxi | 8996 | 8213 | 14698 | 435 | 397 | 711 | 1580 | 946 | 3189 | 76 | 46 | 154 | 408 | 192 | 736 | 20 | 9 | 36 | 137 | 65 | 248 | 7 | 3 | 12 |
| 2015 | Shaanxi | 8162 | 7452 | 13336 | 399 | 364 | 652 | 1434 | 858 | 2894 | 70 | 42 | 141 | 375 | 176 | 677 | 18 | 9 | 33 | 126 | 59 | 228 | 6 | 3 | 11 |
| 2016 | Shaanxi | 8196 | 7483 | 13391 | 404 | 369 | 660 | 1440 | 862 | 2906 | 71 | 42 | 143 | 362 | 170 | 654 | 18 | 8 | 32 | 122 | 57 | 220 | 6 | 3 | 11 |
| 2017 | Shaanxi | 7257 | 6626 | 11858 | 363 | 331 | 593 | 1275 | 763 | 2573 | 64 | 38 | 129 | 298 | 141 | 539 | 15 | 7 | 27 | 101 | 47 | 182 | 5 | 2 | 9 |
| 2010 | Shandong | 10802 | 9863 | 17650 | 180 | 164 | 294 | 1898 | 1136 | 3830 | 32 | 19 | 64 | 301 | 142 | 543 | 5 | 2 | 9 | 101 | 48 | 183 | 2 | 1 | 3 |
| 2011 | Shandong | 9757 | 8909 | 15943 | 173 | 158 | 282 | 1714 | 1026 | 3459 | 30 | 18 | 61 | 258 | 122 | 467 | 5 | 2 | 8 | 87 | 41 | 157 | 2 | 1 | 3 |
| 2012 | Shandong | 9221 | 8419 | 15067 | 170 | 155 | 278 | 1620 | 970 | 3269 | 30 | 18 | 60 | 225 | 106 | 406 | 4 | 2 | 7 | 76 | 36 | 137 | 1 | 1 | 3 |
| 2013 | Shandong | 9278 | 8471 | 15159 | 171 | 156 | 279 | 1630 | 976 | 3290 | 30 | 18 | 61 | 204 | 96 | 369 | 4 | 2 | 7 | 69 | 32 | 124 | 1 | 1 | 2 |
| 2014 | Shandong | 9862 | 9004 | 16114 | 173 | 158 | 283 | 1732 | 1037 | 3497 | 30 | 18 | 61 | 195 | 92 | 353 | 3 | 2 | 6 | 66 | 31 | 119 | 1 | 1 | 2 |
| 2015 | Shandong | 9299 | 8490 | 15194 | 163 | 149 | 267 | 1633 | 978 | 3297 | 29 | 17 | 58 | 162 | 76 | 293 | 3 | 1 | 5 | 55 | 26 | 99 | 1 | 0 | 2 |
| 2016 | Shandong | 13144 | 12001 | 21476 | 223 | 204 | 365 | 2309 | 1382 | 4660 | 39 | 24 | 79 | 188 | 89 | 340 | 3 | 2 | 6 | 64 | 30 | 115 | 1 | 1 | 2 |
| 2017 | Shandong | 13782 | 12584 | 22519 | 227 | 207 | 371 | 2421 | 1450 | 4886 | 40 | 24 | 80 | 160 | 75 | 289 | 3 | 1 | 5 | 54 | 25 | 97 | 1 | 0 | 2 |
| 2010 | Shanghai | 2816 | 2571 | 4601 | 324 | 296 | 529 | 495 | 296 | 998 | 57 | 34 | 115 | 24 | 11 | 43 | 3 | 1 | 5 | 8 | 4 | 14 | 1 | 0 | 2 |
| 2011 | Shanghai | 2808 | 2564 | 4589 | 328 | 300 | 536 | 493 | 295 | 996 | 58 | 35 | 116 | 24 | 12 | 44 | 3 | 1 | 5 | 8 | 4 | 15 | 1 | 0 | 2 |
| 2012 | Shanghai | 3298 | 3011 | 5388 | 361 | 330 | 590 | 579 | 347 | 1169 | 63 | 38 | 128 | 29 | 14 | 53 | 3 | 2 | 6 | 10 | 5 | 18 | 1 | 1 | 2 |
| 2013 | Shanghai | 3200 | 2921 | 5228 | 342 | 312 | 558 | 562 | 337 | 1134 | 60 | 36 | 121 | 29 | 14 | 52 | 3 | 1 | 6 | 10 | 5 | 18 | 1 | 0 | 2 |
| 2014 | Shanghai | 1479 | 1350 | 2416 | 155 | 141 | 253 | 260 | 156 | 524 | 27 | 16 | 55 | 14 | 6 | 25 | 1 | 1 | 3 | 5 | 2 | 8 | 0 | 0 | 1 |
| 2015 | Shanghai | 1167 | 1066 | 1907 | 125 | 114 | 204 | 205 | 123 | 414 | 22 | 13 | 44 | 11 | 5 | 19 | 1 | 1 | 2 | 4 | 2 | 7 | 0 | 0 | 1 |
| 2016 | Shanghai | 1510 | 1379 | 2467 | 159 | 146 | 260 | 265 | 159 | 535 | 28 | 17 | 57 | 13 | 6 | 24 | 1 | 1 | 3 | 5 | 2 | 8 | 0 | 0 | 1 |
| 2017 | Shanghai | 1511 | 1379 | 2468 | 158 | 144 | 258 | 265 | 159 | 536 | 28 | 17 | 56 | 12 | 6 | 22 | 1 | 1 | 2 | 4 | 2 | 7 | 0 | 0 | 1 |
| 2010 | Shanxi | 8394 | 7664 | 13716 | 398 | 363 | 650 | 1475 | 883 | 2976 | 70 | 42 | 141 | 464 | 218 | 838 | 22 | 10 | 40 | 156 | 74 | 282 | 7 | 3 | 13 |
| 2011 | Shanxi | 7974 | 7280 | 13028 | 400 | 366 | 654 | 1401 | 839 | 2827 | 70 | 42 | 142 | 433 | 204 | 782 | 22 | 10 | 39 | 146 | 69 | 264 | 7 | 3 | 13 |
| 2012 | Shanxi | 7759 | 7085 | 12678 | 420 | 384 | 686 | 1363 | 816 | 2751 | 74 | 44 | 149 | 409 | 193 | 739 | 22 | 10 | 40 | 138 | 65 | 249 | 7 | 4 | 13 |
| 2013 | Shanxi | 7496 | 6844 | 12248 | 401 | 366 | 655 | 1317 | 788 | 2658 | 70 | 42 | 142 | 381 | 179 | 688 | 20 | 10 | 37 | 128 | 60 | 232 | 7 | 3 | 12 |
| 2014 | Shanxi | 7424 | 6779 | 12131 | 381 | 348 | 623 | 1304 | 781 | 2632 | 67 | 40 | 135 | 370 | 174 | 668 | 19 | 9 | 34 | 125 | 59 | 225 | 6 | 3 | 12 |
| 2015 | Shanxi | 7123 | 6504 | 11639 | 372 | 340 | 608 | 1251 | 749 | 2526 | 65 | 39 | 132 | 346 | 163 | 626 | 18 | 9 | 33 | 117 | 55 | 211 | 6 | 3 | 11 |
| 2016 | Shanxi | 7414 | 6769 | 12113 | 390 | 356 | 637 | 1302 | 780 | 2628 | 68 | 41 | 138 | 329 | 155 | 594 | 17 | 8 | 31 | 111 | 52 | 200 | 6 | 3 | 11 |
| 2017 | Shanxi | 7098 | 6481 | 11598 | 384 | 351 | 627 | 1247 | 747 | 2517 | 67 | 40 | 136 | 274 | 129 | 494 | 15 | 7 | 27 | 92 | 43 | 167 | 5 | 2 | 9 |
| 2010 | Sichuan | 24327 | 22211 | 39748 | 529 | 483 | 865 | 4273 | 2559 | 8625 | 93 | 56 | 188 | 680 | 320 | 1228 | 15 | 7 | 27 | 229 | 108 | 414 | 5 | 2 | 9 |
| 2011 | Sichuan | 23575 | 21524 | 38519 | 553 | 505 | 904 | 4141 | 2480 | 8358 | 97 | 58 | 196 | 620 | 292 | 1119 | 15 | 7 | 26 | 209 | 98 | 377 | 5 | 2 | 9 |
| 2012 | Sichuan | 20934 | 19113 | 34204 | 495 | 452 | 809 | 3677 | 2202 | 7422 | 87 | 52 | 175 | 515 | 242 | 930 | 12 | 6 | 22 | 174 | 82 | 313 | 4 | 2 | 7 |
| 2013 | Sichuan | 19252 | 17577 | 31455 | 466 | 426 | 762 | 3382 | 2025 | 6826 | 82 | 49 | 165 | 443 | 209 | 801 | 11 | 5 | 19 | 149 | 70 | 270 | 4 | 2 | 7 |
| 2014 | Sichuan | 17716 | 16175 | 28946 | 429 | 392 | 701 | 3112 | 1863 | 6281 | 75 | 45 | 152 | 390 | 184 | 704 | 9 | 4 | 17 | 131 | 62 | 237 | 3 | 1 | 6 |
| 2015 | Sichuan | 14367 | 13118 | 23475 | 350 | 319 | 572 | 2524 | 1511 | 5094 | 61 | 37 | 124 | 304 | 143 | 549 | 7 | 3 | 13 | 102 | 48 | 185 | 2 | 1 | 5 |
| 2016 | Sichuan | 15413 | 14072 | 25183 | 373 | 340 | 609 | 2707 | 1621 | 5465 | 66 | 39 | 132 | 299 | 141 | 540 | 7 | 3 | 13 | 101 | 47 | 182 | 2 | 1 | 4 |
| 2017 | Sichuan | 12150 | 11094 | 19853 | 292 | 267 | 478 | 2134 | 1278 | 4308 | 51 | 31 | 104 | 213 | 100 | 385 | 5 | 2 | 9 | 72 | 34 | 130 | 2 | 1 | 3 |
| 2010 | Tianjin | 2235 | 2040 | 3651 | 353 | 322 | 577 | 393 | 235 | 792 | 62 | 37 | 125 | 34 | 16 | 61 | 5 | 3 | 10 | 11 | 5 | 21 | 2 | 1 | 3 |
| 2011 | Tianjin | 1638 | 1496 | 2677 | 325 | 297 | 531 | 288 | 172 | 581 | 57 | 34 | 115 | 25 | 12 | 45 | 5 | 2 | 9 | 8 | 4 | 15 | 2 | 1 | 3 |
| 2012 | Tianjin | 1743 | 1591 | 2848 | 337 | 307 | 550 | 306 | 183 | 618 | 59 | 35 | 119 | 26 | 12 | 48 | 5 | 2 | 9 | 9 | 4 | 16 | 2 | 1 | 3 |
| 2013 | Tianjin | 1354 | 1236 | 2212 | 251 | 229 | 410 | 238 | 142 | 480 | 44 | 26 | 89 | 20 | 10 | 37 | 4 | 2 | 7 | 7 | 3 | 12 | 1 | 1 | 2 |
| 2014 | Tianjin | 1354 | 1236 | 2212 | 238 | 217 | 388 | 238 | 142 | 480 | 42 | 25 | 84 | 21 | 10 | 38 | 4 | 2 | 7 | 7 | 3 | 13 | 1 | 1 | 2 |
| 2015 | Tianjin | 1127 | 1029 | 1841 | 204 | 186 | 333 | 198 | 119 | 400 | 36 | 21 | 72 | 18 | 8 | 32 | 3 | 1 | 6 | 6 | 3 | 11 | 1 | 1 | 2 |
| 2016 | Tianjin | 1771 | 1617 | 2894 | 306 | 279 | 500 | 311 | 186 | 628 | 54 | 32 | 108 | 26 | 12 | 48 | 5 | 2 | 8 | 9 | 4 | 16 | 2 | 1 | 3 |
| 2017 | Tianjin | 1451 | 1325 | 2371 | 252 | 230 | 411 | 255 | 153 | 514 | 44 | 26 | 89 | 20 | 9 | 36 | 3 | 2 | 6 | 7 | 3 | 12 | 1 | 1 | 2 |
| 2010 | Tibet | 2522 | 2303 | 4121 | 1304 | 1191 | 2131 | 443 | 265 | 894 | 229 | 137 | 462 | 45 | 21 | 72 | 23 | 11 | 37 | 15 | 7 | 24 | 8 | 4 | 12 |
| 2011 | Tibet | 2470 | 2255 | 4036 | 1286 | 1174 | 2101 | 434 | 260 | 876 | 226 | 135 | 456 | 47 | 22 | 72 | 24 | 11 | 38 | 16 | 7 | 24 | 8 | 4 | 13 |
| 2012 | Tibet | 2374 | 2167 | 3878 | 1014 | 926 | 1656 | 417 | 250 | 842 | 178 | 107 | 359 | 47 | 23 | 74 | 20 | 10 | 31 | 16 | 8 | 25 | 7 | 3 | 11 |
| 2013 | Tibet | 2266 | 2069 | 3703 | 1113 | 1016 | 1818 | 398 | 238 | 804 | 195 | 117 | 395 | 47 | 23 | 73 | 23 | 11 | 36 | 16 | 8 | 25 | 8 | 4 | 12 |
| 2014 | Tibet | 2148 | 1961 | 3509 | 872 | 797 | 1425 | 377 | 226 | 762 | 153 | 92 | 309 | 46 | 23 | 72 | 18 | 9 | 29 | 15 | 8 | 24 | 6 | 3 | 10 |
| 2015 | Tibet | 1978 | 1806 | 3231 | 799 | 730 | 1306 | 347 | 208 | 701 | 140 | 84 | 283 | 42 | 20 | 65 | 17 | 8 | 26 | 14 | 7 | 22 | 6 | 3 | 9 |
| 2016 | Tibet | 1864 | 1702 | 3045 | 718 | 656 | 1173 | 327 | 196 | 661 | 126 | 76 | 255 | 37 | 17 | 58 | 14 | 7 | 22 | 12 | 6 | 19 | 5 | 2 | 7 |
| 2017 | Tibet | 1732 | 1581 | 2829 | 634 | 579 | 1037 | 304 | 182 | 614 | 111 | 67 | 225 | 31 | 14 | 48 | 11 | 5 | 18 | 10 | 5 | 16 | 4 | 2 | 6 |
| 2010 | Xinjiang | 11474 | 10476 | 18748 | 673 | 614 | 1099 | 2016 | 1207 | 4068 | 118 | 71 | 238 | 295 | 134 | 467 | 17 | 8 | 27 | 99 | 45 | 157 | 6 | 3 | 9 |
| 2011 | Xinjiang | 11251 | 10273 | 18384 | 665 | 607 | 1086 | 1976 | 1183 | 3989 | 117 | 70 | 236 | 291 | 137 | 453 | 17 | 8 | 27 | 98 | 46 | 153 | 6 | 3 | 9 |
| 2012 | Xinjiang | 10947 | 9994 | 17886 | 609 | 556 | 995 | 1923 | 1151 | 3881 | 107 | 64 | 216 | 281 | 137 | 441 | 16 | 8 | 25 | 95 | 46 | 149 | 5 | 3 | 8 |
| 2013 | Xinjiang | 10660 | 9733 | 17417 | 583 | 533 | 953 | 1873 | 1121 | 3779 | 102 | 61 | 207 | 268 | 133 | 423 | 15 | 7 | 23 | 90 | 45 | 142 | 5 | 2 | 8 |
| 2014 | Xinjiang | 10372 | 9470 | 16946 | 555 | 506 | 906 | 1822 | 1091 | 3677 | 97 | 58 | 197 | 270 | 138 | 432 | 14 | 7 | 23 | 91 | 47 | 145 | 5 | 2 | 8 |
| 2015 | Xinjiang | 9647 | 8808 | 15763 | 512 | 467 | 836 | 1695 | 1015 | 3420 | 90 | 54 | 181 | 269 | 134 | 434 | 14 | 7 | 23 | 91 | 45 | 146 | 5 | 2 | 8 |
| 2016 | Xinjiang | 9418 | 8599 | 15388 | 486 | 443 | 794 | 1654 | 991 | 3339 | 85 | 51 | 172 | 273 | 134 | 454 | 14 | 7 | 23 | 92 | 45 | 153 | 5 | 2 | 8 |
| 2017 | Xinjiang | 9075 | 8285 | 14827 | 446 | 407 | 729 | 1594 | 954 | 3217 | 78 | 47 | 158 | 289 | 95 | 366 | 14 | 5 | 18 | 97 | 32 | 123 | 5 | 2 | 6 |
| 2010 | Yunnan | 25809 | 23564 | 42169 | 856 | 781 | 1398 | 4534 | 2714 | 9150 | 150 | 90 | 303 | 792 | 373 | 1432 | 26 | 12 | 47 | 267 | 126 | 483 | 9 | 4 | 16 |
| 2011 | Yunnan | 22992 | 20992 | 37567 | 789 | 721 | 1290 | 4039 | 2418 | 8152 | 139 | 83 | 280 | 703 | 331 | 1270 | 24 | 11 | 44 | 237 | 112 | 428 | 8 | 4 | 15 |
| 2012 | Yunnan | 22140 | 20214 | 36175 | 766 | 700 | 1252 | 3889 | 2329 | 7850 | 135 | 81 | 272 | 666 | 314 | 1203 | 23 | 11 | 42 | 225 | 106 | 406 | 8 | 4 | 14 |
| 2013 | Yunnan | 19593 | 17888 | 32012 | 681 | 621 | 1112 | 3442 | 2061 | 6946 | 120 | 72 | 241 | 583 | 274 | 1053 | 20 | 10 | 37 | 196 | 93 | 355 | 7 | 3 | 12 |
| 2014 | Yunnan | 18564 | 16949 | 30331 | 646 | 589 | 1055 | 3261 | 1952 | 6582 | 113 | 68 | 229 | 556 | 262 | 1005 | 19 | 9 | 35 | 187 | 88 | 339 | 7 | 3 | 12 |
| 2015 | Yunnan | 15961 | 14573 | 26078 | 562 | 513 | 919 | 2804 | 1679 | 5659 | 99 | 59 | 199 | 477 | 225 | 862 | 17 | 8 | 30 | 161 | 76 | 291 | 6 | 3 | 10 |
| 2016 | Yunnan | 15334 | 14000 | 25054 | 537 | 490 | 877 | 2694 | 1613 | 5436 | 94 | 56 | 190 | 437 | 206 | 790 | 15 | 7 | 28 | 147 | 69 | 266 | 5 | 2 | 9 |
| 2017 | Yunnan | 13772 | 12574 | 22501 | 484 | 442 | 791 | 2419 | 1448 | 4883 | 85 | 51 | 172 | 362 | 170 | 653 | 13 | 6 | 23 | 122 | 57 | 220 | 4 | 2 | 8 |
| 2010 | Zhejiang | 11101 | 10136 | 18138 | 357 | 326 | 584 | 1950 | 1168 | 3936 | 63 | 38 | 127 | 177 | 83 | 320 | 6 | 3 | 10 | 60 | 28 | 108 | 2 | 1 | 3 |
| 2011 | Zhejiang | 9722 | 8876 | 15885 | 379 | 346 | 620 | 1708 | 1023 | 3447 | 67 | 40 | 135 | 149 | 70 | 269 | 6 | 3 | 10 | 50 | 24 | 91 | 2 | 1 | 4 |
| 2012 | Zhejiang | 9907 | 9045 | 16187 | 373 | 340 | 609 | 1740 | 1042 | 3513 | 65 | 39 | 132 | 146 | 69 | 263 | 5 | 3 | 10 | 49 | 23 | 89 | 2 | 1 | 3 |
| 2013 | Zhejiang | 10551 | 9633 | 17240 | 401 | 366 | 655 | 1853 | 1110 | 3741 | 70 | 42 | 142 | 148 | 70 | 268 | 6 | 3 | 10 | 50 | 24 | 90 | 2 | 1 | 3 |
| 2014 | Zhejiang | 9049 | 8262 | 14785 | 339 | 310 | 554 | 1590 | 952 | 3208 | 60 | 36 | 120 | 122 | 57 | 220 | 5 | 2 | 8 | 41 | 19 | 74 | 2 | 1 | 3 |
| 2015 | Zhejiang | 7595 | 6934 | 12410 | 289 | 264 | 473 | 1334 | 799 | 2693 | 51 | 30 | 103 | 96 | 45 | 173 | 4 | 2 | 7 | 32 | 15 | 58 | 1 | 1 | 2 |
| 2016 | Zhejiang | 8102 | 7398 | 13238 | 303 | 277 | 495 | 1423 | 852 | 2873 | 53 | 32 | 108 | 90 | 42 | 163 | 3 | 2 | 6 | 30 | 14 | 55 | 1 | 1 | 2 |
| 2017 | Zhejiang | 8433 | 7700 | 13779 | 314 | 287 | 513 | 1481 | 887 | 2990 | 55 | 33 | 111 | 80 | 38 | 145 | 3 | 1 | 5 | 27 | 13 | 49 | 1 | 0 | 2 |
| 2010 | Central | 111865 | 102135 | 182777 | 347 | 317 | 567 | 19650 | 11766 | 39661 | 61 | 36 | 123 | 4479 | 2109 | 8091 | 14 | 7 | 25 | 1510 | 711 | 2727 | 5 | 2 | 8 |
| 2011 | Central | 101277 | 92468 | 165477 | 320 | 292 | 523 | 17790 | 10652 | 35908 | 56 | 34 | 114 | 3943 | 1856 | 7122 | 12 | 6 | 23 | 1329 | 626 | 2401 | 4 | 2 | 8 |
| 2012 | Central | 99312 | 90674 | 162265 | 315 | 287 | 514 | 17445 | 10445 | 35211 | 55 | 33 | 112 | 3691 | 1738 | 6667 | 12 | 6 | 21 | 1244 | 586 | 2248 | 4 | 2 | 7 |
| 2013 | Central | 93631 | 85487 | 152984 | 296 | 270 | 483 | 16447 | 9848 | 33197 | 52 | 31 | 105 | 3316 | 1562 | 5991 | 10 | 5 | 19 | 1118 | 526 | 2020 | 4 | 2 | 6 |
| 2014 | Central | 89639 | 81842 | 146461 | 281 | 256 | 459 | 15746 | 9428 | 31781 | 49 | 30 | 100 | 3021 | 1423 | 5458 | 9 | 4 | 17 | 1018 | 480 | 1840 | 3 | 2 | 6 |
| 2015 | Central | 84071 | 76759 | 137364 | 266 | 243 | 435 | 14768 | 8842 | 29807 | 47 | 28 | 94 | 2723 | 1282 | 4920 | 9 | 4 | 16 | 918 | 432 | 1658 | 3 | 1 | 5 |
| 2016 | Central | 93658 | 85511 | 153027 | 296 | 270 | 484 | 16452 | 9851 | 33206 | 52 | 31 | 105 | 2745 | 1293 | 4959 | 9 | 4 | 16 | 925 | 436 | 1672 | 3 | 1 | 5 |
| 2017 | Central | 84582 | 77225 | 138198 | 266 | 243 | 435 | 14858 | 8896 | 29988 | 47 | 28 | 94 | 2192 | 1032 | 3960 | 7 | 3 | 12 | 739 | 348 | 1335 | 2 | 1 | 4 |
| 2010 | East | 85508 | 78071 | 139712 | 326 | 298 | 533 | 15020 | 8993 | 30317 | 57 | 34 | 116 | 1377 | 649 | 2488 | 5 | 2 | 9 | 464 | 219 | 839 | 2 | 1 | 3 |
| 2011 | East | 74225 | 67769 | 121277 | 318 | 291 | 520 | 13038 | 7807 | 26316 | 56 | 33 | 113 | 1156 | 544 | 2088 | 5 | 2 | 9 | 390 | 183 | 704 | 2 | 1 | 3 |
| 2012 | East | 78466 | 71641 | 128205 | 319 | 291 | 520 | 13783 | 8253 | 27820 | 56 | 34 | 113 | 1155 | 544 | 2087 | 5 | 2 | 8 | 390 | 183 | 704 | 2 | 1 | 3 |
| 2013 | East | 85225 | 77813 | 139250 | 345 | 315 | 564 | 14971 | 8964 | 30216 | 61 | 36 | 122 | 1179 | 555 | 2129 | 5 | 2 | 9 | 397 | 187 | 718 | 2 | 1 | 3 |
| 2014 | East | 72055 | 65788 | 117731 | 283 | 258 | 462 | 12657 | 7578 | 25547 | 50 | 30 | 100 | 972 | 458 | 1755 | 4 | 2 | 7 | 328 | 154 | 592 | 1 | 1 | 2 |
| 2015 | East | 64739 | 59108 | 105777 | 256 | 234 | 419 | 11372 | 6809 | 22953 | 45 | 27 | 91 | 825 | 389 | 1491 | 3 | 2 | 6 | 278 | 131 | 503 | 1 | 1 | 2 |
| 2016 | East | 78428 | 71607 | 128144 | 306 | 279 | 499 | 13777 | 8249 | 27806 | 54 | 32 | 108 | 905 | 426 | 1635 | 4 | 2 | 6 | 305 | 144 | 551 | 1 | 1 | 2 |
| 2017 | East | 77924 | 71146 | 127320 | 294 | 269 | 481 | 13688 | 8196 | 27628 | 52 | 31 | 104 | 779 | 367 | 1407 | 3 | 1 | 5 | 263 | 124 | 474 | 1 | 0 | 2 |
| 2010 | West | 128003 | 116870 | 209145 | 555 | 507 | 907 | 22485 | 13463 | 45383 | 97 | 58 | 197 | 4093 | 1989 | 7370 | 18 | 9 | 32 | 1380 | 671 | 2484 | 6 | 3 | 11 |
| 2011 | West | 121507 | 110938 | 198530 | 545 | 498 | 890 | 21344 | 12780 | 43080 | 96 | 57 | 193 | 3868 | 1764 | 6684 | 17 | 8 | 30 | 1304 | 595 | 2253 | 6 | 3 | 10 |
| 2012 | West | 113671 | 103784 | 185727 | 513 | 468 | 838 | 19967 | 11955 | 40302 | 90 | 54 | 182 | 3577 | 1649 | 6227 | 16 | 7 | 28 | 1206 | 556 | 2099 | 5 | 3 | 9 |
| 2013 | West | 105358 | 96194 | 172144 | 474 | 433 | 775 | 18507 | 11081 | 37354 | 83 | 50 | 168 | 3244 | 1513 | 5700 | 15 | 7 | 26 | 1094 | 510 | 1922 | 5 | 2 | 9 |
| 2014 | West | 100557 | 91811 | 164301 | 443 | 404 | 724 | 17664 | 10576 | 35652 | 78 | 47 | 157 | 3097 | 1471 | 5528 | 14 | 6 | 24 | 1044 | 496 | 1864 | 5 | 2 | 8 |
| 2015 | West | 88587 | 80882 | 144743 | 393 | 359 | 643 | 15561 | 9317 | 31408 | 69 | 41 | 139 | 2705 | 1282 | 4825 | 12 | 6 | 21 | 912 | 432 | 1627 | 4 | 2 | 7 |
| 2016 | West | 91188 | 83256 | 148992 | 403 | 368 | 659 | 16018 | 9591 | 32330 | 71 | 42 | 143 | 2570 | 1215 | 4596 | 11 | 5 | 20 | 866 | 410 | 1549 | 4 | 2 | 7 |
| 2017 | West | 82621 | 75435 | 134995 | 357 | 326 | 583 | 14513 | 8690 | 29293 | 63 | 38 | 127 | 2168 | 980 | 3753 | 9 | 4 | 16 | 731 | 330 | 1265 | 3 | 1 | 5 |
| 2010 | National | 325376 | 297076 | 531634 | 399 | 364 | 652 | 57156 | 34222 | 115361 | 70 | 42 | 142 | 9949 | 4747 | 17949 | 12 | 6 | 22 | 3354 | 1600 | 6050 | 4 | 2 | 7 |
| 2011 | National | 297009 | 271175 | 485284 | 385 | 351 | 628 | 52172 | 31238 | 105304 | 68 | 40 | 136 | 8967 | 4165 | 15895 | 12 | 5 | 21 | 3023 | 1404 | 5358 | 4 | 2 | 7 |
| 2012 | National | 291448 | 266098 | 476198 | 372 | 340 | 608 | 51196 | 30653 | 103332 | 65 | 39 | 132 | 8423 | 3931 | 14981 | 11 | 5 | 19 | 2839 | 1325 | 5050 | 4 | 2 | 6 |
| 2013 | National | 284214 | 259494 | 464379 | 362 | 330 | 591 | 49925 | 29893 | 100767 | 64 | 38 | 128 | 7739 | 3630 | 13820 | 10 | 5 | 18 | 2609 | 1224 | 4659 | 3 | 2 | 6 |
| 2014 | National | 262251 | 239441 | 428492 | 327 | 299 | 535 | 46067 | 27582 | 92980 | 57 | 34 | 116 | 7090 | 3351 | 12742 | 9 | 4 | 16 | 2390 | 1130 | 4295 | 3 | 1 | 5 |
| 2015 | National | 237397 | 216748 | 387884 | 299 | 273 | 489 | 41701 | 24968 | 84168 | 53 | 31 | 106 | 6254 | 2953 | 11236 | 8 | 4 | 14 | 2108 | 995 | 3788 | 3 | 1 | 5 |
| 2016 | National | 263273 | 240374 | 430163 | 330 | 301 | 539 | 46246 | 27690 | 93343 | 58 | 35 | 117 | 6221 | 2934 | 11191 | 8 | 4 | 14 | 2097 | 989 | 3772 | 3 | 1 | 5 |
| 2017 | National | 245127 | 223806 | 400514 | 301 | 275 | 492 | 43059 | 25781 | 86909 | 53 | 32 | 107 | 5139 | 2379 | 9120 | 6 | 3 | 11 | 1732 | 802 | 3074 | 2 | 1 | 4 |
| 1. Clinical pneumonia cases include severe pneumonia cases. | | | | | | | | | | | | | | | | | | | | | | | | | |
| 2. All Hib meningitis cases are severe. | | | | | | | | | | | | | | | | | | | | | | | | | |
| 3. All Hib NPNM cases are severe. | | | | | | | | | | | | | | | | | | | | | | | | | |

**Webappendix 12: Sensitivity results of pneumococcal pneumonia mortality by different vaccine efficacy in China by region, province, and year**

| Year | Province | Base case | | | | | | VT AOM vaccine efficacy adjustment | | | | | | VT pneumococcal pneumonia efficacy adjustment (from the elderly trial in Netherlands) | | | | | |
| --- | --- | --- | --- | --- | --- | --- | --- | --- | --- | --- | --- | --- | --- | --- | --- | --- | --- | --- | --- |
|  |  | Pneumococcal pneumonia deaths | | | | | | Pneumococcal pneumonia deaths | | | | | | Pneumococcal pneumonia deaths | | | | | |
|  |  | Number | UR | | Rate per 100 000 | UR | | Number | UR | | Rate per 100 000 | UR | | Number | UR | | Rate per 100 000 | UR | |
| 2010 | Anhui | 525 | 373 | 548 | 14 | 10 | 15 | 780 | 523 | 821 | 21 | 14 | 22 | 1009 | 614 | 1080 | 27 | 16 | 29 |
| 2011 | Anhui | 464 | 329 | 483 | 13 | 9 | 13 | 688 | 462 | 724 | 19 | 13 | 20 | 890 | 542 | 954 | 24 | 15 | 26 |
| 2012 | Anhui | 407 | 289 | 424 | 11 | 8 | 12 | 605 | 406 | 636 | 17 | 11 | 17 | 782 | 476 | 837 | 21 | 13 | 23 |
| 2013 | Anhui | 356 | 253 | 371 | 10 | 7 | 10 | 529 | 355 | 557 | 15 | 10 | 15 | 684 | 416 | 733 | 19 | 12 | 20 |
| 2014 | Anhui | 320 | 227 | 333 | 9 | 6 | 9 | 475 | 319 | 499 | 13 | 9 | 14 | 614 | 374 | 658 | 17 | 10 | 18 |
| 2015 | Anhui | 283 | 201 | 295 | 8 | 6 | 8 | 420 | 282 | 442 | 12 | 8 | 12 | 543 | 330 | 581 | 15 | 9 | 16 |
| 2016 | Anhui | 242 | 172 | 253 | 6 | 5 | 7 | 360 | 241 | 379 | 10 | 6 | 10 | 465 | 283 | 498 | 12 | 8 | 13 |
| 2017 | Anhui | 208 | 147 | 217 | 5 | 4 | 6 | 308 | 207 | 324 | 8 | 5 | 8 | 399 | 243 | 427 | 10 | 6 | 11 |
| 2010 | Beijing | 34 | 24 | 35 | 4 | 3 | 4 | 51 | 34 | 53 | 6 | 4 | 6 | 65 | 40 | 70 | 8 | 5 | 8 |
| 2011 | Beijing | 34 | 24 | 36 | 6 | 4 | 6 | 51 | 34 | 54 | 9 | 6 | 9 | 66 | 40 | 71 | 11 | 7 | 12 |
| 2012 | Beijing | 34 | 24 | 36 | 4 | 3 | 4 | 51 | 34 | 53 | 6 | 4 | 7 | 66 | 40 | 70 | 8 | 5 | 9 |
| 2013 | Beijing | 36 | 25 | 37 | 4 | 3 | 5 | 53 | 36 | 56 | 6 | 4 | 7 | 69 | 42 | 73 | 8 | 5 | 9 |
| 2014 | Beijing | 38 | 27 | 39 | 4 | 3 | 5 | 56 | 38 | 59 | 7 | 4 | 7 | 73 | 44 | 78 | 8 | 5 | 9 |
| 2015 | Beijing | 39 | 28 | 40 | 5 | 3 | 5 | 58 | 39 | 61 | 7 | 5 | 7 | 75 | 45 | 80 | 9 | 5 | 9 |
| 2016 | Beijing | 39 | 27 | 40 | 4 | 3 | 5 | 57 | 38 | 60 | 7 | 4 | 7 | 74 | 45 | 79 | 8 | 5 | 9 |
| 2017 | Beijing | 36 | 25 | 37 | 4 | 3 | 4 | 53 | 36 | 56 | 6 | 4 | 6 | 69 | 42 | 73 | 7 | 4 | 8 |
| 2010 | Chongqing | 210 | 149 | 219 | 14 | 10 | 15 | 312 | 209 | 328 | 21 | 14 | 22 | 403 | 245 | 432 | 27 | 17 | 29 |
| 2011 | Chongqing | 188 | 133 | 196 | 13 | 9 | 13 | 279 | 187 | 294 | 19 | 12 | 20 | 361 | 220 | 387 | 24 | 15 | 26 |
| 2012 | Chongqing | 167 | 118 | 174 | 11 | 7 | 11 | 247 | 166 | 260 | 16 | 11 | 16 | 320 | 195 | 342 | 20 | 12 | 22 |
| 2013 | Chongqing | 148 | 105 | 154 | 10 | 7 | 10 | 219 | 147 | 230 | 14 | 10 | 15 | 283 | 172 | 303 | 18 | 11 | 20 |
| 2014 | Chongqing | 133 | 94 | 138 | 8 | 6 | 9 | 197 | 132 | 207 | 12 | 8 | 13 | 255 | 155 | 273 | 16 | 10 | 17 |
| 2015 | Chongqing | 117 | 83 | 122 | 7 | 5 | 8 | 174 | 117 | 183 | 11 | 7 | 12 | 225 | 137 | 241 | 14 | 9 | 15 |
| 2016 | Chongqing | 99 | 70 | 104 | 6 | 5 | 7 | 148 | 99 | 155 | 9 | 6 | 10 | 191 | 116 | 204 | 12 | 7 | 13 |
| 2017 | Chongqing | 84 | 60 | 88 | 5 | 4 | 5 | 125 | 84 | 132 | 8 | 5 | 8 | 162 | 98 | 173 | 10 | 6 | 11 |
| 2010 | Fujian | 263 | 186 | 274 | 11 | 8 | 11 | 390 | 262 | 410 | 16 | 11 | 17 | 504 | 307 | 540 | 20 | 12 | 22 |
| 2011 | Fujian | 237 | 168 | 247 | 11 | 8 | 12 | 352 | 236 | 370 | 17 | 11 | 18 | 455 | 277 | 487 | 22 | 13 | 23 |
| 2012 | Fujian | 212 | 150 | 221 | 9 | 7 | 10 | 315 | 211 | 331 | 14 | 9 | 14 | 407 | 248 | 436 | 18 | 11 | 19 |
| 2013 | Fujian | 189 | 134 | 197 | 8 | 6 | 8 | 281 | 189 | 296 | 12 | 8 | 13 | 363 | 221 | 389 | 16 | 9 | 17 |
| 2014 | Fujian | 173 | 123 | 180 | 7 | 5 | 7 | 257 | 172 | 270 | 11 | 7 | 11 | 332 | 202 | 356 | 14 | 8 | 15 |
| 2015 | Fujian | 154 | 109 | 161 | 6 | 5 | 7 | 229 | 154 | 241 | 10 | 6 | 10 | 296 | 180 | 317 | 12 | 8 | 13 |
| 2016 | Fujian | 133 | 95 | 139 | 5 | 4 | 6 | 198 | 133 | 208 | 8 | 5 | 9 | 256 | 156 | 274 | 10 | 6 | 11 |
| 2017 | Fujian | 115 | 82 | 120 | 5 | 3 | 5 | 171 | 115 | 180 | 7 | 5 | 7 | 222 | 135 | 237 | 9 | 5 | 10 |
| 2010 | Gansu | 571 | 405 | 596 | 36 | 25 | 37 | 848 | 569 | 892 | 53 | 36 | 56 | 1097 | 667 | 1175 | 69 | 42 | 74 |
| 2011 | Gansu | 498 | 353 | 519 | 33 | 24 | 35 | 740 | 496 | 778 | 49 | 33 | 52 | 956 | 582 | 1024 | 64 | 39 | 68 |
| 2012 | Gansu | 433 | 307 | 452 | 31 | 22 | 32 | 643 | 431 | 677 | 45 | 30 | 48 | 832 | 506 | 891 | 59 | 36 | 63 |
| 2013 | Gansu | 374 | 265 | 389 | 27 | 19 | 28 | 555 | 372 | 583 | 39 | 26 | 41 | 717 | 436 | 768 | 51 | 31 | 55 |
| 2014 | Gansu | 331 | 235 | 345 | 23 | 16 | 23 | 492 | 330 | 517 | 33 | 22 | 35 | 636 | 387 | 681 | 43 | 26 | 46 |
| 2015 | Gansu | 288 | 204 | 300 | 20 | 14 | 21 | 427 | 286 | 449 | 29 | 20 | 31 | 552 | 336 | 591 | 38 | 23 | 41 |
| 2016 | Gansu | 245 | 174 | 256 | 17 | 12 | 17 | 364 | 244 | 383 | 25 | 17 | 26 | 471 | 287 | 505 | 32 | 20 | 34 |
| 2017 | Gansu | 211 | 150 | 220 | 14 | 10 | 15 | 313 | 210 | 329 | 21 | 14 | 22 | 405 | 246 | 434 | 28 | 17 | 29 |
| 2010 | Guangdong | 772 | 547 | 805 | 14 | 10 | 14 | 1146 | 769 | 1205 | 20 | 13 | 21 | 1482 | 902 | 1587 | 26 | 16 | 28 |
| 2011 | Guangdong | 702 | 498 | 731 | 13 | 9 | 14 | 1042 | 699 | 1096 | 19 | 13 | 20 | 1347 | 819 | 1443 | 25 | 15 | 27 |
| 2012 | Guangdong | 644 | 457 | 671 | 10 | 7 | 10 | 956 | 641 | 1006 | 15 | 10 | 15 | 1237 | 752 | 1324 | 19 | 12 | 20 |
| 2013 | Guangdong | 602 | 427 | 627 | 9 | 7 | 10 | 894 | 600 | 940 | 14 | 9 | 14 | 1156 | 703 | 1238 | 18 | 11 | 19 |
| 2014 | Guangdong | 580 | 411 | 604 | 9 | 6 | 9 | 861 | 578 | 906 | 13 | 8 | 13 | 1113 | 677 | 1192 | 16 | 10 | 18 |
| 2015 | Guangdong | 548 | 389 | 571 | 8 | 6 | 8 | 814 | 546 | 856 | 12 | 8 | 13 | 1053 | 640 | 1127 | 16 | 9 | 17 |
| 2016 | Guangdong | 500 | 354 | 521 | 7 | 5 | 8 | 742 | 498 | 780 | 11 | 7 | 11 | 959 | 584 | 1027 | 14 | 9 | 15 |
| 2017 | Guangdong | 451 | 320 | 470 | 6 | 4 | 6 | 670 | 449 | 705 | 9 | 6 | 9 | 866 | 527 | 928 | 12 | 7 | 12 |
| 2010 | Guangxi | 397 | 282 | 414 | 11 | 8 | 12 | 590 | 396 | 621 | 17 | 11 | 18 | 763 | 464 | 817 | 22 | 13 | 23 |
| 2011 | Guangxi | 372 | 264 | 388 | 10 | 7 | 11 | 553 | 371 | 581 | 15 | 10 | 16 | 714 | 435 | 765 | 20 | 12 | 21 |
| 2012 | Guangxi | 347 | 246 | 361 | 9 | 7 | 10 | 515 | 345 | 542 | 14 | 9 | 15 | 666 | 405 | 713 | 18 | 11 | 19 |
| 2013 | Guangxi | 321 | 228 | 335 | 8 | 6 | 9 | 477 | 320 | 502 | 13 | 8 | 13 | 617 | 375 | 661 | 16 | 10 | 17 |
| 2014 | Guangxi | 308 | 218 | 321 | 8 | 6 | 8 | 457 | 307 | 481 | 12 | 8 | 12 | 591 | 360 | 633 | 15 | 9 | 16 |
| 2015 | Guangxi | 289 | 205 | 301 | 8 | 5 | 8 | 429 | 288 | 451 | 11 | 7 | 12 | 555 | 338 | 594 | 14 | 9 | 15 |
| 2016 | Guangxi | 261 | 185 | 273 | 7 | 5 | 7 | 388 | 260 | 408 | 10 | 7 | 10 | 502 | 305 | 538 | 13 | 8 | 14 |
| 2017 | Guangxi | 234 | 166 | 243 | 6 | 4 | 6 | 347 | 233 | 365 | 9 | 6 | 9 | 448 | 273 | 480 | 11 | 7 | 12 |
| 2010 | Guizhou | 526 | 373 | 549 | 21 | 15 | 22 | 782 | 524 | 822 | 32 | 21 | 33 | 1011 | 615 | 1082 | 41 | 25 | 44 |
| 2011 | Guizhou | 453 | 321 | 472 | 18 | 13 | 18 | 673 | 451 | 708 | 26 | 18 | 28 | 870 | 529 | 932 | 34 | 21 | 36 |
| 2012 | Guizhou | 390 | 277 | 406 | 15 | 11 | 16 | 579 | 388 | 609 | 23 | 15 | 24 | 749 | 456 | 802 | 30 | 18 | 32 |
| 2013 | Guizhou | 337 | 239 | 351 | 13 | 9 | 14 | 500 | 335 | 526 | 20 | 13 | 21 | 647 | 393 | 693 | 26 | 16 | 27 |
| 2014 | Guizhou | 297 | 211 | 310 | 11 | 8 | 12 | 441 | 296 | 464 | 17 | 11 | 18 | 571 | 347 | 611 | 22 | 13 | 24 |
| 2015 | Guizhou | 256 | 181 | 267 | 10 | 7 | 10 | 380 | 255 | 400 | 15 | 10 | 16 | 491 | 299 | 526 | 19 | 12 | 21 |
| 2016 | Guizhou | 216 | 153 | 225 | 9 | 6 | 9 | 321 | 215 | 338 | 13 | 9 | 13 | 415 | 253 | 445 | 17 | 10 | 18 |
| 2017 | Guizhou | 184 | 131 | 192 | 7 | 5 | 7 | 273 | 183 | 287 | 10 | 6 | 10 | 353 | 215 | 378 | 13 | 8 | 13 |
| 2010 | Hainan | 158 | 112 | 164 | 25 | 17 | 26 | 234 | 157 | 246 | 37 | 25 | 38 | 303 | 184 | 324 | 47 | 29 | 51 |
| 2011 | Hainan | 149 | 106 | 156 | 24 | 17 | 25 | 222 | 149 | 233 | 36 | 24 | 38 | 286 | 174 | 307 | 46 | 28 | 50 |
| 2012 | Hainan | 141 | 100 | 147 | 22 | 16 | 23 | 209 | 140 | 220 | 32 | 22 | 34 | 271 | 165 | 290 | 42 | 26 | 45 |
| 2013 | Hainan | 134 | 95 | 140 | 21 | 15 | 22 | 199 | 133 | 209 | 31 | 21 | 32 | 257 | 156 | 275 | 40 | 24 | 43 |
| 2014 | Hainan | 131 | 93 | 136 | 20 | 14 | 21 | 194 | 130 | 204 | 30 | 20 | 31 | 251 | 153 | 269 | 39 | 23 | 41 |
| 2015 | Hainan | 126 | 89 | 131 | 19 | 14 | 20 | 186 | 125 | 196 | 29 | 19 | 30 | 241 | 147 | 258 | 37 | 23 | 40 |
| 2016 | Hainan | 118 | 83 | 123 | 18 | 13 | 19 | 175 | 117 | 184 | 27 | 18 | 28 | 226 | 137 | 242 | 35 | 21 | 37 |
| 2017 | Hainan | 107 | 76 | 111 | 16 | 11 | 17 | 158 | 106 | 166 | 24 | 16 | 25 | 205 | 124 | 219 | 31 | 19 | 33 |
| 2010 | Hebei | 572 | 406 | 596 | 11 | 8 | 12 | 849 | 570 | 893 | 17 | 11 | 18 | 1098 | 668 | 1177 | 22 | 13 | 23 |
| 2011 | Hebei | 546 | 387 | 569 | 11 | 8 | 12 | 811 | 544 | 853 | 17 | 11 | 18 | 1049 | 638 | 1123 | 22 | 13 | 23 |
| 2012 | Hebei | 519 | 368 | 541 | 10 | 7 | 11 | 771 | 517 | 811 | 15 | 10 | 16 | 997 | 607 | 1068 | 20 | 12 | 21 |
| 2013 | Hebei | 491 | 348 | 512 | 10 | 7 | 10 | 729 | 489 | 766 | 14 | 10 | 15 | 942 | 573 | 1009 | 18 | 11 | 20 |
| 2014 | Hebei | 479 | 340 | 499 | 9 | 7 | 10 | 711 | 477 | 748 | 14 | 9 | 14 | 919 | 559 | 985 | 18 | 11 | 19 |
| 2015 | Hebei | 458 | 325 | 477 | 9 | 6 | 9 | 680 | 456 | 715 | 13 | 9 | 14 | 879 | 535 | 942 | 17 | 11 | 19 |
| 2016 | Hebei | 426 | 302 | 444 | 8 | 6 | 9 | 632 | 424 | 665 | 13 | 8 | 13 | 817 | 497 | 875 | 16 | 10 | 17 |
| 2017 | Hebei | 386 | 274 | 402 | 7 | 5 | 8 | 573 | 384 | 602 | 11 | 7 | 12 | 741 | 451 | 793 | 14 | 9 | 15 |
| 2010 | Heilongjiang | 131 | 93 | 136 | 6 | 5 | 7 | 194 | 130 | 204 | 10 | 6 | 10 | 251 | 153 | 269 | 12 | 7 | 13 |
| 2011 | Heilongjiang | 123 | 87 | 128 | 8 | 5 | 8 | 182 | 122 | 192 | 11 | 8 | 12 | 236 | 143 | 253 | 15 | 9 | 16 |
| 2012 | Heilongjiang | 113 | 80 | 117 | 8 | 6 | 9 | 167 | 112 | 176 | 12 | 8 | 13 | 216 | 132 | 232 | 16 | 10 | 17 |
| 2013 | Heilongjiang | 103 | 73 | 107 | 8 | 6 | 8 | 153 | 102 | 160 | 12 | 8 | 12 | 197 | 120 | 211 | 15 | 9 | 16 |
| 2014 | Heilongjiang | 95 | 67 | 99 | 7 | 5 | 7 | 141 | 95 | 148 | 11 | 7 | 11 | 182 | 111 | 195 | 14 | 8 | 15 |
| 2015 | Heilongjiang | 85 | 60 | 89 | 7 | 5 | 7 | 127 | 85 | 133 | 10 | 7 | 10 | 164 | 100 | 175 | 13 | 8 | 14 |
| 2016 | Heilongjiang | 75 | 53 | 78 | 6 | 4 | 6 | 112 | 75 | 117 | 9 | 6 | 9 | 144 | 88 | 155 | 11 | 7 | 12 |
| 2017 | Heilongjiang | 67 | 47 | 69 | 6 | 4 | 6 | 99 | 66 | 104 | 9 | 6 | 9 | 128 | 78 | 137 | 12 | 7 | 12 |
| 2010 | Henan | 613 | 435 | 639 | 9 | 7 | 10 | 910 | 610 | 957 | 14 | 9 | 15 | 1177 | 716 | 1260 | 18 | 11 | 19 |
| 2011 | Henan | 567 | 402 | 591 | 8 | 6 | 8 | 842 | 565 | 886 | 12 | 8 | 12 | 1089 | 663 | 1167 | 15 | 9 | 16 |
| 2012 | Henan | 522 | 370 | 544 | 7 | 5 | 7 | 775 | 520 | 815 | 10 | 7 | 11 | 1002 | 610 | 1073 | 14 | 8 | 14 |
| 2013 | Henan | 477 | 338 | 497 | 6 | 5 | 7 | 708 | 475 | 745 | 10 | 6 | 10 | 916 | 557 | 981 | 12 | 7 | 13 |
| 2014 | Henan | 447 | 317 | 466 | 6 | 4 | 6 | 663 | 445 | 698 | 9 | 6 | 9 | 858 | 522 | 919 | 12 | 7 | 12 |
| 2015 | Henan | 409 | 290 | 427 | 6 | 4 | 6 | 608 | 408 | 639 | 8 | 6 | 9 | 786 | 478 | 842 | 11 | 7 | 11 |
| 2016 | Henan | 362 | 257 | 377 | 5 | 3 | 5 | 537 | 360 | 565 | 7 | 5 | 8 | 694 | 422 | 744 | 9 | 6 | 10 |
| 2017 | Henan | 318 | 226 | 332 | 4 | 3 | 4 | 473 | 317 | 497 | 6 | 4 | 7 | 611 | 372 | 655 | 8 | 5 | 9 |
| 2010 | Hubei | 267 | 189 | 278 | 8 | 5 | 8 | 396 | 266 | 416 | 11 | 7 | 12 | 512 | 311 | 548 | 14 | 9 | 15 |
| 2011 | Hubei | 248 | 176 | 258 | 8 | 6 | 8 | 367 | 247 | 387 | 12 | 8 | 13 | 475 | 289 | 509 | 15 | 9 | 17 |
| 2012 | Hubei | 225 | 160 | 235 | 8 | 5 | 8 | 335 | 225 | 352 | 11 | 7 | 12 | 433 | 263 | 464 | 14 | 9 | 15 |
| 2013 | Hubei | 203 | 144 | 211 | 7 | 5 | 7 | 301 | 202 | 317 | 10 | 7 | 10 | 389 | 237 | 417 | 13 | 8 | 14 |
| 2014 | Hubei | 186 | 132 | 194 | 6 | 4 | 6 | 277 | 186 | 291 | 9 | 6 | 9 | 358 | 218 | 383 | 11 | 7 | 12 |
| 2015 | Hubei | 167 | 118 | 174 | 5 | 4 | 6 | 247 | 166 | 260 | 8 | 5 | 8 | 320 | 195 | 343 | 10 | 6 | 11 |
| 2016 | Hubei | 145 | 103 | 151 | 5 | 3 | 5 | 215 | 144 | 226 | 7 | 5 | 7 | 278 | 169 | 297 | 9 | 6 | 10 |
| 2017 | Hubei | 125 | 89 | 131 | 4 | 3 | 4 | 186 | 125 | 196 | 6 | 4 | 6 | 241 | 146 | 258 | 7 | 5 | 8 |
| 2010 | Hunan | 272 | 193 | 283 | 7 | 5 | 7 | 403 | 271 | 424 | 10 | 7 | 10 | 522 | 317 | 559 | 13 | 8 | 14 |
| 2011 | Hunan | 250 | 178 | 261 | 6 | 4 | 6 | 372 | 249 | 391 | 9 | 6 | 9 | 481 | 293 | 515 | 11 | 7 | 12 |
| 2012 | Hunan | 227 | 161 | 237 | 5 | 4 | 6 | 337 | 226 | 354 | 8 | 5 | 9 | 436 | 265 | 467 | 11 | 6 | 11 |
| 2013 | Hunan | 203 | 144 | 212 | 5 | 3 | 5 | 301 | 202 | 317 | 7 | 5 | 8 | 390 | 237 | 418 | 9 | 6 | 10 |
| 2014 | Hunan | 186 | 132 | 194 | 4 | 3 | 5 | 277 | 186 | 291 | 6 | 4 | 7 | 358 | 218 | 383 | 8 | 5 | 9 |
| 2015 | Hunan | 165 | 117 | 172 | 4 | 3 | 4 | 246 | 165 | 258 | 6 | 4 | 6 | 318 | 193 | 340 | 7 | 4 | 8 |
| 2016 | Hunan | 142 | 101 | 148 | 3 | 2 | 3 | 211 | 142 | 222 | 5 | 3 | 5 | 273 | 166 | 292 | 6 | 4 | 7 |
| 2017 | Hunan | 123 | 87 | 128 | 3 | 2 | 3 | 182 | 122 | 192 | 4 | 3 | 4 | 236 | 143 | 252 | 5 | 3 | 6 |
| 2010 | Inner Mongolia | 187 | 132 | 195 | 13 | 9 | 14 | 277 | 186 | 292 | 19 | 13 | 20 | 359 | 218 | 384 | 25 | 15 | 27 |
| 2011 | Inner Mongolia | 174 | 124 | 182 | 14 | 10 | 15 | 259 | 174 | 272 | 21 | 14 | 23 | 335 | 204 | 359 | 28 | 17 | 30 |
| 2012 | Inner Mongolia | 162 | 115 | 169 | 14 | 10 | 15 | 241 | 161 | 253 | 22 | 14 | 23 | 311 | 189 | 333 | 28 | 17 | 30 |
| 2013 | Inner Mongolia | 150 | 107 | 157 | 13 | 10 | 14 | 223 | 150 | 235 | 20 | 13 | 21 | 289 | 176 | 309 | 26 | 16 | 28 |
| 2014 | Inner Mongolia | 144 | 102 | 150 | 13 | 9 | 13 | 213 | 143 | 224 | 19 | 13 | 20 | 276 | 168 | 295 | 24 | 15 | 26 |
| 2015 | Inner Mongolia | 135 | 96 | 140 | 12 | 9 | 13 | 200 | 134 | 210 | 18 | 12 | 19 | 259 | 157 | 277 | 23 | 14 | 25 |
| 2016 | Inner Mongolia | 123 | 87 | 128 | 11 | 8 | 11 | 182 | 122 | 191 | 16 | 11 | 17 | 235 | 143 | 252 | 21 | 13 | 23 |
| 2017 | Inner Mongolia | 110 | 78 | 115 | 10 | 7 | 11 | 163 | 110 | 172 | 16 | 10 | 16 | 211 | 129 | 226 | 20 | 12 | 22 |
| 2010 | Jiangsu | 136 | 97 | 142 | 3 | 2 | 3 | 202 | 136 | 213 | 5 | 3 | 5 | 262 | 159 | 280 | 6 | 4 | 6 |
| 2011 | Jiangsu | 124 | 88 | 129 | 3 | 2 | 3 | 184 | 124 | 194 | 5 | 3 | 5 | 238 | 145 | 255 | 6 | 4 | 7 |
| 2012 | Jiangsu | 112 | 80 | 117 | 3 | 2 | 3 | 167 | 112 | 176 | 4 | 3 | 4 | 216 | 131 | 231 | 5 | 3 | 6 |
| 2013 | Jiangsu | 102 | 73 | 107 | 3 | 2 | 3 | 152 | 102 | 160 | 4 | 3 | 4 | 197 | 120 | 211 | 5 | 3 | 5 |
| 2014 | Jiangsu | 95 | 67 | 99 | 2 | 2 | 3 | 141 | 95 | 148 | 4 | 2 | 4 | 182 | 111 | 195 | 5 | 3 | 5 |
| 2015 | Jiangsu | 86 | 61 | 89 | 2 | 2 | 2 | 127 | 85 | 134 | 3 | 2 | 3 | 165 | 100 | 176 | 4 | 3 | 5 |
| 2016 | Jiangsu | 74 | 53 | 77 | 2 | 1 | 2 | 110 | 74 | 116 | 3 | 2 | 3 | 143 | 87 | 153 | 4 | 2 | 4 |
| 2017 | Jiangsu | 64 | 46 | 67 | 2 | 1 | 2 | 96 | 64 | 101 | 3 | 2 | 3 | 124 | 75 | 133 | 3 | 2 | 3 |
| 2010 | Jiangxi | 866 | 614 | 903 | 29 | 20 | 30 | 1286 | 862 | 1352 | 43 | 29 | 45 | 1663 | 1012 | 1781 | 55 | 34 | 59 |
| 2011 | Jiangxi | 773 | 548 | 806 | 24 | 17 | 25 | 1148 | 770 | 1207 | 35 | 24 | 37 | 1484 | 903 | 1590 | 46 | 28 | 49 |
| 2012 | Jiangxi | 690 | 489 | 719 | 20 | 14 | 21 | 1025 | 687 | 1078 | 30 | 20 | 31 | 1325 | 806 | 1419 | 38 | 23 | 41 |
| 2013 | Jiangxi | 617 | 437 | 643 | 18 | 13 | 19 | 916 | 614 | 963 | 27 | 18 | 28 | 1184 | 720 | 1268 | 35 | 21 | 37 |
| 2014 | Jiangxi | 567 | 402 | 591 | 17 | 12 | 18 | 841 | 564 | 885 | 25 | 17 | 26 | 1088 | 662 | 1165 | 32 | 20 | 35 |
| 2015 | Jiangxi | 512 | 363 | 533 | 15 | 11 | 16 | 760 | 509 | 799 | 23 | 15 | 24 | 982 | 597 | 1052 | 30 | 18 | 32 |
| 2016 | Jiangxi | 456 | 324 | 476 | 14 | 10 | 14 | 677 | 454 | 713 | 21 | 14 | 22 | 876 | 533 | 938 | 27 | 16 | 28 |
| 2017 | Jiangxi | 405 | 287 | 422 | 13 | 9 | 14 | 601 | 403 | 633 | 19 | 13 | 20 | 778 | 473 | 833 | 25 | 15 | 27 |
| 2010 | Jilin | 148 | 105 | 154 | 10 | 7 | 11 | 219 | 147 | 231 | 15 | 10 | 16 | 284 | 173 | 304 | 19 | 12 | 21 |
| 2011 | Jilin | 135 | 96 | 141 | 11 | 8 | 12 | 201 | 135 | 211 | 17 | 11 | 18 | 260 | 158 | 278 | 22 | 13 | 23 |
| 2012 | Jilin | 123 | 87 | 128 | 12 | 8 | 12 | 183 | 123 | 192 | 17 | 12 | 18 | 237 | 144 | 253 | 22 | 14 | 24 |
| 2013 | Jilin | 113 | 80 | 118 | 11 | 8 | 11 | 168 | 113 | 177 | 16 | 11 | 17 | 217 | 132 | 232 | 21 | 13 | 22 |
| 2014 | Jilin | 107 | 76 | 112 | 10 | 7 | 11 | 159 | 107 | 167 | 15 | 10 | 16 | 206 | 125 | 220 | 20 | 12 | 21 |
| 2015 | Jilin | 98 | 70 | 103 | 10 | 7 | 10 | 146 | 98 | 154 | 14 | 10 | 15 | 189 | 115 | 202 | 19 | 11 | 20 |
| 2016 | Jilin | 87 | 62 | 90 | 9 | 6 | 9 | 129 | 86 | 136 | 13 | 9 | 13 | 167 | 101 | 179 | 17 | 10 | 18 |
| 2017 | Jilin | 77 | 54 | 80 | 8 | 6 | 8 | 114 | 76 | 120 | 12 | 8 | 13 | 147 | 90 | 158 | 15 | 9 | 17 |
| 2010 | Liaoning | 60 | 43 | 63 | 3 | 2 | 3 | 89 | 60 | 94 | 4 | 3 | 4 | 115 | 70 | 124 | 5 | 3 | 6 |
| 2011 | Liaoning | 55 | 39 | 57 | 3 | 2 | 3 | 82 | 55 | 86 | 5 | 3 | 5 | 106 | 64 | 113 | 6 | 4 | 6 |
| 2012 | Liaoning | 50 | 36 | 52 | 3 | 2 | 3 | 75 | 50 | 79 | 5 | 3 | 5 | 97 | 59 | 103 | 6 | 4 | 7 |
| 2013 | Liaoning | 47 | 33 | 49 | 3 | 2 | 3 | 69 | 46 | 73 | 5 | 3 | 5 | 89 | 54 | 96 | 6 | 4 | 6 |
| 2014 | Liaoning | 44 | 31 | 46 | 3 | 2 | 3 | 66 | 44 | 69 | 4 | 3 | 4 | 85 | 52 | 91 | 5 | 3 | 6 |
| 2015 | Liaoning | 41 | 29 | 43 | 3 | 2 | 3 | 61 | 41 | 64 | 4 | 3 | 4 | 79 | 48 | 84 | 5 | 3 | 6 |
| 2016 | Liaoning | 36 | 26 | 38 | 2 | 2 | 3 | 54 | 36 | 57 | 4 | 2 | 4 | 70 | 43 | 75 | 5 | 3 | 5 |
| 2017 | Liaoning | 32 | 23 | 34 | 2 | 2 | 2 | 48 | 32 | 51 | 3 | 2 | 4 | 62 | 38 | 67 | 4 | 3 | 5 |
| 2010 | Ningxia | 130 | 92 | 135 | 29 | 21 | 31 | 193 | 129 | 203 | 44 | 29 | 46 | 250 | 152 | 267 | 56 | 34 | 60 |
| 2011 | Ningxia | 116 | 82 | 121 | 27 | 19 | 28 | 173 | 116 | 181 | 39 | 26 | 41 | 223 | 136 | 239 | 51 | 31 | 55 |
| 2012 | Ningxia | 104 | 74 | 108 | 24 | 17 | 25 | 154 | 103 | 162 | 35 | 24 | 37 | 199 | 121 | 213 | 46 | 28 | 49 |
| 2013 | Ningxia | 93 | 66 | 97 | 21 | 15 | 22 | 138 | 92 | 145 | 32 | 21 | 33 | 178 | 108 | 191 | 41 | 25 | 44 |
| 2014 | Ningxia | 86 | 61 | 90 | 18 | 13 | 19 | 128 | 86 | 134 | 27 | 18 | 29 | 165 | 101 | 177 | 35 | 22 | 38 |
| 2015 | Ningxia | 79 | 56 | 82 | 17 | 12 | 18 | 117 | 78 | 123 | 26 | 17 | 27 | 151 | 92 | 162 | 33 | 20 | 36 |
| 2016 | Ningxia | 71 | 50 | 74 | 16 | 11 | 16 | 105 | 71 | 111 | 23 | 16 | 25 | 136 | 83 | 146 | 30 | 18 | 32 |
| 2017 | Ningxia | 63 | 45 | 66 | 14 | 10 | 15 | 94 | 63 | 98 | 21 | 14 | 22 | 121 | 74 | 130 | 27 | 16 | 29 |
| 2010 | Qinghai | 224 | 159 | 233 | 61 | 44 | 64 | 333 | 223 | 350 | 91 | 61 | 96 | 430 | 262 | 461 | 118 | 72 | 126 |
| 2011 | Qinghai | 203 | 144 | 211 | 56 | 40 | 58 | 301 | 202 | 317 | 83 | 56 | 87 | 390 | 237 | 417 | 107 | 65 | 115 |
| 2012 | Qinghai | 184 | 130 | 191 | 51 | 36 | 53 | 272 | 183 | 287 | 76 | 51 | 80 | 352 | 214 | 377 | 98 | 60 | 105 |
| 2013 | Qinghai | 167 | 118 | 174 | 48 | 34 | 50 | 248 | 166 | 261 | 71 | 47 | 74 | 320 | 195 | 343 | 91 | 56 | 98 |
| 2014 | Qinghai | 155 | 110 | 162 | 42 | 30 | 43 | 230 | 154 | 242 | 62 | 42 | 65 | 298 | 181 | 319 | 80 | 49 | 86 |
| 2015 | Qinghai | 141 | 100 | 147 | 38 | 27 | 40 | 210 | 141 | 221 | 57 | 38 | 60 | 271 | 165 | 291 | 73 | 45 | 78 |
| 2016 | Qinghai | 123 | 87 | 128 | 33 | 23 | 34 | 182 | 122 | 192 | 49 | 33 | 51 | 236 | 143 | 252 | 63 | 38 | 67 |
| 2017 | Qinghai | 107 | 76 | 112 | 28 | 20 | 30 | 159 | 107 | 167 | 42 | 28 | 44 | 205 | 125 | 220 | 55 | 33 | 58 |
| 2010 | Shaanxi | 480 | 340 | 500 | 21 | 15 | 22 | 713 | 478 | 750 | 31 | 21 | 33 | 922 | 561 | 987 | 40 | 25 | 43 |
| 2011 | Shaanxi | 433 | 307 | 451 | 21 | 15 | 22 | 642 | 431 | 675 | 31 | 21 | 33 | 830 | 505 | 889 | 40 | 25 | 43 |
| 2012 | Shaanxi | 391 | 278 | 408 | 20 | 14 | 21 | 581 | 390 | 611 | 30 | 20 | 31 | 751 | 457 | 805 | 39 | 24 | 41 |
| 2013 | Shaanxi | 354 | 251 | 369 | 18 | 13 | 19 | 526 | 353 | 553 | 27 | 18 | 28 | 680 | 414 | 728 | 34 | 21 | 37 |
| 2014 | Shaanxi | 331 | 235 | 345 | 16 | 11 | 17 | 491 | 329 | 516 | 24 | 16 | 25 | 635 | 386 | 680 | 31 | 19 | 33 |
| 2015 | Shaanxi | 306 | 217 | 318 | 15 | 11 | 16 | 454 | 304 | 477 | 22 | 15 | 23 | 587 | 357 | 628 | 29 | 17 | 31 |
| 2016 | Shaanxi | 275 | 195 | 287 | 14 | 10 | 14 | 409 | 274 | 430 | 20 | 14 | 21 | 528 | 321 | 566 | 26 | 16 | 28 |
| 2017 | Shaanxi | 244 | 173 | 254 | 12 | 9 | 13 | 362 | 243 | 381 | 18 | 12 | 19 | 469 | 285 | 502 | 23 | 14 | 25 |
| 2010 | Shandong | 186 | 132 | 194 | 3 | 2 | 3 | 277 | 186 | 291 | 5 | 3 | 5 | 358 | 218 | 383 | 6 | 4 | 6 |
| 2011 | Shandong | 175 | 124 | 183 | 3 | 2 | 3 | 260 | 174 | 273 | 5 | 3 | 5 | 336 | 205 | 360 | 6 | 4 | 6 |
| 2012 | Shandong | 163 | 115 | 170 | 3 | 2 | 3 | 242 | 162 | 254 | 4 | 3 | 5 | 313 | 190 | 335 | 6 | 4 | 6 |
| 2013 | Shandong | 151 | 107 | 157 | 3 | 2 | 3 | 224 | 150 | 235 | 4 | 3 | 4 | 289 | 176 | 310 | 5 | 3 | 6 |
| 2014 | Shandong | 143 | 101 | 149 | 3 | 2 | 3 | 212 | 142 | 223 | 4 | 2 | 4 | 274 | 167 | 294 | 5 | 3 | 5 |
| 2015 | Shandong | 131 | 93 | 136 | 2 | 2 | 2 | 194 | 130 | 204 | 3 | 2 | 4 | 251 | 153 | 269 | 4 | 3 | 5 |
| 2016 | Shandong | 115 | 81 | 119 | 2 | 1 | 2 | 170 | 114 | 179 | 3 | 2 | 3 | 220 | 134 | 236 | 4 | 2 | 4 |
| 2017 | Shandong | 100 | 71 | 105 | 2 | 1 | 2 | 149 | 100 | 157 | 2 | 2 | 3 | 193 | 117 | 206 | 3 | 2 | 3 |
| 2010 | Shanghai | 42 | 30 | 44 | 5 | 3 | 5 | 63 | 42 | 66 | 7 | 5 | 8 | 81 | 50 | 87 | 9 | 6 | 10 |
| 2011 | Shanghai | 44 | 31 | 45 | 5 | 4 | 5 | 65 | 43 | 68 | 8 | 5 | 8 | 84 | 51 | 90 | 10 | 6 | 10 |
| 2012 | Shanghai | 44 | 31 | 45 | 5 | 3 | 5 | 65 | 43 | 68 | 7 | 5 | 7 | 84 | 51 | 90 | 9 | 6 | 10 |
| 2013 | Shanghai | 44 | 31 | 46 | 5 | 3 | 5 | 66 | 44 | 69 | 7 | 5 | 7 | 85 | 52 | 91 | 9 | 6 | 10 |
| 2014 | Shanghai | 45 | 32 | 47 | 5 | 3 | 5 | 67 | 45 | 70 | 7 | 5 | 7 | 86 | 53 | 92 | 9 | 5 | 10 |
| 2015 | Shanghai | 44 | 31 | 46 | 5 | 3 | 5 | 66 | 44 | 69 | 7 | 5 | 7 | 85 | 52 | 91 | 9 | 6 | 10 |
| 2016 | Shanghai | 43 | 31 | 45 | 5 | 3 | 5 | 64 | 43 | 68 | 7 | 5 | 7 | 83 | 51 | 89 | 9 | 5 | 9 |
| 2017 | Shanghai | 39 | 28 | 41 | 4 | 3 | 4 | 58 | 39 | 61 | 6 | 4 | 6 | 75 | 46 | 81 | 8 | 5 | 8 |
| 2010 | Shanxi | 312 | 221 | 325 | 15 | 10 | 15 | 463 | 311 | 487 | 22 | 15 | 23 | 599 | 364 | 641 | 28 | 17 | 30 |
| 2011 | Shanxi | 293 | 207 | 305 | 15 | 10 | 15 | 434 | 291 | 457 | 22 | 15 | 23 | 562 | 342 | 601 | 28 | 17 | 30 |
| 2012 | Shanxi | 274 | 194 | 286 | 15 | 11 | 15 | 407 | 273 | 428 | 22 | 15 | 23 | 526 | 320 | 563 | 28 | 17 | 31 |
| 2013 | Shanxi | 257 | 182 | 268 | 14 | 10 | 14 | 382 | 256 | 401 | 20 | 14 | 21 | 494 | 300 | 529 | 26 | 16 | 28 |
| 2014 | Shanxi | 249 | 176 | 259 | 13 | 9 | 13 | 369 | 248 | 388 | 19 | 13 | 20 | 478 | 290 | 511 | 25 | 15 | 26 |
| 2015 | Shanxi | 237 | 168 | 247 | 12 | 9 | 13 | 352 | 236 | 370 | 18 | 12 | 19 | 455 | 277 | 487 | 24 | 14 | 25 |
| 2016 | Shanxi | 218 | 155 | 227 | 11 | 8 | 12 | 324 | 217 | 341 | 17 | 11 | 18 | 419 | 255 | 449 | 22 | 13 | 24 |
| 2017 | Shanxi | 198 | 140 | 206 | 11 | 8 | 11 | 293 | 197 | 308 | 16 | 11 | 17 | 379 | 231 | 406 | 21 | 12 | 22 |
| 2010 | Sichuan | 1387 | 984 | 1446 | 30 | 21 | 31 | 2059 | 1381 | 2166 | 45 | 30 | 47 | 2663 | 1620 | 2852 | 58 | 35 | 62 |
| 2011 | Sichuan | 1210 | 858 | 1261 | 28 | 20 | 30 | 1796 | 1205 | 1889 | 42 | 28 | 44 | 2323 | 1413 | 2488 | 55 | 33 | 58 |
| 2012 | Sichuan | 1047 | 742 | 1091 | 25 | 18 | 26 | 1554 | 1042 | 1634 | 37 | 25 | 39 | 2009 | 1222 | 2152 | 47 | 29 | 51 |
| 2013 | Sichuan | 907 | 643 | 945 | 22 | 16 | 23 | 1346 | 903 | 1416 | 33 | 22 | 34 | 1741 | 1059 | 1865 | 42 | 26 | 45 |
| 2014 | Sichuan | 806 | 572 | 840 | 20 | 14 | 20 | 1197 | 803 | 1259 | 29 | 19 | 30 | 1547 | 941 | 1657 | 37 | 23 | 40 |
| 2015 | Sichuan | 707 | 501 | 737 | 17 | 12 | 18 | 1049 | 704 | 1104 | 26 | 17 | 27 | 1357 | 826 | 1453 | 33 | 20 | 35 |
| 2016 | Sichuan | 605 | 429 | 630 | 15 | 10 | 15 | 898 | 602 | 944 | 22 | 15 | 23 | 1161 | 706 | 1244 | 28 | 17 | 30 |
| 2017 | Sichuan | 515 | 365 | 536 | 12 | 9 | 13 | 764 | 513 | 804 | 18 | 12 | 19 | 988 | 601 | 1058 | 24 | 14 | 25 |
| 2010 | Tianjin | 40 | 28 | 41 | 6 | 4 | 7 | 59 | 39 | 62 | 9 | 6 | 10 | 76 | 46 | 82 | 12 | 7 | 13 |
| 2011 | Tianjin | 40 | 28 | 41 | 8 | 6 | 8 | 59 | 40 | 62 | 12 | 8 | 12 | 76 | 46 | 82 | 15 | 9 | 16 |
| 2012 | Tianjin | 40 | 28 | 41 | 8 | 5 | 8 | 59 | 40 | 62 | 11 | 8 | 12 | 76 | 46 | 82 | 15 | 9 | 16 |
| 2013 | Tianjin | 40 | 29 | 42 | 7 | 5 | 8 | 60 | 40 | 63 | 11 | 7 | 12 | 77 | 47 | 83 | 14 | 9 | 15 |
| 2014 | Tianjin | 42 | 30 | 43 | 7 | 5 | 8 | 62 | 42 | 65 | 11 | 7 | 11 | 80 | 49 | 86 | 14 | 9 | 15 |
| 2015 | Tianjin | 42 | 30 | 44 | 8 | 5 | 8 | 62 | 42 | 65 | 11 | 8 | 12 | 80 | 49 | 86 | 15 | 9 | 16 |
| 2016 | Tianjin | 41 | 29 | 42 | 7 | 5 | 7 | 60 | 41 | 64 | 10 | 7 | 11 | 78 | 48 | 84 | 13 | 8 | 14 |
| 2017 | Tianjin | 38 | 27 | 40 | 7 | 5 | 7 | 56 | 38 | 59 | 10 | 7 | 10 | 73 | 44 | 78 | 13 | 8 | 14 |
| 2010 | Tibet | 199 | 141 | 207 | 103 | 73 | 107 | 295 | 198 | 311 | 153 | 102 | 161 | 382 | 232 | 409 | 198 | 120 | 212 |
| 2011 | Tibet | 193 | 137 | 201 | 101 | 71 | 105 | 287 | 192 | 302 | 149 | 100 | 157 | 371 | 226 | 397 | 193 | 118 | 207 |
| 2012 | Tibet | 187 | 132 | 194 | 80 | 56 | 83 | 277 | 186 | 291 | 118 | 79 | 124 | 358 | 218 | 384 | 153 | 93 | 164 |
| 2013 | Tibet | 179 | 127 | 186 | 88 | 62 | 92 | 266 | 178 | 279 | 130 | 87 | 137 | 344 | 209 | 368 | 169 | 103 | 181 |
| 2014 | Tibet | 177 | 126 | 185 | 72 | 51 | 75 | 263 | 176 | 276 | 107 | 72 | 112 | 340 | 207 | 364 | 138 | 84 | 148 |
| 2015 | Tibet | 169 | 120 | 176 | 68 | 48 | 71 | 251 | 168 | 264 | 101 | 68 | 107 | 324 | 197 | 347 | 131 | 80 | 140 |
| 2016 | Tibet | 155 | 110 | 161 | 60 | 42 | 62 | 230 | 154 | 242 | 88 | 59 | 93 | 297 | 181 | 318 | 114 | 70 | 123 |
| 2017 | Tibet | 139 | 99 | 145 | 51 | 36 | 53 | 207 | 139 | 218 | 76 | 51 | 80 | 268 | 163 | 287 | 98 | 60 | 105 |
| 2010 | Xinjiang | 1118 | 793 | 1165 | 66 | 46 | 68 | 1659 | 1113 | 1745 | 97 | 65 | 102 | 2146 | 1305 | 2298 | 126 | 77 | 135 |
| 2011 | Xinjiang | 1058 | 750 | 1103 | 63 | 44 | 65 | 1571 | 1054 | 1653 | 93 | 62 | 98 | 2032 | 1236 | 2176 | 120 | 73 | 129 |
| 2012 | Xinjiang | 996 | 706 | 1038 | 55 | 39 | 58 | 1478 | 991 | 1555 | 82 | 55 | 87 | 1911 | 1163 | 2047 | 106 | 65 | 114 |
| 2013 | Xinjiang | 930 | 659 | 969 | 51 | 36 | 53 | 1380 | 926 | 1452 | 76 | 51 | 79 | 1785 | 1086 | 1912 | 98 | 59 | 105 |
| 2014 | Xinjiang | 894 | 634 | 932 | 48 | 34 | 50 | 1327 | 890 | 1396 | 71 | 48 | 75 | 1716 | 1044 | 1838 | 92 | 56 | 98 |
| 2015 | Xinjiang | 834 | 591 | 869 | 44 | 31 | 46 | 1238 | 831 | 1302 | 66 | 44 | 69 | 1601 | 974 | 1715 | 85 | 52 | 91 |
| 2016 | Xinjiang | 757 | 537 | 789 | 39 | 28 | 41 | 1123 | 754 | 1182 | 58 | 39 | 61 | 1453 | 884 | 1556 | 75 | 46 | 80 |
| 2017 | Xinjiang | 676 | 479 | 704 | 33 | 24 | 35 | 1003 | 673 | 1055 | 49 | 33 | 52 | 1297 | 789 | 1389 | 64 | 39 | 68 |
| 2010 | Yunnan | 1538 | 1091 | 1603 | 51 | 36 | 53 | 2283 | 1532 | 2402 | 76 | 51 | 80 | 2953 | 1796 | 3162 | 98 | 60 | 105 |
| 2011 | Yunnan | 1387 | 983 | 1445 | 48 | 34 | 50 | 2059 | 1381 | 2166 | 71 | 47 | 74 | 2662 | 1620 | 2851 | 91 | 56 | 98 |
| 2012 | Yunnan | 1235 | 876 | 1287 | 43 | 30 | 45 | 1833 | 1230 | 1928 | 63 | 43 | 67 | 2370 | 1442 | 2539 | 82 | 50 | 88 |
| 2013 | Yunnan | 1099 | 779 | 1145 | 38 | 27 | 40 | 1632 | 1095 | 1716 | 57 | 38 | 60 | 2110 | 1284 | 2260 | 73 | 45 | 79 |
| 2014 | Yunnan | 1009 | 715 | 1051 | 35 | 25 | 37 | 1498 | 1005 | 1575 | 52 | 35 | 55 | 1937 | 1178 | 2074 | 67 | 41 | 72 |
| 2015 | Yunnan | 905 | 642 | 943 | 32 | 23 | 33 | 1343 | 901 | 1413 | 47 | 32 | 50 | 1737 | 1057 | 1860 | 61 | 37 | 66 |
| 2016 | Yunnan | 803 | 569 | 837 | 28 | 20 | 29 | 1192 | 800 | 1254 | 42 | 28 | 44 | 1542 | 938 | 1651 | 54 | 33 | 58 |
| 2017 | Yunnan | 704 | 499 | 734 | 25 | 18 | 26 | 1046 | 701 | 1100 | 37 | 25 | 39 | 1352 | 823 | 1448 | 48 | 29 | 51 |
| 2010 | Zhejiang | 181 | 128 | 189 | 6 | 4 | 6 | 269 | 180 | 283 | 9 | 6 | 9 | 348 | 212 | 372 | 11 | 7 | 12 |
| 2011 | Zhejiang | 168 | 119 | 176 | 7 | 5 | 7 | 250 | 168 | 263 | 10 | 7 | 10 | 323 | 197 | 346 | 13 | 8 | 14 |
| 2012 | Zhejiang | 153 | 108 | 159 | 6 | 4 | 6 | 227 | 152 | 239 | 9 | 6 | 9 | 293 | 179 | 314 | 11 | 7 | 12 |
| 2013 | Zhejiang | 141 | 100 | 147 | 5 | 4 | 6 | 210 | 141 | 221 | 8 | 5 | 8 | 272 | 165 | 291 | 10 | 6 | 11 |
| 2014 | Zhejiang | 133 | 95 | 139 | 5 | 4 | 5 | 198 | 133 | 208 | 7 | 5 | 8 | 256 | 156 | 274 | 10 | 6 | 10 |
| 2015 | Zhejiang | 122 | 86 | 127 | 5 | 3 | 5 | 181 | 121 | 190 | 7 | 5 | 7 | 234 | 142 | 251 | 9 | 5 | 10 |
| 2016 | Zhejiang | 106 | 75 | 111 | 4 | 3 | 4 | 158 | 106 | 166 | 6 | 4 | 6 | 204 | 124 | 219 | 8 | 5 | 8 |
| 2017 | Zhejiang | 92 | 65 | 96 | 3 | 2 | 4 | 137 | 92 | 144 | 5 | 3 | 5 | 177 | 108 | 190 | 7 | 4 | 7 |
| 2010 | Central | 3863 | 2739 | 4026 | 12 | 8 | 12 | 5735 | 3847 | 6032 | 18 | 12 | 19 | 7416 | 4512 | 7943 | 23 | 14 | 25 |
| 2011 | Central | 3549 | 2516 | 3698 | 11 | 8 | 12 | 5268 | 3534 | 5541 | 17 | 11 | 18 | 6812 | 4144 | 7296 | 22 | 13 | 23 |
| 2012 | Central | 3242 | 2299 | 3379 | 10 | 7 | 11 | 4813 | 3229 | 5063 | 15 | 10 | 16 | 6224 | 3787 | 6666 | 20 | 12 | 21 |
| 2013 | Central | 2954 | 2095 | 3078 | 9 | 7 | 10 | 4385 | 2942 | 4613 | 14 | 9 | 15 | 5671 | 3450 | 6074 | 18 | 11 | 19 |
| 2014 | Central | 2767 | 1962 | 2883 | 9 | 6 | 9 | 4107 | 2755 | 4320 | 13 | 9 | 14 | 5311 | 3231 | 5689 | 17 | 10 | 18 |
| 2015 | Central | 2540 | 1801 | 2647 | 8 | 6 | 8 | 3771 | 2529 | 3966 | 12 | 8 | 13 | 4876 | 2966 | 5222 | 15 | 9 | 17 |
| 2016 | Central | 2271 | 1610 | 2367 | 7 | 5 | 7 | 3371 | 2261 | 3546 | 11 | 7 | 11 | 4360 | 2652 | 4669 | 14 | 8 | 15 |
| 2017 | Central | 2013 | 1427 | 2098 | 6 | 4 | 7 | 2988 | 2004 | 3143 | 9 | 6 | 10 | 3864 | 2351 | 4138 | 12 | 7 | 13 |
| 2010 | East | 1715 | 1216 | 1787 | 7 | 5 | 7 | 2546 | 1708 | 2678 | 10 | 7 | 10 | 3292 | 2003 | 3526 | 13 | 8 | 13 |
| 2011 | East | 1579 | 1120 | 1646 | 7 | 5 | 7 | 2345 | 1573 | 2466 | 10 | 7 | 11 | 3032 | 1845 | 3247 | 13 | 8 | 14 |
| 2012 | East | 1452 | 1030 | 1513 | 6 | 4 | 6 | 2156 | 1446 | 2267 | 9 | 6 | 9 | 2787 | 1696 | 2985 | 11 | 7 | 12 |
| 2013 | East | 1353 | 959 | 1410 | 5 | 4 | 6 | 2009 | 1347 | 2113 | 8 | 5 | 9 | 2597 | 1580 | 2782 | 11 | 6 | 11 |
| 2014 | East | 1293 | 917 | 1348 | 5 | 4 | 5 | 1920 | 1288 | 2019 | 8 | 5 | 8 | 2482 | 1510 | 2659 | 10 | 6 | 10 |
| 2015 | East | 1207 | 856 | 1258 | 5 | 3 | 5 | 1792 | 1202 | 1885 | 7 | 5 | 7 | 2317 | 1410 | 2482 | 9 | 6 | 10 |
| 2016 | East | 1087 | 771 | 1133 | 4 | 3 | 4 | 1614 | 1083 | 1698 | 6 | 4 | 7 | 2087 | 1270 | 2235 | 8 | 5 | 9 |
| 2017 | East | 969 | 687 | 1010 | 4 | 3 | 4 | 1439 | 965 | 1513 | 5 | 4 | 6 | 1861 | 1132 | 1993 | 7 | 4 | 8 |
| 2010 | West | 6968 | 4941 | 7262 | 30 | 21 | 31 | 10345 | 6939 | 10881 | 45 | 30 | 47 | 13377 | 8138 | 14327 | 58 | 35 | 62 |
| 2011 | West | 6287 | 4458 | 6552 | 28 | 20 | 29 | 9333 | 6260 | 9816 | 42 | 28 | 44 | 12069 | 7342 | 12926 | 54 | 33 | 58 |
| 2012 | West | 5641 | 4000 | 5879 | 25 | 18 | 27 | 8374 | 5617 | 8808 | 38 | 25 | 40 | 10829 | 6588 | 11598 | 49 | 30 | 52 |
| 2013 | West | 5059 | 3587 | 5272 | 23 | 16 | 24 | 7510 | 5037 | 7899 | 34 | 23 | 36 | 9711 | 5908 | 10401 | 44 | 27 | 47 |
| 2014 | West | 4671 | 3312 | 4867 | 21 | 15 | 21 | 6934 | 4651 | 7293 | 31 | 20 | 32 | 8966 | 5455 | 9603 | 39 | 24 | 42 |
| 2015 | West | 4225 | 2996 | 4403 | 19 | 13 | 20 | 6272 | 4207 | 6597 | 28 | 19 | 29 | 8110 | 4934 | 8686 | 36 | 22 | 39 |
| 2016 | West | 3733 | 2647 | 3891 | 17 | 12 | 17 | 5542 | 3718 | 5830 | 25 | 16 | 26 | 7167 | 4360 | 7676 | 32 | 19 | 34 |
| 2017 | West | 3271 | 2320 | 3409 | 14 | 10 | 15 | 4857 | 3258 | 5108 | 21 | 14 | 22 | 6280 | 3821 | 6726 | 27 | 17 | 29 |
| 2010 | National | 12546 | 8896 | 13075 | 15 | 11 | 16 | 18625 | 12493 | 19591 | 23 | 15 | 24 | 24085 | 14652 | 25796 | 30 | 18 | 32 |
| 2011 | National | 11414 | 8094 | 11896 | 15 | 10 | 15 | 16945 | 11367 | 17824 | 22 | 15 | 23 | 21913 | 13331 | 23469 | 28 | 17 | 30 |
| 2012 | National | 10335 | 7329 | 10771 | 13 | 9 | 14 | 15343 | 10292 | 16138 | 20 | 13 | 21 | 19841 | 12070 | 21250 | 25 | 15 | 27 |
| 2013 | National | 9366 | 6641 | 9760 | 12 | 8 | 12 | 13904 | 9326 | 14624 | 18 | 12 | 19 | 17980 | 10938 | 19257 | 23 | 14 | 25 |
| 2014 | National | 8730 | 6191 | 9098 | 11 | 8 | 11 | 12961 | 8694 | 13632 | 16 | 11 | 17 | 16760 | 10196 | 17950 | 21 | 13 | 22 |
| 2015 | National | 7972 | 5653 | 8308 | 10 | 7 | 10 | 11835 | 7938 | 12448 | 15 | 10 | 16 | 15304 | 9310 | 16391 | 19 | 12 | 21 |
| 2016 | National | 7091 | 5029 | 7390 | 9 | 6 | 9 | 10528 | 7062 | 11073 | 13 | 9 | 14 | 13614 | 8282 | 14581 | 17 | 10 | 18 |
| 2017 | National | 6253 | 4434 | 6517 | 8 | 5 | 8 | 9283 | 6227 | 9765 | 11 | 8 | 12 | 12005 | 7303 | 12857 | 15 | 9 | 16 |

**Webappendix 13: Sensitivity results of pneumococcal pneumonia mortality by different sources of all-cause pneumonia in China in 2017**

The present study used modelled provincial all-cause deaths and incidence cases of pneumonia and meningitis among children aged 1–59 months in 2010–17 in China. The data were prepared by Global Burden of Disease, Injuries, and Risk Factors Study (GBD) (Zhou et al. 2019) led by the Chinese CDC and Institute of Health Metrics and Evaluation. In the GBD study, lower respiratory infection was adopted to define pneumonia cases. The raw data of mortality and morbidity in children aged 1–59 months were mainly from the Disease Surveillance Point System maintained by Chinese CDC, the Maternal and Child Health Surveillance System maintained by National Office for Maternal and Child Health Surveillance of China, as well as various surveys, cancer registry, and censures in China. The methods adopted to derive these estimates are described elsewhere (He et al. 2016; Zhou et al. 2019; GBD 2016 Lower Respiratory Infections Collaborators 2018). There are no alternative data sources for provincial all-cause deaths and incidence cases of pneumonia and meningitis in China.

Wahl et al. (2018) estimated global burden of *Streptococcus pneumoniae* and *Haemophilus influenzae* type b disease among children in the era of conjugate vaccines: global, regional, and national estimates for 2000–15. Liu et al. (2016) used data from WHO and Maternal and Child Epidemiology Estimation (MCEE) to obtain country-specific estimates of all-cause pneumonia and meningitis mortality as well as pneumonia morbidity from 2000 to 2015 in China at the national level. However, no provincial estimates of all-cause pneumonia and meningitis mortality or pneumonia morbidity are available in China. We calculated a ratio at the national level of all-cause pneumonia deaths in children under 5 years old from GBD China to those from WHO MCEE, and then applied this ratio to provincial all-cause pneumonia deaths in children under 5 years old from GBD China to obtain provincial estimates of all-cause pneumonia deaths for WHO MCEE.

Song et al. (2016) conducted a systematic review to identify high-quality community-based longitudinal studies on cause of death (COD) in children younger than 5 years in China up to the year of 2015. They developed several single-cause models to predict the number of child deaths for main COD in different age groups at both national and provincial levels, including the proportion of all-cause pneumonia deaths in all-cause deaths among children under 5 years old. We multiplied provincial all-cause deaths with the proportion of all-cause pneumonia deaths at the national level from 2010 to 2015 (provincial proportions were not reported by Song et al. 2016), so as to obtain provincial all-cause pneumonia deaths in China from 2020 to 2015.

He et al. (2017) did a systematic analysis using adjusted empirical data on levels and causes of child mortality collected in the China Maternal and Child Health Surveillance System to generate representative estimates at the national and subnational levels. Similar to Song et al. (2016), they also reported the proportion of all-cause pneumonia for children under 5 years old in all-cause deaths at the national level. We multiplied provincial all-cause deaths in children under 5 years old with the proportion of all-cause pneumonia deaths at the national level from 2010 to 2015 (provincial proportions were not reported by He et al. 2017), so as to generate provincial all-cause pneumonia deaths in China from 2020 to 2015.

Song et al. (2016) and He et al. (2017) did not report the proportion of meningitis in all-cause deaths among children, as meningitis accounted for a very small proportion of all-cause deaths in China. The provincial all-cause pneumonia deaths estimated by above three approaches (Liu et al. 2016; Song et al. 2016; He et al. 2017) were not the actual numbers at the provincial level, so we only used them in the sensitivity analysis. The estimates at the national level might be more comparable than those at the provincial level. Liu et al. (2016), Song et al. (2016), and He et al. (2017) provided estimates in China up to 2015, and we employed linear interpolation of 2010–15 data to 2017. Estimates of meningitis deaths by the GBD China (Zhou et al. 2019) and WHO MCEE (Liu et al. 2016) were very similar, indicating that the sensitivity analysis of meningitis deaths was not necessary.

Webappendix 13.1 reported all-cause pneumonia and meningitis deaths from alternative data sources at the national level. All-cause meningitis deaths form GBD China and WHO MCEE were very similar, and their differences were rather small. All-cause pneumonia deaths from GBD China, WHO MCEE, estimates based on the proportion of Song et al. (2016), and estimates based on the proportion of He et al. (2017) were rather diverse. We admitted that the all-cause pneumonia deaths used in the present study would affect our estimates of pneumococcal and Hib deaths. Webappendix 13.2 showed that the above four approaches accessed all-cause pneumonia in different ways by including different ICD-10 codes.

**Webappendix 13.1 Data sources of all-cause pneumonia and meningitis at the national level in China**

| year | All cause pneumonia deaths | | | | All cause meningitis deaths | |
| --- | --- | --- | --- | --- | --- | --- |
|  | Data from GBD China, Zhou et al. 2019^1^ | Data from WHO MCEE, Liu et al. 2016^2^ | Data from a systematic review, Song et al. 2016^3^ | Data from China MCHSS, He et al. 2017^4^ | Data from GBD China, Zhou et al. 2019^1^ | Data from WHO MCEE, Liu et al. 2016^2^ |
| 2010 | 43266 | 26239 | 47346 | 39455 | 3324 | 3492 |
| 2011 | 39251 | 24115 | 42013 | 36656 | 3157 | 3280 |
| 2012 | 35432 | 22205 | 36400 | 33324 | 2950 | 2965 |
| 2013 | 31997 | 20528 | 32761 | 31547 | 2759 | 2714 |
| 2014 | 29798 | 18926 | 30815 | 28665 | 2610 | 2442 |
| 2015 | 27081 | 17860 | 27772 | 26876 | 2398 | 2315 |
| 2016 | 23940 | 16776 | 25286 | 25498 | 2138 | 2188 |
| 2017 | 21165 | 15782 | 23960 | 25221 | 1876 | 2062 |
| GBD: Global Disease Burden | | |  |  |  |  |
| WHO MCEE: World Health Organization Maternal and Child Epidemiology Estimation | | | | | | |
| China MCHSS: China Maternal and Child Health Surveillance System | | | | | | |
| 1 Zhou M, Wang H, Zeng X, et al. Mortality, morbidity, and risk factors in China and its provinces, 1990-2017: a systematic analysis for the Global Burden of Disease Study 2017. *Lancet* 2019; **394**: 1145–58.  2 Liu L, Oza S, Hogan D, et al. Global, regional, and national causes of under-5 mortality in 2000–15: an updated systematic analysis with implications for the Sustainable Development Goals. *Lancet* 2016; **388**: 3027–35  3 Song P, Theodoratou E, Li X, et al. Causes of death in children younger than five years in China in 2015: an updated analysis. *J Glob Health* 2016; **6**: 020802.  4 He C, Liu L, Chu Y, et al. National and subnational all-cause and cause-specific child mortality in China, 1996-2015: a systematic analysis with implications for the Sustainable Development Goals. *Lancet Glob Health* 2017; **5**: e186–e97. | | | | | | |

**Webappendix 13.2 Comparison of ICD-10 for all cause pneumonia**

| ICD-10 code | GBD China^1^ | WHO MCEE^1^ | China Maternal and Child Health Surveillance System (MCHSS) |
| --- | --- | --- | --- |
| A48.1 | Yes | No | No |
| A70 | Yes | No | No |
| B97.4–B97.6 | Yes | No | No |
| P23.0–P23.4 | Yes | Yes | Yes |
| P23.5–P23.9 | No | Yes | Yes |
| U04–U04.9 | Yes | No | No |
| J00–J08 | No | Yes | No |
| J09 | Yes | Yes | Yes |
| J10.0 | Yes | Yes | Yes |
| J10.1 | Yes | Yes | No |
| J10.8 | Yes | Yes | No |
| J11.0 | Yes | Yes | Yes |
| J11.1–J11.8 | Yes | Yes | No |
| J12.0–J16.8 | Yes | Yes | Yes |
| J17–J18 | No | Yes | Yes |
| J19 | Yes | Yes | No |
| J20–J21.9 | No | Yes | No |
| J22 | No | Yes | No |
| J85 | No | Yes | No |
| H65.0–H66.9 | No | Yes | No |
| Yes: ICD-10 codes were included in modelling pneumonia deaths. | | | |
| No: ICD-10 codes were not included in modelling pneumonia deaths. | | | |
| 1. ICD-10 codes with "Yes" were used in the general estimation modelling for the global study, but some ICD-10 codes might not used to record causes of deaths in China. | | | |

**Webappendix 13.3 Pneumococcal pneumonia deaths estimates by alternative data sources of all-cause pneumonia in 2017**

| Year | Province | All-cause pneumonia deaths data from GBD China, Zhou et al. 2019 | | | All-cause pneumonia deaths data from WHO MCEE, Liu et al. 2016 | | | All-cause pneumonia deaths data from a systematic review, Song et al. 2017 | | | All-cause pneumonia deaths data from China MCHSS, He et al. 2017 | | |
| --- | --- | --- | --- | --- | --- | --- | --- | --- | --- | --- | --- | --- | --- |
|  |  | Pneumococcal pneumonia deaths | UR | | Pneumococcal pneumonia deaths | UR | | Pneumococcal pneumonia deaths | UR | | Pneumococcal pneumonia deaths | UR | |
| 2017 | Anhui | 208 | 147 | 217 | 155 | 110 | 161 | 401 | 285 | 418 | 425 | 301 | 443 |
| 2017 | Beijing | 36 | 25 | 37 | 27 | 19 | 28 | 44 | 31 | 46 | 47 | 33 | 49 |
| 2017 | Chongqing | 84 | 60 | 88 | 63 | 45 | 65 | 167 | 119 | 174 | 177 | 126 | 185 |
| 2017 | Fujian | 115 | 82 | 120 | 86 | 61 | 90 | 168 | 119 | 175 | 178 | 126 | 185 |
| 2017 | Gansu | 211 | 150 | 220 | 157 | 112 | 164 | 283 | 201 | 295 | 300 | 212 | 312 |
| 2017 | Guangdong | 451 | 320 | 470 | 337 | 239 | 351 | 489 | 347 | 509 | 518 | 367 | 540 |
| 2017 | Guangxi | 234 | 166 | 243 | 174 | 124 | 182 | 380 | 269 | 396 | 402 | 285 | 419 |
| 2017 | Guizhou | 184 | 131 | 192 | 137 | 97 | 143 | 458 | 324 | 477 | 485 | 344 | 505 |
| 2017 | Hainan | 107 | 76 | 111 | 79 | 56 | 83 | 88 | 62 | 92 | 93 | 66 | 97 |
| 2017 | Hebei | 386 | 274 | 402 | 288 | 204 | 300 | 508 | 360 | 529 | 538 | 381 | 560 |
| 2017 | Heilongjiang | 67 | 47 | 69 | 50 | 35 | 52 | 100 | 71 | 105 | 106 | 75 | 111 |
| 2017 | Henan | 318 | 226 | 332 | 237 | 168 | 247 | 775 | 549 | 807 | 821 | 582 | 855 |
| 2017 | Hubei | 125 | 89 | 131 | 94 | 66 | 97 | 323 | 229 | 337 | 342 | 243 | 357 |
| 2017 | Hunan | 123 | 87 | 128 | 92 | 65 | 95 | 337 | 239 | 351 | 357 | 253 | 372 |
| 2017 | Inner Mongolia | 110 | 78 | 115 | 82 | 58 | 86 | 112 | 79 | 117 | 119 | 84 | 124 |
| 2017 | Jiangsu | 64 | 46 | 67 | 48 | 34 | 50 | 230 | 163 | 239 | 243 | 173 | 254 |
| 2017 | Jiangxi | 405 | 287 | 422 | 302 | 214 | 315 | 385 | 273 | 402 | 408 | 289 | 425 |
| 2017 | Jilin | 77 | 54 | 80 | 57 | 41 | 60 | 61 | 43 | 63 | 64 | 46 | 67 |
| 2017 | Liaoning | 32 | 23 | 34 | 24 | 17 | 25 | 84 | 59 | 87 | 89 | 63 | 93 |
| 2017 | Ningxia | 63 | 45 | 66 | 47 | 33 | 49 | 63 | 44 | 65 | 66 | 47 | 69 |
| 2017 | Qinghai | 107 | 76 | 112 | 80 | 57 | 83 | 69 | 49 | 72 | 73 | 52 | 76 |
| 2017 | Shaanxi | 244 | 173 | 254 | 182 | 129 | 190 | 272 | 193 | 284 | 288 | 204 | 300 |
| 2017 | Shandong | 100 | 71 | 105 | 75 | 53 | 78 | 622 | 441 | 648 | 658 | 467 | 686 |
| 2017 | Shanghai | 39 | 28 | 41 | 29 | 21 | 30 | 57 | 40 | 59 | 60 | 43 | 63 |
| 2017 | Shanxi | 198 | 140 | 206 | 147 | 104 | 153 | 186 | 132 | 194 | 197 | 140 | 205 |
| 2017 | Sichuan | 515 | 365 | 536 | 384 | 272 | 400 | 533 | 378 | 556 | 565 | 401 | 589 |
| 2017 | Tianjin | 38 | 27 | 40 | 28 | 20 | 30 | 38 | 27 | 39 | 40 | 28 | 42 |
| 2017 | Tibet | 139 | 99 | 145 | 104 | 74 | 108 | 86 | 61 | 90 | 91 | 65 | 95 |
| 2017 | Xinjiang | 676 | 479 | 704 | 504 | 357 | 525 | 465 | 330 | 484 | 492 | 349 | 513 |
| 2017 | Yunnan | 704 | 499 | 734 | 525 | 372 | 547 | 450 | 319 | 469 | 477 | 338 | 497 |
| 2017 | Zhejiang | 92 | 65 | 96 | 69 | 49 | 72 | 192 | 136 | 200 | 203 | 144 | 212 |
| 2017 | Central | 2013 | 1427 | 2098 | 1501 | 1064 | 1564 | 3164 | 2243 | 3297 | 3351 | 2376 | 3493 |
| 2017 | East | 969 | 687 | 1010 | 723 | 512 | 753 | 1922 | 1363 | 2003 | 2036 | 1444 | 2122 |
| 2017 | West | 3271 | 2320 | 3409 | 2439 | 1730 | 2542 | 3337 | 2366 | 3478 | 3535 | 2507 | 3684 |
| 2017 | National | 6253 | 4434 | 6517 | 4663 | 3307 | 4860 | 8423 | 5973 | 8778 | 8922 | 6327 | 9299 |

**Webappendix 13.4 Hib pneumonia deaths estimates by alternative data sources of all-cause pneumonia in 2017**

| Year | Province | All-cause pneumonia deaths data from GBD China, Zhou et al. 2019 | | | All-cause pneumonia deaths data from WHO MCEE, Liu et al. 2016 | | | All-cause pneumonia deaths data from a systematic review, Song et al. 2017 | | | All-cause pneumonia deaths data from China MCHSS, He et al. 2017 | | |
| --- | --- | --- | --- | --- | --- | --- | --- | --- | --- | --- | --- | --- | --- |
|  |  | Hib pneumonia deaths | UR | | Hib pneumonia deaths | UR | | Hib pneumonia deaths | UR | | Hib pneumonia deaths | UR | |
| 2017 | Beijing | 13 | 9 | 17 | 10 | 7 | 13 | 24 | 17 | 32 | 27 | 19 | 35 |
| 2017 | Chongqing | 27 | 19 | 36 | 20 | 14 | 27 | 77 | 54 | 101 | 86 | 60 | 113 |
| 2017 | Fujian | 46 | 32 | 60 | 34 | 24 | 45 | 100 | 71 | 132 | 112 | 79 | 147 |
| 2017 | Gansu | 119 | 84 | 156 | 89 | 62 | 116 | 220 | 155 | 290 | 246 | 173 | 323 |
| 2017 | Guangdong | 125 | 88 | 164 | 93 | 65 | 122 | 213 | 150 | 280 | 238 | 167 | 312 |
| 2017 | Guangxi | 93 | 65 | 122 | 69 | 49 | 91 | 219 | 154 | 288 | 244 | 172 | 321 |
| 2017 | Guizhou | 82 | 57 | 107 | 61 | 43 | 80 | 297 | 209 | 390 | 331 | 233 | 435 |
| 2017 | Hainan | 44 | 31 | 58 | 33 | 23 | 43 | 53 | 37 | 69 | 59 | 41 | 77 |
| 2017 | Hebei | 172 | 121 | 226 | 128 | 90 | 169 | 327 | 230 | 430 | 365 | 257 | 480 |
| 2017 | Heilongjiang | 26 | 19 | 35 | 20 | 14 | 26 | 58 | 41 | 76 | 65 | 46 | 85 |
| 2017 | Henan | 111 | 78 | 146 | 83 | 58 | 109 | 386 | 271 | 507 | 430 | 303 | 565 |
| 2017 | Hubei | 38 | 26 | 49 | 28 | 20 | 37 | 138 | 97 | 181 | 154 | 108 | 202 |
| 2017 | Hunan | 52 | 36 | 68 | 39 | 27 | 51 | 206 | 145 | 270 | 229 | 161 | 301 |
| 2017 | Inner Mongolia | 55 | 39 | 73 | 41 | 29 | 54 | 83 | 58 | 108 | 92 | 65 | 121 |
| 2017 | Jiangsu | 31 | 22 | 41 | 23 | 16 | 31 | 167 | 118 | 220 | 187 | 131 | 245 |
| 2017 | Jiangxi | 149 | 105 | 196 | 111 | 78 | 146 | 204 | 143 | 268 | 227 | 160 | 298 |
| 2017 | Jilin | 33 | 23 | 43 | 25 | 17 | 32 | 39 | 27 | 51 | 44 | 31 | 57 |
| 2017 | Liaoning | 14 | 10 | 19 | 11 | 8 | 14 | 54 | 38 | 71 | 61 | 43 | 80 |
| 2017 | Ningxia | 34 | 24 | 44 | 25 | 18 | 33 | 48 | 34 | 63 | 54 | 38 | 71 |
| 2017 | Qinghai | 58 | 41 | 76 | 43 | 30 | 56 | 54 | 38 | 71 | 61 | 43 | 80 |
| 2017 | Shaanxi | 120 | 84 | 158 | 89 | 63 | 118 | 192 | 135 | 253 | 214 | 151 | 282 |
| 2017 | Shandong | 39 | 27 | 51 | 29 | 20 | 38 | 350 | 246 | 459 | 390 | 274 | 512 |
| 2017 | Shanghai | 6 | 4 | 8 | 5 | 3 | 6 | 14 | 10 | 18 | 15 | 11 | 20 |
| 2017 | Shanxi | 100 | 70 | 132 | 75 | 52 | 98 | 131 | 92 | 172 | 146 | 103 | 192 |
| 2017 | Sichuan | 154 | 108 | 203 | 115 | 81 | 151 | 230 | 162 | 303 | 257 | 181 | 338 |
| 2017 | Tianjin | 10 | 7 | 13 | 7 | 5 | 10 | 15 | 10 | 19 | 16 | 12 | 21 |
| 2017 | Tibet | 77 | 54 | 102 | 58 | 41 | 76 | 70 | 49 | 92 | 78 | 55 | 102 |
| 2017 | Xinjiang | 388 | 273 | 510 | 290 | 204 | 380 | 377 | 265 | 496 | 420 | 296 | 552 |
| 2017 | Yunnan | 305 | 215 | 401 | 228 | 160 | 299 | 285 | 201 | 375 | 318 | 224 | 418 |
| 2017 | Zhejiang | 30 | 21 | 39 | 22 | 16 | 29 | 92 | 65 | 121 | 103 | 72 | 135 |
| 2017 | Central | 804 | 565 | 1056 | 600 | 422 | 788 | 1760 | 1238 | 2312 | 1962 | 1380 | 2578 |
| 2017 | East | 314 | 221 | 413 | 234 | 165 | 308 | 1030 | 724 | 1353 | 1148 | 807 | 1509 |
| 2017 | West | 1512 | 1064 | 1987 | 1128 | 793 | 1482 | 2153 | 1514 | 2828 | 2400 | 1688 | 3153 |
| 2017 | National | 2631 | 1850 | 3457 | 1962 | 1380 | 2578 | 4942 | 3476 | 6494 | 5509 | 3875 | 7239 |

**Webappendix 14: PCV and Hib vaccine coverage by alternative estimation methods in China in 2017**

In China, children are recommended to vaccinate 4 doses of Hib vaccine and 4 doses of PCV. The total number of Hib vaccine and PCV doses in 31 provinces was recorded by the Chinese CDC in 2010–17. We firstly allocated the total vaccine doses by provinces to eligible children with 1, 2, 3 and 4 doses, and then calculated the weighted vaccine coverage by vaccine dose specific efficacy. We conducted a nationally representative survey in 10 provinces in China by collecting vaccination records of more than 6 000 children in 2019. In each province, both rural and urban sites were approached to join the survey. We calculated the distribution of doses received (1, 2, 3, or 4 doses) among those with at least 1 dose in the survey, by vaccine (**Webappendix 14.1**). Most children living in urban cities that were vaccinated with Hib vaccine or PCV received 3 or 4 doses, while those living in rural areas usually received only 1 or 2 doses. The dose distribution was used to allocate the total number of doses administered in each province to children who received 1, 2, 3, and 4 doses. For those provinces not approached in the survey, we used data from a neighboring surveyed province to estimate vaccine coverage in view of their similar socioeconomic development status. Specifically, data from Beijing were used for Tianjin; Jilin data were used for Heilongjiang and Liaoning; Shandong data were used for Hebei; Shanghai data were used for Jiangsu and Zhejiang; Guangdong data were used for Fujian and Hainan; Yunnan data were used for Guizhou and Guangxi; Chongqing data were used for Sichuan, Hubei, and Hunan; Gansu data were used for Inner Mongolia, Ningxia, Qinghai, Xinjiang, and Tibet; Henan data were used for Shanxi and Shaanxi; Jiangxi data were used for Anhui. The number of doses was divided by the number of neonates in each province to estimate dose-specific coverage.

**Webappendix 14.1. Distribution in the number of doses received in a survey of 10 Provinces**

| Province | Rural/ | PCV | | | | |  | Hib Vaccine | | | | |
| --- | --- | --- | --- | --- | --- | --- | --- | --- | --- | --- | --- | --- |
|  | Urban | 0 Dose | 1 Dose | 2 Dose | 3 Dose | 4 Dose |  | 0 Dose | 1 Dose | 2 Dose | 3 Dose | 4 Dose |
| Beijing | Rural | 0.979 | 0.007 | 0.014 | 0.000 | 0.000 |  | 0.957 | 0.036 | 0.007 | 0.000 | 0.000 |
| Beijing | Urban | 0.876 | 0.010 | 0.010 | 0.000 | 0.105 |  | 0.857 | 0.034 | 0.030 | 0.010 | 0.069 |
| Chongqing | Rural | 0.991 | 0.000 | 0.000 | 0.000 | 0.009 |  | 0.680 | 0.136 | 0.058 | 0.078 | 0.049 |
| Chongqing | Urban | 0.927 | 0.013 | 0.009 | 0.009 | 0.043 |  | 0.416 | 0.118 | 0.050 | 0.149 | 0.267 |
| Gansu | Rural | 1.000 | 0.000 | 0.000 | 0.000 | 0.000 |  | 0.993 | 0.000 | 0.007 | 0.000 | 0.000 |
| Gansu | Urban | 0.978 | 0.011 | 0.000 | 0.000 | 0.011 |  | 0.470 | 0.157 | 0.084 | 0.096 | 0.193 |
| Guangdong | Rural | 1.000 | 0.000 | 0.000 | 0.000 | 0.000 |  | 0.558 | 0.221 | 0.106 | 0.097 | 0.018 |
| Guangdong | Urban | 0.925 | 0.009 | 0.004 | 0.018 | 0.044 |  | 0.595 | 0.098 | 0.068 | 0.083 | 0.156 |
| Henan | Rural | 0.980 | 0.010 | 0.000 | 0.010 | 0.000 |  | 0.396 | 0.167 | 0.104 | 0.208 | 0.125 |
| Henan | Urban | 0.958 | 0.017 | 0.000 | 0.004 | 0.021 |  | 0.258 | 0.044 | 0.067 | 0.262 | 0.369 |
| Jiangxi | Rural | 0.989 | 0.000 | 0.005 | 0.000 | 0.005 |  | 0.337 | 0.116 | 0.105 | 0.250 | 0.192 |
| Jiangxi | Urban | 0.935 | 0.000 | 0.018 | 0.012 | 0.036 |  | 0.497 | 0.134 | 0.064 | 0.140 | 0.166 |
| Jilin | Rural | 0.989 | 0.011 | 0.000 | 0.000 | 0.000 |  | 0.826 | 0.150 | 0.012 | 0.012 | 0.000 |
| Jilin | Urban | 0.989 | 0.000 | 0.005 | 0.000 | 0.005 |  | 0.901 | 0.064 | 0.023 | 0.006 | 0.006 |
| Shandong | Rural | 1.000 | 0.000 | 0.000 | 0.000 | 0.000 |  | 0.306 | 0.176 | 0.074 | 0.204 | 0.241 |
| Shandong | Urban | 0.991 | 0.000 | 0.000 | 0.000 | 0.009 |  | 0.671 | 0.089 | 0.089 | 0.067 | 0.084 |
| Shanghai | Rural | 0.939 | 0.037 | 0.000 | 0.012 | 0.012 |  | 0.287 | 0.200 | 0.050 | 0.175 | 0.287 |
| Shanghai | Urban | 0.834 | 0.008 | 0.012 | 0.033 | 0.112 |  | 0.260 | 0.064 | 0.021 | 0.089 | 0.566 |
| Yunnan | Rural | 1.000 | 0.000 | 0.000 | 0.000 | 0.000 |  | 0.598 | 0.174 | 0.092 | 0.071 | 0.065 |
| Yunnan | Urban | 0.959 | 0.005 | 0.005 | 0.026 | 0.005 |  | 0.282 | 0.110 | 0.072 | 0.243 | 0.293 |

The disease burden model used effectiveness for 3-dose coverage, so we adjusted the vaccination status of children who received 1 or 2 doses by the dose-specific efficacy relative to that of 3 doses from clinical trials (Griffiths et al. 2012 for Hib vaccine and Whitney, et al. 2006 for PCV). The efficacy of 3 doses Hib vaccine and PCV was 0.95 and 0.80, respectively (Watt et al. 2009; Lucero, et al. 2009). Overall vaccine coverage was then estimated using the calculated results to provide a weighted total (“Base” method), and also by simply dividing the total doses by 3 per eligible child (“Alternative” method).

**Webappendix 14.2. Effective coverage by Province of Hib and pneumococcal conjugate vaccines**

| Province | Hib vaccine coverage | | |  | | PCV coverage | |
| --- | --- | --- | --- | --- | --- | --- | --- |
|  | Weighted total doses^1^ | Total doses/(3 doses*neonatal population)^2^ |  | | Weighted total doses^3^ | | Total doses/(3 doses*neonatal population)^2^ |
|  | Base method | Alternative method |  | | Base method | | Alternative method |
| Anhui | 0.3609 | 0.3575 |  | | 0.0045 | | 0.0049 |
| Beijing | 0.3859 | 0.3707 |  | | 0.0985 | | 0.1149 |
| Chongqing | 0.4727 | 0.4849 |  | | 0.0208 | | 0.0228 |
| Fujian | 0.2950 | 0.2681 |  | | 0.0120 | | 0.0136 |
| Gansu | 0.0616 | 0.0598 |  | | 0.0009 | | 0.0009 |
| Guangdong | 0.5032 | 0.4573 |  | | 0.0212 | | 0.0240 |
| Guangxi | 0.3203 | 0.3139 |  | | 0.0029 | | 0.0028 |
| Guizhou | 0.2286 | 0.2240 |  | | 0.0040 | | 0.0038 |
| Hainan | 0.2954 | 0.2684 |  | | 0.0139 | | 0.0157 |
| Hebei | 0.2328 | 0.2228 |  | | 0.0025 | | 0.0034 |
| Heilongjiang | 0.3214 | 0.1994 |  | | 0.0137 | | 0.0104 |
| Henan | 0.4206 | 0.4514 |  | | 0.0065 | | 0.0062 |
| Hubei | 0.5116 | 0.5248 |  | | 0.0102 | | 0.0112 |
| Hunan | 0.2778 | 0.2850 |  | | 0.0080 | | 0.0087 |
| Inner Mongolia | 0.1142 | 0.1109 |  | | 0.0001 | | 0.0001 |
| Jiangsu | 0.1316 | 0.1554 |  | | 0.0171 | | 0.0195 |
| Jiangxi | 0.3808 | 0.3772 |  | | 0.0045 | | 0.0048 |
| Jilin | 0.2342 | 0.1453 |  | | 0.0045 | | 0.0034 |
| Liaoning | 0.2363 | 0.1466 |  | | 0.0204 | | 0.0154 |
| Ningxia | 0.0750 | 0.0728 |  | | 0.0044 | | 0.0041 |
| Qinghai | 0.0500 | 0.0485 |  | | 0.0001 | | 0.0001 |
| Shaanxi | 0.1576 | 0.1692 |  | | 0.0102 | | 0.0098 |
| Shandong | 0.3382 | 0.3236 |  | | 0.0039 | | 0.0051 |
| Shanghai | 0.7583 | 0.8956 |  | | 0.1020 | | 0.1164 |
| Shanxi | 0.1580 | 0.1696 |  | | 0.0019 | | 0.0018 |
| Sichuan | 0.5063 | 0.5194 |  | | 0.0145 | | 0.0159 |
| Tianjin | 0.5634 | 0.5412 |  | | 0.0297 | | 0.0346 |
| Tibet | 0.0237 | 0.0230 |  | | 0.0000 | | 0.0000 |
| Xinjiang | 0.0206 | 0.0200 |  | | 0.0027 | | 0.0025 |
| Yunnan | 0.2484 | 0.2434 |  | | 0.0084 | | 0.0079 |
| Zhejiang | 0.4517 | 0.5335 |  | | 0.0556 | | 0.0634 |
| **Central** | 0.3426 | 0.3437 |  | | 0.0061 | | 0.0064 |
| **East** | 0.3809 | 0.3735 |  | | 0.0250 | | 0.0283 |
| **West** | 0.2625 | 0.2640 |  | | 0.0074 | | 0.0076 |
| **National** | 0.3337 | 0.3319 |  | | 0.0132 | | 0.0145 |
| 1. Provincial Hib vaccine rate of j doses = provincial total Hib vaccine dose number for children with j doses/(j doses*provincial total number of neonatal children). Provincial weighted Hib vaccine rate = provincial Hib vaccine 1 dose rate*(0.59/0.93) + provincial Hib vaccine 2 dose rate*(0.92/0.93) + provincial Hib vaccine 3 dose rate + provincial Hib vaccine 4 dose rate. | | | | | | | |
| 1. Provincial coverage = provincial total doses / (3 doses* provincial total number of neonatal children) | | | | | | | |
| 1. Provincial PCV rate of j doses = provincial total PCV dose number for children with j doses/(j doses*provincial total number of neonatal children). Provincial weighted PCV rate = provincial PCV 1 dose rate*(0.73/0.95) + provincial PCV 2 dose rate + provincial PCV 3 dose rate + provincial PCV 4 dose rate. | | | | | | | |

**References:**

1 Griffiths UK, Clark A, Gessner B, et al. Dose-specific efficacy of Haemophilus influenzae type b conjugate vaccines: a systematic review and meta-analysis of controlled clinical trials. *Epidemiol Infect* 2012; **140**: 1343–55.

2 Watt JP, Wolfson LJ, O'Brien KL, et al. Burden of disease caused by Haemophilus influenzae type b in children younger than 5 years: global estimates. *Lancet* 2009; **374**: 903–11.

3 Whitney CG, Pilishvili T, Farley MM, et al. Effectiveness of seven-valent pneumococcal conjugate vaccine against invasive pneumococcal disease: a matched case-control study. *Lancet* 2006; **368**: 1495–502.

4 Lucero MG, Dulalia VE, Nillos LT, et al. Pneumococcal conjugate vaccines for preventing vaccine-type invasive pneumococcal disease and X-ray defined pneumonia in children less than two years of age. *Cochrane Database Syst Rev* 2009: CD004977.

**Webappendix 15: Sensitivity results of disease burden in 2017 by adopting the same vaccine coverage rates in 2010**

In the present study, pathogen-specific pneumococcal and Hib disease burden was adjusted to account for provincial vaccine coverage. We also calculated the disease burden in 2017 by adopting the same three-dose vaccine coverage rates in 2010, i.e. reducing the higher vaccine coverage in 2017 to lower levels of coverage (those in 2010) to visually display the effects of vaccination. Webappendix 15.1, 15.2, 15.3, 15.4 reported Streptococcus pneumoniae and Haemophilus influenzae type b deaths and cases at the provincial level in 2017 by adopting vaccine coverage rates in both 2010 (assumed) and 2017 (real-world). Pneumococcal deaths and cases were very similar when applying PCV coverage rates in 2010 to the 2017 population since the vaccine coverage rates have remained suboptimal in the past decade.

**Webappendix 15.1 Streptococcus pneumoniae mortality in Chinese children aged 1–59 months in 2017 by adopting vaccine coverage rates in 2010 and 2017**

| **Province** | **Streptococcus pneumoniae deaths** | | | | **Streptococcus pneumoniae pneumonia deaths** | | | | **Streptococcus pneumoniae meningitis deaths** | | | | **Streptococcus pneumoniae severe NPNM deaths** | | | |
| --- | --- | --- | --- | --- | --- | --- | --- | --- | --- | --- | --- | --- | --- | --- | --- | --- |
|  | **Number** | | **Rate per 100 000** | | **Number** | | **Rate per 100 000** | | **Number** | | **Rate per 100 000** | | **Number** | | **Rate per 100 000** | |
|  | **2010** | **2017** | **2010** | **2017** | **2010** | **2017** | **2010** | **2017** | **2010** | **2017** | **2010** | **2017** | **2010** | **2017** | **2010** | **2017** |
| Anhui | 278 | 277 | 7 | 7 | 208 | 208 | 5 | 5 | 37 | 37 | 1 | 1 | 33 | 33 | 1 | 1 |
| Beijing | 47 | 45 | 5 | 5 | 37 | 36 | 4 | 4 | 5 | 5 | 1 | 1 | 5 | 4 | 0 | 0 |
| Chongqing | 109 | 107 | 7 | 7 | 85 | 84 | 5 | 5 | 12 | 12 | 1 | 1 | 11 | 11 | 1 | 1 |
| Fujian | 150 | 149 | 6 | 6 | 116 | 115 | 5 | 5 | 18 | 18 | 1 | 1 | 16 | 16 | 1 | 1 |
| Gansu | 285 | 284 | 19 | 19 | 211 | 211 | 14 | 14 | 39 | 39 | 3 | 3 | 35 | 35 | 2 | 2 |
| Guangdong | 586 | 580 | 8 | 8 | 456 | 451 | 6 | 6 | 69 | 68 | 1 | 1 | 61 | 60 | 1 | 1 |
| Guangxi | 290 | 290 | 7 | 7 | 234 | 234 | 6 | 6 | 30 | 30 | 1 | 1 | 27 | 26 | 1 | 1 |
| Guizhou | 217 | 217 | 8 | 8 | 184 | 184 | 7 | 7 | 17 | 17 | 1 | 1 | 15 | 15 | 1 | 1 |
| Hainan | 128 | 127 | 19 | 19 | 107 | 107 | 16 | 16 | 11 | 11 | 2 | 2 | 10 | 10 | 1 | 1 |
| Hebei | 583 | 583 | 11 | 11 | 386 | 386 | 7 | 7 | 104 | 104 | 2 | 2 | 93 | 93 | 2 | 2 |
| Heilongjiang | 101 | 101 | 9 | 9 | 67 | 67 | 6 | 6 | 18 | 18 | 2 | 2 | 16 | 16 | 1 | 1 |
| Henan | 463 | 461 | 6 | 6 | 319 | 318 | 4 | 4 | 76 | 76 | 1 | 1 | 68 | 67 | 1 | 1 |
| Hubei | 155 | 154 | 5 | 5 | 126 | 125 | 4 | 4 | 15 | 15 | 0 | 0 | 14 | 14 | 0 | 0 |
| Hunan | 151 | 151 | 3 | 3 | 123 | 123 | 3 | 3 | 15 | 15 | 0 | 0 | 13 | 13 | 0 | 0 |
| Inner Mongolia | 156 | 156 | 15 | 15 | 110 | 110 | 10 | 10 | 24 | 24 | 2 | 2 | 21 | 21 | 2 | 2 |
| Jiangsu | 91 | 90 | 2 | 2 | 65 | 64 | 2 | 2 | 14 | 13 | 0 | 0 | 12 | 12 | 0 | 0 |
| Jiangxi | 515 | 514 | 16 | 16 | 406 | 405 | 13 | 13 | 58 | 57 | 2 | 2 | 51 | 51 | 2 | 2 |
| Jilin | 116 | 116 | 12 | 12 | 77 | 77 | 8 | 8 | 21 | 21 | 2 | 2 | 19 | 19 | 2 | 2 |
| Liaoning | 47 | 47 | 3 | 3 | 33 | 32 | 2 | 2 | 8 | 8 | 1 | 1 | 7 | 7 | 0 | 0 |
| Ningxia | 79 | 79 | 17 | 17 | 63 | 63 | 14 | 14 | 8 | 8 | 2 | 2 | 7 | 7 | 2 | 2 |
| Qinghai | 135 | 135 | 36 | 36 | 107 | 107 | 28 | 28 | 15 | 15 | 4 | 4 | 13 | 13 | 3 | 3 |
| Shaanxi | 329 | 328 | 16 | 16 | 245 | 244 | 12 | 12 | 44 | 44 | 2 | 2 | 40 | 39 | 2 | 2 |
| Shandong | 158 | 158 | 3 | 3 | 101 | 100 | 2 | 2 | 30 | 30 | 0 | 0 | 27 | 27 | 0 | 0 |
| Shanghai | 52 | 50 | 5 | 5 | 41 | 39 | 4 | 4 | 6 | 6 | 1 | 1 | 5 | 5 | 1 | 1 |
| Shanxi | 270 | 269 | 15 | 15 | 198 | 198 | 11 | 11 | 38 | 38 | 2 | 2 | 34 | 34 | 2 | 2 |
| Sichuan | 615 | 610 | 15 | 15 | 518 | 515 | 12 | 12 | 51 | 51 | 1 | 1 | 45 | 45 | 1 | 1 |
| Tianjin | 49 | 48 | 8 | 8 | 38 | 38 | 7 | 7 | 5 | 5 | 1 | 1 | 5 | 5 | 1 | 1 |
| Tibet | 157 | 157 | 57 | 57 | 139 | 139 | 51 | 51 | 9 | 9 | 3 | 3 | 8 | 8 | 3 | 3 |
| Xinjiang | 782 | 782 | 38 | 38 | 676 | 676 | 33 | 33 | 56 | 56 | 3 | 3 | 50 | 50 | 2 | 2 |
| Yunnan | 823 | 821 | 29 | 29 | 706 | 704 | 25 | 25 | 62 | 61 | 2 | 2 | 55 | 55 | 2 | 2 |
| Zhejiang | 129 | 127 | 5 | 5 | 94 | 92 | 4 | 3 | 19 | 18 | 1 | 1 | 17 | 16 | 1 | 1 |
| Central | 2760 | 2753 | 9 | 9 | 2017 | 2013 | 6 | 6 | 393 | 392 | 1 | 1 | 350 | 349 | 1 | 1 |
| East | 1308 | 1292 | 5 | 5 | 981 | 969 | 4 | 4 | 173 | 171 | 1 | 1 | 154 | 152 | 1 | 1 |
| West | 3975 | 3965 | 17 | 17 | 3280 | 3271 | 14 | 14 | 368 | 367 | 2 | 2 | 328 | 326 | 1 | 1 |
| National | 8043 | 8010 | 10 | 10 | 6278 | 6253 | 8 | 8 | 934 | 929 | 1 | 1 | 831 | 827 | 1 | 1 |

**Webappendix 15.2 Haemophilus influenzae type b mortality in Chinese children aged 1–59 months in 2017 by adopting vaccine coverage rates in 2010 and 2017**

| **Province** | **Hib deaths** | | | | **Hib pneumonia deaths** | | | | **Hib meningitis deaths** | | | | **Hib severe NPNM deaths** | | | |
| --- | --- | --- | --- | --- | --- | --- | --- | --- | --- | --- | --- | --- | --- | --- | --- | --- |
|  | **Number** | | **Rate per 100 000** | | **Number** | | **Rate per 100 000** | | **Number** | | **Rate per 100 000** | | **Number** | | **Rate per 100 000** | |
|  | **2010** | **2017** | **2010** | **2017** | **2010** | **2017** | **2010** | **2017** | **2010** | **2017** | **2010** | **2017** | **2010** | **2017** | **2010** | **2017** |
| Anhui | 102 | 87 | 3 | 2 | 92 | 79 | 2 | 2 | 10 | 8 | 0 | 0 | 0 | 0 | 0 | 0 |
| Beijing | 17 | 14 | 2 | 2 | 15 | 13 | 2 | 1 | 1 | 1 | 0 | 0 | 0 | 0 | 0 | 0 |
| Chongqing | 38 | 30 | 2 | 2 | 35 | 27 | 2 | 2 | 3 | 2 | 0 | 0 | 0 | 0 | 0 | 0 |
| Fujian | 51 | 50 | 2 | 2 | 47 | 46 | 2 | 2 | 4 | 4 | 0 | 0 | 0 | 0 | 0 | 0 |
| Gansu | 139 | 133 | 9 | 9 | 124 | 119 | 8 | 8 | 15 | 14 | 1 | 1 | 0 | 0 | 0 | 0 |
| Guangdong | 149 | 136 | 2 | 2 | 136 | 125 | 2 | 2 | 12 | 11 | 0 | 0 | 0 | 0 | 0 | 0 |
| Guangxi | 114 | 100 | 3 | 2 | 106 | 93 | 3 | 2 | 8 | 7 | 0 | 0 | 0 | 0 | 0 | 0 |
| Guizhou | 105 | 86 | 4 | 3 | 99 | 82 | 4 | 3 | 6 | 5 | 0 | 0 | 0 | 0 | 0 | 0 |
| Hainan | 59 | 47 | 9 | 7 | 55 | 44 | 8 | 7 | 3 | 3 | 1 | 0 | 0 | 0 | 0 | 0 |
| Hebei | 220 | 201 | 4 | 4 | 189 | 172 | 4 | 3 | 31 | 28 | 1 | 1 | 0 | 0 | 0 | 0 |
| Heilongjiang | 39 | 31 | 4 | 3 | 34 | 26 | 3 | 2 | 6 | 4 | 1 | 0 | 0 | 0 | 0 | 0 |
| Henan | 169 | 127 | 2 | 2 | 147 | 111 | 2 | 1 | 21 | 16 | 0 | 0 | 0 | 0 | 0 | 0 |
| Hubei | 52 | 40 | 2 | 1 | 48 | 38 | 2 | 1 | 4 | 3 | 0 | 0 | 0 | 0 | 0 | 0 |
| Hunan | 68 | 56 | 2 | 1 | 64 | 52 | 1 | 1 | 5 | 4 | 0 | 0 | 0 | 0 | 0 | 0 |
| Inner Mongolia | 64 | 63 | 6 | 6 | 56 | 55 | 5 | 5 | 8 | 7 | 1 | 1 | 0 | 0 | 0 | 0 |
| Jiangsu | 29 | 35 | 1 | 1 | 26 | 31 | 1 | 1 | 3 | 4 | 0 | 0 | 0 | 0 | 0 | 0 |
| Jiangxi | 194 | 162 | 6 | 5 | 179 | 149 | 6 | 5 | 16 | 13 | 1 | 0 | 0 | 0 | 0 | 0 |
| Jilin | 43 | 38 | 4 | 4 | 37 | 33 | 4 | 3 | 6 | 5 | 1 | 1 | 0 | 0 | 0 | 0 |
| Liaoning | 19 | 17 | 1 | 1 | 17 | 14 | 1 | 1 | 2 | 2 | 0 | 0 | 0 | 0 | 0 | 0 |
| Ningxia | 37 | 36 | 8 | 8 | 34 | 34 | 7 | 7 | 3 | 3 | 1 | 1 | 0 | 0 | 0 | 0 |
| Qinghai | 66 | 63 | 17 | 17 | 60 | 58 | 16 | 15 | 5 | 5 | 1 | 1 | 0 | 0 | 0 | 0 |
| Shaanxi | 154 | 133 | 8 | 7 | 138 | 120 | 7 | 6 | 15 | 13 | 1 | 1 | 0 | 0 | 0 | 0 |
| Shandong | 50 | 46 | 1 | 1 | 42 | 39 | 1 | 1 | 8 | 7 | 0 | 0 | 0 | 0 | 0 | 0 |
| Shanghai | 15 | 7 | 2 | 1 | 14 | 6 | 1 | 1 | 1 | 1 | 0 | 0 | 0 | 0 | 0 | 0 |
| Shanxi | 128 | 112 | 7 | 6 | 114 | 100 | 6 | 5 | 14 | 12 | 1 | 1 | 0 | 0 | 0 | 0 |
| Sichuan | 232 | 164 | 6 | 4 | 219 | 154 | 5 | 4 | 13 | 9 | 0 | 0 | 0 | 0 | 0 | 0 |
| Tianjin | 18 | 11 | 3 | 2 | 17 | 10 | 3 | 2 | 1 | 1 | 0 | 0 | 0 | 0 | 0 | 0 |
| Tibet | 86 | 84 | 31 | 31 | 79 | 77 | 29 | 28 | 7 | 7 | 3 | 2 | 0 | 0 | 0 | 0 |
| Xinjiang | 424 | 423 | 21 | 21 | 389 | 388 | 19 | 19 | 35 | 35 | 2 | 2 | 0 | 0 | 0 | 0 |
| Yunnan | 394 | 321 | 14 | 11 | 374 | 305 | 13 | 11 | 20 | 16 | 1 | 1 | 0 | 0 | 0 | 0 |
| Zhejiang | 42 | 34 | 2 | 1 | 38 | 30 | 1 | 1 | 4 | 4 | 0 | 0 | 0 | 0 | 0 | 0 |
| Central | 1075 | 902 | 3 | 3 | 959 | 804 | 3 | 3 | 115 | 97 | 0 | 0 | 1 | 0 | 0 | 0 |
| East | 389 | 349 | 1 | 1 | 351 | 314 | 1 | 1 | 38 | 35 | 0 | 0 | 0 | 0 | 0 | 0 |
| West | 1852 | 1636 | 8 | 7 | 1714 | 1512 | 7 | 7 | 137 | 123 | 1 | 1 | 1 | 0 | 0 | 0 |
| National | 3316 | 2888 | 4 | 4 | 3024 | 2631 | 4 | 3 | 290 | 255 | 0 | 0 | 2 | 0 | 0 | 0 |

**Webappendix 15.3 Streptococcus pneumoniae cases in Chinese children aged 1–59 months in 2017 by adopting vaccine coverage rates in 2010 and 2017**

| **Province** | **Streptococcus pneumoniae cases** | | | | **Streptococcus pneumoniae pneumonia cases** | | | | **Streptococcus pneumoniae meningitis cases** | | | | **Streptococcus pneumoniae severe NPNM cases** | | | |
| --- | --- | --- | --- | --- | --- | --- | --- | --- | --- | --- | --- | --- | --- | --- | --- | --- |
|  | **Number** | | **Rate per 100 000** | | **Number** | | **Rate per 100 000** | | **Number** | | **Rate per 100 000** | | **Number** | | **Rate per 100 000** | |
|  | **2010** | **2017** | **2010** | **2017** | **2010** | **2017** | **2010** | **2017** | **2010** | **2017** | **2010** | **2017** | **2010** | **2017** | **2010** | **2017** |
| Anhui | 23440 | 23379 | 607 | 605 | 8594 | 8572 | 222 | 222 | 294 | 293 | 8 | 8 | 334 | 333 | 9 | 9 |
| Beijing | 7994 | 7600 | 854 | 812 | 2931 | 2787 | 313 | 298 | 42 | 39 | 4 | 4 | 47 | 45 | 5 | 5 |
| Chongqing | 11858 | 11714 | 723 | 714 | 4348 | 4295 | 265 | 262 | 99 | 98 | 6 | 6 | 113 | 111 | 7 | 7 |
| Fujian | 14930 | 14825 | 602 | 598 | 5474 | 5435 | 221 | 219 | 142 | 141 | 6 | 6 | 162 | 160 | 7 | 6 |
| Gansu | 10974 | 10968 | 745 | 745 | 4023 | 4021 | 273 | 273 | 311 | 310 | 21 | 21 | 353 | 353 | 24 | 24 |
| Guangdong | 67354 | 66523 | 896 | 885 | 24694 | 24390 | 328 | 324 | 549 | 542 | 7 | 7 | 623 | 615 | 8 | 8 |
| Guangxi | 24225 | 24189 | 601 | 601 | 8882 | 8868 | 221 | 220 | 238 | 238 | 6 | 6 | 270 | 270 | 7 | 7 |
| Guizhou | 15913 | 15883 | 563 | 562 | 5834 | 5823 | 207 | 206 | 137 | 137 | 5 | 5 | 156 | 156 | 6 | 6 |
| Hainan | 4446 | 4410 | 674 | 669 | 1630 | 1617 | 247 | 245 | 88 | 87 | 13 | 13 | 100 | 99 | 15 | 15 |
| Hebei | 34657 | 34605 | 670 | 669 | 12706 | 12687 | 245 | 245 | 833 | 831 | 16 | 16 | 946 | 944 | 18 | 18 |
| Heilongjiang | 9248 | 9173 | 842 | 835 | 3391 | 3363 | 309 | 306 | 145 | 144 | 13 | 13 | 165 | 163 | 15 | 15 |
| Henan | 36935 | 36801 | 498 | 496 | 13542 | 13493 | 183 | 182 | 606 | 604 | 8 | 8 | 688 | 686 | 9 | 9 |
| Hubei | 18419 | 18328 | 571 | 568 | 6753 | 6720 | 209 | 208 | 123 | 122 | 4 | 4 | 139 | 139 | 4 | 4 |
| Hunan | 23173 | 23108 | 522 | 521 | 8496 | 8472 | 191 | 191 | 119 | 119 | 3 | 3 | 135 | 135 | 3 | 3 |
| Inner Mongolia | 8772 | 8772 | 836 | 835 | 3216 | 3216 | 306 | 306 | 193 | 193 | 18 | 18 | 219 | 219 | 21 | 21 |
| Jiangsu | 23921 | 23698 | 625 | 619 | 8770 | 8688 | 229 | 227 | 108 | 107 | 3 | 3 | 123 | 122 | 3 | 3 |
| Jiangxi | 19131 | 19089 | 613 | 611 | 7014 | 6999 | 225 | 224 | 459 | 458 | 15 | 15 | 522 | 521 | 17 | 17 |
| Jilin | 8783 | 8762 | 920 | 918 | 3220 | 3213 | 337 | 336 | 167 | 166 | 17 | 17 | 190 | 189 | 20 | 20 |
| Liaoning | 12413 | 12264 | 883 | 872 | 4551 | 4497 | 324 | 320 | 61 | 60 | 4 | 4 | 69 | 69 | 5 | 5 |
| Ningxia | 3111 | 3103 | 690 | 688 | 1140 | 1138 | 253 | 252 | 66 | 66 | 15 | 15 | 75 | 75 | 17 | 17 |
| Qinghai | 2968 | 2968 | 788 | 788 | 1088 | 1088 | 289 | 289 | 118 | 118 | 31 | 31 | 134 | 134 | 36 | 36 |
| Shaanxi | 13193 | 13118 | 660 | 656 | 4837 | 4810 | 242 | 240 | 355 | 353 | 18 | 18 | 403 | 401 | 20 | 20 |
| Shandong | 31383 | 31314 | 517 | 515 | 11506 | 11481 | 189 | 189 | 242 | 242 | 4 | 4 | 275 | 274 | 5 | 5 |
| Shanghai | 8349 | 7853 | 874 | 822 | 3061 | 2879 | 321 | 301 | 47 | 44 | 5 | 5 | 54 | 50 | 6 | 5 |
| Shanxi | 12908 | 12895 | 698 | 698 | 4733 | 4728 | 256 | 256 | 304 | 304 | 16 | 16 | 346 | 345 | 19 | 19 |
| Sichuan | 36183 | 35875 | 871 | 863 | 13266 | 13153 | 319 | 316 | 407 | 403 | 10 | 10 | 462 | 458 | 11 | 11 |
| Tianjin | 4825 | 4743 | 837 | 823 | 1769 | 1739 | 307 | 302 | 44 | 43 | 8 | 7 | 50 | 49 | 9 | 8 |
| Tibet | 2738 | 2738 | 1003 | 1003 | 1004 | 1004 | 368 | 368 | 33 | 33 | 12 | 12 | 37 | 37 | 14 | 14 |
| Xinjiang | 14306 | 14296 | 703 | 703 | 5245 | 5241 | 258 | 258 | 291 | 291 | 14 | 14 | 331 | 330 | 16 | 16 |
| Yunnan | 27858 | 27765 | 979 | 976 | 10214 | 10180 | 359 | 358 | 493 | 491 | 17 | 17 | 560 | 558 | 20 | 20 |
| Zhejiang | 22832 | 22278 | 850 | 829 | 8371 | 8168 | 312 | 304 | 149 | 146 | 6 | 5 | 169 | 165 | 6 | 6 |
| Central | 191141 | 190550 | 601 | 599 | 70079 | 69862 | 220 | 220 | 3138 | 3129 | 10 | 10 | 3564 | 3554 | 11 | 11 |
| East | 194002 | 191099 | 733 | 722 | 71128 | 70063 | 269 | 265 | 1384 | 1364 | 5 | 5 | 1572 | 1550 | 6 | 6 |
| West | 172098 | 171388 | 743 | 740 | 63097 | 62837 | 273 | 271 | 2741 | 2731 | 12 | 12 | 3113 | 3102 | 13 | 13 |
| National | 557241 | 553037 | 684 | 679 | 204304 | 202762 | 251 | 249 | 7262 | 7225 | 9 | 9 | 8248 | 8206 | 10 | 10 |

**Webappendix 15.4 Haemophilus influenzae type b cases in Chinese children aged 1–59 months in 2017 by adopting vaccine coverage rates in 2010 and 2017**

| **Province** | **Hib cases** | | | | **Hib pneumonia cases** | | | | **Hib meningitis cases** | | | | **Hib severe NPNM cases** | | | |
| --- | --- | --- | --- | --- | --- | --- | --- | --- | --- | --- | --- | --- | --- | --- | --- | --- |
|  | **Number** | | **Rate per 100 000** | | **Number** | | **Rate per 100 000** | | **Number** | | **Rate per 100 000** | | **Number** | | **Rate per 100 000** | |
|  | **2010** | **2017** | **2010** | **2017** | **2010** | **2017** | **2010** | **2017** | **2010** | **2017** | **2010** | **2017** | **2010** | **2017** | **2010** | **2017** |
| Anhui | 11631 | 9966 | 301 | 258 | 2043 | 1751 | 53 | 45 | 221 | 189 | 6 | 5 | 75 | 64 | 2 | 2 |
| Beijing | 3774 | 3276 | 403 | 350 | 663 | 575 | 71 | 61 | 30 | 26 | 3 | 3 | 10 | 9 | 1 | 1 |
| Chongqing | 5485 | 4227 | 334 | 258 | 963 | 743 | 59 | 45 | 71 | 55 | 4 | 3 | 24 | 19 | 1 | 1 |
| Fujian | 7130 | 6952 | 287 | 280 | 1252 | 1221 | 50 | 49 | 97 | 95 | 4 | 4 | 33 | 32 | 1 | 1 |
| Gansu | 6993 | 6685 | 475 | 454 | 1228 | 1174 | 83 | 80 | 329 | 314 | 22 | 21 | 111 | 106 | 8 | 7 |
| Guangdong | 24875 | 22747 | 331 | 303 | 4370 | 3996 | 58 | 53 | 277 | 254 | 4 | 3 | 94 | 86 | 1 | 1 |
| Guangxi | 12439 | 10905 | 309 | 271 | 2185 | 1916 | 54 | 48 | 185 | 162 | 5 | 4 | 62 | 55 | 2 | 1 |
| Guizhou | 9809 | 8060 | 347 | 285 | 1723 | 1416 | 61 | 50 | 127 | 104 | 4 | 4 | 43 | 35 | 2 | 1 |
| Hainan | 2583 | 2070 | 392 | 314 | 454 | 364 | 69 | 55 | 78 | 63 | 12 | 9 | 26 | 21 | 4 | 3 |
| Hebei | 19116 | 17463 | 369 | 337 | 3358 | 3068 | 65 | 59 | 701 | 640 | 14 | 12 | 236 | 216 | 5 | 4 |
| Heilongjiang | 5349 | 4157 | 487 | 378 | 940 | 730 | 86 | 66 | 126 | 98 | 11 | 9 | 42 | 33 | 4 | 3 |
| Henan | 19081 | 14348 | 257 | 193 | 3352 | 2520 | 45 | 34 | 481 | 362 | 6 | 5 | 162 | 122 | 2 | 2 |
| Hubei | 7879 | 6126 | 244 | 190 | 1384 | 1076 | 43 | 33 | 82 | 64 | 3 | 2 | 28 | 21 | 1 | 1 |
| Hunan | 13589 | 11036 | 306 | 249 | 2387 | 1939 | 54 | 44 | 105 | 85 | 2 | 2 | 35 | 29 | 1 | 1 |
| Inner Mongolia | 5128 | 5060 | 488 | 482 | 901 | 889 | 86 | 85 | 171 | 169 | 16 | 16 | 58 | 57 | 5 | 5 |
| Jiangsu | 11103 | 13543 | 290 | 354 | 1950 | 2379 | 51 | 62 | 71 | 86 | 2 | 2 | 24 | 29 | 1 | 1 |
| Jiangxi | 9467 | 7900 | 303 | 253 | 1663 | 1388 | 53 | 44 | 354 | 295 | 11 | 9 | 119 | 99 | 4 | 3 |
| Jilin | 4943 | 4419 | 518 | 463 | 868 | 776 | 91 | 81 | 137 | 123 | 14 | 13 | 46 | 41 | 5 | 4 |
| Liaoning | 7312 | 6228 | 520 | 443 | 1284 | 1094 | 91 | 78 | 55 | 46 | 4 | 3 | 18 | 16 | 1 | 1 |
| Ningxia | 1879 | 1869 | 417 | 414 | 330 | 328 | 73 | 73 | 61 | 61 | 14 | 14 | 21 | 21 | 5 | 5 |
| Qinghai | 1920 | 1829 | 510 | 486 | 337 | 321 | 90 | 85 | 115 | 109 | 30 | 29 | 39 | 37 | 10 | 10 |
| Shaanxi | 8362 | 7257 | 418 | 363 | 1469 | 1275 | 73 | 64 | 344 | 298 | 17 | 15 | 116 | 101 | 6 | 5 |
| Shandong | 14915 | 13782 | 246 | 227 | 2620 | 2421 | 43 | 40 | 173 | 160 | 3 | 3 | 58 | 54 | 1 | 1 |
| Shanghai | 3222 | 1511 | 337 | 158 | 566 | 265 | 59 | 28 | 26 | 12 | 3 | 1 | 9 | 4 | 1 | 0 |
| Shanxi | 8084 | 7098 | 437 | 384 | 1420 | 1247 | 77 | 67 | 312 | 274 | 17 | 15 | 105 | 92 | 6 | 5 |
| Sichuan | 17241 | 12150 | 415 | 292 | 3029 | 2134 | 73 | 51 | 303 | 213 | 7 | 5 | 102 | 72 | 2 | 2 |
| Tianjin | 2406 | 1451 | 418 | 252 | 423 | 255 | 73 | 44 | 33 | 20 | 6 | 3 | 11 | 7 | 2 | 1 |
| Tibet | 1771 | 1732 | 649 | 634 | 311 | 304 | 114 | 111 | 32 | 31 | 12 | 11 | 11 | 10 | 4 | 4 |
| Xinjiang | 9087 | 9075 | 447 | 446 | 1596 | 1594 | 78 | 78 | 289 | 289 | 14 | 14 | 97 | 97 | 5 | 5 |
| Yunnan | 16860 | 13772 | 593 | 484 | 2962 | 2419 | 104 | 85 | 443 | 362 | 16 | 13 | 149 | 122 | 5 | 4 |
| Zhejiang | 10611 | 8433 | 395 | 314 | 1864 | 1481 | 69 | 55 | 101 | 80 | 4 | 3 | 34 | 27 | 1 | 1 |
| Central | 101722 | 84582 | 320 | 266 | 17868 | 14858 | 56 | 47 | 2596 | 2192 | 8 | 7 | 875 | 739 | 3 | 2 |
| East | 85349 | 77924 | 323 | 294 | 14992 | 13688 | 57 | 52 | 862 | 779 | 3 | 3 | 291 | 263 | 1 | 1 |
| West | 96975 | 82621 | 419 | 357 | 17035 | 14513 | 74 | 63 | 2469 | 2168 | 11 | 9 | 832 | 731 | 4 | 3 |
| National | 284046 | 245127 | 349 | 301 | 49895 | 43059 | 61 | 53 | 5928 | 5139 | 7 | 6 | 1998 | 1732 | 2 | 2 |

**Webappendix 16 Pathogen-specific disease burden in Chinese children aged 1–59 months in 2018 and 2019 at the national level**

We used national-level all-cause disease burden in 2018 and 2019 to calculate more contemporary Streptococcus pneumoniae and Haemophilus influenzae type b disease burden estimates. The year 2019 is the latest year before the large-scale outbreak of COVID-19 pandemic and the most updated year with all-cause disease burden data from GBD IHME. The following table reported Streptococcus pneumoniae and Haemophilus influenzae type b deaths and cases at the national level in 2018 and 2019. We also displayed the disease burden in 2017 at the national level as a reference. It was found that the estimated pneumococcal deaths, Hib deaths, pneumococcal cases and Hib cases at the national level decreased from 8 010, 2 888, 553 037 and 245 127 in 2017 to 5 897, 1 979, 511 093 and 208 181 in 2019, respectively.

| **Year** | **Measure ^*^** | **Total** | | | | | | **Pneumonia** | | | | | | **Meningitis** | | | | | | **Severe NPNM** | | | | | |  |  |
| --- | --- | --- | --- | --- | --- | --- | --- | --- | --- | --- | --- | --- | --- | --- | --- | --- | --- | --- | --- | --- | --- | --- | --- | --- | --- | --- | --- |
|  |  | **Number** | | | **Rate per 100 000** | | | **Number** | | | **Rate per 100 000** | | | **Number** | | | **Rate per 100 000** | | | **Number** | | | **Rate per 100 000** | | | |  |
|  |  | **Mean** | **UR** | | **Mean** | **UR** | | **Mean** | **UR** | | **Mean** | **UR** | | **Mean** | **UR** | | **Mean** | **UR** | | **Mean** | **UR** | | **Mean** | **UR** | |  |  |
| 2017 | Spn deaths | 8010 | 5535 | 8912 | 10 | 7 | 11 | 6253 | 4434 | 6517 | 8 | 5 | 8 | 929 | 582 | 1267 | 1 | 1 | 2 | 827 | 519 | 1128 | 1 | 1 | 1 | | |
| 2018 | Spn deaths | 6933 | 4769 | 7701 | 9 | 6 | 10 | 5418 | 3842 | 5646 | 7 | 5 | 7 | 802 | 491 | 1087 | 1 | 1 | 1 | 714 | 437 | 968 | 1 | 1 | 1 | | |
| 2019 | Spn deaths | 5897 | 4077 | 6555 | 7 | 5 | 8 | 4622 | 3277 | 4817 | 6 | 4 | 6 | 675 | 423 | 920 | 1 | 1 | 1 | 601 | 376 | 819 | 1 | 0 | 1 | | |
| 2017 | Hib deaths | 2888 | 1966 | 3900 | 4 | 2 | 5 | 2631 | 1850 | 3457 | 3 | 2 | 4 | 255 | 115 | 440 | 0 | 0 | 1 | 2 | 1 | 4 | 0 | 0 | 0 | | |
| 2018 | Hib deaths | 2326 | 1588 | 3152 | 3 | 2 | 4 | 2120 | 1491 | 2785 | 3 | 2 | 3 | 205 | 97 | 364 | 0 | 0 | 0 | 2 | 1 | 3 | 0 | 0 | 0 | | |
| 2019 | Hib deaths | 1979 | 1348 | 2670 | 2 | 2 | 3 | 1807 | 1271 | 2375 | 2 | 2 | 3 | 170 | 77 | 293 | 0 | 0 | 0 | 1 | 1 | 2 | 0 | 0 | 0 | | |
| 2017 | Spn cases | 553037 | 477172 | 657405 | 679 | 586 | 807 | 202762 | 151908 | 231270 | 249 | 187 | 284 | 7225 | 4510 | 9793 | 9 | 6 | 12 | 8206 | 5123 | 11123 | 10 | 6 | 14 | | |
| 2018 | Spn cases | 538885 | 464962 | 640583 | 675 | 582 | 802 | 197574 | 148021 | 225352 | 247 | 185 | 282 | 6206 | 3817 | 8377 | 8 | 5 | 10 | 7049 | 4335 | 9515 | 9 | 5 | 12 | | |
| 2019 | Spn cases | 511093 | 440982 | 607546 | 628 | 542 | 746 | 187384 | 140387 | 213730 | 230 | 172 | 263 | 5241 | 3272 | 7105 | 6 | 4 | 9 | 5953 | 3716 | 8070 | 7 | 5 | 10 | | |
| 2017 | Hib cases | 245127 | 223806 | 400514 | 301 | 275 | 492 | 43059 | 25781 | 86909 | 53 | 32 | 107 | 5139 | 2379 | 9120 | 6 | 3 | 11 | 1732 | 802 | 3074 | 2 | 1 | 4 | | |
| 2018 | Hib cases | 219624 | 200522 | 358845 | 275 | 251 | 449 | 38579 | 23099 | 77867 | 48 | 29 | 97 | 4020 | 1897 | 7227 | 5 | 2 | 9 | 1355 | 639 | 2436 | 2 | 1 | 3 | | |
| 2019 | Hib cases | 208181 | 190073 | 340147 | 256 | 233 | 418 | 36569 | 21896 | 73810 | 45 | 27 | 91 | 3397 | 1570 | 6020 | 4 | 2 | 7 | 1145 | 529 | 2029 | 1 | 1 | 2 | | |
| ^*^ Spn, Streptococcus pneumoniae; Hib, Haemophilus influenzae type b. | | | | | | | | | | | | | | | | | | | | | | | | | | | |
